# Spatial Organisation of Tumour cDC1 States Correlates with Effector and Stem‐Like CD8^+^ T Cells Location

**DOI:** 10.1002/eji.70011

**Published:** 2025-07-31

**Authors:** Cécile Piot, Mariana Pereira da Costa, Adi Biram, Carlos M. Minutti, Kok Haw Jonathan Lim, Mary Green, Ania Mikolajczak, Robert P. Jenkins, Lucy Meader, Michael D. Buck, Ana Cardoso, Neil Rogers, Erik Sahai, Caetano Reis e Sousa

**Affiliations:** ^1^ Immunobiology Laboratory The Francis Crick Institute London UK; ^2^ Immunoregulation Laboratory Champalimaud Research Champalimaud Centre for the Unknown Lisbon Portugal; ^3^ Division of Cancer Sciences Faculty of Biology Medicine and Health The University of Manchester; and Advanced Immunotherapy and Cell Therapy Team The Christie NHS Foundation Trust Manchester UK; ^4^ Experimental Histopathology The Francis Crick Institute London UK; ^5^ Tumour Cell Biology Laboratory The Francis Crick Institute London UK; ^6^ Medical Department ADM Health & Wellness London UK

**Keywords:** CD8 T cells, dendritic cells, spatial, tumour immunology

## Abstract

CD8^+^ T cells are central to targeting and eliminating cancer cells. Their function is critically supported by type 1 conventional dendritic cells (cDC1s), which both prime antigen‐specific CD8^+^ T cells in tumour‐draining lymph nodes (tdLNs) and sustain primed CD8^+^ T cells within tumours. Despite their importance, the spatiotemporal organisation of cDC1s within tumours and their diverse functional roles remain poorly understood. Here, we use scRNAseq and unbiased spatial analysis to construct a detailed map of cDC1 states and distribution within immunogenic mouse tumours during CD8^+^ T‐cell‐mediated rejection. We reveal two distinct cDC1 activation states characterised by differential expression of genes linked to anti‐tumour immunity, including *Cxcl9* and *Il12b*. Strikingly, *Il12b*‐expressing cDC1s are CCR7^+^ and enriched at tumour borders, where they closely associate with stem‐like TCF1^+^ CD8^+^ T cells. In contrast, CCR7^–^ *Cxcl9*‐expressing cDC1s are preferentially found within the tumour parenchyma alongside effector CD8^+^ T cells. Analysis of a published dataset of human tumours similarly reveals a spatial association between CCR7^+^ cDC1 and stem‐like TCF1^+^ CD8^+^ T cells. These findings uncover a highly spatially coordinated interaction between cDC1s and CD8^+^ T cells within tumours, shedding light on the intricate cellular dynamics that underpin effective anti‐tumour immunity.

## Graphical abstract

Using scRNAseq and spatial analysis, we analyse cDC1 states and spatial distribution in tumours during immune‐mediated rejection. We identify two cDC1 activation states, each occupying different regions and associated with distinct CD8^+^ T cell populations. This reveals the spatial organisation of cDC1 states that may be key to anti‐tumour immunity.

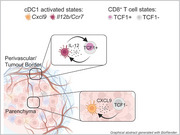

**Abbreviations**
cDCs: conventional dendritic cells
DAMPs: damage‐associated molecular patterns
HCC: hepatocellular carcinoma
i.v.: intravenous
ISGs: interferon‐stimulated genes
PAMPs: pathogen‐associated molecular patterns
RSNs: raster scanned neighbourhoods
s.c.: subcutaneous
tdLNs: tumour‐draining lymph nodes
TME: tumour microenvironment

## 1 Introduction

Conventional dendritic cells (cDCs) are innate immune cells that play key roles in maintaining tissue homeostasis and in surveying the body for threats arising from infection and malignancy. cDCs are divided into two major subsets, type 1 and 2 cDCs (cDC1s and cDC2s, respectively) that differ in expression of innate immune receptors, antigen‐presentation capacity and other properties. As such, they can display differential abilities to prime T cells in tissue‐draining lymph nodes and to orchestrate different types of immune responses.

In the context of anti‐tumour immunity, numerous mouse studies have established a key role for cDC1s, notably in their ability to cross‐present tumour‐derived antigens to CD8^+^ T cells and elicit cytotoxic responses against tumour cells [1, 2]. cDC1s express a range of proteins that have been shown to be involved in the cross‐presentation of cellular antigens, such as DNGR‐1 (a.k.a CLEC9A) and WDFY4, both of which are crucial for anti‐tumour immunity in murine tumour models [3, 4]. cDC1s have also been shown to play key roles locally within the tumour microenvironment (TME), for example, by recruiting and coordinating the localisation and survival of intratumoural CD8^+^ T cells via production of chemokines, cytokines and local antigen presentation [5, 6, 7, 8, 9]. In human tumours, cDC1 abundance and cDC1‐associated gene expression signatures are associated with enhanced CD8^+^ T cell‐dependent tumour control [9, 10, 11, 12].

cDCs are short‐lived cells that need to be replenished by bone marrow (BM)‐derived cDC progenitors (pre‐cDCs). Pre‐cDCs enter tissues via the blood to give rise to differentiated tissue‐resident cDCs. These cDCs are largely quiescent but can become “activated” or “mature” in response to pathogen‐associated or damage‐associated molecular patterns (PAMPs and DAMPs, respectively), as well as inflammatory stimuli associated with infection, carcinogenesis or tissue damage [13]. cDC activation often manifests in enhanced antigen presentation, upregulation of co‐stimulatory molecules, production of cytokines (e.g., IL‐12) and upregulation of chemokine receptors such as CCR7, allowing cDCs to migrate to tissue‐draining LNs and instruct different types of T cell responses. While all forms of cDC activation are accompanied by phenotypic and gene expression changes, the nature of the stimulus can give rise to different “flavours” of activated cDCs [13, 14]. This includes so‐called “homeostatic maturation”, which occurs at steady state in the absence of PAMP signals and, in the case of cDC1s, is now known to be triggered by efferocytosis and consequent accumulation of cholesterol derived from apoptotic corpses [15]. “Homeostatically‐mature” cDCs upregulate CCR7 and MHC class II like their PAMP‐activated counterparts but exhibit lower expression of multiple genes associated with inflammatory responses, such as interferon‐stimulated genes (ISGs) [15, 16]. It is often assumed that PAMP‐activated cDCs are “immunogenic” while homeostatically matured cDCs are “tolerogenic” although this remains to be proven. It is likely that different cDC activation states have multiple functions that may be somewhat overlapping and context‐dependent.

In tumours, multiple factors have been suggested to induce or modulate cDC activation, including tumour‐derived DNA, type I interferons, uptake of tumour‐derived material, oxidised lipids and prostaglandin E2, to name but a few [17, 18, 19, 20, 21, 22]. Activated cDCs have been studied in human and murine tumours by scRNAseq. They include a population variably referred to as “mregDC” (for mature DCs enriched in immunoregulatory molecules), “DC3” or *LAMP3^+^* DCs [20, 23, 24]. While “mregDCs”, “DC3” or *LAMP3^+^* DCs are confusingly sometimes described as a cDC subset, it is clear that they represent an activation state that can be assumed by both cDC1s and cDC2s and is marked by expression of *Ccr7* and other canonical transcripts associated with cDC activation. CCR7 expression is important for cDC migration to the tumour‐draining lymph node [25], but, interestingly, recent studies have suggested that CCR7^+^ cDCs can also remain in tumours and perform local functions [6, 7, 26]. CCR7^+^ cDCs in tumours also produce IL‐12, which was shown to be important for reinvigorating the CD8^+^ T cell response during immune checkpoint blockade [27]. It remains unclear how activated CCR7^+^ cDC in tumours differ from CCR7^+^ cDCs observed in the steady‐state or other inflammatory contexts.

cDCs in tumours can also express interferon‐stimulated genes (ISGs). ISG‐expressing cDC2s have been shown to present tumour antigens to CD8^+^ T cells via a process called “cross‐dressing”, where they acquire intact peptide:MHC‐I complexes from cancer cells [28, 29]. ISGs expressed by some tumour‐associated cDC1s include CXCL9 and CXCL10, which are important for the recruitment and localisation of CD8^+^ T cells [8, 9, 28]. Which signals induce this cellular state in tumours remains unclear, but CXCL9 expression by cDC1s can be augmented therapeutically by increasing uptake of tumour‐derived DNA in response to TIM‐3 blockade [30].

The extent of overlap between CXCL9/CXCL10‐expressing and CCR7‐expressing tumour‐associated cDC1s, whether they represent end‐states or steps along the activation process and whether they have different functions, remains unclear. One approach to this issue is to map cDC1 states determined by scRNAseq to discrete territories within the tumour and characterise other cells in the vicinity. Indeed, despite their central role in anti‐tumour immunity, relatively little is known about how cDC1s are spatially organised within tumours to perform local functions and how these functions mirror cellular states. Here, we set out to characterise the distribution and nature of cDC1s within the tumour microenvironment. Using an immunogenic fibrosarcoma model, we found that a large fraction of cDC1s accumulates in regions rich in blood vessels and CD8^+^ T cells at the tumour borders. Two distinct cDC1 activation states are differentially associated with *Cxcl9* and *Il12b/Ccr7* expression, respectively, each localising to different regions of the tumour, with the *Cxcl9* ^+^ cDC1s being enriched in the parenchyma while the *Il12b* ^+^ cDC1s remain located at the edges. Additionally, we found that stem‐like TCF1^+^ CD8^+^ T cells are enriched in tumour‐bordering regions in association with the *Il12b/Ccr7*‐expressing cDC1s, while effector CD8^+^ T cells were enriched in the parenchyma in proximity to *Cxcl9* ^+^ cDC1s. Lastly, analysis of a published spatial transcriptomic dataset of human tumours revealed that *CCR7* ^+^ cDC1s, the human equivalent of the murine *Il12b* ^+^ cDC1s, are also preferentially associated with TCF1^+^ T cells as compared with the *CXCL9^+^* cDC1s. Overall, these results suggest that distinct cDC1 and CD8^+^ T cell states can occupy discrete territories within tumours, which may have functional implications for tumour control.

## 2 Results

### 2.1 Distribution of cDC1s in Immunogenic Tumours

**FIGURE 1:**
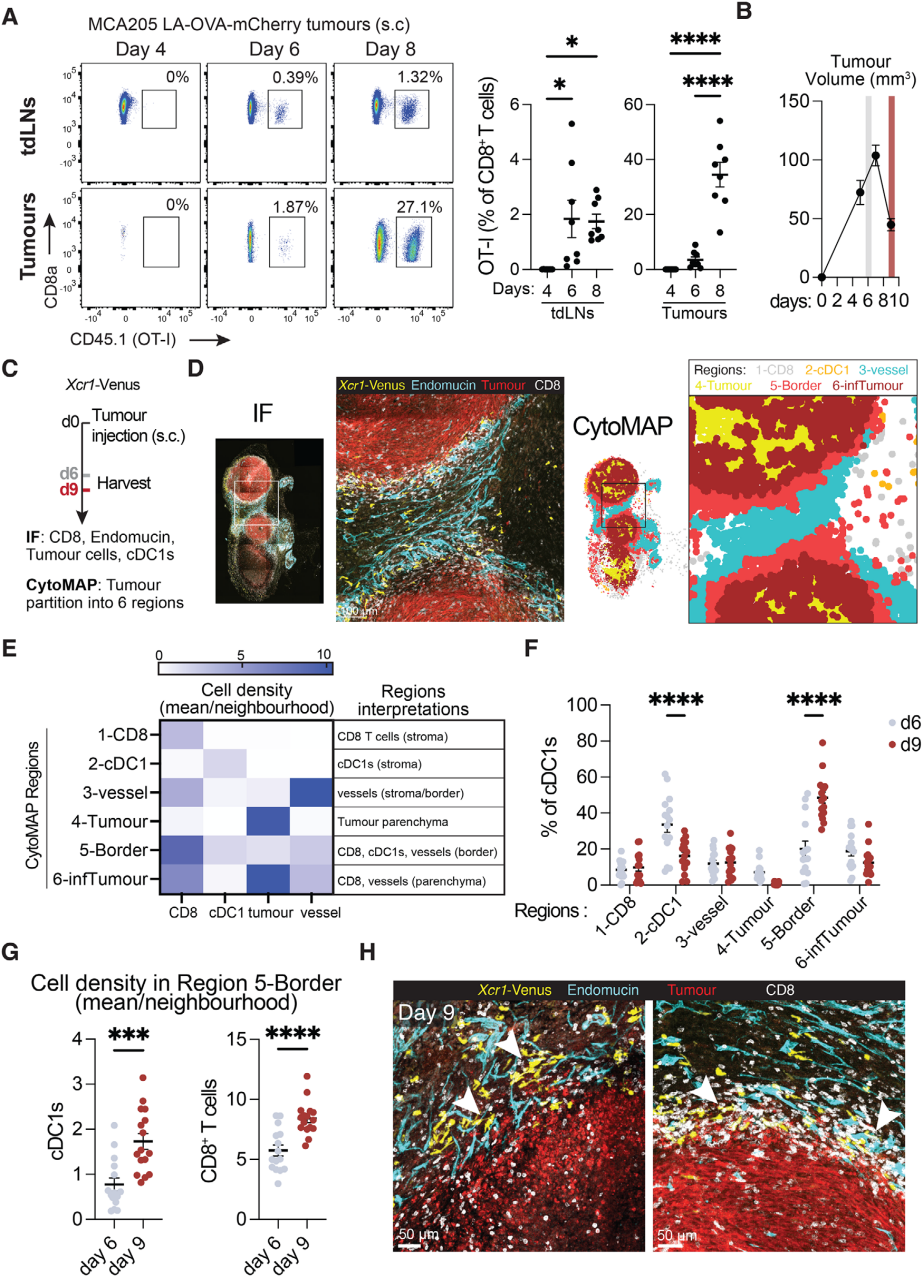
Distribution of cDC1s in immunogenic tumours. **(A)** Kinetics of OVA‐specific CD8^+^ T cell (OT‐I) priming in tdLNs and expansion within MCA205 LA‐OVA‐mCherry tumours. Naïve OT‐I CD8^+^ T cells were transferred i.v. into WT mice, and tumours were injected on the same day. TdLNs and tumours were harvested 4, 6 and 8 days later for flow cytometry analysis. Representative flow cytometry dot plots of CD8^+^ T cells (left) show the percentage of OT‐I cells amongst CD8^+^ T cells in tdLNs (upper row) and tumours (lower row) on days 4, 6 and 8. Quantifications of the flow cytometry data (right) show the percentage of OT‐I in tdLNs and tumours. Statistical analyses were performed using ordinary one‐way ANOVA comparing the mean of each time point. *n* = 8 per time point, from two experiments. **(B)** Representative tumour growth profile of subcutaneous MCA205‐LA‐OVA‐mCherry tumours up to day 9. Grey and red bars represent the time points selected to analyse cDC1 distribution in **(C)** (day 6 and day 9, respectively). *n* = 10 from one experiment. **(C)** Experimental strategy to characterise cDC1 spatiotemporal distribution in tumour. MCA205 LA‐OVA‐mCherry tumours were injected s.c. in *Xcr1‐*Venus mice and collected 6 or 9 days after for imaging cDC1s (Venus^+^ cells), CD8^+^ T cells (CD8^+^), blood vessels (Endomucin^+^) and tumour cells (mCherry^+^). 10–30 µm‐thick whole tissue sections were stained, imaged and segmented from independent tumour samples. CytoMAP was used to divide images into 50 µm‐radius raster scanned neighbourhoods (RSN). The RSN were classified into six regions based on the relative number of cDC1s, CD8^+^ T cells, vessels and tumour cells per RSN. IF = immunofluorescence. **(D)** Representative IF of a whole tumour section showing cDC1s (Venus^+^), CD8^+^ T cells (CD8^+^), vessels (endomucin^+^) and tumour cells (mCherry^+^) (left) and the corresponding 6 CytoMAP regions (right) generated based on the density (number of cells per neighbourhood) of the four cell types. **(E)** Heatmap showing the composition of the CytoMAP regions as mean number of CD8^+^ T cells, cDC1s, tumour cells and blood vessels per RSN neighbourhood classified as Regions 1–6. Regions’ interpretations are described on the right of the heatmap. Heatmap representative of three experiments with at least nine independent whole tumour sections per experiment. **(F)** cDC1 distribution across CytoMAP regions on days 6 and 9 as % of cDC1s per tumour section. All cDC1s of one tumour section were classified into one of the six CytoMAP regions, and the percentage of cDC1s associated with either region was generated for each section. Each dot represents a different whole tumour section. Data are pooled from three independent experiments with 4–6 independent whole tumour sections per time point and per experiment. Statistical analysis was performed using a two‐way ANOVA test and comparing the mean of day 6 with the mean of day 9 for each region using Šídák's multiple comparisons test. **(G)** cDC1s (left) and CD8^+^ T cells (right) density on day 6 and day 9 in Region 5‐Border, shown as mean number of cells per RSN classified as region 5. Each dot represents a different tumour sample. Data are pooled from three independent experiments with 4–6 independent whole tumour sections per time point and per experiment. Statistical analyses were performed using a two‐tailed unpaired *t*‐test. **(H)** Two representative images of IF at the tumour border region on day 9, showing cDC1s, blood vessels and CD8^+^ T cells at the tumour border. White arrows highlight regions of cDC1s, vessels and CD8^+^ T cells at the tumour border regions.

Tumour‐associated cDC1s are important for CD8^+^ T cell priming in tumour‐draining lymph nodes (tdLNs) and for sustaining the activity of CD8^+^ T cells within tumours. To investigate the distribution of cDC1s during these two processes, we first characterised the kinetics of CD8^+^ T cell priming in tdLNs and expansion within tumours. We used the immunogenic fibrosarcoma cell line MCA205 LA‐OVA‐mCherry, which is highly dependent on cDC1 activity for rejection [3], and implanted the cancer cells subcutaneously (s.c.). Naïve OT‐I were injected intravenously (i.v.) on the day of tumour cell injection to quantify antigen‐specific CD8^+^ T cells in tdLNs and tumours (Figure S1A). Expansion of OT‐I CD8^+^ T cells in tdLNs was not patent at day 4 but became obvious by day 6, suggesting that a wave of priming by cDC1s occurred between days 4 and 6 (Figure 1A). OT‐I expansion continued rapidly thereafter, and a nearly 30‐fold increase in their proportion relative to total CD8^+^ T cells could be detected within tumours by day 8 (Figure 1A). Consistent with the kinetics of T cell expansion, tumours underwent rapid rejection from day 8 onwards (Figure 1B).

To investigate the distribution of intratumoural cDC1s during CD8^+^ T cell priming and expansion within tumours, we inoculated MCA205 LA‐OVA‐mCherry cells in *Xcr1‐*Venus mice [31] and harvested tumours on days 6 and 9 postimplantation (Figure 1C). Tumour sections were stained with antibodies to enable visualisation of CD8^+^ T cells (CD8^+^) and blood vessels (Endomucin^+^) given that some cDC1s have been reported to locate close to perivascular niches in tumours [6]. Additionally, endogenous or antibody‐amplified fluorescence was used to image tumour cells (mCherry^+^) and cDC1s (Venus^+^) (Figure 1D). To quantitatively assess the distribution of cDC1s in tumours, we used CytoMAP [32] to generate archetypal tumour‐associated regions based on the local abundance of CD8^+^ T cells, vessels, tumour cells and cDC1s (Figure 1D). Images of whole tumour sections were divided into raster scanned neighbourhoods (RSNs) of 50 µm radius, which were subsequently clustered into regions based on the cellular composition of each RSN and using a self‐organising map. To identify how many clusters should be used for the RSNs clustering, we evaluated Davies Bouldin and Calinski‐Harabasz values obtained with different numbers of clusters. Small Davies Bouldin and large Calinski‐Harabasz values correspond to better clustering results, and we identified a value of six clusters as corresponding to the “elbows” of the value plots, which marked the stabilisation of the Davies Bouldin and the Calinski‐Harabasz values to a low and a high value plateau, respectively (Figure S1B). Based on these results, the RSNs of tumour cDC1s, blood vessels, CD8^+^ T cells and tumour cells were subsequently clustered into six regions for the remainder of this study.

Using this approach, we found 6 CytoMAP regions that were common to all tumours analysed (Figure 1D). Regions 1 and 2 were largely composed solely of CD8^+^ T cells or cDC1s, respectively, and were therefore renamed “1‐CD8” and “2‐cDC1” (Figure 1E). Region 3 consisted of areas more densely populated by blood vessels and CD8^+^ T cells, while Region 4 corresponded to areas of the parenchyma with poor vascularisation and limited cDC1s and CD8^+^ T cells infiltration (Figure 1E). These were, therefore, renamed “3‐vessel” and “4‐Tumour”. Regions 5 and 6 represented regions with multiple cell types and vascularisation. Region 5 was characterised by a mix of CD8^+^ T cells, cDC1s and blood vessels at the tumour border (named “5‐Border”), whereas Region 6 corresponded to areas in the parenchyma with CD8^+^ T cells and blood vessels (named “6‐infTumour”) (Figure 1E). In line with these interpretations, the regions interaction heatmap suggested that neighbourhoods in Regions 1‐CD8 and 2‐cDC1 were mostly disconnected from the other regions, that Regions 4‐Tumour and 6‐infTumour interacted due to their proximity in the parenchyma, and that Region 5‐border shared borders with Regions 1, 2, 3 and 6 (Figure S1C).

We used these regions to examine changes in tumour composition and cDC1 localisation during initial tumour growth and rejection (days 6 and 9). Consistent with tumour rejection on day 9, we found that the fraction of neighbourhoods classified as parenchyma Region 4‐Tumour (poorly‐infiltrated in CD8^+^ T cells and cDC1s) was decreased at this time point (Figure S1D). Instead, tumours exhibited an expansion of the 5‐Border region (Figure S1D), marked by a high density of blood vessels, CD8^+^ T cells, and cDC1s. cDC1 distribution also significantly changed between days 6 and 9. On day 6, cDC1s were proportionally increased in Region 2‐cDC1, while on day 9, cDC1s were increased in Region 5‐Border (Figure 1F). cDC1s were also present in the remaining regions at lower percentages, with their distribution unchanged across these time points (Figure 1F). Interestingly, we also observed an increased density of cDC1s and CD8^+^ T cells in Region 5‐Border on day 9 (Figure 1G). This suggests that not only does this region occupy a larger proportion of the tumour, but that it also contains a higher density of cDC1s and CD8^+^ T cells. Visual inspection of tumour sections confirmed that tumour borders were characterised by high abundance of cDC1s, CD8^+^ T cells and blood vessels on day 9 (Figure 1H).

Overall, these results indicate that cDC1 localisation changes over time, which may reflect differences in cDC1 functions during CD8^+^ T cell priming versus intratumoural CD8^+^ T cell expansion. Before the emergence of primed CD8^+^ T cells, cDC1s are found preferentially sparsely in the stroma, whereas during intratumoural CD8^+^ T cells expansion, cDC1s are enriched in tumour border regions, which could help sustain that expansion.

### 2.2 cDC1s are Found in Different States in Tumours, Including Two Distinct Activation Types

To characterise putative functions and cDC1 cellular states that might be associated with these respective regions, we first characterised by scRNAseq the heterogeneity of intratumoural cDC1s. We sorted pre‐cDCs, cDC1s and cDC2s from MCA205 LA‐OVA tumours on day 5. Pre‐cDCs were included to facilitate distinction from early cDC1s, while inclusion of cDC2s helped identify activated cDC1s because the gene expression signatures of activated cDC1s and cDC2s converge upon activation [15, 20]. Tumours were harvested from wild‐type (WT) mice but also from *sGsn^−/−^* mice and *sGsn^−/−^ Clec9a^−/−^* mice to understand whether modulation of cross‐presentation in cDC1s has an impact on their cellular states. Indeed, we have previously shown that *sGsn^−/−^* mice control MCA205 LA‐OVA tumours better than WT mice because of increased DNGR‐1(CLEC9A)‐dependent cDC1‐cross presentation of dead cell‐associated tumour antigens [3].

**FIGURE 2:**
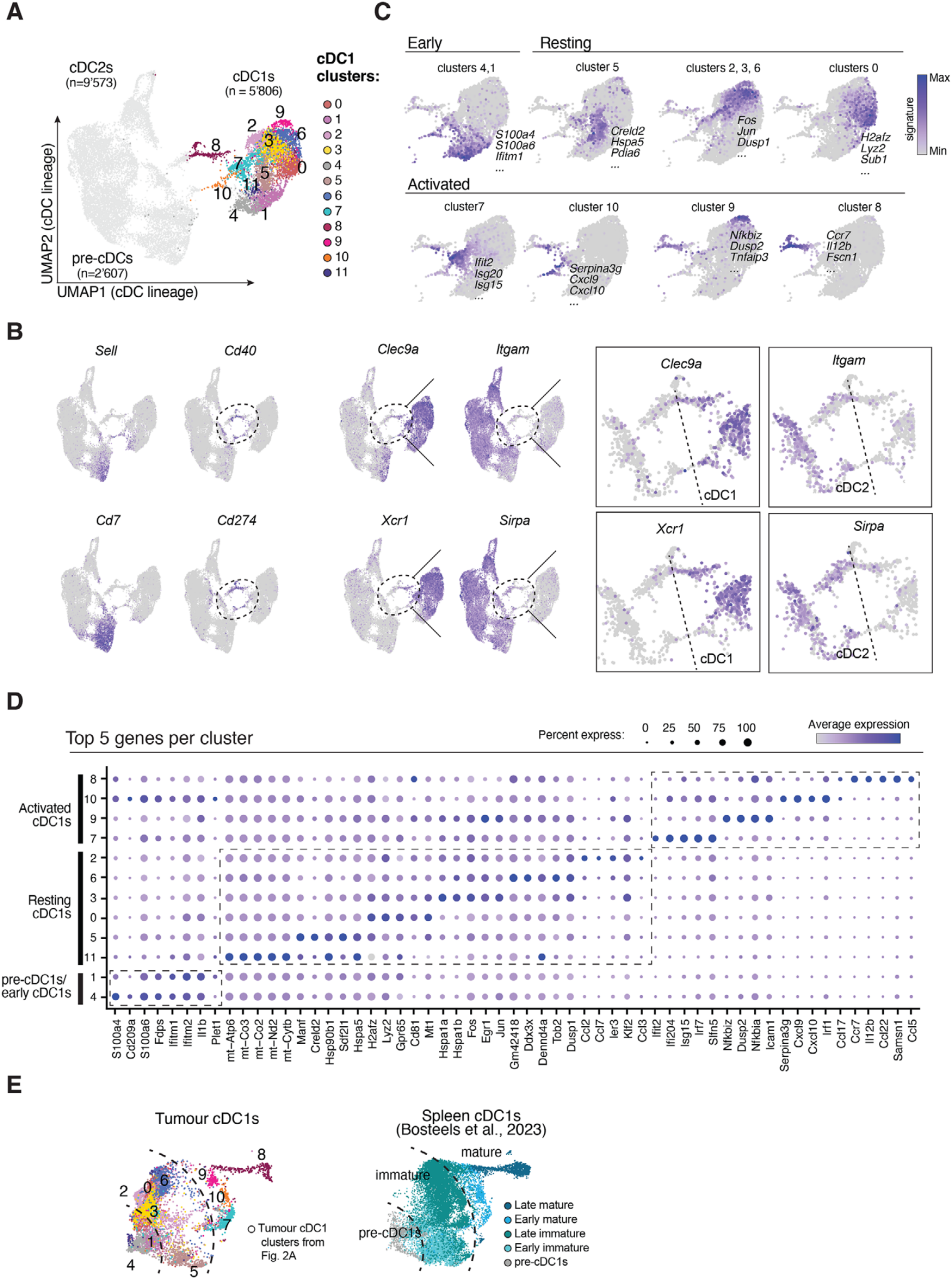
scRNAseq analysis of tumour cDCs identifies distinct cDC1 states in tumours. **(A)** UMAP representation of tumour pre‐cDCs and cDCs analysed by scRNAseq. Pre‐cDCs, cDC1s and cDC2s were FACS‐sorted from MCA205 LA‐OVA tumours and harvested on day 5, and mixed at a 1:1:2 ratio, respectively, for scRNAseq. The pre‐cDCs and cDC2s regions (bottom and left side of the UMAP, respectively) are indicated on the sides of the UMAP, as well as the 12 clusters of cDC1s identified upon re‐clustering the cDC1s separately. **(B)** Expression of selected genes used to annotate the UMAP in **(A)**. Expression of *Sell* and *Cd7* were used to identify pre‐cDCs. Expression of *Cd40* and *Cd274* identified activated cDCs, highlighted with a dotted round shape. cDC1s and cDC2s were identified based on the expression of *Xcr1, Clec9a, Itgam* and *Sirpa*. Shown on the right are close‐ups of the activated cDCs on the UMAP to identify activated cDC1s (right of the dotted line) from activated cDC2s (left of the dotted line). **(C)** Expression of gene signatures to highlight cDC1 states in tumours (early, resting and activated). “Early” cDC1 state includes clusters 4 and 1. “Resting” cDC1s include clusters 5, 2, 3, 6 and 0. “Activated” cDC1s include clusters 7, 10, 9 and 8. Gene signatures correspond to the top 10 DEGs of the indicated clusters (Table S1). Selected genes from the signatures are indicated on the UMAP. **(D)** Dot plot of top 5 genes per cDC1 clusters. The three dashed rectangles highlight genes enriched in pre‐cDC1s/early cDC1s, resting cDC1s and activated cDC1s, respectively. **(E)** UMAP of integrated splenic cDC1s from Bosteels et al. [15], and tumour cDC1s from **(A)**. UMAP on the left shows the tumour cDC1 from **(A)** in the integrated space. UMAP on the right shows splenic cDC1s coloured by cellular state as described in Bosteels et al. [15] (pre‐cDC1s, immature and mature cDCs). Dotted lines represent the transition between pre‐cDC1s, “immature” and “mature” regions of the UMAP based on the annotations from the splenic cDC1s.

Following preparation of libraries, sequencing and exclusion of dying cells, T cells, NK cells, plasmacytoid cells, and proliferating cells (Figure S2A,B), UMAP analysis identified a region of pre‐cDCs (expressing *Cd7, Sell)*, cDC1s (*Clec9a, Xcr1*) and cDC2s (*Itgam, Sirpa)* (Figure 2A,B). The UMAP1 axis largely separated cells based on commitment to either cDC subset, with the cDC2s on the left, pre‐cDCs in the middle, and cDC1s on the right. UMAP2 axis organised cells based on their differentiation stage, with the pre‐cDCs at the bottom and the cDCs at the top (Figure 2A,B). Interestingly, apart from the three main regions of pre‐cDCs, cDC1s, and cDC2s, we observed two “bridges” of cells between the cDC1s and cDC2s regions on the UMAP space (Figure 2A,B). These “bridging” cells were enriched for transcripts associated with cDC activation, such as *Cd40* and *Cd274* (Figure 2B). Consistent with previous studies reporting that activated cDC1s and cDC2s are hard to discriminate by scRNAseq [15, 20], activated cDC1s and cDC2s were found in close proximity on the UMAP space. Nevertheless, they could still be distinguished based on the expression of *Clec9a* and *Xcr1* for the cDC1s, and *Itgam* and *Sirpa* for the cDC2s (Figure 2B).

Having identified tumour cDC1s, we re‐analysed these cells separately and identified 12 clusters of cDC1 states (Figure 2A). Cells from the three genotypes contributed equally to the cDC1 clusters (Figure S2C), indicating that DNGR‐1 triggering does not influence the gene expression profile of tumour cDC1s. Therefore, downstream analyses were performed on pooled cDC1 from all three genotypes. As in other analyses [15], we grouped the cDC1 clusters into “early”, “resting” and “activated” cDC1s (Figure 2C). Early cDC1s included clusters 4 and 1, which localised close to the pre‐cDCs on the UMAP space and expressed *S100a4*, *S100a6* and *Ifitm1* (Figures 2C,D; Table S1). In line with this, clusters 4 and 1 overlapped with splenic pre‐cDC1s upon integration of the data with a scRNAseq dataset of cDC1 states identified in the spleen (Figure 2E) [15]. Resting tumour cDC1s included cluster 5 enriched in transcripts involved in the unfolded protein response (UPR) such as *Creld2, Hspa5*, and *Pdia6*, as well as the clusters 2, 3 and 6 that expressed transcripts such as *Fos, Jun*, and *Dusp1* and cluster 0 that expressed *H2afz, Lyz2, Sub1* (Figures 2C,D; Table S1). Accordingly, clusters 0, 2, 3, 5 and 6 overlapped with the “immature” splenic cDC1s on the integrated UMAP space (Figure 2E) [15]. Cluster 11 was enriched in mitochondrial gene transcripts, likely corresponding to dying cells (Figure 2D; Table S1). Lastly, four clusters of activated cDC1s were identified: clusters 7, 8, 9 and 10 (Figures 2C,D), which corresponded to “mature” cDC1s from the spleen (Figure 2E) [15]. Cluster 7 was enriched in ISGs such as *Ifit2, Isg20* and *Isg15;* cluster 10 in *Serpina3g, Cxcl9* and *Cxcl10*; cluster 9 in *Nfkbiz, Dusp2* and *Tnfaip3*; and cluster 8 in *Ccr7, Il12b* and *Fscn1* (Figures 2C,D; Table S1).

**FIGURE 3:**
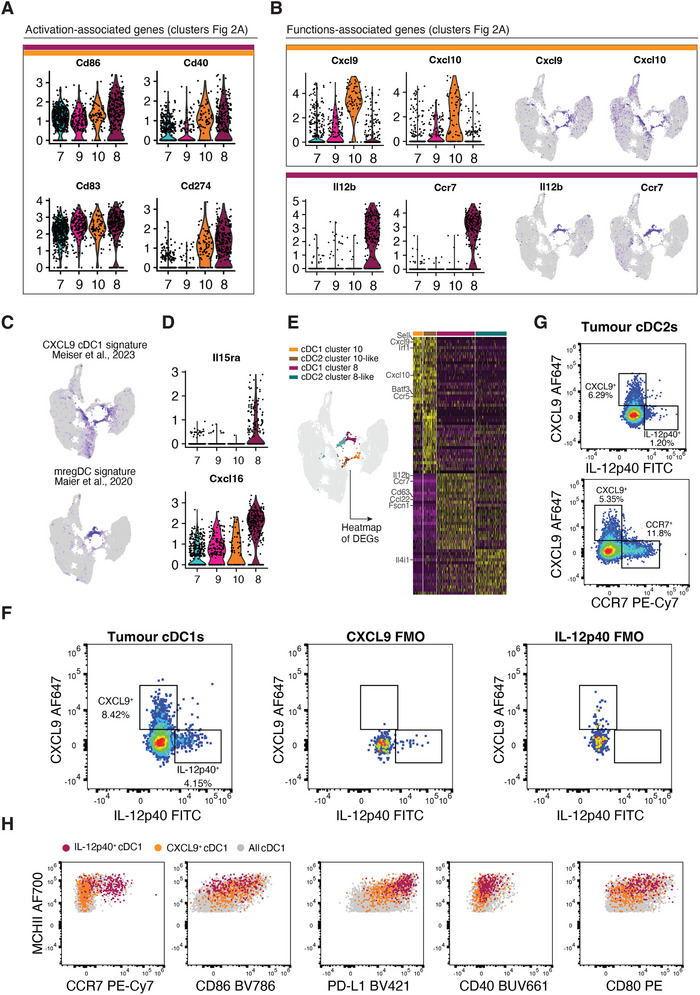
cDC1s are present in two distinct activation states in tumours. **(A)** Violin plots of the activation‐associated genes *Cd86, Cd83, Cd40* and *Cd274* (encoding for PD‐L1) on the activated cDC1 clusters (clusters 7, 9, 10 and 8) identified in Figure 2A. **(B)** Violin plots (left) and feature plots (right) of selected genes associated with cDC1 functions in tumours, including *Cxcl9*, *Cxcl10, Il12b* and *Ccr7*. **(C)** Overlay of the CXCL9 cDC1 signature from Meiser et al., 2023 (upper UMAP) and of the mregDC signature from Maier et al., 2020 (lower UMAP). **(D)** Expression of *Il15ra* and *Cxcl16* across activated cDC1 clusters, reminiscent of the CCR7‐expressing cDCs described by Di Pilato et al., 2021. **(E)** Heatmap showing the expression of *Cxcl9* cDC1 DEGs and *Il12b* cDC1s DEGs in cDC1 cluster 10 (orange), cDC2 cluster 10‐like (brown), cDC1 cluster 8 (maroon), cDC2 cluster 8‐like (teal). **(F)** Left panel: expression of CXCL9 and IL‐12p40 in tumour cDC1s by flow cytometry, as gated in Figure S3A. The data shown are a pool from 10 samples from one experiment. Representative of two experiments. Middle panel: FMO control for CXCL9 expression. Right panel: FMO control for IL‐12p40 expression. **(G)** Expression of CXCL9 and IL‐12p40 (upper panel) or CXCL9 and CCR7 expression (lower panel) in cDC2s. Data from one sample and representative of 10 samples from one experiment. **(H)** Dot plots showing expression of a selection of activation‐associated markers (MHCII, CCR7, CD86, PD‐L1, CD40, CD80) in IL‐12p40^+^ cDC1s (in maroon), CXCL9^+^ cDC1s (in orange) and in ungated cDC1s (in grey). Data pooled from 10 samples from one experiment. Representative of two experiments.

Of those, clusters 10 and 8 mapped to the lower and higher “activation bridges” on the UMAP space (Figure 2A), respectively, and expressed higher level of *Cd274* and *Cd40* (Figures 2B and 3A), suggesting that they might represent endpoint activated cDC1 states in tumours, while clusters 7 and 9 may correspond to transitional states. Interestingly, clusters 10 and 8 expressed genes implicated in cDC1‐driven anti‐tumour immunity (Figures 2D and 3B). Cluster 10 expressed high levels of *Cxcl9* and *Cxcl10* (Figure 3B), which encode for the eponymous chemokines that are important for the recruitment and spatial localisation of CD8^+^ T cells in tumours [8, 9]. Cluster 8 expressed higher levels of *Ccr7* and *Il12b* (Figure 3B), encoding for the chemokine receptor CCR7 and IL‐12p40 subunit common to IL‐12 and IL‐23. These cells were reminiscent of the cDC1 cellular state associated with tdLN migration [25], but also of CCR7^+^ cDC1s shown to sustain CD8^+^ T cells’ survival and proliferation within tumours [6, 7, 26, 27]. Of note, cluster 8 also expressed higher levels of *Il15ra* and *Cxcl16* (Figure 3D), which have been described as enriched in CCR7^+^ cDCs [6]. In addition to *Ccr7*, cluster 8 was also enriched in the “mregDC” gene expression signature [20] (Figure 3C). In contrast, cluster 10 showed enhanced expression of the CXCL9^+^ cDC1 signature described by Meiser et al. [8] (Figure 3C). Interestingly, these signatures were not only expressed in the respective clusters of cDC1s, but they were also enriched in their cDC2 counterparts on the activation bridges (Figure 3C), referred to as “cDC2 cluster 8‐like” and “cDC2 cluster 10‐like”, analogous to the cDC1 clusters (Figure 3E). When looking at the expression profiles of these activated cDC1s and cDC2s, we indeed observed a shared expression profile between cluster 10 of cDC1s and the cDC2 cluster 10‐like, including expression of *Cxcl9, Cxcl10* and *Irf1*, while cDC1 cluster 8 and cDC2 cluster 8‐like shared expression of a core “mregDC”‐like signature (Figure 3E).

Because clustering of scRNAseq datasets depends on resolution parameters and the relative heterogeneity of cells included in the dataset, we set out to validate the existence of the cDC1 clusters 8 and 10 by flow cytometry (Figure S3A). This was particularly important given the difficulty in distinguishing activated cDC1s from cDC2s at the mRNA level [20]. We used IL‐12p40 and CCR7 as flow cytometry markers for cDC1s from cluster 8, and CXCL9 for cDC1s from cluster 10. In line with the scRNAseq data, we observed two clear populations of cDC1s that expressed either IL‐12p40 or CXCL9 protein in a mutually exclusive manner (Figure 3F). IL‐12p40^+^ but not CXCL9^+^ cDC1s expressed high levels of CCR7 (Figure 3H; Figure S3B), again in line with the scRNAseq data. In addition, we analysed the expression of other activation‐associated markers (MHC class II (MHCII), CD80, CD86, CD40 and PD‐L1) on intratumoural cDC1s, and found that IL‐12p40^+^ cDC1s expressed higher levels of MHCII, CD80, CD86, PD‐L1 and CD40 than CXCL9^+^ cDC1s (Figure 3H; Figure S3B). Importantly, CXCL9^+^ and IL‐12p40^+^ cDC1s were not only found in MCA205 LA‐OVA tumours but could also be detected in other tumour models (MC38, YUMMM1.7, COX‐deficient Braf^V600E^ 5555 melanoma, B16‐F10) (Figure S3C), arguing that they are a common feature of murine transplantable tumours.

Interestingly, when we analysed the tumour cDC2 compartment by flow cytometry, CXCL9^+^ cDC2s were also detected while IL‐12p40^+^ cDC2s were very rare (Figure 3G). Nevertheless, when plotting CXCL9 against CCR7, two mutually exclusive populations of CXCL9^+^ and CCR7^+^cDC2s could be found (Figure 3G). This was reminiscent of the cDC1 dichotomy between CXCL9^+^ and IL‐12p40^+^ states and suggests that CCR7 (but not IL‐12p40) can be used to identify cluster 8‐like cDC2s (Figure 3E,G). Overall, the flow cytometry analysis validated the scRNAseq data and showed that activated cDC1s and cDC2s within tumours can exist in two states, differentially expressing CXCL9 or CCR7. For cDC1s but not cDC2s, the CCR7^+^ state corresponds to cells that also produce IL‐12p40. This is consistent with recent studies showing that activated cDC1s are heterogeneous and differentially marked by either CXCL9 or IL‐12p40 expression [8]. These two cDC1 activation states will be hereafter referred to as *Cxcl9* and *Il12b* cDC1s for simplicity.

### 2.3 *Cxcl9*‐ and *Il12b*‐Activated cDC1s Are Enriched in Different Regions of the Tumour

Next, we investigated whether the distinct cDC1 states were associated with specific regions within the tumour. We focused our analysis on day 9 to examine the organisation of cDC1 cellular states at a time point where they might be performing local functions within tumours, such as supporting CD8^+^ T cells expansion and tumour rejection (Figure 1A,B).

**FIGURE 4:**
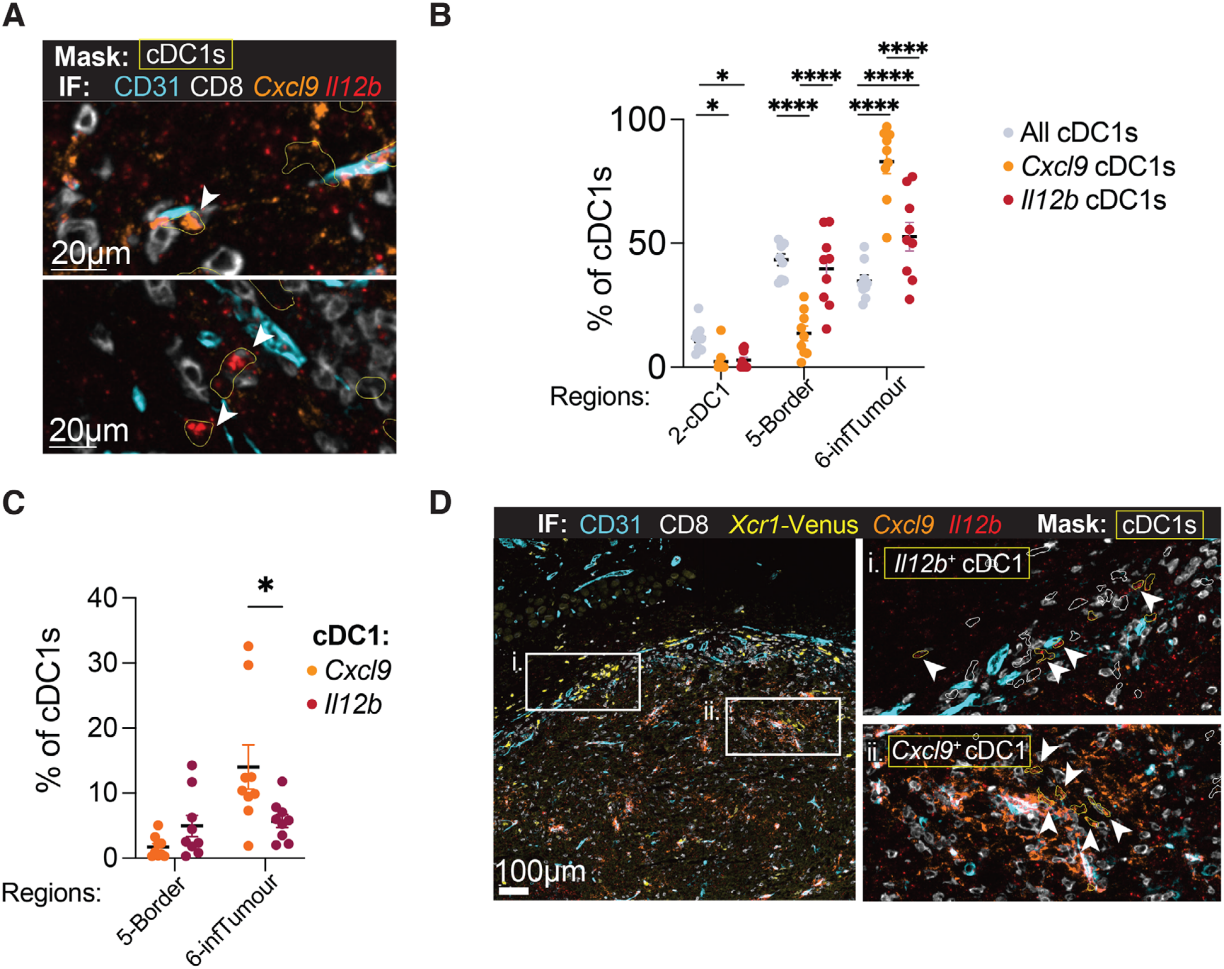
*Cxcl9* and *Il12b* activated cDC1s are enriched in different regions of the tumour. **(A)** Identification of *Cxcl9* (white arrow, upper panel) and *Il12b* (white arrows, lower panel) cDC1s on tumour sections using RNAscope and IF. Tumour sections were harvested on day 9 from *Xcr1‐*Venus mice. Shown are masks of cDC1s identified with Imaris (yellow contour), as well as CD8 (white), CD31 (turquoise), *Cxcl9* (orange) and *Il12b* (red) staining. **(B)** Distribution of total cDC1s (grey dots), *Cxcl9* cDC1s (orange dots) and *Il12b* cDC1s (red dots) across CytoMAP region 2‐cDC1 (stroma), 5‐Border and 6‐infTumour (parenchyma). *Cxcl9* and *Il12b* cDC1s were identified based on the volume of either RNAscope probe that overlapped with cDC1 surfaces. Distribution across CytoMAP regions 1 and 3 is not shown. Statistical analysis was performed using a two‐way ANOVA test, including all six CytoMAP regions and comparing the mean of all cDC1s, *Cxcl9* cDC1s and *Il12b* cDC1s. Each dot represents a tumour section from a different sample. *n* = 9 independent whole tumour sections, from two independent experiments. **(C)** Percentage of cDC1s from Region 5‐Border or Region 6‐infTumour expressing either *Cxcl9* (in orange) or *Il12b* (in red). Statistical analysis was performed using a two‐way ANOVA test and comparing the mean of *Cxcl9* cDC1s and *Il12b* cDC1s. *n* = 9 independent whole tumour sections, from two independent experiments. **(D)** Representative image of CD31, CD8, *Xcr1*‐Venus, *Cxcl9* and *Il12b* on tumour sections showing close‐ups of region enriched in *Il12b* cDC1s (upper right panel, masks outlined in yellow and highlighted with white arrows) and *Cxcl9* cDC1s (lower right panel, masks outlined in yellow and highlighted with white arrows). Masks outlined in white represent ungated cDC1s.

We first used MHCII staining to identify early cDC1s (Venus^+^ MCHII^low^), resting cDC1s (Venus^+^ MCHII^int^), and activated cDC1s (Venus^+^ MCHII^high^) (Figure S4A) because MHCII expression can distinguish pre‐cDCs from differentiated cDCs and strong MHCII staining was recently used to identify activated cDCs by microscopy [8]. As before, we generated CytoMAP regions based on the local abundance of cDCs, CD8^+^ T cells, blood vessels and tumour cells. Overall, the highest proportion of resting and activated cDC1s, as well as early cDC1s, was found in Region 5‐Border (Figure S4B). Next, we asked whether *Cxcl9* and *Il12b* cDC1s segregate to different regions of the tumour. To that end, we used RNAscope to detect *Il12b* and *Cxcl9* transcripts coupled with staining for protein markers for cDC1s (Venus^+^), blood vessels (CD31^+^) and CD8^+^ T cells (CD8^+^). *Cxcl9* and *Il12b* cDC1s were identified by quantifying *Cxcl9* and *Il12b* puncta within Venus^+^ cells (Figure 4A). Interestingly, using CytoMAP, we found different localisation patterns for *Il12b* and *Cxcl9* cDC1s. Proportionally, *Cxcl9* cDC1s localised to a greater extent in the parenchyma (CytoMAP Region 6‐infTumour), while the *Il12b* cDC1s were enriched in tumour border regions (CytoMAP Region 5‐Border) (Figure 4B,D). In addition, proportionally more cDC1s were *Cxcl9‐*positive than *Il12b‐*positive in Region 6‐infTumour, while the opposite trend was observed in Region 5‐Border (Figure 4C). When looking at the overall expression pattern of *Cxcl9* in tumours, irrespective of the cDC1s, we also observed an enrichment of *Cxcl9* transcripts in the parenchymal Region 6‐infTumour (Figure S4C), where *Cxcl9* expression sometimes overlapped with blood vessels (Figure S4D). This indicates that, in addition to cDC1s, vessel‐associated cells in the parenchyma can also express *Cxcl9*.

The localisation of activated cDC1s in immunogenic tumours was recently examined in detail [8]. That study identified a population of MHCII^high^ CCR7^neg^ cDC1s associated with CXCL9 expression at the stroma‐tumour interface [8], in apparent contrast with our findings of *Cxcl9* cDC1s enrichment in the tumour parenchyma. To assess the overlap between MHCII^high^ CCR7^neg^ cDC1s and CXCL9‐expressing cDC1s in our MCA205‐LA‐OVA model, we gated on MHCII^high^CCR7^neg^ cDC1s by flow cytometry and found that not all of them expressed CXCL9 (Figure S4E), as also observed by Meiser et al. using COX‐deficient Braf^V600E^ 5555 tumours. Thus, MHCII^high^ CCR7^neg^ cDC1 include CXCL9^+^ and CXCL9^–^ cells. Therefore, differences in how the CXCL9^+^ cDC1 state is identified on tumour sections, either through direct *Cxcl9* mRNA detection or inferred from the absence of CCR7 expression, may partly explain the discrepancies between our findings and those of Meiser et al. [8].

In sum, our results suggest that *Cxcl9* and *Il12b* cDC1s are enriched in different regions of the tumour, with *Cxcl9* cDC1s preferentially located in the parenchyma and the *Il12b* cDC1s in tumour border regions (Figure 4D).

### 2.4 Stem‐Like TCF1^+^ CD8^+^ T Cells Localise With *Il12b* cDC1s in Tumour‐Bordering Regions

We next focused on the localisation of CD8^+^ T cells in relation to *Cxcl9* and *Il12b* cDC1s. First, we took advantage of the expression of the model antigen OVA by the tumour cells to ask whether antigen‐specific CD8^+^ T cells associate with specific regions of the tumours. We transferred naïve CD45.1 OVA‐specific OT‐I CD8^+^ T cells into CD45.2 *Xcr1*‐Venus mice contemporaneously with tumour cell inoculation and harvested tumours seven days later to analyse OT‐I intratumoural distribution (Figure S5A). OT‐I and non‐OT‐I CD8^+^ T cells could be distinguished in tumour sections by staining for CD45.1 (Figure S5B,C). CytoMAP analyses of these tumours resulted in a slightly different region partition compared with previously. This time, CytoMAP did not generate a cDC1s Region 2‐cDC1 (stroma) and instead generated two regions that we annotated as “5A” and “5B” based on their similarity with the previously described Region 5‐Border (Figure S5D). We observed no difference in the distribution of OT‐I versus other CD8^+^ T cells across all CytoMAP regions (Figure S5E). The lack of OT‐I bias towards specific regions of the tumour compared with other CD8^+^ T cells indicates, therefore, that the overall distribution of CD8^+^ T cells in these tumours can be used as a proxy for those that are truly specific for tumour antigens.

**FIGURE 5:**
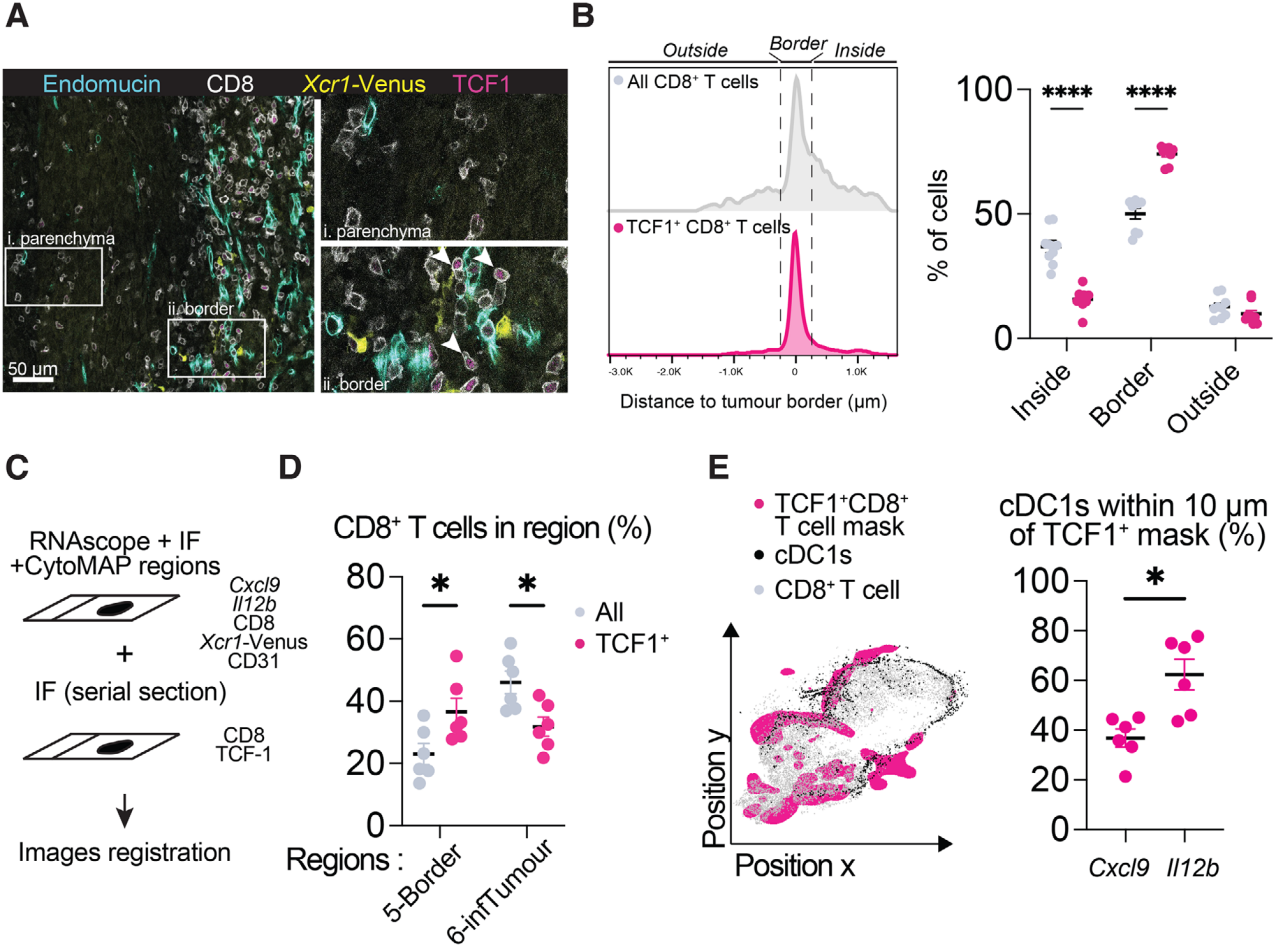
Stem‐like TCF1^+^ CD8^+^ T cells locate with *Il12b* cDC1s in tumour‐bordering regions. **(A)** Identification of stem‐like TCF1^+^ CD8^+^ T cells and TCF1^−^ CD8^+^ T cells by IF. Shown is a representative image of a tumour section stained for endomucin (turquoise), CD8 (white), *Xcr1‐*Venus (yellow) and TCF1 (magenta) (left panel) and close‐ups of parenchyma regions (upper right panel) and tumour border region enriched in blood vessels and cDC1s (lower right panel). White arrows indicate TCF1^+^ CD8^+^ T cells at the tumour border. CD8^+^ T cells were segmented using cellpose, and TCF1 staining outside of segmented CD8^+^ T cells was removed in Fiji for clarity. **(B)** Left: Representative histograms of distances of all CD8^+^ T cells (in grey) and TCF1^+^ CD8^+^ T cells (in magenta) to the tumour border for one tumour section. Right: percentage of cells “inside”, “at the border” and “outside” of the tumour based on the dotted‐line gates shown on the histogram. Each dot represents a different tumour sample. CD8^+^ T cells and TCF1^+^ CD8^+^ T cells were segmented using Imaris, and the shortest distance of each cell to the tumour border was calculated using CytoMAP. Statistical test: two‐way ANOVA test, comparing the mean of total CD8^+^ T cells (in grey) and TCF1^+^ CD8^+^ T cells (in magenta). *n* = 9 independent whole tumour sections, from two independent experiments. **(C)** Schematic of images registration of serial sections that were stained for *Cxcl9, Il12b*, CD8, *Xcr1‐*Venus and CD31 (from Figure 3) and CD8 and TCF‐1 (from Figure 4B). Registration was done using the Fiji bUnwarpJ plugin. Only six sections out of nine were kept for following analyses based on the performance of the registration. **(D)** Percent of total CD8^+^ T cells (in grey) and TCF1^+^ CD8^+^ T cells (in magenta) classified as CytoMAP Regions 5‐Border and 6‐infTumour following registration. Statistical analysis: two‐way ANOVA test and comparing the mean of total CD8^+^ T cells (in grey) and TCF1^+^ CD8^+^ T cells (in magenta). *n* = 6 independent whole tumour sections, from two independent experiments. **(E)** Association of *Cxcl9* and *Il12b* cDC1s with TCF1^+^CD8^+^ regions. Registered TCF1^+^CD8^+^ masks were generated based on the density of TCF1^+^CD8^+^ cells. Distances between cDC1s and the mask were calculated. cDC1s that were inside or within 10 µm distance from the registered masks were considered to be associated with TCF1^+^CD8^+^ regions. Shown on the left is a representative plot from one tumour section of the TCF1^+^CD8^+^ mask (in magenta) overlaid with cDC1s (black dots) and total CD8^+^ T cells (in grey). Shown on the right are the percentage of *Cxcl9* and *Il12b* cDC1s within 10 µm of the mask. *n* = 6 independent whole tumour sections, from two independent experiments. Statistical test: two‐tailed paired *t*‐test.

Tumour‐infiltrating CD8^+^ T cells can be found in different states, including effector cytotoxic CD8^+^ T cells that express granzyme B (*Gzmb*) and perforin (*Prf1*), and stem‐like TCF1^+^ CD8^+^ T cells that lack effector functions but can replenish the cytotoxic CD8^+^ T cell pool [33]. We stained tumour sections for TCF1 and CD8 and observed that stem‐like TCF1^+^CD8^+^ T cells were enriched at the tumour borders but reduced in the parenchyma, compared with total CD8^+^ T cells (Figure 5A,B). Similarly, TCF1^+^ CD4^+^ T cells were enriched at the tumour border compared with total CD4^+^ T cells (Figure S5F).

To assess if the location of stem‐like and effector‐like CD8^+^ T cells correlated with *Il12b* and *Cxcl9* cDC1s, we registered serial sections from the *Il12b* and *Cxcl9* RNAscope experiment (Figure 3) with ones stained for TCF1 (Figure 5B,C). We first inferred TCF1^+^ and TCF1^−^ CD8^+^ T cell association with the 6 CytoMAP regions from the RNAscope by calculating the shortest distances between the registered TCF1^+^ and TCF1^−^ CD8^+^ T cells and the CytoMAP regions. We found that TCF1^+^ CD8^+^ T cells were increased within the tumour border Region 5‐Border compared with the total CD8^+^ T cells and were decreased in parenchyma Region 6‐infTumour, in line with the quantifications above (Figure 5D). This was also the case when looking at the percentage of TCF1^+^ CD8^+^ T cells within 10 µm of each region (Figure S5G). To identify the association of *Cxcl9* and *Il12b* cDC1s with TCF1^+^ CD8^+^ T cells, we generated masks representing areas where TCF1^+^ CD8^+^ T cells densely accumulate within tumours (Figure 5E; Figure S5H). Regional masks rather than precise T cell location were used to assess their association with cDC1s because imperfect section registration does not provide precise cell‐to‐cell correlation. We found a higher percentage of *Il12b* cDC1s within 10 µm of the CD8^+^ TCF1^+^ mask compared with the *Cxcl9* cDC1s, suggesting that *Il12b* cDC1s are enriched in the vicinity of TCF1^+^ CD8^+^ T cells (Figure 5E).

Overall, these results suggest that, similarly to cDC1s, CD8^+^ T cell states are spatially organised in tumours, with TCF1^+^ T cells enriched at tumour borders. In line with this, we find that *Il12b* cDC1s associate more closely with TCF1^+^ CD8^+^ T cells compared with the *Cxcl9* cDC1s located in the parenchyma.

### 2.5 *CCR7* ^+^ cDC1s Associate With TCF1^+^ Regions in Human Tumours

Analysis of cDC1 heterogeneity in samples from cancer patients also reported the existence of *CXCL9* ^+^ and *CCR7* ^+^ cDC1s in human tumours [8]. To assess whether the patterns of cDC1 and CD8^+^ T cell states distributions that we observed in murine tumours are preserved in human tumours, we explored a published MERFISH dataset from hepatocellular carcinoma (HCC) patients [12] that included markers of cDC1s (such as *XCR1, CLEC9A*), CD8 and CD4 T cells (*CD3E, CD4, CD8*), cDC1 states (*CXCL9, IL12B, CCR7*), and T cell states (*TCF1*).

**FIGURE 6:**
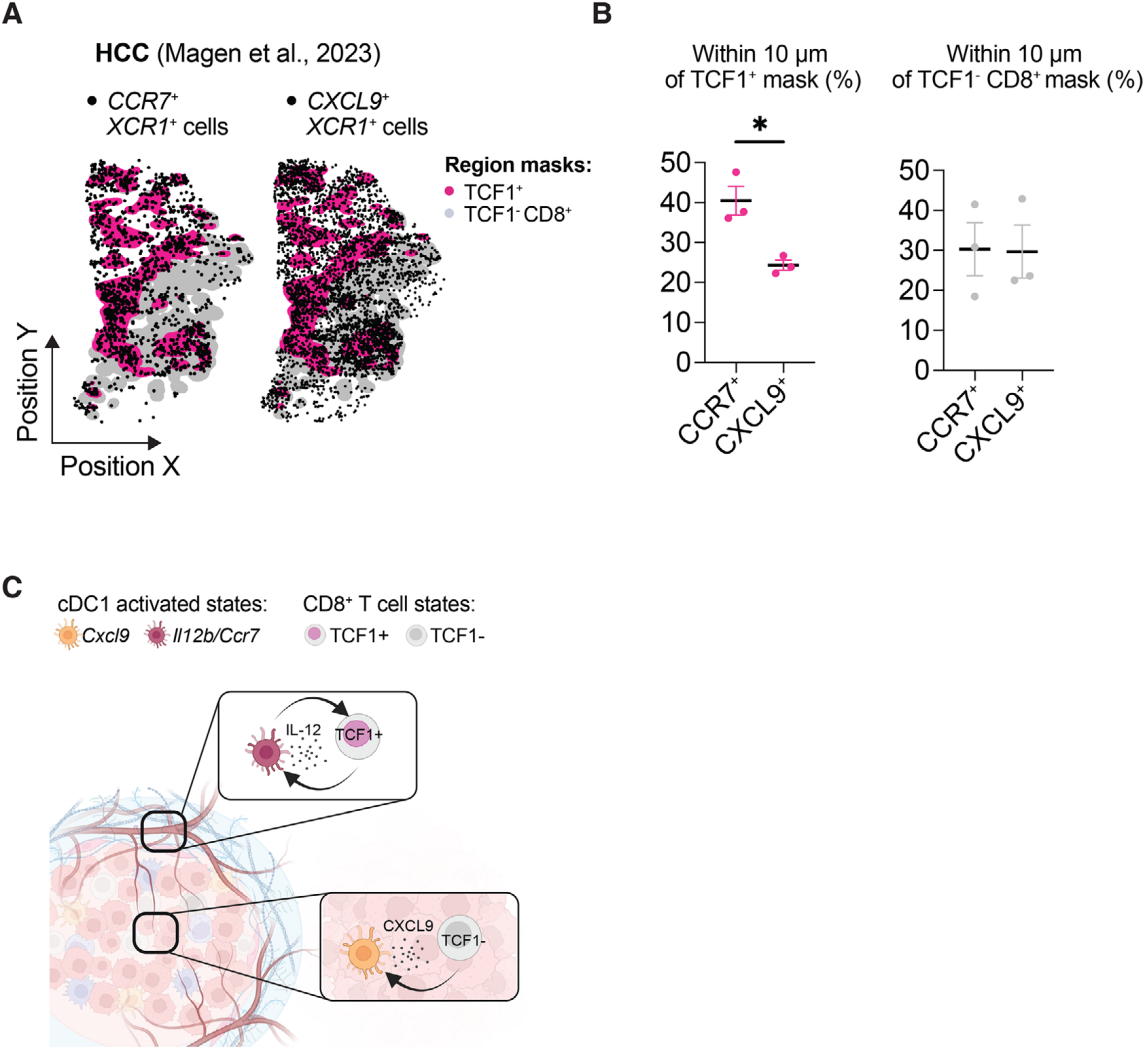
*CCR7* ^+^ cDC1s associate with TCF1^+^ regions in human tumours. **(A)** Association of *CCR7^+^ XCR1^+^* and *CXCL9^+^ XCR1^+^* cDC1s with *TCF1* ^+^ and *TCF1* ^−^ *CD8* ^+^ regions in human HCC tumour sections. *CCR7^+^ XCR1^+^* and *CXCL9^+^ XCR1^+^* cDC1s, as well as *TCF1* ^+^ and *TCF1* ^−^ *CD8* ^+^ cells, were identified in the MERFISH dataset published by [12]. Three samples were included based on whether a population of *CCR7^+^ XCR1^+^* and *CXCL9^+^ XCR1^+^* cDC1s could be identified in the sample. Masks were generated based on the density of *TCF1* ^+^ cells or *TCF1* ^−^ *CD8* ^+^ cells, and the distances between cDC1s and the mask were calculated. Shown are representative plots from one tumour section with TCF1^+^ mask (in magenta) and TCF1^−^ CD8^+^ mask (in grey) overlaid with *CCR7^+^ XCR1^+^* cDC1s (on the left) and *CXCL9^+^ XCR1^+^* cDC1s (on the right). **(B)** Percentage of *CCR7^+^ XCR1^+^* cDC1s (on the left) and *CXCL9^+^ XCR1^+^* cDC1s within 10 µm of the TCF1^+^ mask (left) and TCF1^−^ CD8^+^ mask (right). Statistical test: two‐tailed paired *t*‐test. **(C)** Graphical summary of the study. Intratumoural cDC1s can be found in two distinct activation states associated with either *Cxcl9* or *Il12b* expression and are located in different regions of the tumour. *Cxcl9* cDC1s are located in the tumour parenchyma during tumour rejection, whereas *Il12b* cDC1s are enriched at the tumour borders. These locations correlate with those of CD8^+^ T cell states within tumours, with stem‐like TCF1^+^ CD8^+^ T cells enriched at tumour borders with *Il12b* cDC1s, while cytotoxic TCF1^−^ CD8^+^ infiltrate the tumour parenchyma. The figure was generated with BioRender.

The HCC dataset comprised 10 tumour section samples from 6 patients treated with anti‐PD1 therapy and 1 untreated patient. From these, we selected three samples in which distinct clusters of *CXCL9^+^* and *CCR7^+^* could be identified. We used *CCR7* expression as a proxy to infer the *IL12B* cDC1 state because of the low abundance of *IL12B* transcripts on these sections (not shown) and given that *Ccr7* and *Il12b* expression by tumour cDCs is perfectly correlated (Figure 3H; Figure S3B), as previously described [20, 24]. We identified clusters of TCF1^+^ T cells (including both CD4^+^ and CD8^+^ T cells) and of TCF1^−^ CD8^+^ T cells and projected them on the tumour space. We generated masks of TCF1^+^‐enriched regions or of TCF1^−^ CD8^+^ T cell regions, and overlayed the *CCR7* ^+^ and the *CXCL9* ^+^ cDC1s on these masks (Figure 6A). Similar to what we observed in murine tumours, we identified a higher proportion of *CCR7^+^* cDC1s located within 10 µm of the TCF1^+^ mask compared with *CXCL9^+^* cDC1s (Figure 6B). These results highlight that the spatial organisation of human cDC1 states with CD8^+^ T cells resembles that in murine tumours. Thus, potential interactions between *Il12b* cDC1s and stem‐like TCF1^+^ CD8^+^ T cells, as well as *Cxcl9* cDC1s and effector TCF1^−^ CD8^+^ T cells, might be important for anti‐tumour immunity across species (Figure 6C).

## 3 Discussion

While it is well established that cDC1s perform key roles in anti‐tumour immunity, it is still unclear how cDC1s are organised in space and time to perform these functions. Here, we perform a quantitative analysis of the distribution of cDC1s in immunogenic tumours using CytoMAP as an unbiased and comprehensive framework to describe cell distributions. This strategy complements other studies that focused on identifying cellular neighbourhoods predicted a priori to be important for anti‐tumour immunity [8]. We found that a large proportion of cDC1s are dispersed in the stroma during early initial tumour development, but that their distribution changes over time. At a late time point, during tumour rejection and intratumoural CD8^+^ T cell expansion, cDC1s largely accumulate at the border of the tumours, a region that is also characterised by a high density of blood vessels and CD8^+^ T cells. These distinct patterns might reflect the different roles that cDC1s play at these two time points, related primarily to CD8^+^ T cell priming in tdLNs versus CD8^+^ T cell support within tumours, respectively.

The large number of cDC1s found at the tumour border during tumour rejection is intriguing, considering that a central role for cDC1s in anti‐tumour immunity is thought to be to take up antigens from dying tumour cells, presumably from regions of necrosis in the tumour parenchyma. However, in addition to their role in T cell priming in tdLNs, cDC1s have also been suggested to perform local functions in the tumour, for example, by recruiting and interacting with intratumoural CD8^+^ T cells. Here, we generated an unprecedentedly large scRNAseq dataset of the cDC lineage in tumours to characterise the different states of tumour cDC1s. We identified clusters of early cDC1s/pre‐cDC1s, resting cDC1s, and activated cDC1s, and, in particular, two distinct clusters of activated cDC1s associated with *Cxcl9* and *Il12b/Ccr7* expression, respectively. These cellular states are reminiscent of cDC1‐mediated functions in anti‐tumour immunity described in other studies. CXCL9 expression by cDC1s has been suggested to be important for CD8^+^ T cells recruitment to tumours, including in regions rich in cDC1s at the tumour borders [8, 9]. CXCL9 was also shown to be induced in cDC1s upon TIM3 blockade in mice, which contributed to the therapeutic effect [30]. IL‐12p40 and CCR7 have also been shown to be important for cDC1‐mediated tumour rejection. CCR7 has long been established to be necessary for migration of cDC1s to LNs [25] but has more recently also been suggested to be expressed in activated cDC1s that are retained in the tumours [7], potentially by CCL19‐expressing fibroblasts [26]. CCR7^+^ IL12‐p40^+^ cDCs have been shown to associate with CD8^+^ T cells in perivascular regions of the tumour where they trans‐present IL‐15 to CD8^+^ T cells [6] and are suggested to be important for the efficacy of immune‐checkpoint blockade [27].

Activated cDCs in tumours have largely been associated with a core gene expression signature that includes *Ccr7* and *Il12b*, amongst other markers [6, 7, 20, 24]. However, our scRNAseq and flow cytometry analyses suggest that a distinct population of activated cDC1s is associated, instead, with *Cxcl9/*CXCL9 expression. This is in line with recent work from Meiser et al. [8] that demonstrated the existence of two distinct activated cDC1 populations associated with CXCL9 or IL‐12p40/CCR7 in tumours. Interestingly, a similar dichotomy was also recently shown in the spleen under steady‐state conditions [15], suggesting that these two activation states can exist in other contexts.

We used RNAscope to directly assess the localisation of the activated states identified by scRNAseq. We found that *Cxcl9* and *Il12b* cDC1s were enriched in different regions of the tumour: *Cxcl9* cDC1s were overrepresented in the parenchyma of the tumour, while *Il12b* cDC1s were located preferentially at the border of the tumour. We correlated these findings with the locations of intratumoural CD8^+^ T cell states, using TCF1 to distinguish TCF1^+^ stem‐like from effector T cells. We observed similar dichotomous localisation patterns as for the cDC1 states, with stem‐like CD8^+^ T cells located predominantly at the borders of the tumour. In human tumours, we also observed an enrichment of *CCR7* cDC1s but not *CXCL9* cDC1s within *TCF1* regions, which suggests that discrete spatial co‐localisation is preserved across species. This is in line with recent studies reporting spatial clustering of *CCR7* and *TCF1* cells and more distal *CXCL9* expression within human tumours [26], and with work from Di Pilato et al. [6] that reported the close proximity of CCR7/IL‐12 cDCs to perivascular regions of the tumours. TCF1^+^ CD8^+^ T cells have also been found in close proximity to CD4^+^ T cells, including regulatory T cells, as well as B cells [12, 26]. Therefore, TCF1^+^ CD8^+^ T cells in tumours seem to occupy distinct niches that include multiple cell types beyond CCR7^+^ cDC1s, all of which might impact anti‐tumour immunity.

In apparent contrast to our finding that *Cxcl9* cDC1s are enriched within the tumour parenchyma, the study from Meiser et al. [8] analysed a population of MHCII^high^ CCR7^neg^ cDC1s associated with CXCL9 expression and suggested that they are found at the stroma‐tumour interface. In our analysis (Figure S4E), a large fraction of MHCII^high^ CCR7^neg^ cDC1s did not stain for CXCL9, suggesting that differences in how cDC1 states are identified (either through direct measurement of *Cxcl9* or by absence of CCR7) might account for these discrepancies. Additionally, the use of different transplantable tumour models in the studies could contribute to these differences. Direct comparison between these two studies is further limited by differences in the time points analysed, with the study by Meiser et al. [8] focusing on an earlier time point (day 6) than our analysis, which was performed on day 9. Future studies comparing tumour models and time points will help clarify the overlap between MHCII^+^CCR7^neg^ and *Cxcl9* cDC1s, as well as their localisation within tumours.

Our work focused on the spatial association between activated cDC1s and TCF1⁺ CD8⁺ T cells, but CD8⁺ T cells in tumours span a spectrum of states, including stem‐like, effector, and exhausted. Techniques such as multiplex imaging and spatial transcriptomics can clarify how cDC1s interact with these diverse CD8⁺ T cell types. Preliminary data using an extended panel of phenotypic markers suggest an enrichment of effector CD8⁺ T cells within the tumour parenchyma (data not shown). As such, CXCL9⁺ cDC1s may be key to recruiting effector TCF1^−^ CD8^+^ T cells into the tumour parenchyma. However, other cell types, including cDC2s and endothelial‐associated cells, also express *Cxcl9* (Figure 3G; Figure S4D). In addition, a subset of tumour‐associated macrophages expressing CD206 has also been reported to express CXCL9 and to play a role in supporting the presence of cDC1s, CD8⁺ T cells, and NK cells [34]. These observations raise the question of functional redundancy. Whether cDC1s perform distinct, non‐redundant roles, in particular those associated with *Il12b* or *Cxcl9* activate states, remains to be determined. Furthermore, extending analysis to include cDC2s and pre‐cDCs will improve understanding of how the dendritic cell lineage, beyond cDC1s, participates in anti‐tumour immunity.

It also remains unclear whether *Il12b*‐ and *Cxcl9*‐expressing states reflect stages in a shared trajectory. We interpret them here as coexisting discrete activation programmes, but further studies are needed to identify the upstream cues that shape them and assess plasticity. Prior work suggests uptake of apoptotic cells may drive CCR7‐associated cDC1 activation programmes [15, 35], while IRF1 and NF‐κB pathways have been more generally linked to cDC1 activation in tumours [36]. Defining the molecular circuits governing these states, especially those regulating IL‐12p40 and CXCL9, will be essential to understanding cDC1 contributions to anti‐tumour immunity. Furthermore, our analysis offers a static view of cDC1 localisation. It remains unknown whether cDC1s in the tumour border migrate into the parenchyma, how long they persist in tumours, or whether they exit to tdLNs. This is especially relevant for CCR7⁺ cDCs, for which the mechanisms governing LN migration versus local residency are unclear.

In sum, various studies have reported a role for IL‐12 and CXCL9 in anti‐tumour immunity. Our data suggest that these functions are performed by two distinct activated states of intratumoural cDC1, which are spatially segregated and associated with specific CD8^+^ T cell cellular states. Based on their localisation, *Il12b* cDC1s might be important to sustain TCF1^+^ T cells at the tumour border, potentially through local expression of IL‐12, IL‐15 trans‐presentation and/or antigen (cross‐) presentation. On the other hand, *Cxcl9* cDC1s might be important to recruit effector CD8^+^ T cells within the parenchyma, where they can perform their cytotoxic functions. Future studies targeting these specific axes in cDC1s will be important to test these hypotheses.

### 3.1 Data Limitations and Perspectives

Our study describes the localisation and gene expression signatures of cDC1s in immunogenic tumours, but their functional relevance remains to be directly tested. Existing models for cDC1 depletion do not allow selective targeting of specific activated states. Future tools, for example, an *Xcr1‐*Cre *Cxcl9‐*LSL‐DTR mouse enabling ablation of CXCL9⁺ cDC1s, could be used to address this.

A major challenge remains in distinguishing cDC1 functions within tumours from functions leading to T cell priming in tdLNs. As cDC1 depletion affects both compartments, strategies such as FTY720 treatment, as recently used [8], are needed to block egress of primed T cells from tdLNs and isolate the intratumoural compartment. Combining this approach with selective genetic models targeting activated cDC1 subsets or effector functions could clarify how cDC1s shape CD8⁺ T cell responses within the TME.

A limitation of our study is the focus on a single tumour model. To assess the generalizability of our findings, broader validation across tumours with varying immunogenicity will be critical, including both transplantable models and genetically engineered mouse models. Furthermore, extending these investigations to a wider range of human tumours is also essential to determine whether the spatial patterns observed here are conserved in human tumours. Notably, murine transplantable tumours often exhibit more structured architecture than human tumours, which are typically more heterogeneous and evolutionarily complex. Therefore, larger patient cohorts and systematic spatial analyses will be necessary to evaluate whether localisation patterns identified in mice are preserved in humans and correlate with clinical outcomes.

## 4 Materials and Methods

### 4.1 Experimental Animals

The following mouse strains were bred at The Francis Crick Institute in specific pathogen‐free conditions: C57BL/6J, *sGsn^−/−^* [3], *sGsn^−/−^ Clec9a^−/−^* [3], CD45.1 OT‐I, *Xcr1‐*Venus [31]. Transgenic mice were backcrossed to C57BL/6J. Male and female mice between 6 and 20 weeks of age were used in this work. Animal experiments were performed in accordance with national and institutional guidelines for animal care and were approved by the Francis Crick Institute Biological Resources Facility Strategic Oversight Committee (incorporating the Animal Welfare and Ethical Review Body) and by the Home Office, UK.

### 4.2 Transplantable Tumours

For tumour injection, MCA205 LA‐OVA‐mCherry [3], 5555 COX‐KO [37], YUMM1.7 (from Erik Sahai, ATCC CRL‐3362), B16‐F10 (from Erik Sahai) and MC38 (ENH204‐FP, The Francis Crick Institute) cells were thawed 1 week before injection and cultured in RPMI 1640 supplemented with 2 mM glutamine, 100 units/mL penicillin, 100 µg/mL streptomycin, nonessential amino acids, 10 mM HEPES, 50 µM 2‐mercaptoethanol (all from Gibco) and 10% heat‐inactivated fetal calf serum (FCS) (R10+ medium) in humidified 37°C in a 5% CO_2_ incubator. On the day of injection, cells were collected and injected subcutaneously in 100 µL in one or both flanks. Tumour growth was monitored every 2 to 3 days, and the longest tumour diameter (*l*) and perpendicular width (*w*) were measured using digital Vernier callipers. Tumour volumes were calculated using the formula: length × width^2^/2 and expressed as mm^3^ [38].

### 4.3 Administration of Brefeldin A in Vivo

To analyse cytokine expression in cDCs, mice were injected i.v. or i.p. with Brefeldin A (Sigma, B6542) at 1 mg/mL in 200 µl. Brefeldin A was first reconstituted in DMSO at 20 mg/mL and diluted 20 times in pre‐warmed PBS right before the injection. Mice were culled 6 h later, and tissues were processed in the presence of Monensin solution (Biolegend cat# 420701) until fixation to prevent the secretion of cytokines during tissue processing.

### 4.4 Adoptive Transfer of Naïve CD8^+^ OT‐I Cells

Spleens were harvested from CD45.1 OT‐I mice and mashed on a 70 µm strainer. Naïve CD8^+^ T cells were enriched using the EasySep Mouse CD8^+^ T cell isolation kit (Stem Cell Technologies, cat# 19858). In brief, splenocytes were incubated with rat serum and with the isolation cocktail mix for 10 min. Rapidspheres particles were added to the samples and incubated for 5 min. The sample was placed into the EasySep magnet, and the enriched cell suspension containing CD8^+^ T cells was collected by pouring the unbound fraction of the magnet into a collection tube. A thousand naïve CD8^+^ T cells were injected i.v. in 200 µL of PBS per mouse on the day of tumour injection.

### 4.5 Cell Isolation

LNs and tumours were cut into small pieces and digested for 20 min (LNs) or 40 min (tumours) in collagenase VIII (1 mg mL^−1^, Sigma‐Aldrich) and DNase I (0.4 mg mL^−1^, Roche). Digested tissues were transferred to a 70 µm cell strainer on 50 mL Falcon tubes, and tissues were mashed on the filter using a syringe plunger. Strainers were washed with FACS buffer (PBS supplemented with 3% FCS and 5 mM EDTA). For tumours, leukocytes were enriched using Percoll to improve the quality of the staining. Samples were resuspended in 6 mL of 40% Percoll at RT and added on top of 6 mL of 80% Percoll in a 15 mL Falcon, and centrifuged for 23 min at 325×*g* at room temperature (RT), with an ascending and descending rate of 5. Leukocytes were collected after centrifugation at the interface of the two layers.

### 4.6 Flow Cytometry

Flow cytometry experiments were performed according to the “Guidelines for the use of flow cytometry and cell sorting in immunological studies” [39]. Cells were plated in v‐bottom 96‐well plate and spun down at 1400 RPM for 4 min. For CCR7 staining, cells were resuspended in 100 µl R10+ medium with anti‐CCR7 and purified anti‐CD16/32, and incubated for 20 min at 37°C in a 5% CO_2_ incubator. After washing, cells were resuspended in 100 µL of PBS with fixable live/dead blue or green (Invitrogen, cat# L23105 and # L23101) and left for 5 min at RT in the dark. Cells were spun down and incubated with purified anti‐CD16/32 (if not already done with the CCR7 staining) in 50 µL. An antibody mix was prepared at 2X, and 50 µL was added on top of the CD16/32 blocking step. The antibodies used for flow cytometry are listed in Table S2. Cells were resuspended and incubated for 30–40 min at 4°C in the dark. Samples were washed and fixed for 5 min (FIX&PERM Solution A, Nordic MUbio) and resuspended in FACS buffer.

For cytokines staining, cells were permeabilised using the CytoPerm buffer (BD Cytofix/CytopermTM Fixation/Permeabilisation Kit, cat# 554714) after the fixation step for 20 min, spun down, and antibodies were added at 1:75 in 50 µL of CytoPerm buffer for 45 min. Cells were washed two times with CytoPerm buffer and resuspended in FACS buffer for acquisition. For analysis, samples were acquired using a BD FACSymphony A5 (BD Bioscience) or an ID7000 (Sony Biotechnology). Analyses were performed using FlowJo software (BD Bioscience).

### 4.7 Generation of the scRNAseq Dataset

A cohort of WT (25 males and 12 females), *sGsn^−/−^* (15 males and 16 females) and *sGsn^−/−^ Clec9a^−/−^* (17 females and 10 males) mice was prepared. Mice were co‐housed for more than 3 weeks before being injected with MCA205 LA‐OVA tumours on both flanks, and tumours were harvested on day 5. Tumours were pooled per genotype for the processing and sorting. For the processing and digestion, five tumours were pooled together. Tumours were cut into pieces in GentleMACS C tubes (Miltenyi, cat# 130‐093‐237), and digested in 6 mL of the digestion mix containing Collagenase IV (200 U/mL, Worthington) and DNase I (0.2 mg/mL, Roche) in RPMI. Before incubation, GentleMACS tubes were put on a GentleMACS dissociator using the m_impTumour_02 program. Tumours were then digested on a shaker at 37°C for 40 min, following which the tubes were put again on the GentleMACS dissociator using the m_impTumour_03 program. Tumours were filtered as described above, and leukocytes were enriched using the Percoll strategy as described above. Samples were pooled per genotype for staining. Cells from each genotype were sorted in parallel on three BD FACSAria Fusion cell sorters (BD Bioscience). Pre‐cDCs (CD135^+^ CD43^+^), cDC1s (XCR1^+^) and cDC2s (Sirpa^+^) were identified by first gating on Live (LiveDead Green^−^) CD45^+^Lineage^−^ (CD19^−^, TER‐119^−^, Ly6G^−^, B220^−^, Cd3e^−^, NK1.1^−^, Siglec‐F^−^ and Ly6D^−^) CD11c^+^ MHCII^+^ CD88^−^ and in Eppendorf tubes prefilled with RPMI and 10% of FCS. A maximum of 20,000–40,000 cDC2s were sorted per genotype. For each genotype, between 8000–13,000 cDC1s, 11,000–14,000 pre‐cDCs, and 10,000–20,000 cDC2s were pooled, centrifuged, and resuspended in 50 µL. Viability was assessed, and samples were loaded onto a 10X Genomics Chromium. Libraries were generated and sequenced by Illumina sequencing.

### 4.8 scRNAseq Analysis

Raw reads were processed using the Cell Ranger pipeline and aligned to the mm10 reference transcriptome using the count function of cellranger. Barcodes, features and matrix files were generated for each genotype and explored using the Seurat package (v3) [42] in R (v4.0.0.). Seurat objects were generated for each genotype using cells for which at least 200 “features” (or genes) were detected, and genes that were expressed in at least three cells. For each genotype, the data were normalised, and the top 2000 most variable genes were identified using the “vst” selection method embedded in Seurat. The three Seurat objects were integrated to reduce putative batch effects using the integration functions of Seurat. Principal component analysis (PCA) of the integrated scaled data was run, and a UMAP was generated based on the first 50 PCs. Clusters were generated using the FindNeighbors on the PCA reduction of the data and the FindClusters function of Seurat. Differentially expressed genes (DEGs) were generated for each cluster using a Wilcoxon rank‐sum test. Clusters were annotated on the basis of canonical genes expressed on different immune populations, such as *Cd7* for pre‐cDCs, *Xcr1* for cDC1s, *Itgam* for cDC2s and *Ccr7* migratory cDCs, respectively. *Siglech*, *Cd8*, *Mki67* and *Ncr1* expression were used to identify pDCs, CD8^+^ T cells, proliferating cells and NK cells, respectively. For the initial exploration of the data, obvious clusters of dying cells that were enriched in mitochondrial transcripts were identified and excluded from further analyses. For the subsequent analyses, the means and the standard deviations of the percentages of mitochondrial transcripts were calculated, and cells for which this value was higher than the mean plus two times the standard deviation were removed.

Cells from the cDC lineage were re‐analysed separately by extracting their cell IDs and by generating new Seurat objects, and using the same strategy as described above. DEGs of the cDC1 clusters were generated using the FindAllMarkers function of Seurat, and the top5 or top10 DEGs for each cluster were generated based on the average log2FC.

Tumour cDC1s and splenic cDC1s [15] were integrated using Seurat, as described above. Metadata from splenic cDC1s were overlayed on the integrated UMAP space to identify regions of pre‐cDC1s, “immature” cDC1s and “mature” cDC1s.

### 4.9 Tissue Preparation for Microscopy

Tumours were harvested and transferred immediately in 4% paraformaldehyde (PFA) by diluting four times 16% PFA (Generon, Electron Microscopy Sciences) in PBS, and left overnight at 4°C.

For fixed‐frozen sections, tissues were next transferred to 30% sucrose and left for at least 24 h at 4°C. Tumours were dried gently by using tissues to absorb liquid and transferred to a cryomold cassette of 15 mm × 15 mm × 15 mm (Sakura), prefilled with OCT compound (VWR). The cassettes were transferred on a metal block that had been put on dry ice 30 min before to cool down. Tumours in OCT were preserved at −80°C.

Tumour sections were prepared using a Leica 3050 cryostat to generate 10–30 µm frozen sections on SuperFrost Plus adhesion slides (Thermo Fisher). Sections were left to dry for 20 min and transferred to −80°C until staining.

For FFPE sections, tissues were transferred in 70% ethanol after fixation and processed to paraffin on the Sakura VIP6 AI tissue processor before embedding in paraffin and storing at room temperature. 3–8 µm sections were cut on a rotary microtome onto Plus+Frost positively charged slides (Solmedia Ltd.). Sections were drained, baked for 60 min on a 60°C hotplate and then stored at 4°C until staining.

### 4.10 Immunofluorescence Staining

For fixed frozen sections, slides were taken out of the −80°C, dried for 30 min and rehydrated using PBS. Sections were blocked for 1 h at room temperature (RT) using blocking buffer containing 0.3% Triton and 3% BSA in PBS. Primary antibody staining mix was prepared in blocking buffer and applied to the tissue section by drawing boundaries around the tumour using a hydrophobic PAP pen (Vector Laboratories) or using Parafilm to cover and spread the staining mix homogeneously on the slide. The antibodies used for staining are listed in Table S2. The primary staining was performed overnight in a humidified chamber at 4°C. Slides were washed three times for 10 min by adding fresh PBS to the tumour sections. When needed, sections were blocked with goat, rat or rabbit serum at 1:50 before the secondary staining step. Secondary staining was performed for 1 h at RT. Slides were washed three times with PBS. Optionally, tumours were stained for 5 min with Hoechst (Thermo Fisher Scientific, 1:500 in PBS) and washed again three times. Sections were mounted using ProLongTM Diamond Antifade Mountant (Thermo Fisher Scientific).

FFPE sections were stained on the Leica Bond Rx automated stainer using sequential application of primary antibody, HRP‐secondary and Opal TSA fluorophore (Akoya Biosciences), followed by antigen retrieval heat stripping of the primary–secondary complex and application of the next antibody (Table S2).

### 4.11 Combined RNAscope and Immunofluorescence Staining

8–10 µm FFPE sections from MCA205 LA‐OVA‐mCherry tumours from *Xcr1‐* Venus mice were defrosted, fixed for 10 min in 10% NBF, washed with PBS and baked for 30 min at 60°C. Sections were then stained on the Leica Bond Rx automated stainer using RNAscope LS Multiplex Fluorescent assay (322800 ACD Bio‐Techne), applying a 5 min target retrieval at 88 ° C followed by a 10 min protease treatment at room temperature. For 5 µm FFPE sections, a 15 min target retrieval at 95°C followed by 15 min protease treatment at 37°C was used.

The following probes were used: Mm‐Cxcl9 (489348 Bio‐Techne) and Mm‐Il12B‐C2 (319558‐C2 Bio‐Techne). Following RNAscope, sections were stained with anti‐GFP to amplify the Venus signal using the antibody at 1:300 (Ab6673 Abcam) and ImmPRESSÒ HRP Horse Anti‐Goat IgG Polymer (MP‐7405‐50 2BScientific), and with anti‐CD8 (Abcam ab217344) and anti‐CD31(R&D AF3628) to stain additionally for blood vasculature and CD8^+^ T cells. Opal TSA fluorophores (Akoya Biosciences) were used (Table S2).

### 4.12 Image Acquisition and Stitching

Sections were imaged using a 10X air or a 40X oil immersion objective on a LSM880 inverted confocal microscope (Zeiss), or using the Phenoimager HT (Akoya Biosciences). Spectral unmixing was performed when necessary to remove bleedthrough between Opal fluorophores using the Inform software from Akoya Biosciences, using a synthetic Opal spectral library and a representative autofluorescent image of the tumour tissue. For confocal microscopy, tile scans and z‐stacks were acquired with an optimal step size, a pinhole of 1 AU, and 0% overlap between tiles. Images were acquired using 1024 × 1024 voxel density and a bit depth of 16 Bit. Images were stitched in Zen Blue.

Imaging software such as Imaris or Fiji was used to generate the figures. For the representation of TCF1 staining in CD8^+^ T cells, CD8^+^ T cell masks were identified using cellpose v3.0.8 [40] and overlayed on the image in Fiji. TCF1 staining was shown within CD8^+^ T cells to generate Figure 5A.

### 4.13 Surfaces Extraction Using Imaris

Imaris (Imaris version 9.9.1) was used to extract surfaces (or masks) of cells and features of the TME.

Briefly, cDC1, CD8^+^ T cell, CD4^+^ T cell and TCF1 surfaces were generated by smoothing the data using a surface detail of 1.66 µm, and the thresholding was done after background subtraction. The thresholding was done by manually adjusting the minimum signal above the background. For CD8^+^ T cells, touching cells were separated into distinct surfaces by enabling the “split touching objects” function based on morphological split. Objects below or above a certain size were filtered out by manually setting up the thresholds.

To extract surfaces of tumour cells and endothelial cells, tumour cell areas and blood vessels were artificially divided into smaller surfaces that could be used to capture the distribution of endothelial “cells” and tumour “cells” by CytoMAP. As for the cDC1s and CD8^+^ T cells, blood vessel surfaces were generated using the smoothing function and background subtraction. After removing the background signal by setting a threshold manually, the vessel structures were artificially separated into smaller surfaces using the function “split touching objects”. A similar strategy was used to generate the tumour cell surfaces, but a surface detail of 5 µm was used. For some tumour sections where tumour cells had not been stained, autofluorescence from other channels and/or serial sections stained for tumour cells were used to manually identified tumour regions in Imaris.

IL12b and Cxcl9 surfaces were extracted using a similar strategy as described above. cDC1s were first generated in Imaris as described above and then classified as *Il12b* or *Cxcl9* based on the overlap (generated with the surface‐to‐surface statistic functions of Imaris) between the cDC1 surfaces and the respective RNAscope probe surfaces.

Metadata for individual surfaces were extracted using the “Statistics” function of Imaris.

### 4.14 Image Registration

Serial sections were registered using the bUnwarpJ plugin in Fiji. A transformation matrix was obtained by specifying a source and a target image, corresponding to the two serial sections to be aligned. Landmark points were added on the source image and moved manually in the target image to identify corresponding regions in the two images. bUnwarpJ was used to generate the transformation matrix based on the respective landmark. The transformation matrix was converted into a raw transformation matrix and applied to the coordinates of Imaris surfaces.

### 4.15 CytoMAP Analysis

Individual csv files were generated for each cell type and each section using R. Briefly, the files generated by Imaris were merged into tables of features (including Position X, Position Y, Position Z, intensity of channels, etc.) for each of the surfaces from a cell type and exported as csv file. The CytoMAP platform was run locally on MATLAB. Samples, each corresponding to a folder containing four csv files (one for CD8^+^ T cells, one for blood vessels, one for cDC1s and one for the tumour cells), were loaded on the CytoMAP platform. Raster‐scanned neighbourhoods of 50 µm were generated for each sample, and neighbourhoods were classified into regions using NN self‐organising map and based on the cell type composition of the neighbourhoods. To choose the number of regions, we evaluated the Davies Bouldin and Calinski‐Harabasz values obtained for different numbers of clusters using a dataset of nine samples from one experiment on the CytoMAP platform. When plotting these values for each number of clusters obtained, we identified six as a good number of regions giving rise to a high Calinski‐Harabasz value and a low Davies Bouldin value, while marking an elbow on the respective plots. In addition, CytoMAP was also used to calculate distances to cells, masks and manually‐defined polygons. Statistics were exported to obtain the regions’ compositions (as mean number of CD8^+^ T cells, blood vessels, tumour cells, and cDC1s per neighbourhood), the classification of individual cells and neighbourhoods into regions, and, when applicable, the distance of individual cells to specific features. When stated, the region interaction heatmap was exported for each tumour to represent the percentage of shared borders between regions.

Plots showing CytoMAP regions and/or cells on tumour sections were generated using R.

### 4.16 Generating TCF1^+^ CD8^+^ T Cell‐Enriched Regions

To calculate distances of cDC1s and cDC1 states to regions enriched in TCF1^+^ CD8^+^ T cells, density distributions of TCF1^+^ CD8^+^ T cells were generated in MATLAB using the ksdensity function based on the coordinates of TCF1^+^ CD8^+^ T cells. A Bandwidth of 100 and BoundaryCorrection reflection were used to calculate the ksdensity densities. Regions enriched in TCF1^+^ CD8^+^ T cells were established by thresholding the distributions above 0.5 × 10^7^ to represent regions dense in TCF1^+^ CD8^+^ T cells.

### 4.17 Analysis of MERFISH Dataset

Data were downloaded from https://zenodo.org/records/7758080 [12]. Segmented cells were analysed separately using Seurat for each patient. *CCR7* and *CXCL9* cDC1s were identified by first filtering out cells for which *XCR1* counts were lower than 3 and generating UMAP embedding and clustering using Seurat as described above. Cells in clusters enriched in either *CCR7* or *CXCL9* were exported for the following analyses.

Of note, only three samples included clear populations of *CCR7* or *CXCL9* cDC1s and were used for follow‐up analyses: Sample 1003, Sample 1012_0 and Sample 1012_1. For these samples, segmented cells enriched in *TCF7* were identified using a similar strategy: segmented cells, for which *CD3E* counts were higher than 5, were clustered using Seurat and clusters enriched in *TCF7* transcripts were kept. Regions enriched in *TCF7* cells were generated as described above. Distances of *CCR7* and *CXCL9* cDC1s to these masks were calculated using CytoMAP as described above.

### 4.18 Statistics

GraphPad Prism software (GraphPad) was used to generate statistical analyses. The means and the standard error of the mean SEM were plotted, unless stated otherwise. The statistical analyses used are indicated in the figure legends.

## Author Contributions

**Conceptualisation**: Cécile Piot, Caetano Reis e Sousa. **Experimental design**: Cécile Piot, Mariana Pereira da Costa, Adi Biram, Carlos M. Minutti, Michael D. Buck, Neil Rogers, Caetano Reis e Sousa. **Experimental procedures/investigation**: Cécile Piot, Mariana Pereira da Costa, Adi Biram, Carlos M. Minutti, Kok Haw Jonathan Lim, Mary Green, Ania Mikolajczak, Lucy Meader, Michael D. Buck, Ana Cardoso, Neil Rogers. **Data analysis**: Cécile Piot, Robert P. Jenkins. **Writing–original draft**: Cécile Piot, Caetano Reis e Sousa. **Writing–revisions**: Cécile Piot, Caetano Reis e Sousa. **Funding acquisition**: Caetano Reis e Sousa. **Project administration**: Caetano Reis e Sousa. **Provision of resources**: Caetano Reis e Sousa, Erik Sahai.

## Conflicts of Interest

C.R.S. is a founder of Adendra Therapeutics and owns stock options in or is a paid consultant for Adendra Therapeutics, Montis Biosciences and Bicycle Therapeutics, all unrelated to this work. E.S. receives grant support from Novartis and consults for Phenomic AI. The remaining authors declare no conflicts of interest.

## Peer Review

The peer review history for this article is available at https://publons.com/publon/10.1002/eji.70011.

## Supporting information




**Supporting File 1**: eji70011‐sup‐0001‐SuppMat.pdf.

## Data Availability

The scRNAseq data that support the findings of this study are openly available in Gene Expression Omnibus under accession number GSE299649.
