# Supplementary material for: Spatial Organisation of Tumour cDC1 States Correlates with Effector and Stem‐Like CD8+ T Cells Location
Source: Eur J Immunol. 2025 Jul 31;55(8):e70011. doi: 10.1002/eji.70011 (PMC12314343; doi:10.1002/eji.70011)
Supplement: Supplementary file 1 — Supporting File 1: eji70011‐sup‐0001‐SuppMat.pdf. [file EJI-55-e70011-s001.pdf]

Supplementary Information

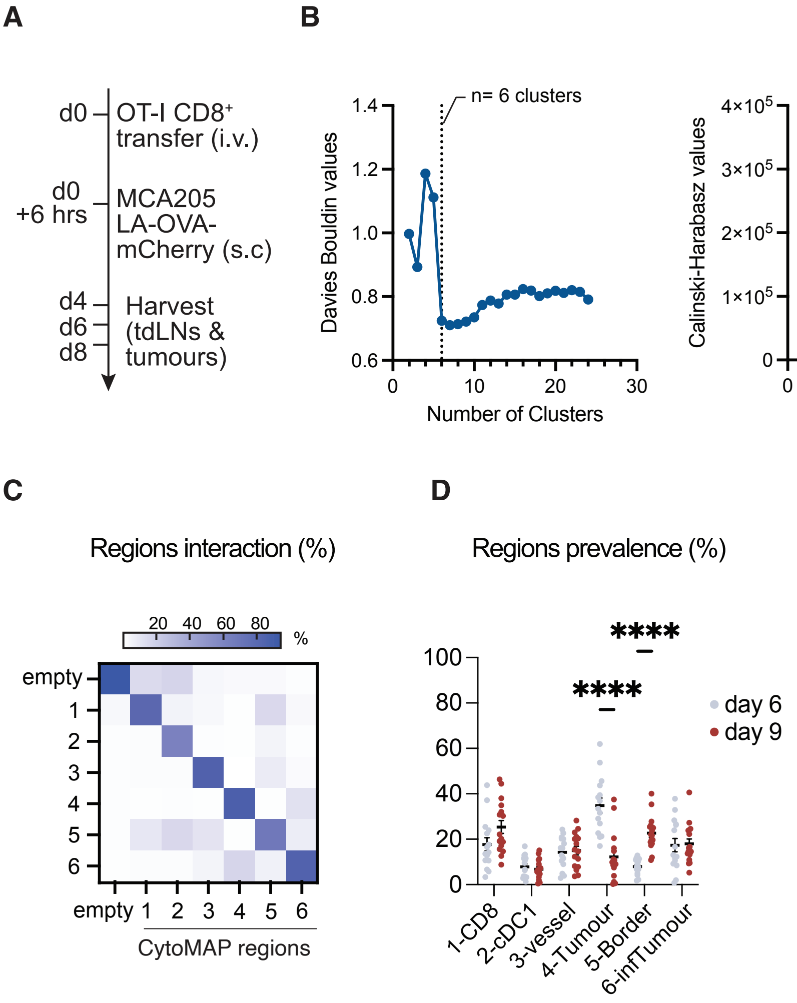

Fig. S1. Related to Fig. 1.

A. Experimental setup used to characterise OVA-specific CD8+ T cells (OT-I) priming and expansion in tdLNs and tumours. 1'000 naïve CD45.1+ CD8+ OT-I were transferred i.v. into WT mice and tumours were injected 6 hours later. TdLNs and tumours were harvested 4, 6 and 8 days later for flow cytometry.

B. Identification of the number of regions to cluster the RSNs for the CytoMAP analysis. The Davies Bouldin and Calinski-Harabasz values were calculated when using different number of clusters (2-24 clusters) on a dataset of 9 independent tumour sections, using the CytoMAP platform. Small and large Davies Bouldin and Calinski-Harabasz values, respectively, correspond to better clustering solutions. After consideration of the values "elbow-plots" plotted here, a value of 6 clusters (indicated with a dotted line) was decided for subsequent RSNs clustering of tumour cDC1s, CD8+ T cells, blood vessels and tumour cells.

C. Interactions between CytoMAP Regions as % of shared borders between regions.

Shown are the mean percentages of samples from one experiment with n= 9 independent whole tumour sections. Representative of two experiments with at least 9 samples per experiment.

D. CytoMAP Regions prevalence on day 6 and day 9. RSNs were classified into CytoMAP regions, and each dot represents the percentage of RSN classified as each region for one sample. Data are pooled from three independent experiments with 4-6 independent whole tumour sections per time points and per experiment. Statistical analysis was performed using a 2-way ANOVA test and comparing the mean of day 6 with the mean of day 9 for each region using a Šídák's multiple comparisons test.

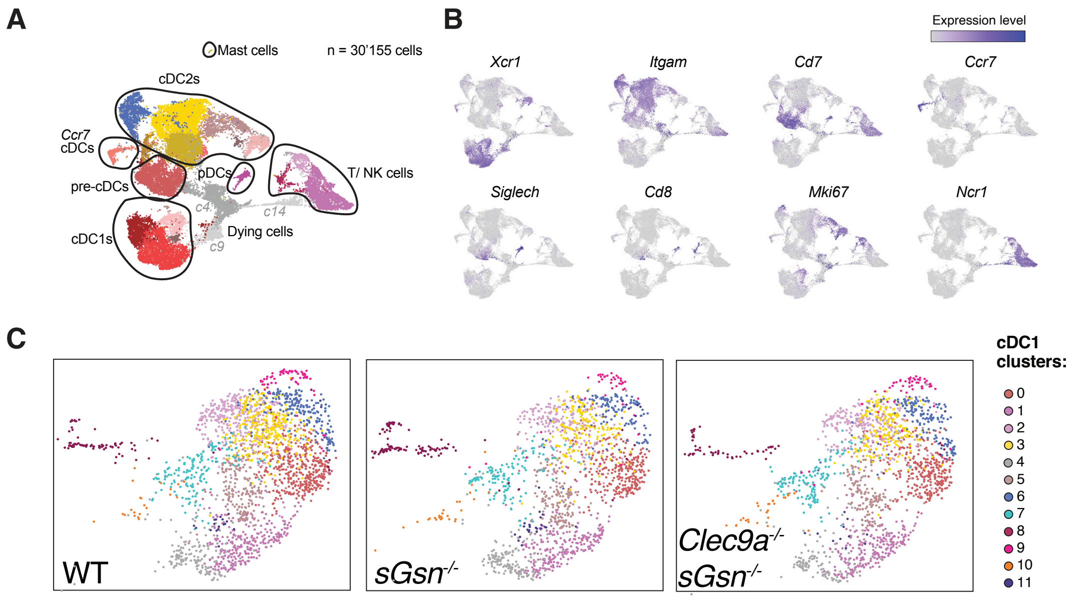

Fig. S2. Related to Fig.2

A. UMAP representation of the scRNAseq data from Figure 2 showing regions of cDC2s, T/NK cells, pDCs, cDC1s, pre-cDCs, *Ccr7* cDCs, and a small cluster of mast cells. In grey are highlighted dying cells.

B. Expression of selected genes used to annotate the UMAP in **A**. *Xcr1*, *Itgam, Cd7* and *Ccr7* were used (amongst other) to identify cDC1s, cDC2s, pre-cDCs and *Ccr7* cDCs respectively. *Siglech, Cd8, Mki67* and *Nrc1* were used to identify pDCs, $CD8^{+}$ T cells, proliferating cells and NK cells respectively.

C. cDC1 clusters on the UMAP from Figure 2A (zoomed on the cDC1s) split by genotype.

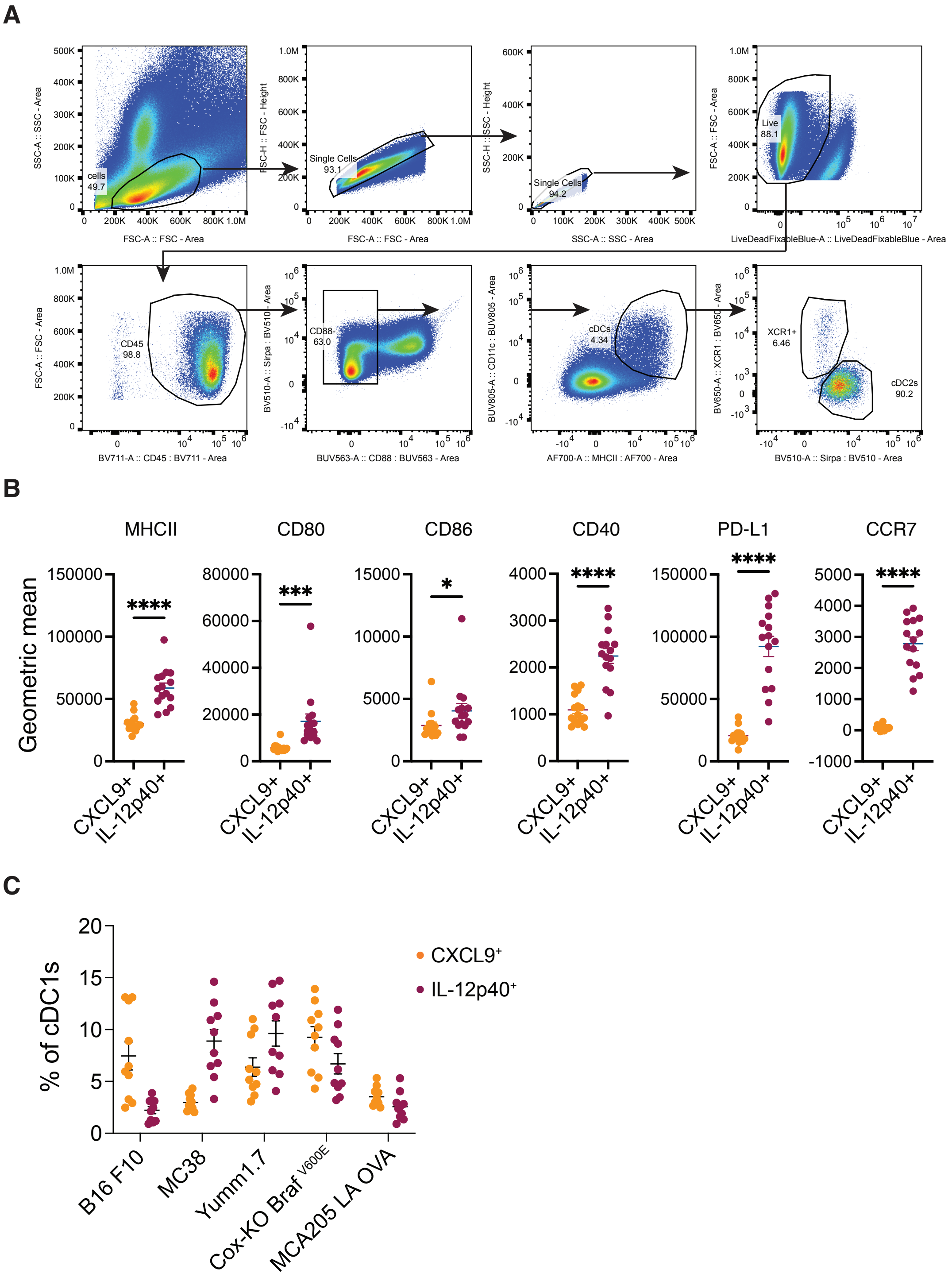

Fig. S3. Related to Fig.3

A. Representative flow cytometry gating strategy of tumour cDC1s and cDC2s.

B. Geometric mean of MHCII, CD80, CD86, CD40, PD-L1 and CCR7 expression by flow cytometry of CXCL9+ and IL-12p40+ cDC1s from MCA205 LA-OVA tumours. n=15 independent samples, from two experiments. Statistical test: paired t-test.

C. Flow cytometry analysis of CXCL9 and IL-12p40 expression by tumour cDC1s across different transplantable tumour models. n =10 independent tumour samples per tumour model, from two experiments.

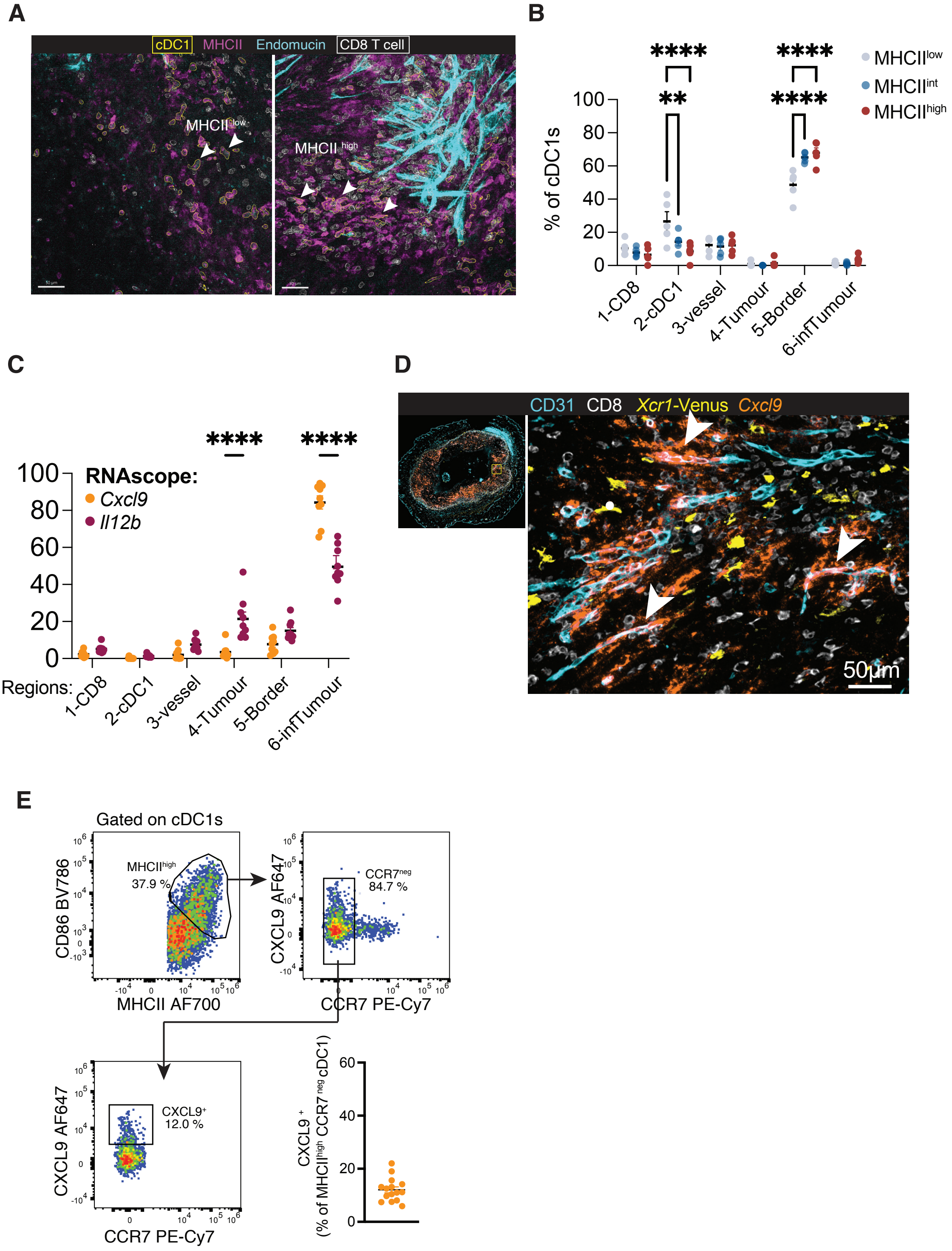

Fig. S4. Related to Fig.4

A. Representative images of MHCII expression in cDC1s in the stroma (left) or close to a vascularised tumour border region (right). Tumours were harvested from *Xcr1*-Venus mice on day 9 and cDC1s were segmented in Imaris based on Venus expression. White arrows show MHCII^{low} cDC1s (left panel) and MHCII^{high} cDC1s (right panel).

B. Distribution of MHCII^{low} (in grey), MHCII^{int} (in blue) and MHCII^{high} (in red) cDC1s across CytoMAP regions generated based on the abundance of CD8^{+} T cells, cDC1s, blood vessels and tumour cells, as described in Figure 1B. n= 5 independent whole tumour sections, from one experiment. Statistical analysis is 2-way ANOVA test and comparing the mean of MHCII^{low} , MHCII^{int} and MHCII^{high} cDC1s.
C. Distribution of *Cxcl9* and *Il12b* expression across CytoMAP regions. Puncti of *Cxcl9* and *Il12b* expression were segmented by Imaris and associated to CytoMAP regions based on the shortest distance to RSNs from Region 1-6. n=9 independent whole tumour sections, from Figure 4. Statistical analysis: 2-way ANOVA and comparing the means for *Cxcl9* and *Il12b* puncti.
D. Representative image of *Cxcl9* expression in the parenchyma. Tumour sections from Figure 4. White arrows highlight *Cxcl9* expression around blood vessels
E. CXCL9 expression in MHCII^{high} CCR7^{neg} tumour cDC1s by flow cytometry. Flow cytometry dot plots show data pooled from 10 samples from one experiment. Quantification (bottom right) depicts n = 15 samples from two experiments.

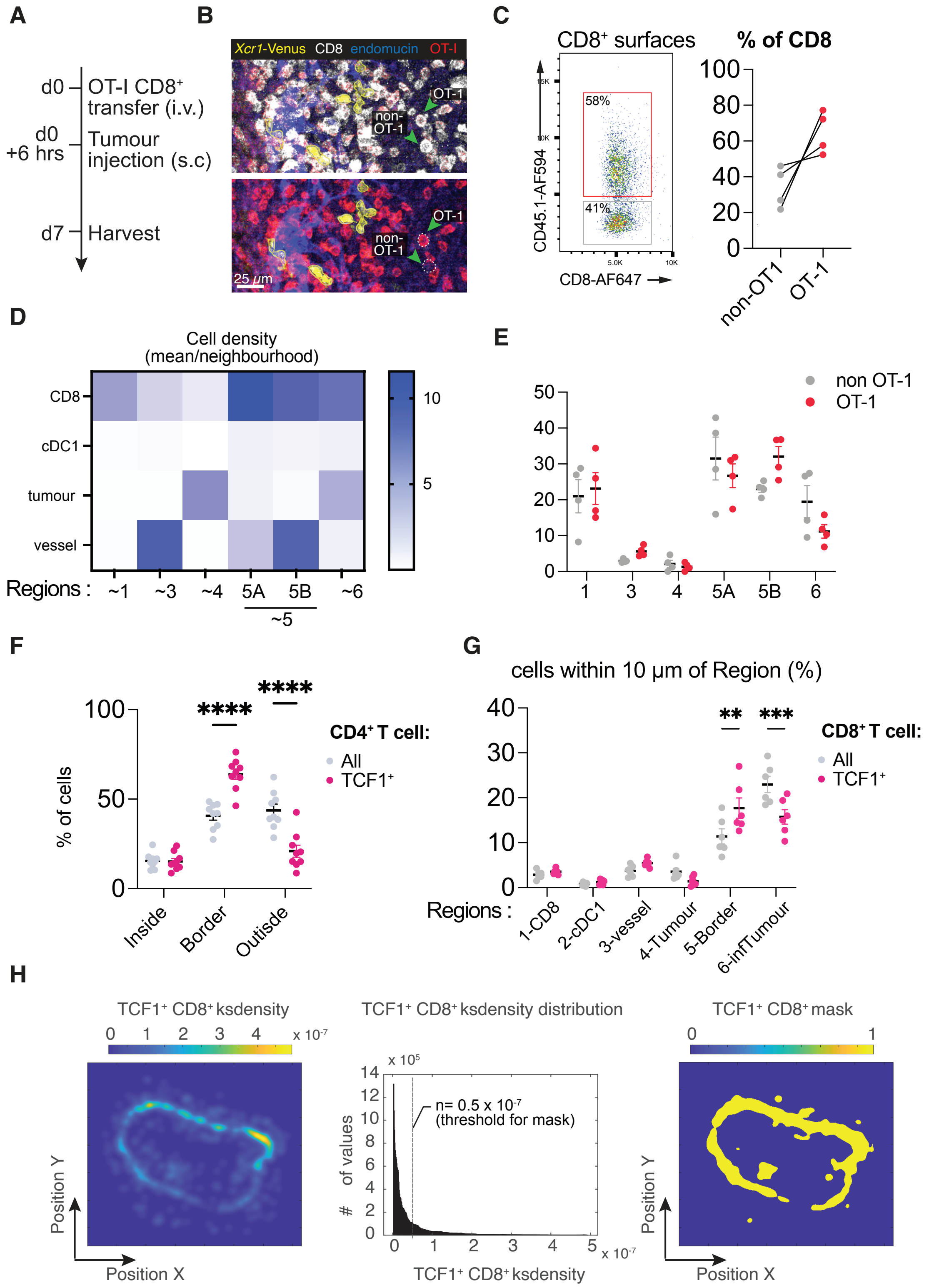

Fig. S5. Related to Fig.5

A. Experimental setup used to characterise the location of OVA-specific CD8$^+$ T cells (OT-I) in tumours. Naïve CD45.1$^+$ CD8$^+$ OT-I were transferred i.v. and tumours were injected on the same day. Tumours were harvested 7 days after.

B. Upper panel: Representative IF image of total $CD8^+$ T cells (in white), $CD45.1^+$ OT-I T cells (in red), endomucin (in blue) and cDC1s (in yellow). Green arrows indicate examples of OT-I and non-OT-I $CD8^+$ T cells. Lower panel: Same IF image but without the CD8 staining. Dotted circles indicate cells highlighted with the green arrows.
C. Representative histocytometry plot of $CD8^+$ T cell surfaces extracted using Imaris and classified as non-OT-I or OT-I based on CD45.1-AF594 mean intensity for one tumour section (left panel). Quantification of the histocytometry gates showing non-OT-I and OT-I as percentage of $CD8^+$ T cell per tumour section (right panel). Each dot represents a whole tumour section. n = 4 independent whole tumour sections from one experiment.
D. Heatmap showing the density of $CD8^+$ T cells, cDC1s, tumour cells and vessels per CytoMAP regions for tumours of Figure S5A as mean number per neighbourhood. CytoMAP regions were generated as described above to describe the location of OT-I and non OT-I T cells. The interpretation of the resulting CytoMAP regions was different from the CytoMAP analysis above but were annotated to best match the previous annotations from Figure 1.
E. Distribution of non OT-I (in grey) and OT-I (in red) $CD8^+$ T cells across CytoMAP regions from D. Statistical analysis was performed using a 2 way ANOVA and comparing the mean for the OT-I and the OT-I per CytoMAP regions.
F. Similar to Figure 4B but for $CD4^+$ T cells. Quantification shows the percentage of $CD4^+$ T cells (in grey) and $TCF1^+$ $CD4^+$ T cells (in magenta) “inside”, “at the border” and “outside” of the tumour as done for Figure 4B. $CD4^+$ T cells and $TCF1^+$ $CD4^+$ T cells were segmented using Imaris and the shortest distance of each cell to the tumour border was calculated. Statistical test: 2-way ANOVA test and comparing the mean of total $CD4^+$ T cells and $TCF1^+$ $CD4^+$ T cells. n = 9 independent whole tumour sections, from 2 experiments.
G. Related to Figure 4D. Quantification shows the percentage of total $CD8^+$ T cells (in grey) and $TCF1^+$ $CD8^+$ T cells (in magenta) from Figure 4B inside or within 10 µm of CytoMAP regions. Statistical analysis: 2-way ANOVA test and comparing the mean of total $CD8^+$ T cells and $TCF1^+$ $CD8^+$ T cells. n = 6 independent whole tumour sections, from 2 experiments.
H. Representative image of the ksdensity of $TCF1^+$ $CD8^+$ cells on a tumour section (left) and the respective distribution of ksdensity values plotted as histogram (middle). A threshold of 0.5×10^{-7} was used to create the $TCF1^+$ $CD8^+$ masks (right).

Table S1

DEGs of cDC1 clusters (Figure 2A) were generated using the FindAllMarkers function of Seurat, and the top 5 DEGs for each cluster were generated by ranking the genes based on the average log2FC.

cDC1 clusters Top 5 DEGs (clusters from Figure 2A)

	p_val	avg_log2FC	pct.1	pct.2	p_val_adj	cluster	gene
S100a41	2.24E-189	1.98494516	0.883	0.405	4.16E-185	4	S100a4
Cd209a	3.94E-160	1.30438433	0.42	0.065	7.32E-156	4	Cd209a
S100a61	3.10E-133	1.35866292	0.98	0.878	5.76E-129	4	S100a6
Fdps1	2.11E-90	1.32353134	0.771	0.428	3.92E-86	4	Fdps
Ifitm11	6.44E-58	1.53510063	0.686	0.42	1.20E-53	4	Ifitm1
Fdps	2.22E-108	0.91234161	0.759	0.409	4.13E-104	1	Fdps
Ifitm2	1.48E-99	0.98698845	0.988	0.954	2.74E-95	1	Ifitm2
Il1b	1.68E-73	0.94863524	0.956	0.887	3.13E-69	1	Il1b
Ifitm1	4.28E-68	1.87400256	0.648	0.41	7.95E-64	1	Ifitm1
Plet1	6.11E-59	0.95861522	0.346	0.131	1.14E-54	1	Plet1
mt-Atp62	2.33E-38	2.51333521	1	0.996	4.32E-34	11	mt-Atp6
mt-Co31	4.87E-38	2.14240119	1	0.999	9.05E-34	11	mt-Co3
mt-Co21	2.35E-37	2.22513958	1	0.998	4.37E-33	11	mt-Co2
mt-Nd2	4.68E-37	2.39320372	1	0.977	8.70E-33	11	mt-Nd2
mt-Cytb1	2.04E-34	2.39930912	1	0.989	3.78E-30	11	mt-Cytb
Manf	8.73E-232	1.85177693	0.986	0.773	1.62E-227	5	Manf
Creld2	2.91E-222	1.48081045	0.867	0.321	5.41E-218	5	Creld2
Hsp90b1	1.07E-214	1.51465924	0.998	0.945	1.98E-210	5	Hsp90b1
Sdf2l1	1.16E-212	1.81225313	0.971	0.716	2.16E-208	5	Sdf2l1

Hspa5	1.00E-203	1.490695	0.998	0.979	1.87E-199	5	Hspa5
H2afz	2.34E-133	0.65137938	1	0.989	4.35E-129	0	H2afz
Lyz2	3.31E-107	0.80166463	0.995	0.897	6.15E-103	0	Lyz2
Gpr65	1.36E-92	0.61876464	0.959	0.787	2.52E-88	0	Gpr65
Cd81	2.50E-90	0.6038437	0.798	0.493	4.65E-86	0	Cd81
Mt1	2.30E-72	0.86399724	0.905	0.704	4.27E-68	0	Mt1
Hspa1a1	1.61E-197	1.68967788	0.975	0.596	2.99E-193	3	Hspa1a
Hspa1b	5.97E-176	1.41496114	0.924	0.473	1.11E-171	3	Hspa1b
Fos1	4.22E-171	1.15154637	1	0.973	7.83E-167	3	Fos
Egr1	2.12E-167	1.24120917	0.994	0.761	3.95E-163	3	Egr1
Jun	7.12E-158	1.36848262	0.975	0.802	1.32E-153	3	Jun
Gm42418	2.32E-123	1.96326948	1	1	4.31E-119	6	Gm42418
Ddx3x	6.02E-109	1.13827387	0.871	0.735	1.12E-104	6	Ddx3x
Dennd4a	4.14E-105	1.10593937	0.954	0.899	7.70E-101	6	Dennd4a
Tob21	5.71E-92	1.15277954	0.882	0.739	1.06E-87	6	Tob2
Dusp12	6.44E-66	1.15390816	0.954	0.879	1.20E-61	6	Dusp1
Ccl2	2.90E-171	1.77167169	0.641	0.213	5.38E-167	2	Ccl2
Ccl7	9.07E-139	1.85168629	0.33	0.058	1.68E-134	2	Ccl7
Ier3	3.96E-84	0.94863956	0.656	0.346	7.35E-80	2	Ier3
Klf2	6.37E-68	0.67258873	0.925	0.723	1.18E-63	2	Klf2
Ccl3	6.04E-51	0.79783837	0.308	0.116	1.12E-46	2	Ccl3
Ifit2	0	1.90150097	0.659	0.072	0	7	Ifit2
Ifi204	5.67E-268	1.7620789	0.915	0.238	1.05E-263	7	Ifi204

Isg15	4.16E-261	2.86191608	0.976	0.379	7.73E-257	7	Isg15
Irf7	1.71E-238	2.46421635	0.998	0.549	3.18E-234	7	Irf7
Slfn51	1.67E-176	2.05692063	0.991	0.754	3.10E-172	7	Slfn5
Nfkbiz1	3.52E-136	2.08651402	0.914	0.21	6.54E-132	9	Nfkbiz
Dusp22	2.55E-114	2.50104376	0.951	0.309	4.74E-110	9	Dusp2
Nfkbia2	3.32E-93	2.43747754	1	0.7	6.18E-89	9	Nfkbia
Icam12	5.03E-74	1.86174784	0.938	0.526	9.34E-70	9	Icam1
Egr12	1.49E-51	1.8422854	0.994	0.783	2.77E-47	9	Egr1
Serpina3g	2.04E-188	2.3666988	0.737	0.038	3.80E-184	10	Serpina3g
Cxcl93	5.82E-129	4.76678545	0.921	0.106	1.08E-124	10	Cxcl9
Cxcl102	2.40E-95	4.05063229	0.711	0.076	4.47E-91	10	Cxcl10
Irf14	1.60E-54	2.29098222	0.974	0.325	2.97E-50	10	Irf1
Ccl171	1.57E-13	2.07131892	0.237	0.05	2.91E-09	10	Ccl17
Ccr7	0	4.56913441	0.827	0.015	0	8	Ccr7
Il12b	0	4.25355436	0.698	0.035	0	8	Il12b
Ccl22	0	4.04046222	0.78	0.035	0	8	Ccl22
Samsn1	3.37E-188	3.77536514	0.886	0.235	6.27E-184	8	Samsn1
Ccl5	3.59E-125	4.802528	0.62	0.134	6.67E-121	8	Ccl5

cDC1 clusters all DEGs (clusters from Figure 2A)

	p_val	avg_log2FC	pct.1	pct.2	p_val_adj	cluster	gene
H2afz	2.34E-133	0.65137938	1	0.989	4.35E-129	0	H2afz
Lyz2	3.31E-107	0.80166463	0.995	0.897	6.15E-103	0	Lyz2

Sub1	1.18E-103	0.42711426	0.998	0.981	2.19E-99	0	Sub1
Gpr65	1.36E-92	0.61876464	0.959	0.787	2.52E-88	0	Gpr65
Cd81	2.50E-90	0.6038437	0.798	0.493	4.65E-86	0	Cd81
Gpcpd1	7.53E-86	0.57637571	0.907	0.671	1.40E-81	0	Gpcpd1
H2-Aa	5.57E-83	0.24726148	1	0.999	1.04E-78	0	H2-Aa
Mt1	2.30E-72	0.86399724	0.905	0.704	4.27E-68	0	Mt1
Sep-03	8.26E-72	0.45189764	0.956	0.694	1.53E-67	0	Sep-03
BC028528	1.15E-66	0.40487115	0.984	0.859	2.14E-62	0	BC028528
Cst3	4.99E-64	0.27076402	1	1	9.27E-60	0	Cst3
Atox1	4.34E-63	0.31529949	1	0.966	8.06E-59	0	Atox1
H2-Eb1	1.99E-62	0.24696501	1	0.999	3.69E-58	0	H2-Eb1
Atpif1	1.40E-58	0.34917055	0.999	0.956	2.59E-54	0	Atpif1
Jdp2	6.72E-58	0.49511981	0.705	0.447	1.25E-53	0	Jdp2
Pianp	9.04E-54	0.30056382	0.638	0.359	1.68E-49	0	Pianp
Btg1	1.33E-53	0.34263992	1	0.986	2.46E-49	0	Btg1
Cd9	2.58E-53	0.33682417	0.983	0.883	4.79E-49	0	Cd9
Ckb	6.10E-51	0.35134411	0.999	0.959	1.13E-46	0	Ckb
Slc25a20	6.15E-51	0.34719062	0.89	0.688	1.14E-46	0	Slc25a20
Ncoa7	6.69E-51	0.40504589	0.927	0.783	1.24E-46	0	Ncoa7
Got1	8.11E-51	0.35670373	0.873	0.659	1.51E-46	0	Got1
Plek	3.22E-50	0.35447126	0.985	0.937	5.99E-46	0	Plek
Stk38l	9.44E-50	0.29394077	0.439	0.217	1.75E-45	0	Stk38l
Arpc2	3.80E-49	0.22257606	0.999	0.988	7.05E-45	0	Arpc2

Plpp1	5.28E-49	0.29365955	0.505	0.266	9.82E-45	0	Plpp1
Rps29	5.48E-49	0.20557531	1	0.999	1.02E-44	0	Rps29
Il18	1.10E-48	0.33732872	0.729	0.465	2.04E-44	0	Il18
Rps13	1.27E-48	0.23768471	1	0.991	2.37E-44	0	Rps13
Rplp1	5.59E-48	0.22223266	1	0.996	1.04E-43	0	Rplp1
Zdhhc23	1.10E-47	0.33689387	0.735	0.493	2.05E-43	0	Zdhhc23
Naga	3.12E-47	0.29145769	0.993	0.907	5.79E-43	0	Naga
Unc119	1.57E-46	0.32174041	0.97	0.824	2.92E-42	0	Unc119
Btg2	3.58E-46	0.32575286	1	0.979	6.66E-42	0	Btg2
Gm2a	3.62E-45	0.28667083	0.999	0.962	6.73E-41	0	Gm2a
Ndufa6	3.76E-45	0.29007393	0.996	0.931	6.99E-41	0	Ndufa6
Fau	5.16E-45	0.21471196	1	0.999	9.59E-41	0	Fau
Eef1b2	4.32E-43	0.24812379	1	0.984	8.03E-39	0	Eef1b2
Ndel1	1.21E-42	0.3097051	0.89	0.718	2.24E-38	0	Ndel1
Agpat4	1.86E-42	0.2864906	0.845	0.634	3.45E-38	0	Agpat4
Kctd12	1.80E-41	0.33137305	0.99	0.926	3.34E-37	0	Kctd12
Sirt2	9.99E-40	0.30848819	0.727	0.493	1.86E-35	0	Sirt2
Rpl36	1.04E-39	0.23583626	1	0.989	1.94E-35	0	Rpl36
Rpl34	5.69E-39	0.21440534	1	0.991	1.06E-34	0	Rpl34
Ubald2	6.86E-39	0.30728491	0.885	0.709	1.27E-34	0	Ubald2
Sri	9.67E-39	0.29306551	0.992	0.918	1.80E-34	0	Sri
S100a11	1.43E-38	0.30093825	1	0.983	2.66E-34	0	S100a11
Ppt1	1.90E-38	0.26599859	0.994	0.925	3.54E-34	0	Ppt1

Rtl8a	2.14E-38	0.29464626	0.659	0.431	3.97E-34	0	Rtl8a
Rps28	2.17E-38	0.20575149	1	0.995	4.03E-34	0	Rps28
Rpl30	3.39E-38	0.20058441	1	0.992	6.29E-34	0	Rpl30
Egr3	5.32E-38	0.35822854	0.872	0.659	9.89E-34	0	Egr3
Cox7c	6.20E-38	0.22935502	0.999	0.967	1.15E-33	0	Cox7c
Gpr171	1.40E-37	0.35412832	0.951	0.818	2.59E-33	0	Gpr171
Fgfr1	2.08E-37	0.21842324	0.714	0.463	3.87E-33	0	Fgfr1
Bcl2a1a	6.34E-37	0.25003706	0.586	0.359	1.18E-32	0	Bcl2a1a
Nfil3	1.36E-36	0.28760072	0.686	0.463	2.53E-32	0	Nfil3
Ptpn18	1.47E-35	0.25234974	0.998	0.94	2.73E-31	0	Ptpn18
Prelid2	3.15E-35	0.27388289	0.547	0.34	5.85E-31	0	Prelid2
Nr4a2	2.48E-34	0.32569657	0.977	0.878	4.61E-30	0	Nr4a2
Bcl2a1d	5.05E-34	0.20049208	0.842	0.632	9.39E-30	0	Bcl2a1d
Rnase6	7.45E-33	0.27775598	0.961	0.849	1.38E-28	0	Rnase6
H2-Ob	4.73E-32	0.25992647	0.896	0.728	8.78E-28	0	H2-Ob
Ucp2	5.48E-32	0.23542856	0.998	0.961	1.02E-27	0	Ucp2
Rab11fip1	4.01E-31	0.22287779	0.844	0.654	7.45E-27	0	Rab11fip1
Rtl8b	3.01E-30	0.26477723	0.595	0.393	5.60E-26	0	Rtl8b
Psmb9	5.67E-30	0.21651658	0.999	0.956	1.05E-25	0	Psmb9
Atp6v0e	6.56E-30	0.23284551	0.994	0.949	1.22E-25	0	Atp6v0e
Uqcrb	1.08E-29	0.2517246	0.987	0.903	2.02E-25	0	Uqcrb
Atp5md	1.19E-29	0.25598699	0.996	0.926	2.20E-25	0	Atp5md
P2ry10	1.23E-29	0.20760826	0.951	0.814	2.29E-25	0	P2ry10

St3gal5	2.00E-29	0.25468431	0.882	0.713	3.71E-25	0	St3gal5
Cd14	1.76E-28	0.41547818	0.351	0.196	3.28E-24	0	Cd14
Litaf	3.03E-28	0.25263831	0.836	0.676	5.64E-24	0	Litaf
Tent5c	4.24E-28	0.22668794	0.919	0.758	7.88E-24	0	Tent5c
Hfe	9.30E-28	0.27201664	0.821	0.633	1.73E-23	0	Hfe
Zfand5	9.43E-28	0.31265035	0.97	0.874	1.75E-23	0	Zfand5
Havcr2	1.24E-27	0.23263904	0.827	0.669	2.30E-23	0	Havcr2
Gna13	1.62E-27	0.22653504	0.923	0.814	3.01E-23	0	Gna13
Lmnb1	7.20E-27	0.23997121	0.96	0.87	1.34E-22	0	Lmnb1
Rab43	1.10E-26	0.20026682	0.966	0.898	2.04E-22	0	Rab43
Sgk1	1.18E-26	0.36250592	0.619	0.454	2.19E-22	0	Sgk1
Anxa1	1.27E-26	0.25544367	0.971	0.865	2.37E-22	0	Anxa1
Tbca	1.65E-26	0.21871499	0.988	0.912	3.07E-22	0	Tbca
Mrpl42	2.27E-26	0.25803564	0.887	0.75	4.21E-22	0	Mrpl42
Mkrn1	3.03E-26	0.24180824	0.905	0.766	5.63E-22	0	Mkrn1
Ccdc107	3.21E-26	0.24031354	0.666	0.488	5.96E-22	0	Ccdc107
Tdgf1	1.15E-25	0.23491053	0.211	0.094	2.14E-21	0	Tdgf1
Stk17b	3.70E-25	0.24989956	0.97	0.877	6.88E-21	0	Stk17b
Fcgr2b	5.60E-25	0.21111895	0.835	0.642	1.04E-20	0	Fcgr2b
Cnbp	6.81E-25	0.20520047	0.99	0.93	1.26E-20	0	Cnbp
Eps8	1.58E-24	0.20404724	0.839	0.662	2.93E-20	0	Eps8
Rheb	2.38E-24	0.22393446	0.924	0.801	4.43E-20	0	Rheb
Arf2	3.08E-24	0.25981318	0.562	0.396	5.73E-20	0	Arf2

Eif4e	4.09E-24	0.22276314	0.751	0.583	7.60E-20	0	Eif4e
Nt5c	6.05E-23	0.22264659	0.929	0.809	1.12E-18	0	Nt5c
Lgals3	9.79E-23	0.20342173	0.999	0.973	1.82E-18	0	Lgals3
Timd4	2.47E-22	0.29750127	0.26	0.137	4.58E-18	0	Timd4
Arf6	7.87E-22	0.21441225	0.864	0.723	1.46E-17	0	Arf6
Cd300c2	1.05E-21	0.21576463	0.802	0.619	1.94E-17	0	Cd300c2
Hepacam2	1.24E-20	0.20386109	0.658	0.479	2.30E-16	0	Hepacam2
Sep-06	1.24E-20	0.20420051	0.928	0.801	2.31E-16	0	Sep-06
Lmna	1.45E-20	0.22585398	0.703	0.523	2.69E-16	0	Lmna
Vps37b	3.53E-20	0.28011184	0.736	0.588	6.56E-16	0	Vps37b
Clec9a	3.12E-16	0.24099853	0.63	0.526	5.79E-12	0	Clec9a
Fth1	4.20E-203	0.83753552	0.999	0.995	7.79E-199	1	Fth1
Spint1	1.52E-181	0.62505505	0.567	0.145	2.82E-177	1	Spint1
Smim3	1.26E-124	0.70355039	0.693	0.322	2.34E-120	1	Smim3
Gng10	1.55E-115	0.61225053	0.965	0.919	2.88E-111	1	Gng10
Tmsb4x	3.17E-115	0.41623801	1	1	5.89E-111	1	Tmsb4x
Dbi	1.01E-113	0.67773114	0.948	0.827	1.88E-109	1	Dbi
Lpcat2	5.81E-113	0.3979581	0.415	0.11	1.08E-108	1	Lpcat2
Fdps	2.22E-108	0.91234161	0.759	0.409	4.13E-104	1	Fdps
Prdx6	3.28E-108	0.67647477	0.883	0.645	6.10E-104	1	Prdx6
Neurl3	1.89E-107	0.65430612	0.7	0.331	3.51E-103	1	Neurl3
Ifi30	3.07E-105	0.67031551	0.988	0.945	5.70E-101	1	Ifi30
Klrd1	4.56E-105	0.81726659	0.68	0.336	8.48E-101	1	Klrd1

Mrpl52	2.58E-101	0.55176035	0.982	0.935	4.80E-97	1	Mrpl52
Mpc1	1.09E-100	0.62419976	0.923	0.758	2.03E-96	1	Mpc1
Ifitm2	1.48E-99	0.98698845	0.988	0.954	2.74E-95	1	Ifitm2
Gdi2	7.42E-98	0.4969773	0.982	0.972	1.38E-93	1	Gdi2
Dab2	3.13E-95	0.67601746	0.535	0.218	5.81E-91	1	Dab2
Aif1	5.41E-93	0.55482821	0.972	0.922	1.01E-88	1	Aif1
Gngt2	6.65E-93	0.32462986	0.378	0.107	1.24E-88	1	Gngt2
Cd52	1.75E-90	0.46941016	1	0.993	3.26E-86	1	Cd52
Tnfaip8	1.53E-89	0.64777329	0.955	0.852	2.83E-85	1	Tnfaip8
Cldn1	5.06E-88	0.29231718	0.304	0.074	9.39E-84	1	Cldn1
Itgax	2.11E-87	0.49301745	0.621	0.297	3.92E-83	1	Itgax
Ndufb1-ps	2.35E-84	0.46649108	0.983	0.946	4.36E-80	1	Ndufb1-ps
Taldo1	3.26E-80	0.4319791	0.991	0.966	6.05E-76	1	Taldo1
Ppia	4.95E-78	0.38370991	0.996	0.986	9.20E-74	1	Ppia
Rpl8	7.57E-78	0.30912808	0.998	0.994	1.41E-73	1	Rpl8
Ctsh	1.44E-77	0.49313418	0.962	0.868	2.68E-73	1	Ctsh
Il1r2	6.17E-77	0.74063693	0.624	0.331	1.15E-72	1	Il1r2
Cmtm7	4.40E-74	0.53727576	0.904	0.75	8.17E-70	1	Cmtm7
Il1b	1.68E-73	0.94863524	0.956	0.887	3.13E-69	1	Il1b
Rpl15	5.59E-71	0.32254172	0.998	0.987	1.04E-66	1	Rpl15
Idi1	5.32E-69	0.38193319	0.482	0.2	9.88E-65	1	Idi1
Ptma	3.48E-68	0.35443316	1	0.992	6.46E-64	1	Ptma
Ifitm1	4.28E-68	1.87400256	0.648	0.41	7.95E-64	1	Ifitm1

Rps8	3.15E-67	0.25588338	0.998	0.996	5.85E-63	1	Rps8
Rps27l	7.18E-67	0.39724866	0.971	0.917	1.33E-62	1	Rps27l
Pomp	3.46E-64	0.36727005	0.988	0.966	6.42E-60	1	Pomp
Rps2	1.13E-63	0.31446981	0.993	0.991	2.09E-59	1	Rps2
Prdx5	1.13E-63	0.50279982	0.884	0.732	2.10E-59	1	Prdx5
Rpl21	3.81E-63	0.29887526	0.993	0.985	7.08E-59	1	Rpl21
Entpd1	1.70E-60	0.37660641	0.455	0.203	3.16E-56	1	Entpd1
Hck	4.57E-60	0.45995591	0.79	0.574	8.48E-56	1	Hck
Rps27a	1.51E-59	0.24371527	1	0.996	2.81E-55	1	Rps27a
Gatm	2.12E-59	0.46874808	0.889	0.711	3.94E-55	1	Gatm
Plet1	6.11E-59	0.95861522	0.346	0.131	1.14E-54	1	Plet1
Lgmn	8.18E-57	0.24909102	0.45	0.197	1.52E-52	1	Lgmn
Grasp	4.83E-56	0.60788678	0.841	0.675	8.98E-52	1	Grasp
Rpl13	6.41E-56	0.24829678	1	0.998	1.19E-51	1	Rpl13
Atp5g1	8.21E-56	0.43531279	0.955	0.886	1.52E-51	1	Atp5g1
Ncf4	3.24E-55	0.43785035	0.923	0.805	6.02E-51	1	Ncf4
Rpl11	4.43E-55	0.26051615	1	0.994	8.23E-51	1	Rpl11
Ctss	6.74E-54	0.3766912	0.994	0.979	1.25E-49	1	Ctss
Cdk2ap2	1.06E-53	0.42590144	0.868	0.692	1.97E-49	1	Cdk2ap2
Gpr1711	2.28E-53	0.52301584	0.948	0.823	4.24E-49	1	Gpr171
Pold4	7.22E-53	0.43854734	0.884	0.754	1.34E-48	1	Pold4
Cotl1	1.08E-52	0.4153981	0.97	0.92	2.00E-48	1	Cotl1
Plac8	1.06E-50	0.51990735	0.916	0.711	1.98E-46	1	Plac8

Cox5a	1.44E-50	0.36062127	0.966	0.903	2.68E-46	1	Cox5a
Cyp51	1.75E-49	0.40831574	0.589	0.345	3.24E-45	1	Cyp51
Psmb8	4.01E-49	0.28669275	0.991	0.986	7.44E-45	1	Psmb8
Snx18	5.16E-49	0.31273058	0.376	0.165	9.59E-45	1	Snx18
Pycard	5.85E-49	0.49641889	0.861	0.732	1.09E-44	1	Pycard
Ears2	6.76E-49	0.38256292	0.359	0.155	1.26E-44	1	Ears2
H2afj	1.41E-47	0.43581928	0.879	0.749	2.61E-43	1	H2afj
Uqcrq	1.48E-46	0.35005762	0.957	0.921	2.75E-42	1	Uqcrq
Nme2	3.66E-46	0.36652388	0.978	0.95	6.80E-42	1	Nme2
Igsf8	4.79E-46	0.2782402	0.41	0.196	8.90E-42	1	Igsf8
Ifitm3	5.02E-46	0.60459928	0.972	0.914	9.34E-42	1	Ifitm3
Rpl28	6.05E-45	0.24335356	0.999	0.992	1.12E-40	1	Rpl28
Nop10	1.29E-44	0.39466181	0.8	0.635	2.40E-40	1	Nop10
Rpl41	1.42E-44	0.20324097	1	0.995	2.64E-40	1	Rpl41
Fxyd5	1.60E-44	0.30781039	0.989	0.968	2.97E-40	1	Fxyd5
Cox8a	1.71E-44	0.30123597	0.987	0.962	3.18E-40	1	Cox8a
Svbp	2.26E-44	0.31418112	0.593	0.353	4.19E-40	1	Svbp
Ndufa4	3.49E-44	0.34167785	0.966	0.923	6.49E-40	1	Ndufa4
Mef2c	6.09E-44	0.43715011	0.744	0.542	1.13E-39	1	Mef2c
Rplp0	5.32E-43	0.24470908	0.999	0.992	9.89E-39	1	Rplp0
Snrpd2	7.67E-43	0.35073939	0.92	0.847	1.43E-38	1	Snrpd2
Tecr	1.19E-42	0.31871046	0.684	0.47	2.21E-38	1	Tecr
Park7	1.23E-42	0.34316095	0.95	0.879	2.28E-38	1	Park7

Ifitm6	1.35E-42	0.41645603	0.318	0.131	2.50E-38	1	Ifitm6
Elob	1.10E-41	0.32039887	0.972	0.934	2.04E-37	1	Elob
Ybx1	2.14E-41	0.30570816	0.99	0.958	3.98E-37	1	Ybx1
Pmepa1	2.97E-41	0.36341033	0.646	0.415	5.52E-37	1	Pmepa1
Atp5h	6.36E-41	0.28461751	0.983	0.957	1.18E-36	1	Atp5h
Naca	1.71E-40	0.25351552	0.995	0.978	3.17E-36	1	Naca
Rps19	1.92E-40	0.22789825	1	0.99	3.57E-36	1	Rps19
Rpl36a	2.13E-40	0.26527699	0.991	0.971	3.95E-36	1	Rpl36a
Ssr4	6.86E-40	0.30760978	0.976	0.924	1.27E-35	1	Ssr4
Rpl14	7.50E-39	0.23518085	0.995	0.984	1.39E-34	1	Rpl14
Slc25a33	9.96E-39	0.35131248	0.304	0.134	1.85E-34	1	Slc25a33
S100a6	6.42E-38	0.63757143	0.944	0.878	1.19E-33	1	S100a6
D8Ertd738e	6.84E-38	0.33052349	0.956	0.894	1.27E-33	1	D8Ertd738e
Tmsb10	1.73E-37	0.29667609	0.999	0.993	3.21E-33	1	Tmsb10
Crtc3	1.55E-36	0.26905363	0.43	0.232	2.87E-32	1	Crtc3
Bin2	1.85E-36	0.33441125	0.704	0.492	3.43E-32	1	Bin2
Pde4b	1.13E-35	0.32833355	0.757	0.544	2.09E-31	1	Pde4b
Tmem256	1.61E-35	0.33987283	0.913	0.822	2.99E-31	1	Tmem256
Tmem50b	1.94E-35	0.36098821	0.709	0.521	3.61E-31	1	Tmem50b
Ywhah	2.79E-35	0.34867702	0.94	0.883	5.18E-31	1	Ywhah
Rpl6	2.85E-35	0.20143888	0.998	0.993	5.29E-31	1	Rpl6
Eif3i	3.00E-35	0.31323467	0.912	0.836	5.57E-31	1	Eif3i
Tyrobp	4.81E-35	0.2762921	0.993	0.975	8.93E-31	1	Tyrobp

Idh2	4.92E-35	0.22431807	0.411	0.216	9.14E-31	1	Idh2
Ypel3	5.59E-35	0.27056782	0.463	0.266	1.04E-30	1	Ypel3
Dapk1	6.10E-35	0.234892	0.338	0.163	1.13E-30	1	Dapk1
Rps3	1.01E-34	0.20644083	0.999	0.992	1.88E-30	1	Rps3
Mrps14	1.52E-34	0.30437329	0.88	0.768	2.83E-30	1	Mrps14
Tkt	2.48E-34	0.32483686	0.899	0.797	4.61E-30	1	Tkt
Mif	2.59E-34	0.37949664	0.861	0.738	4.82E-30	1	Mif
Psmb5	5.72E-34	0.30117445	0.94	0.851	1.06E-29	1	Psmb5
Atp5d	1.39E-33	0.28317661	0.968	0.922	2.58E-29	1	Atp5d
Sec61b	1.44E-33	0.26347961	0.971	0.946	2.67E-29	1	Sec61b
Snrpf	1.64E-33	0.29597675	0.966	0.913	3.04E-29	1	Snrpf
Rpl10	1.85E-33	0.2256179	0.995	0.984	3.43E-29	1	Rpl10
Cox5b	4.82E-33	0.28285722	0.968	0.925	8.96E-29	1	Cox5b
Psma2	6.87E-33	0.27448036	0.935	0.88	1.28E-28	1	Psma2
Wdr83os	7.53E-33	0.31143737	0.737	0.56	1.40E-28	1	Wdr83os
Rps17	1.71E-32	0.26422944	0.987	0.968	3.18E-28	1	Rps17
Arpc3	2.46E-32	0.22744343	0.984	0.973	4.57E-28	1	Arpc3
Mdh2	2.68E-32	0.31965537	0.844	0.715	4.98E-28	1	Mdh2
Cycs	3.37E-32	0.31941145	0.959	0.899	6.27E-28	1	Cycs
Rpl35	3.64E-32	0.21031641	0.993	0.983	6.77E-28	1	Rpl35
Rpl3	6.43E-32	0.20409016	0.998	0.988	1.19E-27	1	Rpl3
Rps4x	8.17E-32	0.2012153	1	0.994	1.52E-27	1	Rps4x
Psma1	9.96E-32	0.3078104	0.921	0.813	1.85E-27	1	Psma1

Rexo2	1.09E-31	0.28466274	0.727	0.561	2.03E-27	1	Rexo2
Rbis	1.25E-31	0.29661623	0.833	0.704	2.33E-27	1	Rbis
Aldh2	1.50E-31	0.27949986	0.515	0.327	2.79E-27	1	Aldh2
Atp5e	2.68E-31	0.2282891	0.993	0.963	4.97E-27	1	Atp5e
Rpl9	2.72E-31	0.20693969	0.999	0.992	5.06E-27	1	Rpl9
Ndufa13	3.23E-31	0.27475203	0.976	0.929	5.99E-27	1	Ndufa13
Vps37b1	3.34E-31	0.42688131	0.754	0.59	6.20E-27	1	Vps37b
Ndufc2	4.23E-31	0.29713121	0.898	0.803	7.85E-27	1	Ndufc2
Gabarapl2	4.46E-31	0.29662763	0.865	0.748	8.29E-27	1	Gabarapl2
Erp29	9.56E-31	0.25290012	0.977	0.949	1.78E-26	1	Erp29
Uqcr11	1.10E-30	0.28688278	0.938	0.88	2.04E-26	1	Uqcr11
Golm1	1.15E-30	0.20313469	0.257	0.114	2.13E-26	1	Golm1
Ndufab1	1.35E-30	0.3051105	0.844	0.741	2.51E-26	1	Ndufab1
Psmb2	1.51E-30	0.29765862	0.934	0.829	2.80E-26	1	Psmb2
Runx3	2.28E-30	0.32859233	0.776	0.629	4.24E-26	1	Runx3
Gpx4	4.50E-30	0.29890824	0.851	0.727	8.37E-26	1	Gpx4
Magohb	5.69E-30	0.29537924	0.546	0.364	1.06E-25	1	Magohb
Hmgb1	6.03E-30	0.29792604	0.948	0.879	1.12E-25	1	Hmgb1
Retreg1	6.33E-30	0.25716196	0.55	0.361	1.18E-25	1	Retreg1
Rbx1	1.66E-29	0.29594894	0.945	0.898	3.09E-25	1	Rbx1
Rps18	3.64E-29	0.21374805	0.999	0.99	6.77E-25	1	Rps18
Atp5j2	3.96E-29	0.26816742	0.97	0.936	7.36E-25	1	Atp5j2
Pebp1	4.04E-29	0.28025333	0.872	0.747	7.50E-25	1	Pebp1

Atp5j	6.42E-29	0.26532839	0.951	0.901	1.19E-24	1	Atp5j
Dpm3	6.84E-29	0.2902166	0.765	0.599	1.27E-24	1	Dpm3
Higd1a	8.70E-29	0.29437952	0.852	0.725	1.62E-24	1	Higd1a
Lgals1	3.69E-28	0.35223793	0.948	0.875	6.85E-24	1	Lgals1
Atp5f1	4.43E-28	0.23159316	0.979	0.948	8.22E-24	1	Atp5f1
Gng5	4.51E-28	0.24348823	0.966	0.932	8.38E-24	1	Gng5
Clic1	5.15E-28	0.22119401	0.99	0.972	9.56E-24	1	Clic1
Gapdh	9.57E-28	0.3008751	0.971	0.946	1.78E-23	1	Gapdh
Ndufb9	1.47E-27	0.27201905	0.923	0.853	2.74E-23	1	Ndufb9
Cox6b1	1.63E-27	0.25328594	0.966	0.931	3.04E-23	1	Cox6b1
Prdx2	4.64E-27	0.25032054	0.937	0.882	8.63E-23	1	Prdx2
Atp5k	4.68E-27	0.27053957	0.96	0.916	8.70E-23	1	Atp5k
Jtb	5.29E-27	0.3187364	0.816	0.699	9.83E-23	1	Jtb
Cox7b	6.53E-27	0.26873347	0.962	0.924	1.21E-22	1	Cox7b
Ndufa7	6.98E-27	0.25972273	0.949	0.888	1.30E-22	1	Ndufa7
Tomm7	1.01E-26	0.22756595	0.974	0.946	1.87E-22	1	Tomm7
Itm2b	1.24E-26	0.21476654	0.988	0.972	2.31E-22	1	Itm2b
Eif5a	1.59E-26	0.25225876	0.987	0.962	2.95E-22	1	Eif5a
Snrpa1	3.11E-26	0.2920188	0.727	0.571	5.77E-22	1	Snrpa1
Nfe2l2	3.19E-26	0.33198066	0.844	0.695	5.93E-22	1	Nfe2l2
Rpl31	5.95E-26	0.2447375	0.985	0.964	1.11E-21	1	Rpl31
Smad7	8.18E-26	0.26443093	0.543	0.371	1.52E-21	1	Smad7
H3f3a	1.11E-25	0.20019671	0.995	0.986	2.06E-21	1	H3f3a

Mrpl34	1.49E-25	0.27539327	0.661	0.501	2.76E-21	1	Mrpl34
H2-DMb1	1.97E-25	0.24038631	0.996	0.964	3.66E-21	1	H2-DMb1
Mpc2	2.49E-25	0.26544204	0.889	0.801	4.63E-21	1	Mpc2
Fam49b	2.50E-25	0.24500795	0.963	0.935	4.65E-21	1	Fam49b
Ndufb8	2.59E-25	0.25045594	0.928	0.853	4.80E-21	1	Ndufb8
Mrpl57	3.14E-25	0.2515429	0.801	0.653	5.83E-21	1	Mrpl57
Rpl29	3.27E-25	0.22474855	0.99	0.969	6.07E-21	1	Rpl29
Dad1	3.63E-25	0.25866024	0.951	0.889	6.75E-21	1	Dad1
Ndufs5	4.03E-25	0.2577047	0.88	0.776	7.48E-21	1	Ndufs5
Snx20	4.73E-25	0.2807987	0.909	0.818	8.78E-21	1	Snx20
Pdap1	5.06E-25	0.31157404	0.846	0.751	9.40E-21	1	Pdap1
Ndufc1	5.35E-25	0.29054133	0.885	0.78	9.94E-21	1	Ndufc1
Nedd8	7.09E-25	0.22272185	0.963	0.937	1.32E-20	1	Nedd8
Rheb1	9.80E-25	0.28663486	0.893	0.811	1.82E-20	1	Rheb
Swi5	1.22E-24	0.26137016	0.867	0.758	2.27E-20	1	Swi5
Psmb3	1.42E-24	0.26055034	0.938	0.877	2.64E-20	1	Psmb3
Psmb6	1.63E-24	0.26426626	0.912	0.839	3.02E-20	1	Psmb6
Ndufb3	2.06E-24	0.27559999	0.843	0.714	3.83E-20	1	Ndufb3
Ndufb11	2.11E-24	0.25075086	0.937	0.879	3.92E-20	1	Ndufb11
Rpl4	2.31E-24	0.20417511	0.991	0.974	4.30E-20	1	Rpl4
Ly6e	4.92E-24	0.29574541	0.888	0.775	9.14E-20	1	Ly6e
Cxcl16	8.44E-24	0.29935679	0.705	0.573	1.57E-19	1	Cxcl16
Nme1	9.89E-24	0.31356422	0.846	0.744	1.84E-19	1	Nme1

Snrpd1	1.20E-23	0.25875528	0.871	0.776	2.24E-19	1	Snrpd1
Micos13	2.23E-23	0.25137184	0.862	0.775	4.15E-19	1	Micos13
Tm2d2	2.58E-23	0.27895676	0.711	0.584	4.79E-19	1	Tm2d2
Ranbp1	7.35E-23	0.28405089	0.893	0.802	1.37E-18	1	Ranbp1
Flot1	8.06E-23	0.27750086	0.768	0.623	1.50E-18	1	Flot1
Lamtor4	1.19E-22	0.27196812	0.677	0.515	2.21E-18	1	Lamtor4
Polr2e	1.21E-22	0.27239832	0.788	0.662	2.24E-18	1	Polr2e
Tmem147	1.65E-22	0.22853491	0.583	0.426	3.06E-18	1	Tmem147
Romo1	1.72E-22	0.23778745	0.873	0.764	3.19E-18	1	Romo1
Pkm	2.22E-22	0.29966132	0.901	0.829	4.13E-18	1	Pkm
Trmt112	2.35E-22	0.27079241	0.795	0.669	4.37E-18	1	Trmt112
Csf2rb	2.41E-22	0.24006545	0.817	0.684	4.47E-18	1	Csf2rb
Set	2.52E-22	0.24870342	0.957	0.93	4.68E-18	1	Set
Ifngr2	2.96E-22	0.27533889	0.695	0.559	5.51E-18	1	Ifngr2
Brk1	3.45E-22	0.22357703	0.941	0.887	6.41E-18	1	Brk1
U2af1	4.96E-22	0.24454511	0.895	0.798	9.21E-18	1	U2af1
Fam107b	8.11E-22	0.27461009	0.48	0.32	1.51E-17	1	Fam107b
Tmem14c	8.89E-22	0.25490804	0.898	0.828	1.65E-17	1	Tmem14c
Ssh2	9.56E-22	0.20889835	0.466	0.304	1.78E-17	1	Ssh2
Ndufs8	1.18E-21	0.25261865	0.816	0.702	2.19E-17	1	Ndufs8
Cisd1	1.21E-21	0.2745527	0.52	0.373	2.25E-17	1	Cisd1
Rgs10	1.43E-21	0.29703056	0.917	0.864	2.65E-17	1	Rgs10
Lta4h	1.46E-21	0.2193626	0.491	0.335	2.71E-17	1	Lta4h

Vdac2	1.54E-21	0.22947862	0.945	0.899	2.86E-17	1	Vdac2
Ndufa5	1.64E-21	0.25161043	0.756	0.622	3.05E-17	1	Ndufa5
Tmem160	1.94E-21	0.25995304	0.896	0.794	3.61E-17	1	Tmem160
Ldha	1.99E-21	0.21713279	0.988	0.966	3.69E-17	1	Ldha
Nudc	2.82E-21	0.22694767	0.844	0.735	5.24E-17	1	Nudc
Mrps24	3.00E-21	0.26081455	0.827	0.706	5.57E-17	1	Mrps24
Ndufb6	3.12E-21	0.21732491	0.783	0.649	5.79E-17	1	Ndufb6
Sumo2	3.28E-21	0.21042364	0.961	0.896	6.09E-17	1	Sumo2
Ran	4.39E-21	0.26814394	0.935	0.871	8.16E-17	1	Ran
Mrpl20	5.82E-21	0.23196795	0.82	0.709	1.08E-16	1	Mrpl20
Aprt	6.20E-21	0.26065975	0.773	0.653	1.15E-16	1	Aprt
Ahr	6.71E-21	0.25097875	0.863	0.75	1.25E-16	1	Ahr
Emc6	7.80E-21	0.23413764	0.756	0.634	1.45E-16	1	Emc6
Coro1a	1.06E-20	0.20446909	0.998	0.986	1.97E-16	1	Coro1a
Hexb	1.49E-20	0.24324346	0.627	0.482	2.77E-16	1	Hexb
Gtf2h5	1.57E-20	0.22294124	0.809	0.683	2.93E-16	1	Gtf2h5
Psma7	1.76E-20	0.2138844	0.98	0.947	3.27E-16	1	Psma7
Prmt1	2.21E-20	0.24638154	0.765	0.641	4.10E-16	1	Prmt1
Osgep	2.77E-20	0.23249088	0.632	0.484	5.15E-16	1	Osgep
Cdk4	4.54E-20	0.23176244	0.528	0.383	8.44E-16	1	Cdk4
Bax	4.63E-20	0.30125891	0.889	0.816	8.60E-16	1	Bax
Ndufb4	6.29E-20	0.237195	0.804	0.704	1.17E-15	1	Ndufb4
Cox7a2	6.46E-20	0.21555216	0.96	0.915	1.20E-15	1	Cox7a2

Ndufa11	6.55E-20	0.22593387	0.821	0.716	1.22E-15	1	Ndufa11
Hsbp1	6.92E-20	0.2492844	0.746	0.621	1.29E-15	1	Hsbp1
Pdcd5	7.06E-20	0.23005938	0.784	0.684	1.31E-15	1	Pdcd5
Znrd1	8.29E-20	0.24716756	0.807	0.707	1.54E-15	1	Znrd1
Mrps21	1.03E-19	0.23602886	0.844	0.748	1.91E-15	1	Mrps21
Gm34084	1.39E-19	0.21263163	0.215	0.105	2.59E-15	1	Gm34084
Ppib	1.45E-19	0.20236981	0.977	0.951	2.70E-15	1	Ppib
Dnajb6	1.90E-19	0.26213901	0.937	0.894	3.54E-15	1	Dnajb6
Tomm20	2.03E-19	0.23703729	0.939	0.896	3.77E-15	1	Tomm20
Hist1h1e	2.03E-19	0.30561822	0.398	0.253	3.77E-15	1	Hist1h1e
Atp5c1	2.12E-19	0.20403031	0.966	0.927	3.94E-15	1	Atp5c1
Tpst2	2.47E-19	0.2153416	0.523	0.373	4.59E-15	1	Tpst2
Calm2	2.61E-19	0.24040776	0.941	0.901	4.84E-15	1	Calm2
Hsd17b10	3.05E-19	0.23227388	0.634	0.504	5.67E-15	1	Hsd17b10
Klrb1b	3.50E-19	0.29386165	0.798	0.682	6.50E-15	1	Klrb1b
Tmem258	4.21E-19	0.23701276	0.926	0.868	7.82E-15	1	Tmem258
Asah1	4.26E-19	0.21165734	0.851	0.759	7.92E-15	1	Asah1
Anp32a	4.56E-19	0.23135703	0.841	0.743	8.47E-15	1	Anp32a
Uqcr10	4.90E-19	0.21800828	0.937	0.876	9.11E-15	1	Uqcr10
H3f3b	5.12E-19	0.20402549	0.996	0.995	9.51E-15	1	H3f3b
Ndufa2	5.21E-19	0.20614199	0.951	0.907	9.68E-15	1	Ndufa2
Prdx1	5.21E-19	0.20991307	0.955	0.914	9.69E-15	1	Prdx1
Snrpd3	5.71E-19	0.24798954	0.856	0.777	1.06E-14	1	Snrpd3

Eef1g	6.57E-19	0.21742629	0.963	0.934	1.22E-14	1	Eef1g
Ndufv2	6.69E-19	0.207542	0.818	0.696	1.24E-14	1	Ndufv2
Hint1	8.95E-19	0.2020233	0.976	0.93	1.66E-14	1	Hint1
Emb	9.16E-19	0.20532828	0.443	0.296	1.70E-14	1	Emb
Timm23	1.07E-18	0.20188894	0.83	0.718	1.99E-14	1	Timm23
Ndufv3	1.42E-18	0.23371552	0.81	0.714	2.64E-14	1	Ndufv3
Ndufb10	1.83E-18	0.22435938	0.888	0.827	3.39E-14	1	Ndufb10
Gsn	1.94E-18	0.27491643	0.829	0.78	3.61E-14	1	Gsn
Lsm7	2.22E-18	0.2201143	0.611	0.471	4.12E-14	1	Lsm7
Ndufa1	2.40E-18	0.21767374	0.922	0.871	4.47E-14	1	Ndufa1
Mrpl23	2.45E-18	0.21434821	0.67	0.531	4.56E-14	1	Mrpl23
Gna15	3.95E-18	0.22903729	0.624	0.479	7.34E-14	1	Gna15
Nudcd2	5.53E-18	0.23225068	0.495	0.347	1.03E-13	1	Nudcd2
Thoc7	5.88E-18	0.21245273	0.712	0.58	1.09E-13	1	Thoc7
Esd	5.99E-18	0.22830812	0.888	0.802	1.11E-13	1	Esd
Tomm6	9.30E-18	0.22123547	0.918	0.864	1.73E-13	1	Tomm6
Etfb	1.06E-17	0.22845066	0.791	0.694	1.97E-13	1	Etfb
Ndufb2	1.13E-17	0.22105492	0.846	0.765	2.09E-13	1	Ndufb2
Chchd1	1.33E-17	0.24641427	0.776	0.681	2.47E-13	1	Chchd1
Lsm6	1.36E-17	0.2408762	0.841	0.744	2.54E-13	1	Lsm6
Timm8b	1.44E-17	0.22383341	0.68	0.564	2.67E-13	1	Timm8b
Atp5mpl	1.72E-17	0.2051131	0.945	0.901	3.20E-13	1	Atp5mpl
Rnf7	2.63E-17	0.20674579	0.722	0.585	4.88E-13	1	Rnf7

Slc16a6	2.83E-17	0.22569404	0.461	0.326	5.26E-13	1	Slc16a6
Cenpx	3.96E-17	0.20469659	0.735	0.594	7.36E-13	1	Cenpx
Hist1h1c	4.31E-17	0.31816722	0.459	0.319	8.01E-13	1	Hist1h1c
Creg1	4.98E-17	0.22856164	0.749	0.637	9.25E-13	1	Creg1
Psma5	9.03E-17	0.21485495	0.883	0.821	1.68E-12	1	Psma5
Sarnp	1.29E-16	0.21934801	0.884	0.798	2.40E-12	1	Sarnp
Sptssa	1.32E-16	0.20071812	0.7	0.561	2.46E-12	1	Sptssa
Vdac1	2.10E-16	0.226785	0.672	0.551	3.90E-12	1	Vdac1
Pgls	2.20E-16	0.21214408	0.922	0.881	4.08E-12	1	Pgls
Ndufa12	2.66E-16	0.22185917	0.766	0.661	4.95E-12	1	Ndufa12
Ffar4	4.98E-16	0.21895844	0.418	0.29	9.24E-12	1	Ffar4
Mrps15	6.30E-16	0.21576249	0.722	0.603	1.17E-11	1	Mrps15
Ncoa71	7.77E-16	0.23622097	0.884	0.795	1.44E-11	1	Ncoa7
Ndufs6	8.07E-16	0.20591458	0.837	0.753	1.50E-11	1	Ndufs6
Selenof	9.85E-16	0.20785553	0.895	0.805	1.83E-11	1	Selenof
Ccdc12	1.10E-15	0.21411381	0.96	0.914	2.04E-11	1	Ccdc12
Bola2	1.27E-15	0.21138545	0.838	0.763	2.36E-11	1	Bola2
Tnfaip2	1.64E-15	0.2837258	0.324	0.204	3.05E-11	1	Tnfaip2
S100a4	1.71E-15	0.42134026	0.548	0.43	3.18E-11	1	S100a4
Hist3h2a	2.03E-15	0.27017884	0.491	0.367	3.77E-11	1	Hist3h2a
Sla	2.67E-15	0.24389715	0.788	0.69	4.97E-11	1	Sla
Mrpl33	3.21E-15	0.21225547	0.893	0.832	5.96E-11	1	Mrpl33
Arhgdib	3.84E-15	0.21362388	0.966	0.936	7.13E-11	1	Arhgdib

Bex3	4.84E-15	0.24391975	0.579	0.453	9.00E-11	1	Bex3
Cuedc2	9.36E-15	0.20222081	0.582	0.462	1.74E-10	1	Cuedc2
Pa2g4	2.68E-14	0.20194233	0.712	0.596	4.97E-10	1	Pa2g4
Eno1	3.17E-14	0.21999755	0.651	0.54	5.90E-10	1	Eno1
Aldoa	3.36E-14	0.27321793	0.911	0.862	6.25E-10	1	Aldoa
Krtcap2	4.57E-14	0.20452963	0.887	0.792	8.50E-10	1	Krtcap2
Sod2	4.75E-14	0.20745178	0.724	0.616	8.83E-10	1	Sod2
Fkbp3	2.48E-13	0.20421446	0.657	0.541	4.60E-09	1	Fkbp3
Dctpp1	2.71E-13	0.22419224	0.749	0.644	5.03E-09	1	Dctpp1
Pgam1	4.44E-13	0.21245054	0.915	0.864	8.25E-09	1	Pgam1
Nhp2	6.96E-13	0.21337593	0.744	0.66	1.29E-08	1	Nhp2
Sf3b5	8.95E-13	0.2049249	0.816	0.73	1.66E-08	1	Sf3b5
Gcnt2	2.04E-12	0.22393146	0.833	0.72	3.78E-08	1	Gcnt2
Pet100	2.96E-12	0.20003274	0.841	0.793	5.50E-08	1	Pet100
Havcr21	1.23E-11	0.20207538	0.774	0.683	2.28E-07	1	Havcr2
Ifrd1	4.69E-09	0.22340058	0.826	0.773	8.71E-05	1	Ifrd1
Pts	5.39E-09	0.23095609	0.579	0.489	0.00010008	1	Pts
Arl4c	4.70E-08	0.20400497	0.634	0.557	0.00087343	1	Arl4c
Ccl2	2.90E-171	1.77167169	0.641	0.213	5.38E-167	2	Ccl2
Ccl7	9.07E-139	1.85168629	0.33	0.058	1.68E-134	2	Ccl7
Naaa	5.36E-100	0.49499728	1	0.951	9.95E-96	2	Naaa
Gm2a1	5.94E-89	0.54727084	0.999	0.964	1.10E-84	2	Gm2a
Ier3	3.96E-84	0.94863956	0.656	0.346	7.35E-80	2	Ier3

Naga1	1.06E-83	0.50676658	0.984	0.911	1.97E-79	2	Naga
Egr2	2.62E-75	0.63126805	0.67	0.359	4.87E-71	2	Egr2
Rnase61	5.30E-70	0.49721984	0.968	0.852	9.84E-66	2	Rnase6
Klf2	6.37E-68	0.67258873	0.925	0.723	1.18E-63	2	Klf2
Sep-31	1.07E-67	0.53215122	0.939	0.706	2.00E-63	2	Sep-03
Cxcr3	3.35E-67	0.45842637	0.732	0.438	6.22E-63	2	Cxcr3
BC0285281	6.61E-67	0.47522096	0.973	0.865	1.23E-62	2	BC028528
Tubb2a	1.84E-66	0.48282901	0.815	0.532	3.42E-62	2	Tubb2a
Dusp1	4.95E-66	0.57399575	0.955	0.874	9.20E-62	2	Dusp1
Adrb2	1.91E-65	0.56452883	0.596	0.314	3.55E-61	2	Adrb2
Irf8	1.42E-64	0.39557833	0.998	0.975	2.64E-60	2	Irf8
Gadd45b	3.18E-61	0.60860722	0.714	0.452	5.90E-57	2	Gadd45b
Cd180	5.13E-61	0.50505415	0.75	0.488	9.54E-57	2	Cd180
Hfe1	1.40E-60	0.52953284	0.865	0.632	2.60E-56	2	Hfe
Fos	2.16E-60	0.58570359	0.989	0.975	4.01E-56	2	Fos
Fcrla	8.57E-60	0.53969198	0.605	0.331	1.59E-55	2	Fcrla
H2-Eb11	1.16E-59	0.27477831	1	0.999	2.15E-55	2	H2-Eb1
Unc1191	2.58E-59	0.46711328	0.958	0.831	4.79E-55	2	Unc119
Nfkbid	5.13E-58	0.36868981	0.665	0.386	9.53E-54	2	Nfkbid
Atox11	2.63E-56	0.3314881	0.999	0.968	4.89E-52	2	Atox1
Dyrk3	8.10E-53	0.21496941	0.244	0.073	1.50E-48	2	Dyrk3
Xcr1	2.84E-52	0.45302266	0.911	0.708	5.27E-48	2	Xcr1
Cd24a	1.52E-51	0.36374109	0.987	0.937	2.82E-47	2	Cd24a

Ucp21	3.86E-51	0.33364308	0.995	0.963	7.18E-47	2	Ucp2
Ccl3	6.04E-51	0.79783837	0.308	0.116	1.12E-46	2	Ccl3
Cd300c21	2.32E-50	0.42503703	0.827	0.621	4.30E-46	2	Cd300c2
Ppt11	1.88E-49	0.38100911	0.991	0.928	3.50E-45	2	Ppt1
Cbfa2t3	2.56E-49	0.40305831	0.966	0.892	4.75E-45	2	Cbfa2t3
Pianp1	3.11E-49	0.33345237	0.642	0.368	5.78E-45	2	Pianp
Gcsam	3.58E-49	0.41160103	0.8	0.572	6.65E-45	2	Gcsam
H2-Aa1	4.04E-49	0.2096385	1	0.999	7.51E-45	2	H2-Aa
Ndufa61	2.68E-47	0.34621347	0.979	0.936	4.97E-43	2	Ndufa6
Man2b1	9.41E-45	0.31913948	0.977	0.914	1.75E-40	2	Man2b1
Gm3336	3.98E-42	0.31798516	0.469	0.25	7.40E-38	2	Gm3336
Jaml	8.57E-42	0.36018508	0.977	0.89	1.59E-37	2	Jaml
Mar-01	7.58E-41	0.36097835	0.893	0.738	1.41E-36	2	Mar-01
Fgfr11	4.18E-40	0.32055025	0.74	0.468	7.77E-36	2	Fgfr1
Cadm1	5.68E-40	0.33643184	0.672	0.415	1.06E-35	2	Cadm1
Lyz21	2.30E-39	0.36701155	0.984	0.902	4.28E-35	2	Lyz2
Malat1	7.39E-38	0.22061296	0.999	1	1.37E-33	2	Malat1
Psmb91	1.48E-37	0.28546962	0.996	0.958	2.75E-33	2	Psmb9
Hspa1a	3.31E-37	0.29547163	0.865	0.604	6.14E-33	2	Hspa1a
Crip1	7.46E-37	0.31156178	1	0.995	1.39E-32	2	Crip1
Adgre5	1.92E-36	0.32659764	0.887	0.706	3.58E-32	2	Adgre5
H2-DMa	5.18E-36	0.2506306	0.998	0.969	9.62E-32	2	H2-DMa
Olfm1	1.29E-35	0.36423389	0.935	0.831	2.40E-31	2	Olfm1

Pglyrp1	2.81E-35	0.35513675	0.506	0.299	5.22E-31	2	Pglyrp1
Id2	3.18E-35	0.36654901	0.968	0.89	5.90E-31	2	Id2
Atp6v0e1	5.26E-35	0.26097574	0.99	0.951	9.77E-31	2	Atp6v0e
2810013P06Rik	6.23E-35	0.24849833	0.462	0.258	1.16E-30	2	2810013P06Rik
Cst31	6.31E-35	0.22349476	1	1	1.17E-30	2	Cst3
Fcgr2b1	1.01E-34	0.38682141	0.811	0.653	1.87E-30	2	Fcgr2b
Tnfaip8l2	1.29E-34	0.30030947	0.67	0.453	2.40E-30	2	Tnfaip8l2
H2-Ob1	2.13E-34	0.31314344	0.901	0.733	3.97E-30	2	H2-Ob
Snx22	3.47E-34	0.31606297	0.7	0.504	6.44E-30	2	Snx22
Ptpn181	5.19E-34	0.28182276	0.996	0.943	9.64E-30	2	Ptpn18
Rilpl2	5.85E-32	0.34834489	0.771	0.571	1.09E-27	2	Rilpl2
Eef1b21	4.53E-31	0.23806245	0.998	0.985	8.41E-27	2	Eef1b2
Tnf	6.19E-31	0.20793958	0.23	0.093	1.15E-26	2	Tnf
Phf11a	1.06E-30	0.31971602	0.767	0.568	1.97E-26	2	Phf11a
Klf6	6.81E-30	0.24366549	0.979	0.919	1.27E-25	2	Klf6
Cd521	8.59E-30	0.2702895	0.999	0.993	1.60E-25	2	Cd52
Zfp36l1	2.27E-29	0.35355827	0.844	0.697	4.21E-25	2	Zfp36l1
Ckb1	2.59E-29	0.28499987	0.999	0.961	4.81E-25	2	Ckb
Atpif11	5.15E-29	0.25976856	0.994	0.959	9.57E-25	2	Atpif1
9930111J21Rik2	6.49E-29	0.26686886	0.604	0.407	1.21E-24	2	9930111J21Rik2
Rab32	1.87E-28	0.28250231	0.849	0.679	3.47E-24	2	Rab32
Pfkp	3.29E-28	0.26256767	0.951	0.877	6.12E-24	2	Pfkp
Lemd2	4.47E-28	0.28434113	0.716	0.524	8.31E-24	2	Lemd2

Grap	9.28E-28	0.21445542	0.414	0.234	1.72E-23	2	Grap
Tlr3	1.61E-27	0.34184445	0.842	0.697	2.99E-23	2	Tlr3
Cass4	5.10E-27	0.26152843	0.501	0.316	9.47E-23	2	Cass4
Atf7ip	1.04E-26	0.26163353	0.506	0.323	1.93E-22	2	Atf7ip
Timm10b	1.85E-26	0.27459922	0.96	0.9	3.43E-22	2	Timm10b
Slc31a2	3.13E-26	0.24898878	0.602	0.409	5.82E-22	2	Slc31a2
Alox5ap	4.04E-26	0.23158133	0.993	0.952	7.50E-22	2	Alox5ap
Scimp	6.11E-26	0.25105934	0.75	0.561	1.14E-21	2	Scimp
Gpsm3	9.10E-26	0.29176252	0.929	0.852	1.69E-21	2	Gpsm3
Ccl4	9.67E-26	0.60602176	0.768	0.627	1.80E-21	2	Ccl4
Cited2	1.49E-25	0.30411378	0.632	0.435	2.76E-21	2	Cited2
Ptms	1.62E-25	0.23639878	0.991	0.963	3.01E-21	2	Ptms
Zbtb46	4.05E-25	0.21749785	0.599	0.413	7.53E-21	2	Zbtb46
Il181	6.17E-25	0.26825407	0.676	0.483	1.15E-20	2	Il18
Btla	7.04E-25	0.23246228	0.848	0.685	1.31E-20	2	Btla
Itgae	1.79E-24	0.25813782	0.799	0.593	3.33E-20	2	Itgae
Txndc15	5.96E-24	0.24457935	0.898	0.773	1.11E-19	2	Txndc15
Acox3	5.57E-23	0.23397446	0.718	0.549	1.03E-18	2	Acox3
Eid1	1.05E-22	0.2594162	0.871	0.761	1.95E-18	2	Eid1
Rnd3	1.52E-22	0.24254069	0.525	0.341	2.83E-18	2	Rnd3
Slamf7	1.66E-22	0.2505341	0.944	0.841	3.08E-18	2	Slamf7
Napsa	2.30E-22	0.23190429	0.99	0.959	4.27E-18	2	Napsa
Npc2	2.89E-22	0.21384634	0.994	0.98	5.38E-18	2	Npc2

Ier5	1.17E-21	0.25949196	0.983	0.967	2.18E-17	2	Ier5
Rsrp1	1.44E-21	0.23710127	0.911	0.813	2.68E-17	2	Rsrp1
Pik3cb	3.30E-21	0.21027128	0.809	0.631	6.12E-17	2	Pik3cb
Metrnl	3.32E-21	0.23048312	0.635	0.462	6.16E-17	2	Metrnl
Anpep	5.31E-21	0.23243315	0.763	0.604	9.87E-17	2	Anpep
Arsb	9.25E-21	0.22451667	0.771	0.595	1.72E-16	2	Arsb
Shtn1	4.12E-20	0.21478648	0.963	0.871	7.66E-16	2	Shtn1
Sat1	6.67E-20	0.31974677	0.881	0.81	1.24E-15	2	Sat1
Ciita	1.85E-19	0.23195262	0.877	0.767	3.44E-15	2	Ciita
Phf11b	2.28E-19	0.20366813	0.886	0.757	4.24E-15	2	Phf11b
Agpat3	5.34E-19	0.20033923	0.679	0.512	9.93E-15	2	Agpat3
Rtl8a1	6.41E-19	0.27385228	0.6	0.449	1.19E-14	2	Rtl8a
Ubb	1.34E-18	0.20035355	0.996	0.985	2.50E-14	2	Ubb
Fgd2	1.38E-18	0.2191166	0.918	0.824	2.57E-14	2	Fgd2
St3gal51	1.40E-18	0.23104502	0.857	0.723	2.60E-14	2	St3gal5
Polr1d	2.19E-18	0.20709634	0.96	0.907	4.07E-14	2	Polr1d
Gltp	3.93E-18	0.20137405	0.92	0.838	7.30E-14	2	Gltp
Sirt21	7.37E-18	0.24073449	0.651	0.514	1.37E-13	2	Sirt2
Batf3	1.01E-17	0.20079183	0.952	0.883	1.88E-13	2	Batf3
Vamp8	1.29E-17	0.20208812	0.958	0.892	2.39E-13	2	Vamp8
Rnf19b	1.56E-17	0.28658832	0.661	0.525	2.90E-13	2	Rnf19b
Tubb5	1.60E-17	0.20427816	0.868	0.769	2.98E-13	2	Tubb5
Wfdc17	1.65E-17	0.30594187	0.79	0.658	3.06E-13	2	Wfdc17

Cd48	6.77E-17	0.22589576	0.933	0.86	1.26E-12	2	Cd48
Dnajc8	1.12E-16	0.20026001	0.933	0.878	2.08E-12	2	Dnajc8
Rtl8b1	3.61E-16	0.25437488	0.55	0.407	6.70E-12	2	Rtl8b
Rinl	1.00E-15	0.20261032	0.475	0.341	1.86E-11	2	Rinl
Plp2	1.81E-15	0.2166037	0.772	0.658	3.37E-11	2	Plp2
Myd88	1.54E-14	0.20807517	0.786	0.658	2.85E-10	2	Myd88
Grcc10	2.42E-14	0.2000166	0.936	0.837	4.49E-10	2	Grcc10
2410006H16Rik	5.03E-11	0.20797722	0.947	0.898	9.35E-07	2	2410006H16Rik
Furin	3.42E-09	0.21228324	0.621	0.521	6.35E-05	2	Furin
Gm6377	6.07E-07	0.200581	0.817	0.762	0.01127756	2	Gm6377
Klf4	8.58E-07	0.20413754	0.779	0.693	0.01594603	2	Klf4
Hspa1a1	1.61E-197	1.68967788	0.975	0.596	2.99E-193	3	Hspa1a
Hspa1b	5.97E-176	1.41496114	0.924	0.473	1.11E-171	3	Hspa1b
Fos1	4.22E-171	1.15154637	1	0.973	7.83E-167	3	Fos
Egr1	2.12E-167	1.24120917	0.994	0.761	3.95E-163	3	Egr1
Jun	7.12E-158	1.36848262	0.975	0.802	1.32E-153	3	Jun
Hsp90aa1	6.58E-147	0.8854452	1	0.972	1.22E-142	3	Hsp90aa1
Ubc	5.41E-136	0.68720719	0.999	0.991	1.01E-131	3	Ubc
Dnajb1	1.85E-128	0.94121579	0.918	0.6	3.44E-124	3	Dnajb1
Hsp90ab1	2.52E-124	0.60625035	1	0.996	4.68E-120	3	Hsp90ab1
Ier2	7.55E-118	0.82430361	0.997	0.927	1.40E-113	3	Ier2
Dnaja1	1.50E-117	0.83111443	1	0.964	2.78E-113	3	Dnaja1
Junb	2.15E-116	0.71159036	1	0.989	3.99E-112	3	Junb

Klf21	4.63E-111	0.99785 53	0.981	0.721	8.59E-107	3	Klf2
Ier51	2.24E-105	0.65412 851	1	0.965	4.15E-101	3	Ier5
Dusp11	2.78E-101	0.90790 82	0.994	0.871	5.17E-97	3	Dusp1
Pmaip1	1.94E-99	0.77355 628	0.98	0.844	3.61E-95	3	Pmaip1
Hspa8	4.59E-98	0.56708 182	1	0.996	8.54E-94	3	Hspa8
Zfp36	2.99E-95	0.68142 267	0.996	0.949	5.56E-91	3	Zfp36
Atf3	2.65E-94	0.97846 024	0.988	0.905	4.92E-90	3	Atf3
Klf61	1.76E-92	0.76888 303	0.997	0.918	3.27E-88	3	Klf6
Fosb	1.09E-86	0.75817 1	0.996	0.905	2.03E-82	3	Fosb
Hsph1	2.56E-80	0.64942 35	0.74	0.405	4.75E-76	3	Hsph1
Snhg1	2.35E-75	0.59158 819	0.962	0.812	4.36E-71	3	Snhg1
Jund	1.35E-74	0.55215 329	1	0.973	2.51E-70	3	Jund
Rsrp11	9.96E-69	0.55194 991	0.966	0.808	1.85E-64	3	Rsrp1
Ubb1	2.43E-67	0.42362 286	1	0.985	4.51E-63	3	Ubb
Neat1	1.08E-57	0.55871 526	0.946	0.749	2.00E-53	3	Neat1
Mcl1	1.15E-57	0.39205 307	0.999	0.98	2.14E-53	3	Mcl1
Tubb2a1	1.03E-56	0.47830 093	0.823	0.539	1.92E-52	3	Tubb2a
Ccnl1	3.14E-52	0.39623 782	0.993	0.945	5.84E-48	3	Ccnl1
Sertad1	1.03E-51	0.41497 295	0.594	0.324	1.92E-47	3	Sertad1
Ppp1r15a	8.53E-51	0.38557 229	0.958	0.8	1.59E-46	3	Ppp1r15a
Irf81	1.46E-49	0.35012 654	0.999	0.975	2.71E-45	3	Irf8
Wsb1	6.31E-49	0.40413 444	0.855	0.64	1.17E-44	3	Wsb1
Rnd31	1.22E-46	0.53569 794	0.601	0.335	2.26E-42	3	Rnd3

Klf41	4.57E-46	0.61775002	0.892	0.68	8.50E-42	3	Klf4
Clk1	7.61E-46	0.35726466	0.987	0.891	1.41E-41	3	Clk1
Nr4a1	1.81E-45	0.43489023	0.996	0.942	3.36E-41	3	Nr4a1
Brd2	4.65E-45	0.43020687	0.925	0.799	8.64E-41	3	Brd2
Eif4a2	2.21E-42	0.40262777	0.887	0.725	4.11E-38	3	Eif4a2
Ppp1r10	9.90E-42	0.41975221	0.712	0.499	1.84E-37	3	Ppp1r10
Tra2b	2.48E-40	0.38258525	0.982	0.931	4.60E-36	3	Tra2b
Pnrc1	2.03E-38	0.36329937	0.98	0.869	3.78E-34	3	Pnrc1
Ddx5	9.43E-38	0.29355359	1	0.992	1.75E-33	3	Ddx5
Arhgap17	1.50E-37	0.31611576	0.887	0.69	2.78E-33	3	Arhgap17
Fgfr12	2.65E-37	0.30661035	0.749	0.474	4.92E-33	3	Fgfr1
Cebpa	1.91E-36	0.39681011	0.412	0.214	3.55E-32	3	Cebpa
Slc5a3	1.48E-35	0.21882564	0.262	0.099	2.74E-31	3	Slc5a3
Hspe1	9.93E-35	0.43935318	0.975	0.922	1.84E-30	3	Hspe1
Btg21	1.14E-34	0.42241879	1	0.98	2.12E-30	3	Btg2
Dnajb9	1.45E-33	0.43822178	0.75	0.54	2.69E-29	3	Dnajb9
Lmna1	1.76E-32	0.50063402	0.744	0.529	3.27E-28	3	Lmna
2410006H16Rik1	3.73E-32	0.38451588	0.978	0.896	6.94E-28	3	2410006H16Rik
Cd37	6.79E-32	0.31293007	0.797	0.589	1.26E-27	3	Cd37
Pabpc1	1.46E-31	0.26041304	0.991	0.976	2.72E-27	3	Pabpc1
Cited21	5.87E-31	0.37430252	0.67	0.435	1.09E-26	3	Cited2
Eif5	2.34E-30	0.30056012	0.966	0.913	4.34E-26	3	Eif5
Tent5c1	3.20E-30	0.38381364	0.921	0.768	5.95E-26	3	Tent5c

Adrb21	3.66E-30	0.42103367	0.539	0.329	6.80E-26	3	Adrb2
Pcf11	4.58E-30	0.26895031	0.67	0.45	8.52E-26	3	Pcf11
Unc1192	5.69E-30	0.31748503	0.956	0.835	1.06E-25	3	Unc119
Mat2a	6.50E-30	0.33428545	0.86	0.694	1.21E-25	3	Mat2a
Cadm11	7.73E-30	0.29828492	0.661	0.423	1.44E-25	3	Cadm1
Hspd1	1.24E-29	0.39510676	0.877	0.755	2.30E-25	3	Hspd1
Egr21	2.09E-29	0.28572646	0.605	0.376	3.88E-25	3	Egr2
Icam1	2.80E-29	0.23710978	0.731	0.512	5.21E-25	3	Icam1
Cbfa2t31	3.69E-29	0.26741509	0.975	0.892	6.86E-25	3	Cbfa2t3
Olfm11	9.51E-29	0.35572355	0.937	0.834	1.77E-24	3	Olfm1
Midn	1.04E-27	0.32286539	0.664	0.478	1.94E-23	3	Midn
Naga2	1.27E-27	0.26703866	0.99	0.912	2.35E-23	3	Naga
Adgre51	1.37E-27	0.35112604	0.864	0.714	2.54E-23	3	Adgre5
Cxcr31	1.42E-27	0.25829731	0.671	0.453	2.63E-23	3	Cxcr3
Naaa1	3.88E-27	0.23448374	1	0.952	7.21E-23	3	Naaa
Bag3	6.13E-27	0.20539611	0.294	0.136	1.14E-22	3	Bag3
9930111J21Rik21	8.13E-27	0.20079627	0.64	0.408	1.51E-22	3	9930111J21Rik2
Grap1	9.93E-27	0.24013399	0.423	0.238	1.85E-22	3	Grap
Sik1	2.78E-26	0.2459082	0.795	0.616	5.16E-22	3	Sik1
Sde2	3.90E-26	0.27679604	0.74	0.556	7.25E-22	3	Sde2
Sep-32	3.94E-26	0.27372493	0.928	0.713	7.32E-22	3	Sep-03
Oser1	4.33E-26	0.24874886	0.639	0.434	8.04E-22	3	Oser1
Jmjd6	1.93E-25	0.24967657	0.583	0.397	3.59E-21	3	Jmjd6

Rilpl21	4.07E-24	0.37267209	0.768	0.577	7.56E-20	3	Rilpl2
Rhob	5.54E-24	0.23864211	0.304	0.154	1.03E-19	3	Rhob
Ciita1	5.84E-24	0.23876018	0.909	0.765	1.08E-19	3	Ciita
Lemd21	6.37E-24	0.23067976	0.725	0.528	1.18E-19	3	Lemd2
Pianp2	1.14E-23	0.23972987	0.595	0.381	2.13E-19	3	Pianp
Nuak2	1.95E-23	0.23078884	0.757	0.594	3.62E-19	3	Nuak2
Rassf1	2.30E-23	0.23871502	0.807	0.634	4.27E-19	3	Rassf1
Taf1d	2.63E-23	0.26152283	0.861	0.738	4.89E-19	3	Taf1d
Srsf2	2.75E-23	0.26054625	0.962	0.9	5.11E-19	3	Srsf2
Gm63771	3.26E-23	0.30535426	0.895	0.753	6.05E-19	3	Gm6377
Arl13b	6.42E-23	0.23855469	0.409	0.238	1.19E-18	3	Arl13b
Gadd45g	1.56E-22	0.331416	0.523	0.338	2.91E-18	3	Gadd45g
Ahsa1	1.87E-22	0.26197045	0.686	0.532	3.48E-18	3	Ahsa1
Zc3h10	2.72E-22	0.21373041	0.436	0.261	5.06E-18	3	Zc3h10
Rab321	5.34E-22	0.22554443	0.839	0.685	9.93E-18	3	Rab32
Ighm	6.71E-22	0.22827366	0.806	0.601	1.25E-17	3	Ighm
Fgd21	6.94E-22	0.22176758	0.931	0.824	1.29E-17	3	Fgd2
Chordc1	9.76E-22	0.23720548	0.702	0.538	1.81E-17	3	Chordc1
Plekha5	1.22E-21	0.20562392	0.681	0.486	2.27E-17	3	Plekha5
Dynll1	1.42E-21	0.28794233	0.977	0.917	2.63E-17	3	Dynll1
Mt11	1.93E-20	0.42548148	0.873	0.72	3.59E-16	3	Mt1
Irs2	3.88E-20	0.25314485	0.513	0.347	7.21E-16	3	Irs2
Rtl8b2	1.72E-19	0.28930429	0.57	0.408	3.19E-15	3	Rtl8b

Tob2	1.81E-19	0.23041437	0.867	0.735	3.37E-15	3	Tob2
Snx221	3.81E-19	0.23622102	0.694	0.51	7.08E-15	3	Snx22
Tiparp	6.66E-19	0.2786301	0.882	0.758	1.24E-14	3	Tiparp
Ints6	8.57E-19	0.20347514	0.537	0.362	1.59E-14	3	Ints6
Smc4	1.38E-18	0.26027829	0.624	0.46	2.57E-14	3	Smc4
Vim	1.99E-18	0.30197019	0.994	0.981	3.70E-14	3	Vim
1810026B05Rik	2.06E-18	0.25646885	0.661	0.509	3.83E-14	3	1810026B05Rik
Rsrc2	3.42E-18	0.25941997	0.857	0.749	6.35E-14	3	Rsrc2
Tuba4a	4.39E-18	0.22084028	0.532	0.377	8.16E-14	3	Tuba4a
Pi4k2a	4.40E-18	0.20636513	0.759	0.604	8.18E-14	3	Pi4k2a
St3gal52	1.80E-17	0.21911456	0.863	0.726	3.34E-13	3	St3gal5
Tubb6	2.17E-17	0.21333666	0.659	0.487	4.04E-13	3	Tubb6
Snhg12	2.21E-17	0.21411484	0.604	0.443	4.10E-13	3	Snhg12
Tnfaip8l21	4.08E-17	0.20239865	0.623	0.465	7.58E-13	3	Tnfaip8l2
Tsc22d3	4.78E-17	0.26041732	0.702	0.545	8.89E-13	3	Tsc22d3
Rpl22l1	3.00E-16	0.2248383	0.974	0.914	5.58E-12	3	Rpl22l1
Slc20a1	4.64E-16	0.20711676	0.465	0.308	8.62E-12	3	Slc20a1
Ccr2	6.47E-16	0.20941358	0.927	0.789	1.20E-11	3	Ccr2
Zfas1	2.04E-15	0.21502149	0.781	0.642	3.79E-11	3	Zfas1
Spty2d1	4.02E-15	0.22971368	0.844	0.735	7.48E-11	3	Spty2d1
Acox31	6.44E-15	0.20311146	0.699	0.556	1.20E-10	3	Acox3
Baz1a	1.26E-14	0.2256815	0.949	0.899	2.34E-10	3	Baz1a
Eif1a	1.53E-14	0.21714273	0.882	0.778	2.84E-10	3	Eif1a

Zfp36l2	2.33E-13	0.22440538	0.971	0.903	4.32E-09	3	Zfp36l2
Ube2s	2.60E-13	0.21394844	0.898	0.803	4.83E-09	3	Ube2s
Rgs2	1.19E-11	0.29265205	0.974	0.916	2.22E-07	3	Rgs2
Tubb4b	2.38E-11	0.22358598	0.583	0.466	4.43E-07	3	Tubb4b
Snhg15	2.78E-09	0.22264488	0.573	0.46	5.17E-05	3	Snhg15
Txndc16	8.63E-07	0.20316879	0.558	0.455	0.01602638	3	Txndc16
Socs3	5.31E-06	0.24165352	0.472	0.405	0.0987289	3	Socs3
Rgs1	9.66E-06	0.22510161	0.686	0.619	0.1794833	3	Rgs1
Ctsc	5.49E-05	0.25167943	0.823	0.793	1	3	Ctsc
Ifitm61	1.20E-230	1.2603175	0.637	0.111	2.23E-226	4	Ifitm6
Sell	3.69E-195	0.90816175	0.469	0.064	6.85E-191	4	Sell
Serpinb2	3.54E-194	0.48546031	0.236	0.008	6.58E-190	4	Serpinb2
S100a41	2.24E-189	1.98494516	0.883	0.405	4.16E-185	4	S100a4
Cd209a	3.94E-160	1.30438433	0.42	0.065	7.32E-156	4	Cd209a
Rasgrp2	1.59E-157	0.44584303	0.332	0.037	2.96E-153	4	Rasgrp2
Cd7	5.17E-148	0.55936597	0.303	0.032	9.60E-144	4	Cd7
I830077J02Rik	2.08E-134	0.5261043	0.453	0.09	3.87E-130	4	I830077J02Rik
S100a61	3.10E-133	1.35866292	0.98	0.878	5.76E-129	4	S100a6
Ms4a6c	6.59E-128	1.12391036	0.941	0.619	1.22E-123	4	Ms4a6c
Emilin2	9.02E-121	0.59635882	0.428	0.091	1.68E-116	4	Emilin2
Cd300a	3.30E-117	1.04054723	0.814	0.418	6.13E-113	4	Cd300a
Tagln2	1.57E-115	0.96070501	0.992	0.955	2.92E-111	4	Tagln2
S100a10	1.07E-107	1.06325281	0.977	0.869	1.98E-103	4	S100a10

Rpl81	8.08E-106	0.47096095	0.998	0.994	1.50E-101	4	Rpl8
Tmsb101	4.34E-104	0.85612967	0.996	0.993	8.07E-100	4	Tmsb10
Vim1	2.95E-102	0.93795939	1	0.981	5.48E-98	4	Vim
Ldlr	1.31E-93	0.74111669	0.504	0.154	2.43E-89	4	Ldlr
Fdps1	2.11E-90	1.32353134	0.771	0.428	3.92E-86	4	Fdps
Upb1	2.66E-88	0.39955413	0.26	0.043	4.95E-84	4	Upb1
Plac81	1.28E-87	0.97161477	0.953	0.719	2.38E-83	4	Plac8
Plaur	2.75E-86	0.50841787	0.328	0.07	5.11E-82	4	Plaur
H3f3a1	2.48E-77	0.45224691	0.998	0.986	4.60E-73	4	H3f3a
Idi11	3.14E-76	0.95009649	0.537	0.211	5.83E-72	4	Idi1
Fcer1g	2.40E-74	0.73511191	0.98	0.927	4.45E-70	4	Fcer1g
Ms4a4c	5.77E-73	0.64104959	0.559	0.221	1.07E-68	4	Ms4a4c
Fam129b	1.35E-72	0.38519804	0.33	0.083	2.50E-68	4	Fam129b
Bex6	1.20E-70	0.29552345	0.225	0.039	2.24E-66	4	Bex6
Cyp511	1.45E-69	1.00443981	0.664	0.352	2.69E-65	4	Cyp51
Il17ra	1.16E-67	0.43622134	0.396	0.126	2.16E-63	4	Il17ra
Ifi301	1.26E-67	0.67354452	0.986	0.947	2.33E-63	4	Ifi30
Rpl151	1.21E-66	0.39268666	0.988	0.988	2.24E-62	4	Rpl15
Msmo1	1.44E-66	0.87347492	0.508	0.214	2.67E-62	4	Msmo1
Ifitm31	1.71E-66	0.98384291	0.979	0.917	3.18E-62	4	Ifitm3
Rpl211	2.34E-66	0.3903248	0.994	0.986	4.35E-62	4	Rpl21
Gmfg	1.18E-65	0.64798579	0.906	0.754	2.20E-61	4	Gmfg
Rps27a1	2.49E-65	0.32112555	1	0.996	4.63E-61	4	Rps27a

Plec	1.23E-64	0.61953353	0.467	0.177	2.28E-60	4	Plec
Rps81	1.37E-63	0.31065987	1	0.996	2.54E-59	4	Rps8
Ccnd3	2.59E-62	0.64495228	0.822	0.598	4.82E-58	4	Ccnd3
Tyrobp1	1.51E-61	0.51502437	0.984	0.977	2.80E-57	4	Tyrobp
Rpl281	2.35E-61	0.34641211	0.996	0.993	4.36E-57	4	Rpl28
Coro1a1	3.89E-61	0.41133365	0.998	0.986	7.23E-57	4	Coro1a
Itgam	5.61E-61	0.39426129	0.279	0.07	1.04E-56	4	Itgam
H2afj1	4.33E-60	0.60828172	0.896	0.755	8.05E-56	4	H2afj
Arid5a	5.83E-60	0.51028203	0.508	0.216	1.08E-55	4	Arid5a
Ppia1	2.60E-58	0.40479874	0.998	0.987	4.82E-54	4	Ppia
Ifitm11	6.44E-58	1.53510063	0.686	0.42	1.20E-53	4	Ifitm1
Anxa2	3.92E-57	0.61150183	0.988	0.952	7.28E-53	4	Anxa2
Zeb2	6.55E-57	0.3995596	0.287	0.077	1.22E-52	4	Zeb2
Ctsh1	2.75E-55	0.51745507	0.963	0.873	5.11E-51	4	Ctsh
L1cam	4.19E-55	0.32962131	0.281	0.076	7.79E-51	4	L1cam
Rpl131	1.73E-54	0.29994632	1	0.998	3.22E-50	4	Rpl13
Rps3a1	4.44E-54	0.29478542	1	0.995	8.25E-50	4	Rps3a1
Pycard1	1.54E-53	0.61777902	0.877	0.738	2.87E-49	4	Pycard
Igsf6	2.13E-53	0.33049637	0.254	0.065	3.95E-49	4	Igsf6
Rps26	3.14E-52	0.35995181	0.986	0.986	5.83E-48	4	Rps26
Lgals11	3.69E-52	0.76029208	0.949	0.879	6.86E-48	4	Lgals1
Tkt1	7.85E-52	0.53554117	0.906	0.802	1.46E-47	4	Tkt
Trappc5	6.63E-51	0.55483992	0.564	0.311	1.23E-46	4	Trappc5

Bin21	7.65E-51	0.53050039	0.75	0.5	1.42E-46	4	Bin2
Ramp1	1.21E-50	0.63358333	0.672	0.434	2.25E-46	4	Ramp1
Pou2f2	1.40E-50	0.52292115	0.457	0.192	2.60E-46	4	Pou2f2
Cdkn2d	8.59E-50	0.61023396	0.607	0.34	1.60E-45	4	Cdkn2d
Ptma1	8.98E-50	0.38966526	1	0.992	1.67E-45	4	Ptma
Abcb1b	4.27E-49	0.34626148	0.299	0.09	7.93E-45	4	Abcb1b
Rplp01	4.50E-49	0.32319368	0.994	0.993	8.36E-45	4	Rplp0
Hmgcs1	1.07E-48	0.94806733	0.475	0.23	1.98E-44	4	Hmgcs1
Atp2b4	2.91E-48	0.25862644	0.205	0.046	5.41E-44	4	Atp2b4
Rps15a	1.20E-47	0.30936024	1	0.99	2.24E-43	4	Rps15a
Rpl351	2.97E-47	0.32362018	0.998	0.983	5.52E-43	4	Rpl35
Emp3	4.08E-47	0.56238808	0.939	0.851	7.59E-43	4	Emp3
Rpl111	5.16E-47	0.2848482	1	0.995	9.59E-43	4	Rpl11
Sqle	1.94E-46	0.36291371	0.279	0.087	3.61E-42	4	Sqle
Cks2	3.16E-46	0.56119097	0.424	0.182	5.87E-42	4	Cks2
Naca1	4.53E-46	0.37790662	0.994	0.979	8.43E-42	4	Naca
Rps191	6.15E-46	0.29832658	0.996	0.991	1.14E-41	4	Rps19
Ncf2	7.98E-46	0.47142601	0.605	0.353	1.48E-41	4	Ncf2
Rps4x1	3.26E-45	0.28710285	0.996	0.995	6.05E-41	4	Rps4x
Tmem176b	8.62E-45	0.42361364	0.297	0.097	1.60E-40	4	Tmem176b
Rpl32	3.81E-42	0.30751256	0.996	0.995	7.07E-38	4	Rpl32
Rps21	7.47E-42	0.31534657	0.996	0.991	1.39E-37	4	Rps2
Lst1	3.38E-41	0.4672039	0.51	0.271	6.28E-37	4	Lst1

Hmgb11	3.99E-40	0.4399084	0.943	0.884	7.41E-36	4	Hmgb1
Mef2c1	4.28E-40	0.55481653	0.76	0.552	7.95E-36	4	Mef2c
Eif5a1	6.41E-40	0.39942127	0.984	0.963	1.19E-35	4	Eif5a
Rpl61	8.71E-40	0.25471204	0.996	0.994	1.62E-35	4	Rpl6
Ybx3	5.25E-39	0.45440088	0.602	0.374	9.75E-35	4	Ybx3
Fam49b1	7.08E-39	0.39739402	0.98	0.935	1.32E-34	4	Fam49b
Ciao2a	2.95E-38	0.44884353	0.75	0.558	5.49E-34	4	Ciao2a
Rps11	3.65E-38	0.27223929	1	0.995	6.77E-34	4	Rps11
Rpl35a	3.70E-38	0.22654505	0.998	0.995	6.87E-34	4	Rpl35a
Rpl7	1.52E-37	0.26123367	0.996	0.989	2.82E-33	4	Rpl7
Fdft1	2.24E-37	0.30623311	0.256	0.086	4.16E-33	4	Fdft1
Polr2e1	6.38E-37	0.41231643	0.822	0.666	1.19E-32	4	Polr2e
Cebpd	7.95E-37	0.42779063	0.426	0.199	1.48E-32	4	Cebpd
Arhgdib1	1.65E-36	0.38025885	0.98	0.937	3.07E-32	4	Arhgdib
Pdlim1	2.52E-36	0.28201568	0.225	0.068	4.68E-32	4	Pdlim1
Slc44a2	2.92E-36	0.31308152	0.346	0.146	5.43E-32	4	Slc44a2
Rps171	3.59E-36	0.35278484	0.98	0.97	6.68E-32	4	Rps17
Tmem176a	8.15E-36	0.26035596	0.262	0.088	1.51E-31	4	Tmem176a
Rps12	9.76E-36	0.27155643	0.99	0.99	1.81E-31	4	Rps12
Rps16	2.31E-35	0.22035458	0.998	0.995	4.30E-31	4	Rps16
Klrd11	2.35E-35	0.61011981	0.59	0.365	4.36E-31	4	Klrd1
Prdx61	5.12E-35	0.48510009	0.822	0.665	9.52E-31	4	Prdx6
Rpl33	1.17E-34	0.29866896	1	0.988	2.17E-30	4	Rpl3

Hmgcr	2.16E-34	0.67079009	0.666	0.474	4.01E-30	4	Hmgcr
Neurl31	3.35E-34	0.48026447	0.602	0.362	6.22E-30	4	Neurl3
Rexo21	9.40E-34	0.456361	0.73	0.57	1.75E-29	4	Rexo2
Hist1h1e1	1.89E-33	0.53670191	0.475	0.254	3.51E-29	4	Hist1h1e
Rps31	3.33E-33	0.24482521	1	0.992	6.19E-29	4	Rps3
Eef1a1	4.00E-33	0.2034061	0.998	0.997	7.43E-29	4	Eef1a1
Psma71	4.70E-33	0.35126634	0.969	0.95	8.74E-29	4	Psma7
Hsd17b7	8.52E-33	0.27559206	0.221	0.072	1.58E-28	4	Hsd17b7
Ctss1	3.28E-32	0.35693197	0.998	0.979	6.10E-28	4	Ctss
Cyba	5.08E-32	0.28653183	0.994	0.986	9.44E-28	4	Cyba
Dbi1	1.38E-31	0.45241806	0.926	0.836	2.56E-27	4	Dbi
Eif1	1.69E-31	0.28517946	0.998	0.992	3.14E-27	4	Eif1
Rps7	2.39E-31	0.23235585	0.994	0.989	4.44E-27	4	Rps7
Anxa11	2.81E-31	0.55664775	0.941	0.878	5.23E-27	4	Anxa1
Card19	3.37E-31	0.46480474	0.707	0.558	6.26E-27	4	Card19
Gpx41	3.81E-31	0.42911033	0.844	0.735	7.07E-27	4	Gpx4
Ssh21	3.90E-31	0.34950044	0.52	0.308	7.24E-27	4	Ssh2
Smim31	4.19E-31	0.3525503	0.58	0.354	7.78E-27	4	Smim3
Ccr5	9.23E-31	0.42757417	0.484	0.267	1.72E-26	4	Ccr5
Il1r21	1.05E-30	0.65721075	0.562	0.354	1.94E-26	4	Il1r2
Rpl18	1.83E-30	0.2343665	1	0.993	3.40E-26	4	Rpl18
Ms4a6b	2.42E-30	0.38038539	0.4	0.202	4.49E-26	4	Ms4a6b
Rpl7a	1.15E-29	0.25141794	0.996	0.983	2.13E-25	4	Rpl7a

Rpl42	1.75E-29	0.26654932	0.99	0.975	3.24E-25	4	Rpl4
Tecr1	2.94E-29	0.37315813	0.658	0.485	5.45E-25	4	Tecr
H3f3b1	4.58E-29	0.2936704	1	0.994	8.51E-25	4	H3f3b
Msrb1	6.29E-29	0.44032535	0.521	0.343	1.17E-24	4	Msrb1
Rpl91	1.28E-28	0.23459758	0.998	0.993	2.38E-24	4	Rpl9
Rpl18a	1.32E-28	0.22969656	0.998	0.995	2.46E-24	4	Rpl18a
Sms	1.73E-28	0.42137169	0.717	0.564	3.21E-24	4	Sms
Lgmn1	2.03E-28	0.20835002	0.424	0.214	3.77E-24	4	Lgmn
Fth11	3.44E-28	0.54489691	0.998	0.995	6.38E-24	4	Fth1
Bcl3	8.74E-28	0.36421382	0.438	0.246	1.62E-23	4	Bcl3
Rpl10a	1.21E-27	0.25532488	0.992	0.989	2.26E-23	4	Rpl10a
Rgcc	1.36E-27	0.42782595	0.232	0.087	2.53E-23	4	Rgcc
U2af11	1.42E-27	0.3613131	0.877	0.805	2.64E-23	4	U2af1
Emb1	1.48E-27	0.35974434	0.496	0.3	2.74E-23	4	Emb
Nop101	1.89E-27	0.42781648	0.789	0.645	3.52E-23	4	Nop10
Itgb7	2.29E-27	0.35596094	0.957	0.878	4.26E-23	4	Itgb7
Dock4	2.70E-27	0.21310047	0.238	0.09	5.01E-23	4	Dock4
Fxyd51	2.79E-27	0.35311282	0.988	0.969	5.18E-23	4	Fxyd5
Anxa5	4.35E-27	0.37553433	0.936	0.883	8.08E-23	4	Anxa5
Nme21	4.79E-27	0.30950148	0.982	0.951	8.90E-23	4	Nme2
Selenop	6.87E-27	0.22932864	0.232	0.089	1.28E-22	4	Selenop
Srsf3	9.19E-27	0.32597498	0.982	0.965	1.71E-22	4	Srsf3
Snx181	1.37E-26	0.31697579	0.359	0.179	2.55E-22	4	Snx18

Emp1	1.52E-26	0.36423527	0.277	0.118	2.83E-22	4	Emp1
Lmo4	1.61E-26	0.43541107	0.605	0.423	2.99E-22	4	Lmo4
Sirt3	1.74E-26	0.30737508	0.387	0.205	3.23E-22	4	Sirt3
Sc5d	2.86E-26	0.3441405	0.297	0.138	5.31E-22	4	Sc5d
Map2k3	5.43E-26	0.36949135	0.697	0.539	1.01E-21	4	Map2k3
Dhcr24	6.58E-26	0.24044303	0.215	0.08	1.22E-21	4	Dhcr24
Rnf130	1.11E-25	0.37772436	0.729	0.594	2.05E-21	4	Rnf130
Eif4e3	1.37E-25	0.23886432	0.283	0.127	2.55E-21	4	Eif4e3
Nfe2l21	2.11E-25	0.42145588	0.855	0.702	3.92E-21	4	Nfe2l2
Stard4	2.56E-25	0.32906245	0.268	0.118	4.76E-21	4	Stard4
Sorl1	3.27E-25	0.29273909	0.307	0.143	6.08E-21	4	Sorl1
Reep5	5.25E-25	0.32316775	0.963	0.935	9.76E-21	4	Reep5
Ybx11	7.67E-25	0.29153991	0.988	0.96	1.43E-20	4	Ybx1
Dusp2	1.09E-24	0.40412969	0.506	0.309	2.02E-20	4	Dusp2
Ndufb1-ps1	1.24E-24	0.26680706	0.961	0.951	2.31E-20	4	Ndufb1-ps
Crtc31	1.70E-24	0.33830838	0.426	0.244	3.15E-20	4	Crtc3
Rpl24	1.97E-24	0.21196911	0.998	0.991	3.65E-20	4	Rpl24
Atp5h1	2.09E-24	0.2870837	0.98	0.959	3.88E-20	4	Atp5h
Ly86	2.58E-24	0.3615394	0.848	0.778	4.79E-20	4	Ly86
Cdkn1a	3.12E-24	0.38815208	0.637	0.451	5.80E-20	4	Cdkn1a
Mrpl521	3.35E-24	0.35250309	0.977	0.938	6.22E-20	4	Mrpl52
Pitpna	5.15E-24	0.35295132	0.979	0.932	9.57E-20	4	Pitpna
Trf	5.38E-24	0.4701813	0.658	0.511	9.99E-20	4	Trf

Insig1	5.82E-24	0.47476121	0.465	0.29	1.08E-19	4	Insig1
Camkk2	5.99E-24	0.20800502	0.26	0.111	1.11E-19	4	Camkk2
Denr	6.84E-24	0.34253241	0.588	0.411	1.27E-19	4	Denr
Ifitm21	1.23E-23	0.65802277	0.957	0.959	2.29E-19	4	Ifitm2
Cdk2ap21	1.47E-23	0.4333205	0.818	0.707	2.74E-19	4	Cdk2ap2
H2afy	2.53E-23	0.29171421	0.994	0.976	4.71E-19	4	H2afy
Rpl101	3.45E-23	0.22503874	0.99	0.985	6.40E-19	4	Rpl10
Pde4b1	6.62E-23	0.403559	0.74	0.558	1.23E-18	4	Pde4b
Mar-02	1.13E-22	0.33243112	0.699	0.543	2.09E-18	4	Mar-02
Mvd	2.36E-22	0.30906061	0.24	0.105	4.38E-18	4	Mvd
Lrrfip1	3.05E-22	0.32036501	0.85	0.774	5.67E-18	4	Lrrfip1
Cib1	3.70E-22	0.31290236	0.445	0.277	6.88E-18	4	Cib1
Btf3	5.68E-22	0.24044105	0.98	0.968	1.06E-17	4	Btf3
Pnp	5.76E-22	0.29090478	0.867	0.765	1.07E-17	4	Pnp
Rpl36a1	6.92E-22	0.24185822	0.988	0.973	1.29E-17	4	Rpl36a
Psenen	9.32E-22	0.30916994	0.844	0.752	1.73E-17	4	Psenen
Rpl311	9.49E-22	0.26438259	0.98	0.966	1.76E-17	4	Rpl31
Ccnd2	1.61E-21	0.25656887	0.459	0.266	2.99E-17	4	Ccnd2
Rpl22l11	1.65E-21	0.33663782	0.957	0.918	3.06E-17	4	Rpl22l1
Rps25	1.68E-21	0.23316994	0.994	0.981	3.12E-17	4	Rps25
Hint11	2.70E-21	0.3011647	0.963	0.934	5.02E-17	4	Hint1
Gcnt21	2.85E-21	0.46611212	0.861	0.723	5.29E-17	4	Gcnt2
Degs1	3.40E-21	0.31209462	0.531	0.369	6.32E-17	4	Degs1

Lpcat21	3.93E-21	0.22308696	0.293	0.14	7.30E-17	4	Lpcat2
Rnh1	3.95E-21	0.3989184	0.947	0.9	7.34E-17	4	Rnh1
Limd2	5.13E-21	0.3824313	0.613	0.46	9.52E-17	4	Limd2
Lamp1	6.40E-21	0.3119211	0.652	0.489	1.19E-16	4	Lamp1
Casp1	8.97E-21	0.29018456	0.441	0.277	1.67E-16	4	Casp1
Vrk1	1.47E-20	0.35013886	0.873	0.796	2.73E-16	4	Vrk1
Cfp	1.64E-20	0.42359836	0.701	0.597	3.04E-16	4	Cfp
Tmem1471	1.90E-20	0.30688064	0.584	0.435	3.53E-16	4	Tmem147
Snrpf1	3.12E-20	0.28537581	0.961	0.916	5.80E-16	4	Snrpf
Npm1	3.47E-20	0.26098052	0.992	0.973	6.45E-16	4	Npm1
Ahnak	4.20E-20	0.40528151	0.99	0.946	7.80E-16	4	Ahnak
Magohb1	4.23E-20	0.30013962	0.533	0.376	7.85E-16	4	Magohb
Sptssa1	8.08E-20	0.32595793	0.689	0.57	1.50E-15	4	Sptssa
Anp32b	8.74E-20	0.3101169	0.943	0.91	1.62E-15	4	Anp32b
Ly6e1	1.00E-19	0.32583041	0.904	0.78	1.86E-15	4	Ly6e
Gas5	1.10E-19	0.35249875	0.986	0.956	2.05E-15	4	Gas5
Rac2	1.22E-19	0.32372522	0.873	0.787	2.26E-15	4	Rac2
Snrpg	1.25E-19	0.25716223	0.965	0.942	2.32E-15	4	Snrpg
Milr1	1.48E-19	0.25205976	0.367	0.21	2.75E-15	4	Milr1
Hnrnpa1	1.95E-19	0.30310172	0.975	0.938	3.63E-15	4	Hnrnpa1
mt-Cytb	1.98E-19	0.25082061	0.994	0.989	3.68E-15	4	mt-Cytb
Ywhae	2.70E-19	0.28110553	0.971	0.954	5.01E-15	4	Ywhae
Cd44	5.61E-19	0.38757583	0.807	0.694	1.04E-14	4	Cd44

Hck1	6.40E-19	0.30196857	0.729	0.592	1.19E-14	4	Hck
Cmtm71	6.46E-19	0.34368086	0.846	0.764	1.20E-14	4	Cmtm7
Wdr83os1	7.12E-19	0.29833222	0.693	0.574	1.32E-14	4	Wdr83os
Sec11c	8.16E-19	0.28907219	0.918	0.873	1.52E-14	4	Sec11c
Itgax1	8.22E-19	0.28953413	0.488	0.329	1.53E-14	4	Itgax
Gnb2	1.17E-18	0.24263101	0.982	0.965	2.17E-14	4	Gnb2
Psmd8	1.23E-18	0.29610501	0.893	0.827	2.28E-14	4	Psmd8
Mrfap1	1.56E-18	0.3043919	0.807	0.723	2.89E-14	4	Mrfap1
Lbr	2.14E-18	0.29132747	0.498	0.337	3.98E-14	4	Lbr
Gch1	2.50E-18	0.32315136	0.533	0.386	4.64E-14	4	Gch1
Crip11	2.65E-18	0.3091958	0.998	0.996	4.93E-14	4	Crip1
Ezr	2.87E-18	0.33799692	0.846	0.738	5.34E-14	4	Ezr
Syk	3.31E-18	0.27876856	0.576	0.43	6.15E-14	4	Syk
Cebpb	4.00E-18	0.44096023	0.518	0.372	7.44E-14	4	Cebpb
Ctsz	4.29E-18	0.24982743	0.979	0.963	7.98E-14	4	Ctsz
Dpm31	4.31E-18	0.28626227	0.734	0.612	8.01E-14	4	Dpm3
Fkbp31	5.03E-18	0.30425995	0.674	0.546	9.35E-14	4	Fkbp3
Pid1	5.18E-18	0.3233187	0.654	0.522	9.62E-14	4	Pid1
Cox5a1	6.10E-18	0.25604181	0.936	0.91	1.13E-13	4	Cox5a
BC035044	8.69E-18	0.30164034	0.502	0.355	1.61E-13	4	BC035044
Set1	8.94E-18	0.30107797	0.959	0.931	1.66E-13	4	Set
Lamtor41	1.01E-17	0.33917355	0.643	0.528	1.87E-13	4	Lamtor4
Treml2	1.24E-17	0.26899505	0.285	0.15	2.30E-13	4	Treml2

Jtb1	1.24E-17	0.33335736	0.803	0.707	2.31E-13	4	Jtb
Serbp1	1.28E-17	0.26988328	0.973	0.949	2.37E-13	4	Serbp1
Ppib1	1.73E-17	0.2342291	0.982	0.952	3.22E-13	4	Ppib
Scpep1	1.73E-17	0.24200161	0.504	0.349	3.22E-13	4	Scpep1
Mdh21	1.96E-17	0.3442231	0.781	0.728	3.64E-13	4	Mdh2
Lat2	2.63E-17	0.30737106	0.68	0.552	4.89E-13	4	Lat2
Pa2g41	2.79E-17	0.32066643	0.715	0.602	5.19E-13	4	Pa2g4
Ctsa	3.47E-17	0.31406309	0.475	0.33	6.46E-13	4	Ctsa
Stk40	3.71E-17	0.26302865	0.443	0.288	6.89E-13	4	Stk40
Acaa2	4.44E-17	0.23619013	0.418	0.27	8.25E-13	4	Acaa2
Akr7a5	5.00E-17	0.26836559	0.469	0.323	9.30E-13	4	Akr7a5
Ndufb101	5.02E-17	0.27706797	0.885	0.831	9.32E-13	4	Ndufb10
Mrto4	5.54E-17	0.29759369	0.549	0.414	1.03E-12	4	Mrto4
Krtcap21	5.71E-17	0.26771727	0.861	0.8	1.06E-12	4	Krtcap2
Rassf11	7.26E-17	0.31976993	0.736	0.647	1.35E-12	4	Rassf1
Mgst3	7.73E-17	0.26224159	0.357	0.216	1.44E-12	4	Mgst3
Cdk41	8.32E-17	0.27793007	0.525	0.392	1.55E-12	4	Cdk4
Idh21	9.58E-17	0.23739728	0.375	0.231	1.78E-12	4	Idh2
Emc61	9.71E-17	0.27277919	0.754	0.641	1.80E-12	4	Emc6
Magoh	1.14E-16	0.27837932	0.793	0.681	2.11E-12	4	Magoh
Npm3	1.36E-16	0.24180272	0.539	0.402	2.54E-12	4	Npm3
Tmem109	1.41E-16	0.25688548	0.473	0.34	2.61E-12	4	Tmem109
Mrpl331	1.46E-16	0.27468734	0.893	0.836	2.72E-12	4	Mrpl33

Ppdpf	1.73E-16	0.23018599	0.496	0.353	3.22E-12	4	Ppdpf
Stap1	1.75E-16	0.30994023	0.463	0.321	3.26E-12	4	Stap1
Ran1	2.46E-16	0.28233487	0.93	0.875	4.57E-12	4	Ran
Eif4a1	3.17E-16	0.24541435	0.979	0.97	5.89E-12	4	Eif4a1
Snrpd31	3.72E-16	0.27596004	0.848	0.783	6.91E-12	4	Snrpd3
Arl6ip5	3.91E-16	0.2964628	0.721	0.62	7.27E-12	4	Arl6ip5
Cuedc21	4.66E-16	0.24052029	0.59	0.469	8.66E-12	4	Cuedc2
Tpm4	4.71E-16	0.27606197	0.943	0.905	8.75E-12	4	Tpm4
Napsa1	6.85E-16	0.23668392	0.992	0.961	1.27E-11	4	Napsa
Hist1h4d	8.97E-16	0.26190826	0.428	0.286	1.67E-11	4	Hist1h4d
Mettl23	1.16E-15	0.25363539	0.645	0.528	2.15E-11	4	Mettl23
Mtpn	1.24E-15	0.30135537	0.838	0.792	2.31E-11	4	Mtpn
Tle3	1.30E-15	0.30430572	0.557	0.424	2.42E-11	4	Tle3
Cycs1	1.89E-15	0.29265948	0.938	0.904	3.50E-11	4	Cycs
Clic11	2.61E-15	0.20138259	0.99	0.973	4.85E-11	4	Clic1
Atp5g11	2.82E-15	0.22411175	0.943	0.891	5.24E-11	4	Atp5g1
Iqgap2	3.09E-15	0.28642215	0.721	0.602	5.75E-11	4	Iqgap2
Tgfb1	4.13E-15	0.28887464	0.771	0.701	7.67E-11	4	Tgfb1
Thoc71	4.37E-15	0.29500365	0.697	0.589	8.11E-11	4	Thoc7
BC005624	5.15E-15	0.25531659	0.701	0.599	9.56E-11	4	BC005624
Trmt1121	5.91E-15	0.28318223	0.77	0.679	1.10E-10	4	Trmt112
Selenof1	6.78E-15	0.27070923	0.881	0.812	1.26E-10	4	Selenof
Vapa	6.82E-15	0.25171327	0.842	0.782	1.27E-10	4	Vapa

Nol7	9.27E-15	0.26367494	0.918	0.856	1.72E-10	4	Nol7
Dkc1	9.43E-15	0.21246732	0.387	0.247	1.75E-10	4	Dkc1
Pdap11	1.02E-14	0.22837176	0.84	0.757	1.89E-10	4	Pdap1
Slc25a5	1.05E-14	0.23293286	0.971	0.928	1.95E-10	4	Slc25a5
2310039H08Rik	2.29E-14	0.21004538	0.348	0.22	4.26E-10	4	2310039H08Rik
Il1b1	2.83E-14	0.53858152	0.936	0.893	5.27E-10	4	Il1b
Aldh21	3.16E-14	0.23016096	0.475	0.342	5.87E-10	4	Aldh2
Msn	3.51E-14	0.26738262	0.943	0.921	6.52E-10	4	Msn
Mif1	4.19E-14	0.26588464	0.826	0.748	7.78E-10	4	Mif
Sec61b1	4.25E-14	0.2094536	0.963	0.948	7.90E-10	4	Sec61b
Ranbp11	4.61E-14	0.28911821	0.852	0.811	8.57E-10	4	Ranbp1
Sigmar1	5.25E-14	0.26933173	0.676	0.568	9.76E-10	4	Sigmar1
AA467197	6.34E-14	0.30943838	0.264	0.147	1.18E-09	4	AA467197
Gng51	6.41E-14	0.22015327	0.971	0.934	1.19E-09	4	Gng5
Cnbp1	6.55E-14	0.22541573	0.955	0.939	1.22E-09	4	Cnbp
2410006H16Rik2	7.14E-14	0.27372599	0.939	0.902	1.33E-09	4	2410006H16Rik
Eif2s2	7.61E-14	0.23640864	0.977	0.956	1.41E-09	4	Eif2s2
Irf2bpl	7.90E-14	0.21515603	0.299	0.177	1.47E-09	4	Irf2bpl
Lsm71	8.09E-14	0.2296074	0.6	0.48	1.50E-09	4	Lsm7
Lta4h1	1.07E-13	0.26000478	0.473	0.345	1.99E-09	4	Lta4h
Klf13	1.20E-13	0.2713888	0.631	0.521	2.22E-09	4	Klf13
D8Ertd738e1	1.30E-13	0.24486746	0.938	0.899	2.42E-09	4	D8Ertd738e
Snrpd21	1.44E-13	0.2421778	0.898	0.853	2.67E-09	4	Snrpd2

Tmem14c1	1.53E-13	0.23823654	0.889	0.833	2.84E-09	4	Tmem14c
Srsf7	1.74E-13	0.21607799	0.939	0.899	3.23E-09	4	Srsf7
Dhx40	1.84E-13	0.30161793	0.391	0.263	3.42E-09	4	Dhx40
Nfkbia	1.87E-13	0.25637995	0.811	0.698	3.48E-09	4	Nfkbia
Lilrb4a	2.12E-13	0.24610178	0.373	0.243	3.93E-09	4	Lilrb4a
Tctex1d2	2.90E-13	0.20304904	0.312	0.196	5.39E-09	4	Tctex1d2
Hist1h1c1	4.75E-13	0.29807618	0.465	0.327	8.83E-09	4	Hist1h1c
Dnajc15	5.36E-13	0.24369429	0.705	0.619	9.97E-09	4	Dnajc15
Mrpl231	5.71E-13	0.22724631	0.643	0.542	1.06E-08	4	Mrpl23
Ptpn6	6.37E-13	0.21680447	0.766	0.689	1.18E-08	4	Ptpn6
Hnrnpa3	6.42E-13	0.25526509	0.971	0.951	1.19E-08	4	Hnrnpa3
Nudc1	7.53E-13	0.23721345	0.809	0.745	1.40E-08	4	Nudc
Ier21	7.70E-13	0.23723581	0.961	0.933	1.43E-08	4	Ier2
Snhg8	8.06E-13	0.276187	0.775	0.71	1.50E-08	4	Snhg8
Ccdc28b	8.45E-13	0.22725127	0.455	0.339	1.57E-08	4	Ccdc28b
Gapdh1	8.82E-13	0.206454	0.973	0.947	1.64E-08	4	Gapdh
Smc3	1.20E-12	0.25468298	0.541	0.417	2.22E-08	4	Smc3
Tnfaip81	1.24E-12	0.25905171	0.916	0.862	2.30E-08	4	Tnfaip8
Nop58	1.30E-12	0.3380096	0.811	0.724	2.41E-08	4	Nop58
Taf13	1.59E-12	0.22134395	0.572	0.461	2.95E-08	4	Taf13
Hk2	1.78E-12	0.30753108	0.742	0.645	3.31E-08	4	Hk2
Lsm12	2.06E-12	0.24053047	0.785	0.717	3.83E-08	4	Lsm12
Hist1h2ae	2.59E-12	0.21690502	0.258	0.15	4.82E-08	4	Hist1h2ae

Flna	2.93E-12	0.21679267	0.916	0.836	5.45E-08	4	Flna
Atp5d1	2.97E-12	0.20460261	0.959	0.925	5.52E-08	4	Atp5d
Mcl11	3.28E-12	0.21610863	0.986	0.981	6.09E-08	4	Mcl1
Eif3f	3.52E-12	0.22981007	0.977	0.946	6.54E-08	4	Eif3f
Psmb21	4.35E-12	0.20006751	0.893	0.839	8.08E-08	4	Psmb2
Ndufa71	4.68E-12	0.2275188	0.928	0.894	8.70E-08	4	Ndufa7
Znrd11	4.94E-12	0.23452066	0.793	0.714	9.17E-08	4	Znrd1
Eif3i1	6.26E-12	0.21361367	0.898	0.841	1.16E-07	4	Eif3i
Gabarapl1	8.03E-12	0.20056021	0.379	0.254	1.49E-07	4	Gabarapl1
Tppp3	8.23E-12	0.30768498	0.23	0.131	1.53E-07	4	Tppp3
Park71	9.15E-12	0.2096615	0.918	0.886	1.70E-07	4	Park7
Rsl1d1	1.76E-11	0.25059834	0.656	0.573	3.26E-07	4	Rsl1d1
Macf1	1.88E-11	0.31760651	0.639	0.545	3.49E-07	4	Macf1
Ebp	2.75E-11	0.24307054	0.6	0.509	5.10E-07	4	Ebp
Abracl	2.90E-11	0.21002822	0.943	0.893	5.39E-07	4	Abracl
Anp32a1	3.11E-11	0.23074569	0.809	0.752	5.79E-07	4	Anp32a
0610012G03Rik	3.26E-11	0.23333755	0.564	0.472	6.06E-07	4	0610012G03Rik
Ms4a6d	3.37E-11	0.24759622	0.207	0.113	6.27E-07	4	Ms4a6d
Erh	3.41E-11	0.2136248	0.605	0.512	6.34E-07	4	Erh
Tle5	3.46E-11	0.20146141	0.85	0.792	6.43E-07	4	Tle5
Snu13	3.92E-11	0.23531883	0.877	0.821	7.29E-07	4	Snu13
Sf3b6	4.04E-11	0.21024279	0.861	0.815	7.51E-07	4	Sf3b6
Ndufa9	4.61E-11	0.20343874	0.604	0.5	8.57E-07	4	Ndufa9

Serinc3	4.88E-11	0.22056315	0.715	0.642	9.06E-07	4	Serinc3
Ric8a	4.91E-11	0.23665512	0.525	0.431	9.13E-07	4	Ric8a
D16Ertd472e	6.88E-11	0.24868657	0.465	0.355	1.28E-06	4	D16Ertd472e
Hist3h2a1	7.24E-11	0.26492303	0.488	0.374	1.35E-06	4	Hist3h2a
Eef1e1	7.64E-11	0.20841841	0.5	0.395	1.42E-06	4	Eef1e1
Snx201	1.05E-10	0.27053167	0.875	0.827	1.94E-06	4	Snx20
Cldnd1	1.07E-10	0.27603251	0.867	0.79	1.98E-06	4	Cldnd1
Hsd17b12	1.10E-10	0.24457649	0.689	0.624	2.04E-06	4	Hsd17b12
Tcf4	1.17E-10	0.26211851	0.412	0.297	2.17E-06	4	Tcf4
Cmas	1.63E-10	0.21879456	0.57	0.472	3.02E-06	4	Cmas
Cebpz	1.66E-10	0.24845249	0.828	0.786	3.08E-06	4	Cebpz
Tomm61	1.75E-10	0.22253838	0.908	0.868	3.26E-06	4	Tomm6
Rwdd1	1.88E-10	0.23887504	0.793	0.734	3.49E-06	4	Rwdd1
Fbl	1.95E-10	0.24958827	0.887	0.845	3.62E-06	4	Fbl
Prmt11	2.31E-10	0.21774461	0.725	0.652	4.29E-06	4	Prmt1
Irf1	3.07E-10	0.28078092	0.445	0.323	5.70E-06	4	Irf1
Ndufb41	4.11E-10	0.20449949	0.781	0.712	7.63E-06	4	Ndufb4
Vps37b2	4.73E-10	0.27202623	0.697	0.605	8.79E-06	4	Vps37b
Mrpl571	5.15E-10	0.217645	0.74	0.668	9.58E-06	4	Mrpl57
Gng101	6.05E-10	0.21336079	0.971	0.921	1.12E-05	4	Gng10
Paip2	6.50E-10	0.20821637	0.719	0.654	1.21E-05	4	Paip2
Ctsl	7.31E-10	0.21054528	0.232	0.139	1.36E-05	4	Ctsl
Otulinl	7.99E-10	0.24781562	0.838	0.773	1.48E-05	4	Otulinl

Hist1h2ap	1.12E-09	0.25645533	0.43	0.339	2.08E-05	4	Hist1h2ap
Parp1	1.12E-09	0.21652241	0.414	0.311	2.08E-05	4	Parp1
Ccdc121	1.18E-09	0.20408117	0.947	0.918	2.20E-05	4	Ccdc12
Nme11	1.55E-09	0.21749179	0.816	0.753	2.87E-05	4	Nme1
Rab10os	2.13E-09	0.23628841	0.51	0.426	3.95E-05	4	Rab10os
Snrpb	2.23E-09	0.20129855	0.939	0.889	4.14E-05	4	Snrpb
Arhgap45	2.31E-09	0.24265463	0.662	0.591	4.30E-05	4	Arhgap45
Scd2	4.56E-09	0.21788785	0.363	0.263	8.47E-05	4	Scd2
Mxd1	4.61E-09	0.24018282	0.424	0.314	8.57E-05	4	Mxd1
Morf4l2	4.84E-09	0.22993776	0.646	0.583	8.99E-05	4	Morf4l2
Clint1	4.98E-09	0.24561438	0.725	0.687	9.26E-05	4	Clint1
Naa38	5.08E-09	0.20850836	0.629	0.564	9.43E-05	4	Naa38
Cotl11	5.26E-09	0.21147419	0.936	0.926	9.78E-05	4	Cotl1
Lyn	6.28E-09	0.21757286	0.945	0.916	0.00011662	4	Lyn
B4galnt1	8.05E-09	0.22725347	0.518	0.438	0.00014952	4	B4galnt1
Tmed5	1.10E-08	0.22050336	0.896	0.867	0.00020508	4	Tmed5
Bnip3l	1.19E-08	0.21421764	0.557	0.48	0.00022078	4	Bnip3l
Matk	1.39E-08	0.2054536	0.27	0.179	0.00025743	4	Matk
Eno11	1.75E-08	0.20777958	0.609	0.551	0.00032487	4	Eno1
Tsc22d31	1.77E-08	0.2528316	0.652	0.555	0.00032853	4	Tsc22d3
Prr7	1.97E-08	0.2150289	0.299	0.212	0.00036607	4	Prr7
Il6ra	2.30E-08	0.22371991	0.555	0.474	0.00042721	4	Il6ra
Rbis1	2.93E-08	0.20244353	0.764	0.718	0.00054382	4	Rbis

Zfand51	4.14E-08	0.28070987	0.916	0.888	0.00076928	4	Zfand5
Smc6	5.82E-08	0.220142	0.607	0.528	0.00108141	4	Smc6
Lrrfip2	5.99E-08	0.23536822	0.639	0.595	0.00111241	4	Lrrfip2
Slc15a3	7.27E-08	0.20655945	0.4	0.305	0.00135041	4	Slc15a3
Tnfaip21	7.29E-08	0.29532567	0.309	0.213	0.00135506	4	Tnfaip2
Rheb2	8.20E-08	0.20016937	0.848	0.82	0.00152315	4	Rheb
Tep1	8.76E-08	0.21618453	0.611	0.542	0.00162669	4	Tep1
Cct6a	8.96E-08	0.20180987	0.773	0.738	0.0016655	4	Cct6a
Ttc7	1.77E-07	0.21203323	0.645	0.598	0.00329433	4	Ttc7
Ifrd11	2.86E-07	0.22756353	0.836	0.775	0.00530514	4	Ifrd1
Pnn	5.34E-07	0.20414444	0.605	0.535	0.00991557	4	Pnn
Fmnl1	5.48E-07	0.20155158	0.674	0.618	0.01018741	4	Fmnl1
Xist	5.79E-07	0.23914769	0.734	0.668	0.01075473	4	Xist
Ddx21	7.06E-07	0.20325111	0.814	0.79	0.01310968	4	Ddx21
Runx31	7.34E-07	0.27414825	0.678	0.647	0.01363382	4	Runx3
Xrn2	1.18E-06	0.20446884	0.836	0.818	0.02199755	4	Xrn2
Irf2	1.67E-06	0.20338512	0.67	0.638	0.03110911	4	Irf2
Ncl	1.68E-06	0.21325258	0.965	0.952	0.03121656	4	Ncl
Smap2	2.04E-06	0.21778133	0.596	0.549	0.03797411	4	Smap2
Ing2	5.25E-06	0.20715252	0.365	0.287	0.09752447	4	Ing2
Socs31	1.06E-05	0.26965823	0.477	0.407	0.19716719	4	Socs3
Ypel31	1.18E-05	0.20546387	0.354	0.288	0.21951748	4	Ypel3
Ywhah1	1.54E-05	0.20442497	0.895	0.891	0.28605842	4	Ywhah

Tnni2	7.98E-05	0.22837598	0.635	0.593	1	4	Tnni2
Ccl6	0.00011942	0.33897874	0.451	0.401	1	4	Ccl6
Wfdc171	0.0015194	0.29653611	0.701	0.674	1	4	Wfdc17
Manf	8.73E-232	1.85177693	0.986	0.773	1.62E-227	5	Manf
Creld2	2.91E-222	1.48081045	0.867	0.321	5.41E-218	5	Creld2
Hsp90b1	1.07E-214	1.51465924	0.998	0.945	1.98E-210	5	Hsp90b1
Sdf2l1	1.16E-212	1.81225313	0.971	0.716	2.16E-208	5	Sdf2l1
Hspa5	1.00E-203	1.490695	0.998	0.979	1.87E-199	5	Hspa5
Pdia6	4.09E-195	1.43057145	0.984	0.844	7.61E-191	5	Pdia6
Pdia3	1.14E-171	1.13411192	0.998	0.953	2.12E-167	5	Pdia3
Spcs2	4.50E-167	1.12966913	0.975	0.822	8.36E-163	5	Spcs2
Ccnb2	3.06E-160	0.503878	0.253	0.017	5.69E-156	5	Ccnb2
Calr	5.69E-160	1.1517699	0.994	0.939	1.06E-155	5	Calr
Dnajb11	1.82E-142	1.02300714	0.931	0.651	3.38E-138	5	Dnajb11
Dnajc3	7.07E-139	1.11494783	0.953	0.722	1.31E-134	5	Dnajc3
Sec61g	2.59E-137	0.80413247	0.994	0.963	4.81E-133	5	Sec61g
Pdia4	1.35E-133	1.04748896	0.816	0.405	2.51E-129	5	Pdia4
Slc35b1	2.70E-128	0.83949607	0.863	0.454	5.02E-124	5	Slc35b1
Tmed9	3.61E-127	0.85421868	0.978	0.816	6.70E-123	5	Tmed9
Nucb2	1.19E-123	1.04601848	0.831	0.448	2.21E-119	5	Nucb2
Sec61b2	2.44E-121	0.76443628	0.988	0.946	4.53E-117	5	Sec61b
Ppib2	3.87E-120	0.72831599	0.994	0.951	7.18E-116	5	Ppib
Hyou1	1.48E-117	0.82198541	0.737	0.317	2.75E-113	5	Hyou1

Ostc	9.21E-116	0.86533678	0.955	0.756	1.71E-111	5	Ostc
Phgdh	1.42E-111	0.49860076	0.314	0.049	2.64E-107	5	Phgdh
Selenok	1.70E-111	0.75674471	0.986	0.894	3.16E-107	5	Selenok
Mydgf	2.29E-111	0.78456605	0.916	0.593	4.25E-107	5	Mydgf
Tmed2	2.73E-110	0.81439783	0.961	0.789	5.07E-106	5	Tmed2
Fkbp2	4.54E-108	0.80709784	0.947	0.676	8.43E-104	5	Fkbp2
Krtcap22	2.39E-107	0.84615235	0.961	0.791	4.44E-103	5	Krtcap2
Sec11c1	2.47E-97	0.70700729	0.982	0.867	4.59E-93	5	Sec11c
Herpud1	5.37E-97	1.09412638	0.914	0.656	9.98E-93	5	Herpud1
Rpl36al	9.62E-96	0.50342835	0.998	0.98	1.79E-91	5	Rpl36al
Ssr2	3.19E-94	0.78885475	0.902	0.665	5.93E-90	5	Ssr2
Ddost	3.11E-90	0.70367087	0.906	0.701	5.78E-86	5	Ddost
Ube2c	1.92E-88	0.29443003	0.222	0.029	3.57E-84	5	Ube2c
Selenos	3.14E-88	0.72248305	0.955	0.819	5.83E-84	5	Selenos
Cdk2ap22	5.49E-87	0.8097788	0.914	0.698	1.02E-82	5	Cdk2ap2
Ssr3	6.03E-85	0.64300668	0.947	0.751	1.12E-80	5	Ssr3
Rpn1	1.88E-84	0.74455397	0.918	0.65	3.49E-80	5	Rpn1
Hist1h2bc	8.96E-78	0.851736	0.504	0.18	1.66E-73	5	Hist1h2bc
Selenof2	1.75E-72	0.56650436	0.945	0.806	3.25E-68	5	Selenof
Srp9	2.53E-71	0.59648098	0.945	0.823	4.69E-67	5	Srp9
Surf4	2.57E-70	0.5801316	0.845	0.583	4.78E-66	5	Surf4
Tmem2581	2.15E-68	0.57520525	0.961	0.868	3.99E-64	5	Tmem258
Sec11a	8.27E-68	0.5698278	0.906	0.68	1.54E-63	5	Sec11a

Hdlbp	9.54E-68	0.66737819	0.865	0.648	1.77E-63	5	Hdlbp
Eif2s21	1.51E-67	0.63875078	0.986	0.955	2.81E-63	5	Eif2s2
Serp1	1.58E-65	0.58383236	0.959	0.85	2.93E-61	5	Serp1
Eif4ebp1	7.58E-64	0.78562112	0.708	0.431	1.41E-59	5	Eif4ebp1
Gapdh2	2.15E-63	0.53877287	0.984	0.946	4.00E-59	5	Gapdh
Lman1	7.64E-63	0.52379742	0.649	0.329	1.42E-58	5	Lman1
Tbcb	2.90E-62	0.58896236	0.91	0.774	5.38E-58	5	Tbcb
Rrbp1	2.93E-62	0.57977564	0.978	0.926	5.45E-58	5	Rrbp1
Nars	5.30E-61	0.63061296	0.957	0.856	9.85E-57	5	Nars
Ppia2	1.52E-60	0.46833202	1	0.987	2.82E-56	5	Ppia
Srm	1.59E-60	0.4294051	0.482	0.189	2.96E-56	5	Srm
Cope	1.83E-60	0.56550654	0.924	0.777	3.39E-56	5	Cope
Rbm3	2.49E-59	0.44750747	0.982	0.965	4.63E-55	5	Rbm3
Elob1	2.60E-57	0.49532728	0.982	0.935	4.83E-53	5	Elob
Canx	6.44E-56	0.50896035	0.951	0.808	1.20E-51	5	Canx
P4hb	7.91E-56	0.55844561	0.937	0.78	1.47E-51	5	P4hb
Dnajb91	8.71E-56	0.60622247	0.824	0.539	1.62E-51	5	Dnajb9
Ift20	9.77E-56	0.53992074	0.833	0.599	1.82E-51	5	Ift20
Hspa13	4.64E-55	0.30231643	0.375	0.125	8.62E-51	5	Hspa13
Nme22	4.86E-55	0.50705503	0.99	0.95	9.04E-51	5	Nme2
Lman2	9.56E-55	0.51482281	0.9	0.731	1.78E-50	5	Lman2
Dad11	2.67E-54	0.48344028	0.969	0.891	4.97E-50	5	Dad1
AI662270	3.57E-54	0.52530529	0.929	0.77	6.62E-50	5	AI662270

Atp5b	2.76E-53	0.42624967	0.99	0.957	5.12E-49	5	Atp5b
Uba5	1.27E-52	0.43424139	0.635	0.342	2.37E-48	5	Uba5
Stmn1	7.48E-51	0.50698434	0.473	0.204	1.39E-46	5	Stmn1
Bcat2	8.08E-51	0.39686727	0.563	0.27	1.50E-46	5	Bcat2
Bet1	6.05E-50	0.42523566	0.531	0.256	1.12E-45	5	Bet1
Cks1b	9.73E-50	0.43006011	0.535	0.262	1.81E-45	5	Cks1b
Nans	1.40E-49	0.46763103	0.657	0.387	2.61E-45	5	Nans
Rpn2	2.46E-49	0.47284786	0.843	0.613	4.56E-45	5	Rpn2
Srp19	3.38E-49	0.52469946	0.824	0.61	6.27E-45	5	Srp19
Syvn1	8.91E-49	0.34188346	0.488	0.212	1.65E-44	5	Syvn1
Hist1h1c2	9.46E-49	0.61704404	0.608	0.313	1.76E-44	5	Hist1h1c
Atp5f11	5.71E-47	0.39795056	0.994	0.949	1.06E-42	5	Atp5f1
Lgals12	2.82E-46	0.61428854	0.975	0.877	5.25E-42	5	Lgals1
Slc7a5	2.83E-46	0.36838723	0.424	0.172	5.25E-42	5	Slc7a5
Spcs3	5.00E-45	0.46564195	0.771	0.507	9.29E-41	5	Spcs3
Snrpa	1.08E-44	0.44683828	0.749	0.503	2.01E-40	5	Snrpa
Trabd	2.33E-44	0.41311531	0.676	0.403	4.32E-40	5	Trabd
Oat	3.55E-43	0.43422251	0.706	0.429	6.59E-39	5	Oat
Slc39a7	3.79E-43	0.35863805	0.584	0.316	7.04E-39	5	Slc39a7
Psmb61	6.14E-43	0.42518917	0.935	0.841	1.14E-38	5	Psmb6
Ufm1	3.53E-42	0.43398893	0.859	0.676	6.56E-38	5	Ufm1
Snrpg1	4.00E-42	0.37434629	0.988	0.94	7.44E-38	5	Snrpg
Uqcrq1	4.93E-42	0.40766394	0.973	0.922	9.16E-38	5	Uqcrq

Srp72	7.71E-42	0.47011033	0.814	0.641	1.43E-37	5	Srp72
Ssr1	1.45E-41	0.42527501	0.837	0.604	2.70E-37	5	Ssr1
Tfg	2.86E-41	0.4076401	0.714	0.473	5.31E-37	5	Tfg
Plpp5	1.22E-40	0.25480437	0.294	0.1	2.27E-36	5	Plpp5
Tma7	1.72E-40	0.39853588	0.973	0.919	3.20E-36	5	Tma7
Ddx39	4.06E-40	0.40894985	0.735	0.492	7.55E-36	5	Ddx39
Atp2a2	4.06E-40	0.42513925	0.722	0.477	7.55E-36	5	Atp2a2
Hmgn2	1.01E-39	0.46488565	0.553	0.303	1.88E-35	5	Hmgn2
Rps172	1.46E-39	0.34460823	0.988	0.969	2.72E-35	5	Rps17
Nme12	2.60E-39	0.46767117	0.912	0.744	4.82E-35	5	Nme1
Hint12	5.02E-39	0.37787122	0.984	0.932	9.33E-35	5	Hint1
Ccdc134	8.17E-39	0.31287341	0.429	0.193	1.52E-34	5	Ccdc134
Uso1	9.35E-39	0.35493803	0.602	0.343	1.74E-34	5	Uso1
Sec61a1	9.92E-39	0.42854638	0.735	0.509	1.84E-34	5	Sec61a1
Ppa1	1.19E-38	0.40661618	0.735	0.491	2.20E-34	5	Ppa1
Derl1	1.32E-38	0.41072563	0.812	0.6	2.45E-34	5	Derl1
Cox5b1	3.08E-38	0.39464406	0.973	0.927	5.72E-34	5	Cox5b
Eif5a2	5.83E-38	0.35755813	0.984	0.963	1.08E-33	5	Eif5a
Kdelr2	9.36E-38	0.3889194	0.637	0.401	1.74E-33	5	Kdelr2
Tram1	1.09E-37	0.41653956	0.798	0.612	2.03E-33	5	Tram1
Tmed10	1.13E-37	0.38561775	0.971	0.897	2.10E-33	5	Tmed10
Mrps141	1.25E-37	0.39599101	0.91	0.772	2.32E-33	5	Mrps14
Gorasp2	1.95E-37	0.35806258	0.612	0.362	3.63E-33	5	Gorasp2

Sarnp1	4.27E-37	0.40307253	0.914	0.8	7.93E-33	5	Sarnp
Ufc1	3.71E-36	0.41209271	0.843	0.66	6.89E-32	5	Ufc1
Mthfd2	5.88E-36	0.49923136	0.614	0.396	1.09E-31	5	Mthfd2
Nucb1	6.35E-36	0.37000082	0.616	0.373	1.18E-31	5	Nucb1
S100a101	1.16E-35	0.55058275	0.965	0.87	2.16E-31	5	S100a10
Ftl1	2.24E-35	0.28435242	1	0.992	4.17E-31	5	Ftl1
Sdhb	3.38E-35	0.37557688	0.88	0.712	6.28E-31	5	Sdhb
Mgat2	6.52E-35	0.37220165	0.694	0.476	1.21E-30	5	Mgat2
Ufl1	1.12E-34	0.30002435	0.5	0.256	2.08E-30	5	Ufl1
Ndufb111	1.69E-34	0.38324571	0.947	0.882	3.14E-30	5	Ndufb11
Ndufa131	1.88E-34	0.3518832	0.986	0.93	3.48E-30	5	Ndufa13
Ptma2	2.67E-34	0.29332019	1	0.992	4.95E-30	5	Ptma
Hist1h3e	7.23E-34	0.20896305	0.284	0.103	1.34E-29	5	Hist1h3e
Mettl1	1.16E-33	0.33551083	0.51	0.27	2.16E-29	5	Mettl1
Selenoh	1.17E-33	0.45491848	0.661	0.446	2.17E-29	5	Selenoh
Lsm4	4.18E-33	0.40381603	0.91	0.807	7.77E-29	5	Lsm4
Hist1h1e2	7.08E-33	0.41489415	0.486	0.253	1.32E-28	5	Hist1h1e
Cox7b1	7.79E-33	0.3530401	0.98	0.924	1.45E-28	5	Cox7b
Ormdl2	7.94E-33	0.33182101	0.578	0.348	1.48E-28	5	Ormdl2
Ndufb81	8.65E-33	0.38511859	0.949	0.855	1.61E-28	5	Ndufb8
Psmb31	1.36E-32	0.36196771	0.959	0.879	2.52E-28	5	Psmb3
Btf31	2.59E-32	0.27564569	0.994	0.966	4.82E-28	5	Btf3
H1f0	2.65E-32	0.37902363	0.418	0.197	4.92E-28	5	H1f0

Hmgb12	3.25E-32	0.40210419	0.951	0.883	6.03E-28	5	Hmgb1
Sel1l	4.47E-32	0.31312057	0.48	0.254	8.30E-28	5	Sel1l
Cox8a1	5.61E-32	0.30661968	0.986	0.963	1.04E-27	5	Cox8a
Srprb	5.96E-32	0.24041373	0.388	0.178	1.11E-27	5	Srprb
Sars	6.41E-32	0.54888267	0.725	0.548	1.19E-27	5	Sars
Gmppb	6.47E-32	0.36028929	0.598	0.371	1.20E-27	5	Gmppb
Arf1	1.59E-31	0.35582717	0.951	0.895	2.95E-27	5	Arf1
Lrrc59	1.83E-31	0.38669296	0.716	0.492	3.40E-27	5	Lrrc59
Saraf	2.84E-31	0.37154156	0.771	0.576	5.27E-27	5	Saraf
Ddit3	4.79E-31	0.45603462	0.58	0.358	8.90E-27	5	Ddit3
Stt3a	1.57E-30	0.37431001	0.688	0.477	2.92E-26	5	Stt3a
Arpc5l	2.13E-30	0.45558879	0.896	0.765	3.95E-26	5	Arpc5l
Mrps28	2.17E-30	0.37297493	0.694	0.484	4.03E-26	5	Mrps28
Cdk5rap3	2.63E-30	0.33432533	0.578	0.352	4.88E-26	5	Cdk5rap3
Atp5a1	4.90E-30	0.31650719	0.969	0.919	9.10E-26	5	Atp5a1
Snrpf2	2.30E-29	0.33830944	0.982	0.914	4.27E-25	5	Snrpf
Ndufa21	2.35E-29	0.32872863	0.961	0.908	4.37E-25	5	Ndufa2
Txndc5	2.55E-29	0.33232974	0.708	0.469	4.74E-25	5	Txndc5
Copb2	3.82E-29	0.34838846	0.741	0.573	7.10E-25	5	Copb2
Psmc3	7.21E-29	0.36012405	0.882	0.769	1.34E-24	5	Psmc3
Iars	7.54E-29	0.31488287	0.508	0.285	1.40E-24	5	Iars
Mdh1	7.61E-29	0.38162798	0.904	0.79	1.41E-24	5	Mdh1
Atp5j1	9.61E-29	0.33004457	0.976	0.901	1.78E-24	5	Atp5j

Grpel1	1.12E-28	0.34577637	0.82	0.658	2.08E-24	5	Grpel1
Cox6a1	1.55E-28	0.31769093	0.967	0.903	2.88E-24	5	Cox6a1
Pdap12	1.55E-28	0.37158812	0.898	0.752	2.89E-24	5	Pdap1
Atp5g12	1.58E-28	0.36860883	0.957	0.89	2.94E-24	5	Atp5g1
Kctd14	1.72E-28	0.28976611	0.4	0.199	3.20E-24	5	Kctd14
Cnpy2	3.37E-28	0.31579991	0.588	0.376	6.26E-24	5	Cnpy2
H3f3b2	3.60E-28	0.34684723	1	0.994	6.69E-24	5	H3f3b
Prrc1	3.84E-28	0.24914236	0.406	0.205	7.13E-24	5	Prrc1
Ssr41	5.19E-28	0.33250747	0.978	0.927	9.63E-24	5	Ssr4
Erp44	7.57E-28	0.34174084	0.753	0.569	1.41E-23	5	Erp44
Erp291	1.20E-27	0.31415879	0.988	0.95	2.23E-23	5	Erp29
Txndc161	1.26E-27	0.78432902	0.667	0.448	2.35E-23	5	Txndc16
Chchd2	1.33E-27	0.26690778	0.994	0.971	2.47E-23	5	Chchd2
Vcp	1.58E-27	0.35056926	0.914	0.822	2.93E-23	5	Vcp
Psmb51	4.41E-27	0.33133681	0.937	0.857	8.20E-23	5	Psmb5
Stx5a	7.35E-27	0.28362138	0.584	0.37	1.37E-22	5	Stx5a
Cetn3	8.72E-27	0.33344149	0.771	0.556	1.62E-22	5	Cetn3
Atp5k1	9.34E-27	0.34129888	0.976	0.917	1.74E-22	5	Atp5k
Txndc11	1.16E-26	0.21956874	0.359	0.17	2.15E-22	5	Txndc11
Ndufa111	1.36E-26	0.33096711	0.851	0.719	2.52E-22	5	Ndufa11
Nmt1	1.58E-26	0.32330254	0.892	0.76	2.94E-22	5	Nmt1
Derl2	2.01E-26	0.29899929	0.68	0.479	3.73E-22	5	Derl2
Timm13	2.14E-26	0.33424469	0.906	0.807	3.99E-22	5	Timm13

Tomm71	5.06E-26	0.27839216	0.98	0.947	9.40E-22	5	Tomm7
Lysmd3	7.03E-26	0.32613612	0.543	0.332	1.31E-21	5	Lysmd3
Txnl1	1.23E-25	0.34350384	0.847	0.715	2.28E-21	5	Txnl1
Ndufa41	1.51E-25	0.31464862	0.976	0.924	2.81E-21	5	Ndufa4
Stt3b	1.66E-25	0.25345475	0.463	0.255	3.08E-21	5	Stt3b
Selenom	2.18E-25	0.24306371	0.355	0.171	4.05E-21	5	Selenom
Cox7a21	2.46E-25	0.31289484	0.961	0.917	4.58E-21	5	Cox7a2
Tes	3.96E-25	0.33605176	0.775	0.605	7.37E-21	5	Tes
Mlec	4.08E-25	0.3394101	0.782	0.594	7.59E-21	5	Mlec
Srsf71	5.80E-25	0.31256195	0.969	0.896	1.08E-20	5	Srsf7
Smdt1	5.86E-25	0.28255032	0.99	0.955	1.09E-20	5	Smdt1
Swi51	3.09E-24	0.34164112	0.869	0.765	5.75E-20	5	Swi5
Far1	5.48E-24	0.34321451	0.725	0.561	1.02E-19	5	Far1
Edem1	6.40E-24	0.2974568	0.62	0.419	1.19E-19	5	Edem1
Srp14	7.66E-24	0.29154273	0.9	0.793	1.42E-19	5	Srp14
Fmc1	8.42E-24	0.24198089	0.461	0.263	1.56E-19	5	Fmc1
Oaz1	1.20E-23	0.21331459	0.998	0.985	2.23E-19	5	Oaz1
Rps111	1.90E-23	0.20334442	1	0.995	3.54E-19	5	Rps11
Ebp1	2.00E-23	0.2943786	0.694	0.5	3.72E-19	5	Ebp
Mcfd2	2.28E-23	0.22077434	0.38	0.198	4.24E-19	5	Mcfd2
Aimp1	2.29E-23	0.3202938	0.9	0.779	4.26E-19	5	Aimp1
Naca2	2.68E-23	0.23024081	0.998	0.979	4.98E-19	5	Naca
Arl1	2.79E-23	0.2816693	0.731	0.574	5.18E-19	5	Arl1

Sec31a	3.59E-23	0.28114176	0.582	0.395	6.68E-19	5	Sec31a
Gng102	7.33E-23	0.33372449	0.982	0.92	1.36E-18	5	Gng10
Prelid1	9.09E-23	0.33125372	0.922	0.835	1.69E-18	5	Prelid1
Atp5o	1.34E-22	0.28519152	0.941	0.846	2.49E-18	5	Atp5o
Scfd1	1.64E-22	0.26091457	0.567	0.363	3.05E-18	5	Scfd1
Nus1	1.64E-22	0.28509482	0.531	0.34	3.05E-18	5	Nus1
Xbp1	1.93E-22	0.31202339	0.861	0.735	3.58E-18	5	Xbp1
Ndufs51	2.59E-22	0.31636013	0.871	0.783	4.81E-18	5	Ndufs5
Tvp23b	2.60E-22	0.23812418	0.404	0.222	4.83E-18	5	Tvp23b
Tmem248	3.27E-22	0.26519328	0.539	0.353	6.08E-18	5	Tmem248
Eif1a1	3.71E-22	0.3402437	0.894	0.78	6.89E-18	5	Eif1a
Polr2i	7.88E-22	0.30000876	0.682	0.515	1.46E-17	5	Polr2i
Rpl43	8.69E-22	0.22722851	0.992	0.975	1.61E-17	5	Rpl4
Pnn1	1.07E-21	0.31713211	0.708	0.525	1.98E-17	5	Pnn
Mrps18b	1.07E-21	0.26587493	0.561	0.371	1.99E-17	5	Mrps18b
Cox4i1	1.33E-21	0.22309022	0.99	0.972	2.48E-17	5	Cox4i1
Calm3	1.53E-21	0.34310071	0.871	0.793	2.84E-17	5	Calm3
Copa	1.58E-21	0.31242012	0.724	0.567	2.94E-17	5	Copa
Tars	1.72E-21	0.27868722	0.478	0.296	3.20E-17	5	Tars
Mmadhc	1.92E-21	0.21035322	0.433	0.246	3.56E-17	5	Mmadhc
Magoh1	2.55E-21	0.30534389	0.812	0.679	4.74E-17	5	Magoh
Hmbs	2.73E-21	0.20126718	0.31	0.151	5.08E-17	5	Hmbs
Ndufa121	3.58E-21	0.29798285	0.814	0.663	6.66E-17	5	Ndufa12

Tmco1	4.73E-21	0.2814651	0.839	0.695	8.80E-17	5	Tmco1
Ibtk	4.85E-21	0.20391888	0.337	0.168	9.01E-17	5	Ibtk
Sf3b61	4.97E-21	0.29887887	0.916	0.809	9.23E-17	5	Sf3b6
Magt1	5.04E-21	0.28451646	0.769	0.615	9.36E-17	5	Magt1
Itm2c	7.30E-21	0.2941889	0.924	0.857	1.36E-16	5	Itm2c
Anapc16	8.04E-21	0.2799764	0.641	0.459	1.49E-16	5	Anapc16
Sec22b	8.66E-21	0.23383426	0.459	0.274	1.61E-16	5	Sec22b
Emg1	9.80E-21	0.29507766	0.776	0.64	1.82E-16	5	Emg1
Uqcrc1	1.04E-20	0.26567453	0.824	0.66	1.92E-16	5	Uqcrc1
Yipf5	1.07E-20	0.21138122	0.547	0.345	1.98E-16	5	Yipf5
Rpl22l12	1.15E-20	0.33928284	0.976	0.916	2.14E-16	5	Rpl22l1
Sep-11	1.34E-20	0.21853797	0.51	0.313	2.49E-16	5	Sep-11
Snrpd22	1.52E-20	0.27895922	0.929	0.85	2.82E-16	5	Snrpd2
Tor1a	1.83E-20	0.27072362	0.835	0.71	3.41E-16	5	Tor1a
Psmb81	1.91E-20	0.2384714	1	0.985	3.55E-16	5	Psmb8
Srp68	2.36E-20	0.25489939	0.424	0.247	4.39E-16	5	Srp68
Snrpd32	4.00E-20	0.28135324	0.888	0.779	7.44E-16	5	Snrpd3
Tomm62	6.41E-20	0.29913401	0.939	0.865	1.19E-15	5	Tomm6
Set2	7.14E-20	0.28500767	0.973	0.93	1.33E-15	5	Set
Timm17a	1.14E-19	0.24828151	0.627	0.453	2.12E-15	5	Timm17a
Ybx12	1.33E-19	0.24492974	0.984	0.96	2.47E-15	5	Ybx1
Hist1h2ap1	1.64E-19	0.34651227	0.51	0.331	3.05E-15	5	Hist1h2ap
Abracl1	2.07E-19	0.26811699	0.975	0.89	3.84E-15	5	Abracl

Psma72	2.52E-19	0.25082107	0.99	0.948	4.69E-15	5	Psma7
Spp1	4.25E-19	0.43259415	0.514	0.327	7.90E-15	5	Spp1
Ddx50	4.47E-19	0.27916934	0.755	0.61	8.30E-15	5	Ddx50
Atp5l	4.88E-19	0.20828526	0.984	0.957	9.06E-15	5	Atp5l
Aars	5.24E-19	0.20541871	0.351	0.191	9.73E-15	5	Aars
Calu	6.36E-19	0.20225056	0.404	0.23	1.18E-14	5	Calu
Psmb1	7.43E-19	0.23598071	0.971	0.925	1.38E-14	5	Psmb1
Sar1a	9.96E-19	0.24100868	0.751	0.593	1.85E-14	5	Sar1a
Nktr	1.16E-18	0.27335739	0.818	0.687	2.15E-14	5	Nktr
Srsf21	1.21E-18	0.28954405	0.957	0.903	2.25E-14	5	Srsf2
Tbrg1	1.27E-18	0.28031925	0.841	0.724	2.36E-14	5	Tbrg1
Ost4	1.32E-18	0.28977816	0.935	0.871	2.46E-14	5	Ost4
H2-M3	1.57E-18	0.27952467	0.784	0.618	2.92E-14	5	H2-M3
Anp32b1	1.59E-18	0.25288543	0.951	0.909	2.95E-14	5	Anp32b
Ndufc11	1.71E-18	0.28396499	0.875	0.787	3.17E-14	5	Ndufc1
Ndufv31	1.81E-18	0.27633621	0.837	0.717	3.36E-14	5	Ndufv3
Psmd6	1.92E-18	0.26784802	0.753	0.596	3.57E-14	5	Psmd6
Arcn1	2.33E-18	0.2284812	0.659	0.485	4.34E-14	5	Arcn1
Tmsb102	3.03E-18	0.26279466	0.998	0.993	5.63E-14	5	Tmsb10
Psma11	3.46E-18	0.27958479	0.896	0.822	6.43E-14	5	Psma1
Ppp1ca	4.16E-18	0.24472842	0.976	0.932	7.73E-14	5	Ppp1ca
Ndufa14	5.30E-18	0.25515769	0.941	0.872	9.86E-14	5	Ndufa1
Trappc4	5.64E-18	0.27359766	0.89	0.785	1.05E-13	5	Trappc4

Morf4l21	6.34E-18	0.26077061	0.72	0.576	1.18E-13	5	Morf4l2
Aldh9a1	6.41E-18	0.27403171	0.614	0.445	1.19E-13	5	Aldh9a1
Mrps12	6.98E-18	0.24970678	0.649	0.49	1.30E-13	5	Mrps12
Clptm1l	7.11E-18	0.24943283	0.643	0.471	1.32E-13	5	Clptm1l
Nol71	7.40E-18	0.25619205	0.927	0.855	1.37E-13	5	Nol7
Ap2m1	7.49E-18	0.24809052	0.757	0.605	1.39E-13	5	Ap2m1
Pin1	7.50E-18	0.27060804	0.696	0.548	1.39E-13	5	Pin1
Isyna1	8.36E-18	0.29492159	0.622	0.467	1.55E-13	5	Isyna1
Eef1g1	8.66E-18	0.22532863	0.969	0.935	1.61E-13	5	Eef1g
Eif1ax	1.07E-17	0.26339316	0.692	0.531	1.98E-13	5	Eif1ax
Ppp1r14b	1.28E-17	0.30446355	0.753	0.625	2.38E-13	5	Ppp1r14b
Akr1a1	1.29E-17	0.20753109	0.969	0.922	2.39E-13	5	Akr1a1
Lars	1.45E-17	0.3075821	0.586	0.44	2.69E-13	5	Lars
Ssbp1	1.54E-17	0.23623401	0.661	0.48	2.87E-13	5	Ssbp1
Ndufa8	1.59E-17	0.24513417	0.837	0.686	2.95E-13	5	Ndufa8
Ly6e2	1.83E-17	0.2951223	0.904	0.78	3.40E-13	5	Ly6e
Cox6c	1.85E-17	0.21562866	0.996	0.964	3.43E-13	5	Cox6c
Ran2	2.07E-17	0.28173483	0.933	0.875	3.85E-13	5	Ran
Cct8	2.85E-17	0.23840163	0.937	0.861	5.29E-13	5	Cct8
Golgb1	3.57E-17	0.29778352	0.612	0.443	6.64E-13	5	Golgb1
Ndufb61	3.88E-17	0.26538808	0.792	0.656	7.20E-13	5	Ndufb6
Lsm72	4.07E-17	0.25348617	0.635	0.477	7.56E-13	5	Lsm7
Smim14	4.83E-17	0.27210467	0.81	0.688	8.97E-13	5	Smim14

Ndufb91	6.29E-17	0.27876211	0.937	0.856	1.17E-12	5	Ndufb9
Tmed3	6.37E-17	0.26531605	0.778	0.645	1.18E-12	5	Tmed3
Srpr	6.41E-17	0.2559239	0.686	0.528	1.19E-12	5	Srpr
Tomm40	6.46E-17	0.21203362	0.633	0.461	1.20E-12	5	Tomm40
Psmb22	7.06E-17	0.25945886	0.924	0.836	1.31E-12	5	Psmb2
Reep51	8.04E-17	0.2388777	0.975	0.934	1.49E-12	5	Reep5
Dap	8.29E-17	0.24317718	0.641	0.47	1.54E-12	5	Dap
Copb1	8.85E-17	0.26594159	0.665	0.519	1.64E-12	5	Copb1
Mpc11	1.00E-16	0.2915741	0.875	0.772	1.87E-12	5	Mpc1
Fubp1	1.49E-16	0.24124355	0.68	0.522	2.77E-12	5	Fubp1
Sem1	1.51E-16	0.20664178	0.992	0.966	2.80E-12	5	Sem1
Kdelr1	1.67E-16	0.2324996	0.633	0.472	3.10E-12	5	Kdelr1
Vapa1	1.69E-16	0.25450378	0.849	0.781	3.14E-12	5	Vapa
Eif3i2	2.20E-16	0.25176117	0.922	0.839	4.09E-12	5	Eif3i
Cox6b11	2.27E-16	0.25116052	0.98	0.932	4.21E-12	5	Cox6b1
Eif3c	2.30E-16	0.25871178	0.933	0.856	4.27E-12	5	Eif3c
Park72	2.31E-16	0.24297612	0.949	0.883	4.29E-12	5	Park7
Ubxn4	2.74E-16	0.24679926	0.814	0.685	5.10E-12	5	Ubxn4
Pfdn1	3.19E-16	0.2377667	0.682	0.513	5.93E-12	5	Pfdn1
Atp5md1	3.86E-16	0.2398785	0.965	0.936	7.17E-12	5	Atp5md
Rwdd11	4.53E-16	0.25416028	0.853	0.728	8.42E-12	5	Rwdd1
Anp32e	4.98E-16	0.2684535	0.776	0.648	9.25E-12	5	Anp32e
Hras	5.28E-16	0.23129116	0.569	0.426	9.81E-12	5	Hras

Ufsp2	5.46E-16	0.24831842	0.571	0.407	1.01E-11	5	Ufsp2
Tmem167	5.85E-16	0.25743501	0.82	0.735	1.09E-11	5	Tmem167
Ndufab11	6.69E-16	0.24225293	0.857	0.746	1.24E-11	5	Ndufab1
Ndufa3	7.11E-16	0.23962795	0.953	0.908	1.32E-11	5	Ndufa3
Rars	7.38E-16	0.21050479	0.573	0.409	1.37E-11	5	Rars
Pfdn6	8.38E-16	0.25603594	0.702	0.561	1.56E-11	5	Pfdn6
Sec63	8.96E-16	0.20596117	0.578	0.407	1.67E-11	5	Sec63
Kcnq1ot1	9.69E-16	0.28018877	0.445	0.285	1.80E-11	5	Kcnq1ot1
Wdr18	1.19E-15	0.20458217	0.508	0.34	2.22E-11	5	Wdr18
Mrpl51	1.31E-15	0.22516957	0.659	0.499	2.44E-11	5	Mrpl51
Ndufs61	1.39E-15	0.22823406	0.871	0.755	2.58E-11	5	Ndufs6
Uqcr111	1.40E-15	0.23515679	0.939	0.883	2.60E-11	5	Uqcr11
Idh22	1.45E-15	0.20334342	0.382	0.23	2.69E-11	5	Idh2
Hist1h4d1	1.64E-15	0.22389248	0.449	0.284	3.04E-11	5	Hist1h4d
Gars	2.03E-15	0.28203762	0.592	0.451	3.77E-11	5	Gars
Odc1	2.34E-15	0.21844485	0.845	0.701	4.35E-11	5	Odc1
Atp6v0d1	2.55E-15	0.26012438	0.782	0.662	4.74E-11	5	Atp6v0d1
Taldo11	2.56E-15	0.22576198	0.994	0.967	4.75E-11	5	Taldo1
Cd91	2.77E-15	0.28382558	0.951	0.895	5.14E-11	5	Cd9
Edem2	3.81E-15	0.22238842	0.469	0.312	7.09E-11	5	Edem2
Rpf2	4.08E-15	0.23053759	0.612	0.457	7.58E-11	5	Rpf2
Psmb7	4.17E-15	0.26119289	0.849	0.745	7.75E-11	5	Psmb7
Dynll11	4.98E-15	0.27884772	0.967	0.92	9.26E-11	5	Dynll1

Ywhaq	5.14E-15	0.21578331	0.898	0.806	9.55E-11	5	Ywhaq
Psmc5	5.96E-15	0.23412874	0.78	0.674	1.11E-10	5	Psmc5
Prmt12	6.65E-15	0.23572306	0.775	0.647	1.24E-10	5	Prmt1
Tm9sf3	7.09E-15	0.23669825	0.798	0.698	1.32E-10	5	Tm9sf3
Pts1	7.16E-15	0.20084006	0.653	0.487	1.33E-10	5	Pts
Snrpc	7.31E-15	0.22989571	0.782	0.663	1.36E-10	5	Snrpc
Mar-21	9.32E-15	0.21681714	0.696	0.544	1.73E-10	5	Mar-02
Tomm22	9.48E-15	0.2250453	0.9	0.8	1.76E-10	5	Tomm22
Pno1	1.05E-14	0.21132916	0.447	0.297	1.95E-10	5	Pno1
Cebpb1	1.17E-14	0.29658517	0.52	0.372	2.17E-10	5	Cebpb
Tomm5	1.23E-14	0.25435423	0.837	0.715	2.29E-10	5	Tomm5
Atp5c11	1.33E-14	0.21628413	0.976	0.928	2.48E-10	5	Atp5c1
Cwc15	2.14E-14	0.25068426	0.859	0.761	3.98E-10	5	Cwc15
Psmb4	3.29E-14	0.22080447	0.955	0.91	6.12E-10	5	Psmb4
Yif1b	3.32E-14	0.20301244	0.618	0.465	6.16E-10	5	Yif1b
Hmgb2	3.83E-14	0.56954039	0.924	0.868	7.11E-10	5	Hmgb2
Atp5j21	3.89E-14	0.22959074	0.978	0.937	7.24E-10	5	Atp5j2
Spcs1	4.27E-14	0.23869568	0.784	0.683	7.93E-10	5	Spcs1
Zranb2	4.56E-14	0.21359138	0.627	0.465	8.48E-10	5	Zranb2
Etfb1	5.09E-14	0.21728397	0.824	0.697	9.46E-10	5	Etfb
Mrpl201	5.48E-14	0.24402306	0.82	0.715	1.02E-09	5	Mrpl20
Eny2	5.97E-14	0.22261748	0.68	0.542	1.11E-09	5	Eny2
Sub11	6.79E-14	0.21598665	0.998	0.983	1.26E-09	5	Sub1

Psma21	8.42E-14	0.2261972	0.953	0.881	1.56E-09	5	Psma2
H2afv	9.19E-14	0.34395153	0.675	0.564	1.71E-09	5	H2afv
Mrps151	9.79E-14	0.25253693	0.741	0.608	1.82E-09	5	Mrps15
Ndufb7	1.45E-13	0.23343282	0.955	0.891	2.70E-09	5	Ndufb7
Nr2c2ap	1.59E-13	0.20436717	0.602	0.458	2.96E-09	5	Nr2c2ap
Atp5h2	1.69E-13	0.20186836	0.982	0.958	3.14E-09	5	Atp5h
Cct5	1.88E-13	0.21239998	0.92	0.862	3.48E-09	5	Cct5
Gmps	1.96E-13	0.21283543	0.522	0.385	3.64E-09	5	Gmps
Dynlrb1	2.59E-13	0.21849811	0.845	0.736	4.81E-09	5	Dynlrb1
BC031181	2.86E-13	0.21670221	0.81	0.706	5.32E-09	5	BC031181
Atp5g3	3.22E-13	0.22464032	0.916	0.874	5.98E-09	5	Atp5g3
Csnk2b	3.57E-13	0.20467739	0.867	0.771	6.63E-09	5	Csnk2b
Cct7	3.78E-13	0.21817286	0.88	0.818	7.02E-09	5	Cct7
Ciao2a1	4.42E-13	0.21960876	0.686	0.564	8.22E-09	5	Ciao2a
Akr1b3	4.45E-13	0.22896073	0.653	0.519	8.27E-09	5	Akr1b3
Ckb2	4.62E-13	0.20225716	1	0.963	8.59E-09	5	Ckb
Dph3	4.71E-13	0.21392346	0.737	0.587	8.76E-09	5	Dph3
Rbx11	5.37E-13	0.20178723	0.953	0.9	9.97E-09	5	Rbx1
Lamtor2	5.43E-13	0.22806957	0.875	0.796	1.01E-08	5	Lamtor2
Rnaseh2c	5.51E-13	0.24435749	0.465	0.332	1.02E-08	5	Rnaseh2c
Nedd81	5.52E-13	0.20363636	0.984	0.936	1.03E-08	5	Nedd8
Atf4	6.15E-13	0.20165119	0.927	0.894	1.14E-08	5	Atf4
Idh3g	6.43E-13	0.23038323	0.669	0.526	1.19E-08	5	Idh3g

Sec13	7.86E-13	0.22742457	0.59	0.453	1.46E-08	5	Sec13
Tmem14c2	8.93E-13	0.21548195	0.91	0.831	1.66E-08	5	Tmem14c
Serbp11	9.02E-13	0.21012871	0.98	0.948	1.68E-08	5	Serbp1
Emc7	9.52E-13	0.22364722	0.749	0.613	1.77E-08	5	Emc7
Hnrnpa31	9.68E-13	0.25496146	0.982	0.95	1.80E-08	5	Hnrnpa3
Atp5mpl1	1.07E-12	0.20760264	0.943	0.904	1.99E-08	5	Atp5mpl
Eprs	1.24E-12	0.25208758	0.716	0.609	2.31E-08	5	Eprs
Ndufs3	1.40E-12	0.20062816	0.655	0.502	2.60E-08	5	Ndufs3
Psmd81	1.63E-12	0.20233003	0.91	0.826	3.02E-08	5	Psmd8
Hp1bp3	2.07E-12	0.21268284	0.618	0.467	3.84E-08	5	Hp1bp3
Ubfd1	2.10E-12	0.22005207	0.631	0.482	3.91E-08	5	Ubfd1
Ndufb102	2.63E-12	0.20435607	0.908	0.829	4.88E-08	5	Ndufb10
Snrpa11	2.87E-12	0.21037541	0.712	0.581	5.33E-08	5	Snrpa1
Psmc2	3.27E-12	0.21864866	0.735	0.626	6.07E-08	5	Psmc2
Mrpl30	3.29E-12	0.21582545	0.843	0.753	6.12E-08	5	Mrpl30
Churc1	5.48E-12	0.20635257	0.725	0.587	1.02E-07	5	Churc1
Cfp1	7.37E-12	0.20428555	0.741	0.593	1.37E-07	5	Cfp
Pdia5	7.38E-12	0.21804478	0.455	0.326	1.37E-07	5	Pdia5
Commd1	8.97E-12	0.22974777	0.72	0.617	1.67E-07	5	Commd1
Lsm5	9.36E-12	0.28449463	0.616	0.503	1.74E-07	5	Lsm5
Rabac1	9.57E-12	0.23361001	0.802	0.719	1.78E-07	5	Rabac1
Plac82	1.26E-11	0.25044954	0.849	0.73	2.35E-07	5	Plac8
Mrpl54	1.42E-11	0.20183896	0.824	0.734	2.63E-07	5	Mrpl54

Acbd3	2.99E-11	0.20754402	0.575	0.435	5.55E-07	5	Acbd3
Ifi302	4.68E-11	0.21116777	0.973	0.949	8.69E-07	5	Ifi30
Pdcd51	4.76E-11	0.22180122	0.786	0.69	8.84E-07	5	Pdcd5
Eif3m	5.73E-11	0.21306291	0.943	0.866	1.06E-06	5	Eif3m
Nop581	5.74E-11	0.21464989	0.82	0.723	1.07E-06	5	Nop58
Guk1	5.83E-11	0.22441661	0.604	0.482	1.08E-06	5	Guk1
Timm231	6.00E-11	0.20923462	0.825	0.725	1.12E-06	5	Timm23
Eif2s1	1.54E-10	0.20458526	0.833	0.755	2.87E-06	5	Eif2s1
Gadd45g1	2.15E-10	0.30058485	0.475	0.349	3.99E-06	5	Gadd45g
Cox16	2.37E-10	0.20252874	0.653	0.537	4.41E-06	5	Cox16
Ndufb21	6.25E-10	0.20466287	0.851	0.769	1.16E-05	5	Ndufb2
Hmgn1	6.63E-10	0.23973542	0.804	0.704	1.23E-05	5	Hmgn1
Cdv3	1.18E-09	0.20182872	0.796	0.708	2.20E-05	5	Cdv3
Dctn3	1.23E-09	0.20631634	0.89	0.823	2.28E-05	5	Dctn3
Snhg11	1.03E-08	0.22764197	0.892	0.824	0.00019053	5	Snhg1
Ctsc1	1.84E-08	0.2195609	0.876	0.789	0.00034095	5	Ctsc
B930036N10Rik	8.56E-08	0.21534661	0.41	0.301	0.00159044	5	B930036N10Rik
Sgk11	4.04E-06	0.22716953	0.559	0.474	0.07510674	5	Sgk1
Plet11	0.00044755	0.23909044	0.214	0.156	1	5	Plet1
Psap	9.76E-204	1.03783785	1	0.993	1.81E-199	6	Psap
Wdfy4	5.28E-151	1.09485293	0.988	0.928	9.82E-147	6	Wdfy4
Mpeg1	1.35E-147	0.97321265	1	0.971	2.51E-143	6	Mpeg1
Dock10	5.11E-134	0.99821168	0.984	0.93	9.49E-130	6	Dock10

Gm42418	2.32E-123	1.96326948	1	1	4.31E-119	6	Gm42418
Naaa2	4.90E-112	0.83951272	0.998	0.954	9.11E-108	6	Naaa
Laptm5	2.83E-110	0.76312579	0.98	0.98	5.26E-106	6	Laptm5
Ddx3x	6.02E-109	1.13827387	0.871	0.735	1.12E-104	6	Ddx3x
Iqgap1	1.93E-107	0.91636112	0.982	0.965	3.58E-103	6	Iqgap1
Pfkp1	6.93E-106	1.05818976	0.962	0.88	1.29E-101	6	Pfkp
Dennd4a	4.14E-105	1.10593937	0.954	0.899	7.70E-101	6	Dennd4a
Lars2	2.06E-102	1.0586071	0.9	0.814	3.83E-98	6	Lars2
Plekho2	8.80E-100	0.89367856	0.924	0.857	1.64E-95	6	Plekho2
Ptprc	1.90E-98	0.82599274	0.986	0.958	3.53E-94	6	Ptprc
Flt3	6.04E-98	1.03139877	0.886	0.789	1.12E-93	6	Flt3
Ahnak1	8.86E-98	1.04171104	0.984	0.946	1.65E-93	6	Ahnak
Nr4a11	2.10E-97	0.88072229	0.976	0.946	3.90E-93	6	Nr4a1
Picalm	5.60E-97	0.94884788	0.964	0.927	1.04E-92	6	Picalm
Kdm6b	6.10E-96	0.90098278	0.938	0.842	1.13E-91	6	Kdm6b
Tob21	5.71E-92	1.15277954	0.882	0.739	1.06E-87	6	Tob2
Sep-33	4.47E-90	0.92985258	0.926	0.721	8.30E-86	6	Sep-03
Mbnl1	2.83E-89	0.77162855	0.98	0.96	5.27E-85	6	Mbnl1
Ccnl11	3.30E-89	0.84325663	0.986	0.948	6.12E-85	6	Ccnl1
Atp1a1	3.40E-86	0.96637227	0.916	0.852	6.32E-82	6	Atp1a1
Fosb1	2.06E-85	1.0424418	0.97	0.911	3.83E-81	6	Fosb
Fnbp1	3.70E-84	0.7277768	0.972	0.954	6.88E-80	6	Fnbp1
Pabpc11	7.09E-83	0.68546981	0.982	0.977	1.32E-78	6	Pabpc1

Sdc3	5.31E-82	0.90969432	0.839	0.686	9.86E-78	6	Sdc3
Pip4k2a	6.57E-82	0.85159945	0.908	0.86	1.22E-77	6	Pip4k2a
Gm2a2	1.46E-78	0.68085849	1	0.966	2.70E-74	6	Gm2a
Wnk1	3.83E-75	1.09366321	0.956	0.925	7.12E-71	6	Wnk1
Ece1	1.09E-74	0.77018116	0.896	0.798	2.03E-70	6	Ece1
Nr4a21	1.28E-73	0.89725707	0.948	0.89	2.38E-69	6	Nr4a2
Pim1	6.12E-73	0.69751834	0.988	0.973	1.14E-68	6	Pim1
Synj1	3.86E-72	0.90118	0.835	0.705	7.16E-68	6	Synj1
Kctd121	5.90E-72	0.80969108	0.952	0.936	1.10E-67	6	Kctd12
Egr31	7.89E-72	1.05481631	0.871	0.679	1.47E-67	6	Egr3
BC005537	6.90E-71	0.92899088	0.823	0.7	1.28E-66	6	BC005537
Sgms1	9.03E-71	1.05777953	0.739	0.511	1.68E-66	6	Sgms1
Rab7b	7.79E-69	0.76278253	0.904	0.887	1.45E-64	6	Rab7b
Etv3	8.14E-69	0.78913313	0.882	0.778	1.51E-64	6	Etv3
Cd24a1	5.91E-68	0.68273759	0.956	0.943	1.10E-63	6	Cd24a
Plek1	1.04E-67	0.77043608	0.966	0.943	1.94E-63	6	Plek
Nr4a3	1.36E-67	1.00492761	0.859	0.698	2.54E-63	6	Nr4a3
Naga3	5.69E-67	0.68975387	0.966	0.917	1.06E-62	6	Naga
Spag9	1.25E-66	0.77980328	0.964	0.962	2.32E-62	6	Spag9
Cbfa2t32	1.38E-66	0.74002923	0.958	0.897	2.56E-62	6	Cbfa2t3
Dusp12	6.44E-66	1.15390816	0.954	0.879	1.20E-61	6	Dusp1
Camk1d	8.36E-66	0.75507474	0.902	0.817	1.55E-61	6	Camk1d
Itpr1	5.40E-65	0.86736261	0.819	0.665	1.00E-60	6	Itpr1

Marcks	7.46E-65	0.69296648	0.952	0.927	1.39E-60	6	Marcks
Fus	8.36E-65	0.72569621	0.926	0.907	1.55E-60	6	Fus
Gls	1.34E-64	0.89463589	0.849	0.742	2.50E-60	6	Gls
Sfpq	4.32E-64	0.70022368	0.888	0.865	8.03E-60	6	Sfpq
Purb	3.90E-63	0.76529776	0.783	0.623	7.25E-59	6	Purb
Neat11	1.99E-62	1.10429689	0.88	0.762	3.70E-58	6	Neat1
Itgal	9.14E-62	0.81437638	0.741	0.567	1.70E-57	6	Itgal
Pik3cb1	1.58E-61	0.77923349	0.793	0.643	2.94E-57	6	Pik3cb
Trerf1	1.08E-60	0.81951763	0.787	0.627	2.01E-56	6	Trerf1
Inpp5d	1.36E-60	0.69679063	0.886	0.819	2.53E-56	6	Inpp5d
Slc38a1	6.92E-60	1.06363299	0.855	0.751	1.29E-55	6	Slc38a1
Atp2b1	1.25E-59	1.09350989	0.894	0.837	2.33E-55	6	Atp2b1
Tapbp	1.20E-58	0.63481548	0.928	0.881	2.23E-54	6	Tapbp
Kdm7a	5.11E-58	0.78301627	0.843	0.721	9.50E-54	6	Kdm7a
Sep-09	6.04E-58	0.83461873	0.789	0.705	1.12E-53	6	Sep-09
Rab431	2.01E-57	0.63098288	0.946	0.906	3.73E-53	6	Rab43
Trib1	3.60E-56	0.85022429	0.851	0.781	6.69E-52	6	Trib1
Egr11	7.92E-56	1.04124753	0.918	0.777	1.47E-51	6	Egr1
Dennd1b	2.27E-55	0.73584723	0.753	0.589	4.21E-51	6	Dennd1b
Hnrnpa0	2.94E-55	0.74577558	0.771	0.657	5.47E-51	6	Hnrnpa0
Jmjd1c	3.28E-55	0.85940668	0.839	0.754	6.09E-51	6	Jmjd1c
Klf62	5.91E-55	0.80502833	0.964	0.924	1.10E-50	6	Klf6
Ptp4a2	1.98E-54	0.70255148	0.876	0.855	3.68E-50	6	Ptp4a2

Fgd22	2.09E-54	0.67058806	0.886	0.833	3.88E-50	6	Fgd2
Hnrnph1	2.21E-54	0.76607159	0.745	0.593	4.10E-50	6	Hnrnph1
Csrnp1	1.45E-53	0.79733118	0.835	0.767	2.69E-49	6	Csrnp1
Pmaip11	2.08E-53	0.78714283	0.932	0.853	3.86E-49	6	Pmaip1
Ywhaz	7.48E-52	0.48656119	0.976	0.978	1.39E-47	6	Ywhaz
Usp7	7.78E-52	0.67417008	0.624	0.413	1.45E-47	6	Usp7
Fgfr13	1.79E-51	0.78437216	0.713	0.487	3.32E-47	6	Fgfr1
Ddx17	7.46E-49	0.65326602	0.763	0.623	1.39E-44	6	Ddx17
Myo9a	7.72E-49	0.72035006	0.719	0.553	1.43E-44	6	Myo9a
Tent5c2	1.07E-48	0.76371837	0.837	0.781	1.99E-44	6	Tent5c
Lrrk2	3.46E-48	0.75361182	0.749	0.61	6.43E-44	6	Lrrk2
Ranbp2	1.10E-47	0.7255078	0.841	0.75	2.05E-43	6	Ranbp2
Rab11fip11	1.27E-46	0.68465437	0.789	0.677	2.37E-42	6	Rab11fip1
Notch2	5.51E-46	0.71921429	0.761	0.651	1.02E-41	6	Notch2
Elmsan1	4.12E-45	0.87412001	0.639	0.442	7.65E-41	6	Elmsan1
Xcr11	8.24E-45	0.71393747	0.861	0.725	1.53E-40	6	Xcr1
Mob1a	1.34E-44	0.56497342	0.831	0.787	2.49E-40	6	Mob1a
Csde1	1.66E-44	0.58756819	0.835	0.819	3.09E-40	6	Csde1
Pbx1	1.87E-44	0.68245554	0.751	0.63	3.47E-40	6	Pbx1
Rbpj	2.05E-44	0.57544327	0.958	0.961	3.80E-40	6	Rbpj
Celf2	3.72E-44	0.5737227	0.896	0.859	6.91E-40	6	Celf2
Gpd2	4.15E-44	0.8057558	0.745	0.633	7.70E-40	6	Gpd2
Cdk14	4.78E-44	0.69893844	0.763	0.657	8.89E-40	6	Cdk14

Map3k1	6.43E-44	0.64329239	0.711	0.545	1.19E-39	6	Map3k1
Gpcpd11	1.32E-43	0.83128914	0.803	0.703	2.45E-39	6	Gpcpd1
Anpep1	4.13E-43	0.61769198	0.747	0.615	7.67E-39	6	Anpep
Slk	6.96E-43	0.63793868	0.845	0.782	1.29E-38	6	Slk
Cadm12	1.67E-42	0.7362567	0.655	0.432	3.10E-38	6	Cadm1
Eif3a	3.30E-40	0.53280805	0.914	0.889	6.13E-36	6	Eif3a
Lcp1	4.34E-40	0.50425179	0.97	0.969	8.06E-36	6	Lcp1
Zfp36l21	4.81E-40	0.70684145	0.932	0.909	8.94E-36	6	Zfp36l2
Zfp361	4.99E-40	0.80092793	0.94	0.956	9.27E-36	6	Zfp36
Pum2	8.71E-40	0.61778521	0.711	0.607	1.62E-35	6	Pum2
Stat3	1.08E-39	0.62988322	0.789	0.725	2.01E-35	6	Stat3
Tbc1d9	3.68E-39	0.64762116	0.717	0.61	6.84E-35	6	Tbc1d9
Ccdc88b	6.31E-39	0.57530049	0.552	0.356	1.17E-34	6	Ccdc88b
Btla1	6.69E-39	0.6220073	0.797	0.699	1.24E-34	6	Btla
Baz1a1	1.06E-38	0.61092211	0.888	0.907	1.97E-34	6	Baz1a
Arhgap30	1.14E-38	0.56722066	0.845	0.816	2.12E-34	6	Arhgap30
Fchsd2	1.38E-38	0.65092406	0.753	0.62	2.56E-34	6	Fchsd2
Sod1	5.81E-38	0.73616849	0.801	0.809	1.08E-33	6	Sod1
Actr2	7.67E-38	0.50383397	0.95	0.95	1.42E-33	6	Actr2
Arap1	9.43E-38	0.58998332	0.572	0.383	1.75E-33	6	Arap1
Ago2	1.88E-37	0.64709109	0.745	0.641	3.49E-33	6	Ago2
Eef2	1.92E-37	0.46216979	0.956	0.972	3.57E-33	6	Eef2
Sik11	3.46E-37	0.63310846	0.755	0.626	6.43E-33	6	Sik1

Trip12	3.75E-37	0.63591693	0.665	0.529	6.96E-33	6	Trip12
Usp9x	5.94E-37	0.61695181	0.624	0.48	1.10E-32	6	Usp9x
Jarid2	1.33E-36	0.62382148	0.813	0.753	2.47E-32	6	Jarid2
Wdr1	3.57E-36	0.53988166	0.859	0.837	6.63E-32	6	Wdr1
Itgae1	4.46E-36	0.67839744	0.773	0.608	8.29E-32	6	Itgae
Clk11	6.52E-36	0.65190117	0.908	0.902	1.21E-31	6	Clk1
Arhgdia	9.11E-35	0.48905389	0.93	0.956	1.69E-30	6	Arhgdia
Ppp1r15a1	9.95E-35	0.61540441	0.859	0.814	1.85E-30	6	Ppp1r15a
Tgfbi	1.55E-34	0.66235462	0.811	0.737	2.87E-30	6	Tgfbi
Atf41	1.78E-34	0.57521431	0.876	0.898	3.30E-30	6	Atf4
Itm2c1	2.34E-34	0.50746289	0.878	0.862	4.35E-30	6	Itm2c
Lbh	2.46E-34	0.70765885	0.687	0.567	4.57E-30	6	Lbh
Cd2ap	2.55E-34	0.53968245	0.867	0.842	4.74E-30	6	Cd2ap
Nrip1	3.05E-34	0.70998564	0.663	0.527	5.66E-30	6	Nrip1
Arsb1	3.77E-34	0.65061347	0.715	0.611	7.00E-30	6	Arsb
Rbm47	3.87E-34	0.54653696	0.558	0.38	7.19E-30	6	Rbm47
Parp8	4.22E-34	0.61906733	0.723	0.627	7.83E-30	6	Parp8
Grk2	5.43E-34	0.55185193	0.797	0.762	1.01E-29	6	Grk2
Nup210	5.62E-34	0.60587989	0.622	0.474	1.04E-29	6	Nup210
Erbin	1.24E-33	0.55637965	0.635	0.487	2.30E-29	6	Erbin
Dock8	1.71E-33	0.53180408	0.783	0.7	3.17E-29	6	Dock8
P2ry101	1.80E-33	0.67678563	0.831	0.838	3.35E-29	6	P2ry10
Smg1	2.93E-33	0.58288414	0.604	0.463	5.43E-29	6	Smg1

Atp8a1	3.71E-33	0.57132246	0.695	0.603	6.89E-29	6	Atp8a1
Fgl2	7.66E-33	0.51267856	0.876	0.833	1.42E-28	6	Fgl2
Ddx6	1.03E-32	0.51630731	0.797	0.753	1.92E-28	6	Ddx6
Dock2	1.51E-32	0.56816613	0.775	0.737	2.81E-28	6	Dock2
Rel	1.60E-32	0.50837578	0.928	0.893	2.97E-28	6	Rel
Tut7	3.77E-32	0.5772779	0.789	0.734	7.01E-28	6	Tut7
Ppp1r15b	4.73E-32	0.62317713	0.681	0.578	8.79E-28	6	Ppp1r15b
Ppp2ca	4.75E-32	0.48341377	0.89	0.894	8.83E-28	6	Ppp2ca
Csnk1a1	4.85E-32	0.51196543	0.853	0.845	9.02E-28	6	Csnk1a1
Lpcat1	5.51E-32	0.63200566	0.669	0.567	1.02E-27	6	Lpcat1
Qk	8.08E-32	0.61461129	0.669	0.572	1.50E-27	6	Qk
Olfm12	8.09E-32	0.52782426	0.898	0.841	1.50E-27	6	Olfm1
Runx1	1.15E-31	0.53359396	0.765	0.685	2.14E-27	6	Runx1
Srebf2	1.28E-31	0.52626719	0.821	0.779	2.38E-27	6	Srebf2
Eif4g2	2.92E-31	0.47842315	0.869	0.875	5.42E-27	6	Eif4g2
Hsp90aa11	4.12E-31	0.60460624	0.982	0.975	7.66E-27	6	Hsp90aa1
Cltc	4.18E-31	0.56011481	0.763	0.736	7.76E-27	6	Cltc
Atf6	5.49E-31	0.58976153	0.653	0.529	1.02E-26	6	Atf6
Atrx	6.23E-31	0.55455177	0.803	0.755	1.16E-26	6	Atrx
Wipf1	6.79E-31	0.58149355	0.687	0.586	1.26E-26	6	Wipf1
Cd441	7.02E-31	0.85377252	0.781	0.697	1.30E-26	6	Cd44
Ptbp3	1.53E-30	0.45824347	0.857	0.842	2.85E-26	6	Ptbp3
Kpna4	1.76E-30	0.61468816	0.829	0.825	3.26E-26	6	Kpna4

Efhd2	2.78E-30	0.43709928	0.948	0.955	5.17E-26	6	Efhd2
Htr7	4.44E-30	0.5285539	0.58	0.432	8.25E-26	6	Htr7
Myh9	4.52E-30	0.5583734	0.835	0.834	8.39E-26	6	Myh9
Alcam	5.49E-30	0.59158791	0.801	0.766	1.02E-25	6	Alcam
Pdcd6ip	1.20E-29	0.53446737	0.701	0.617	2.22E-25	6	Pdcd6ip
Clic4	1.68E-29	0.53019673	0.815	0.751	3.12E-25	6	Clic4
Tyk2	2.46E-29	0.56230823	0.624	0.497	4.57E-25	6	Tyk2
Hnrnpa2b1	4.26E-29	0.37657759	0.96	0.971	7.92E-25	6	Hnrnpa2b1
Akap13	4.39E-29	0.51321079	0.861	0.852	8.17E-25	6	Akap13
Huwe1	5.09E-29	0.5815107	0.699	0.617	9.46E-25	6	Huwe1
mt-Co3	5.40E-29	0.55702035	0.998	0.999	1.00E-24	6	mt-Co3
Wdr26	1.43E-28	0.52586375	0.695	0.617	2.65E-24	6	Wdr26
Eif4g3	2.41E-28	0.49804878	0.707	0.615	4.48E-24	6	Eif4g3
Tra2b1	4.34E-28	0.47637377	0.948	0.937	8.06E-24	6	Tra2b
Pik3cd	5.42E-28	0.54697789	0.602	0.484	1.01E-23	6	Pik3cd
Gm36723	9.69E-28	0.55160353	0.526	0.366	1.80E-23	6	Gm36723
Plbd1	1.53E-27	0.3771368	0.984	0.972	2.85E-23	6	Plbd1
Mar-11	1.64E-27	0.55711515	0.783	0.758	3.04E-23	6	Mar-01
Skil	1.80E-27	0.50212471	0.884	0.886	3.35E-23	6	Skil
Sf1	2.06E-27	0.51320752	0.737	0.684	3.83E-23	6	Sf1
Dync1h1	2.26E-27	0.52265144	0.629	0.525	4.20E-23	6	Dync1h1
Usp38	3.62E-27	0.46615737	0.448	0.282	6.72E-23	6	Usp38
Cbl	4.19E-27	0.51597599	0.751	0.711	7.79E-23	6	Cbl

Irf82	4.20E-27	0.37773763	0.99	0.977	7.80E-23	6	Irf8
Dpysl2	4.23E-27	0.52905627	0.695	0.628	7.86E-23	6	Dpysl2
Zfp36l11	4.29E-27	0.59264209	0.789	0.711	7.96E-23	6	Zfp36l1
Ciita2	4.71E-27	0.54685092	0.825	0.778	8.76E-23	6	Ciita
Tbc1d8	4.90E-27	0.55676127	0.727	0.664	9.10E-23	6	Tbc1d8
Myadm	5.05E-27	0.52430087	0.841	0.811	9.38E-23	6	Myadm
Ikzf1	6.33E-27	0.52717783	0.681	0.579	1.18E-22	6	Ikzf1
Tax1bp1	6.87E-27	0.42220448	0.924	0.921	1.28E-22	6	Tax1bp1
Hnrnpu	7.25E-27	0.45730578	0.869	0.874	1.35E-22	6	Hnrnpu
Lnpep	7.90E-27	0.51736746	0.649	0.545	1.47E-22	6	Lnpep
Dpy19l1	8.14E-27	0.54833255	0.608	0.49	1.51E-22	6	Dpy19l1
Macf11	9.48E-27	0.62221261	0.647	0.545	1.76E-22	6	Macf1
Cd83	1.08E-26	0.44580591	0.956	0.959	2.01E-22	6	Cd83
Ep300	1.08E-26	0.51200493	0.606	0.484	2.01E-22	6	Ep300
Hspa1b1	1.23E-26	1.09679887	0.661	0.514	2.29E-22	6	Hspa1b
Lyst	1.34E-26	0.53268464	0.719	0.656	2.48E-22	6	Lyst
5031439G07Rik	1.62E-26	0.53084762	0.723	0.641	3.00E-22	6	5031439G07Rik
Hcls1	1.66E-26	0.43608668	0.871	0.864	3.09E-22	6	Hcls1
Ptprj	1.81E-26	0.48343584	0.554	0.414	3.36E-22	6	Ptprj
Cyth4	2.22E-26	0.49923932	0.91	0.912	4.13E-22	6	Cyth4
Aff1	2.49E-26	0.64028882	0.669	0.596	4.63E-22	6	Aff1
Egr22	3.17E-26	0.76273924	0.562	0.388	5.90E-22	6	Egr2
Eif4b	3.51E-26	0.52489866	0.667	0.596	6.52E-22	6	Eif4b

Ankrd44	4.32E-26	0.50953687	0.709	0.637	8.02E-22	6	Ankrd44
Arfgef1	4.45E-26	0.48720768	0.57	0.439	8.27E-22	6	Arfgef1
Fam120a	4.67E-26	0.54970869	0.552	0.415	8.67E-22	6	Fam120a
Bcl6	6.61E-26	0.57615358	0.627	0.534	1.23E-21	6	Bcl6
Map4k4	7.68E-26	0.31976796	0.727	0.617	1.43E-21	6	Map4k4
Rell1	9.01E-26	0.58097647	0.534	0.39	1.67E-21	6	Rell1
Irs21	1.54E-25	0.5825527	0.502	0.353	2.86E-21	6	Irs2
Med13	2.27E-25	0.48797257	0.518	0.369	4.21E-21	6	Med13
Tiam1	2.93E-25	0.52960815	0.653	0.554	5.44E-21	6	Tiam1
Wsb11	3.10E-25	0.58620601	0.723	0.66	5.77E-21	6	Wsb1
Ubash3b	3.53E-25	0.51178417	0.717	0.632	6.56E-21	6	Ubash3b
Taok3	4.56E-25	0.51188226	0.759	0.731	8.48E-21	6	Taok3
Larp4b	5.36E-25	0.56241777	0.627	0.53	9.95E-21	6	Larp4b
Rock1	5.97E-25	0.47547503	0.837	0.827	1.11E-20	6	Rock1
Itgb1	6.50E-25	0.451604	0.892	0.906	1.21E-20	6	Itgb1
Gdi21	7.09E-25	0.35476055	0.964	0.975	1.32E-20	6	Gdi2
Rasgrp4	7.10E-25	0.516862	0.647	0.574	1.32E-20	6	Rasgrp4
Rgs21	9.10E-25	0.6159152	0.92	0.923	1.69E-20	6	Rgs2
Arhgap26	1.02E-24	0.49457739	0.562	0.437	1.89E-20	6	Arhgap26
Ppt12	1.21E-24	0.41058309	0.976	0.933	2.25E-20	6	Ppt1
Ppp1r9b	1.59E-24	0.43713955	0.458	0.311	2.95E-20	6	Ppp1r9b
Taok1	1.93E-24	0.49282355	0.512	0.375	3.58E-20	6	Taok1
St3gal53	5.65E-24	0.49710329	0.775	0.739	1.05E-19	6	St3gal5

Nop53	8.04E-24	0.45094697	0.765	0.761	1.49E-19	6	Nop53
Rfx7	8.18E-24	0.51015981	0.538	0.414	1.52E-19	6	Rfx7
Chuk	9.99E-24	0.54209733	0.574	0.469	1.86E-19	6	Chuk
Cass41	1.46E-23	0.52214273	0.482	0.329	2.71E-19	6	Cass4
Gpr132	2.78E-23	0.54328394	0.731	0.7	5.16E-19	6	Gpr132
Il6ra1	4.09E-23	0.52124182	0.582	0.471	7.60E-19	6	Il6ra
Tet2	4.11E-23	0.53778572	0.594	0.47	7.63E-19	6	Tet2
Pi4k2a1	4.56E-23	0.56903009	0.695	0.615	8.47E-19	6	Pi4k2a
Coro2a	4.63E-23	0.45226083	0.689	0.605	8.61E-19	6	Coro2a
Eif4a21	5.04E-23	0.47588534	0.761	0.742	9.36E-19	6	Eif4a2
Arih1	5.74E-23	0.50337254	0.669	0.613	1.07E-18	6	Arih1
Kdm5a	7.28E-23	0.53529519	0.594	0.505	1.35E-18	6	Kdm5a
Crybg1	1.32E-22	0.45384726	0.582	0.469	2.45E-18	6	Crybg1
Aff4	1.47E-22	0.54966691	0.598	0.499	2.73E-18	6	Aff4
Zyx	1.51E-22	0.37070932	0.916	0.941	2.81E-18	6	Zyx
Fcgr2b2	1.80E-22	0.45602035	0.753	0.667	3.35E-18	6	Fcgr2b
Chsy3	2.09E-22	0.38484415	0.277	0.137	3.88E-18	6	Chsy3
Anxa6	2.51E-22	0.48380512	0.807	0.827	4.66E-18	6	Anxa6
Diaph1	2.85E-22	0.47038187	0.763	0.723	5.29E-18	6	Diaph1
Hook3	2.86E-22	0.60552549	0.578	0.476	5.31E-18	6	Hook3
Prkar2a	2.92E-22	0.53436877	0.729	0.683	5.42E-18	6	Prkar2a
Zmiz1	3.36E-22	0.47010638	0.717	0.657	6.25E-18	6	Zmiz1
Prcp	3.40E-22	0.48769472	0.729	0.714	6.32E-18	6	Prcp

Srrm2	5.00E-22	0.4758135	0.94	0.953	9.29E-18	6	Srrm2
Tap1	7.56E-22	0.44090638	0.896	0.894	1.40E-17	6	Tap1
Klf42	9.14E-22	0.60563904	0.773	0.698	1.70E-17	6	Klf4
Brd21	9.21E-22	0.53132651	0.809	0.814	1.71E-17	6	Brd2
Plekha51	9.47E-22	0.51681545	0.6	0.5	1.76E-17	6	Plekha5
Eps81	9.49E-22	0.53140543	0.735	0.688	1.76E-17	6	Eps8
Trio	9.54E-22	0.4717681	0.57	0.447	1.77E-17	6	Trio
Gm26917	1.34E-21	0.61560264	0.466	0.322	2.49E-17	6	Gm26917
Fam168a	1.50E-21	0.43469773	0.438	0.301	2.78E-17	6	Fam168a
Fam129a	1.55E-21	0.39058539	0.703	0.63	2.88E-17	6	Fam129a
Rps15	1.73E-21	0.29635878	0.976	0.981	3.21E-17	6	Rps15
Eif51	2.36E-21	0.36870685	0.918	0.919	4.38E-17	6	Eif5
Peli1	2.36E-21	0.55988381	0.681	0.608	4.38E-17	6	Peli1
Arhgap171	3.95E-21	0.45025499	0.753	0.709	7.33E-17	6	Arhgap17
Arhgap18	4.70E-21	0.44662932	0.767	0.757	8.73E-17	6	Arhgap18
Vcp1	4.74E-21	0.4186358	0.813	0.832	8.80E-17	6	Vcp
Esyt2	4.90E-21	0.52519154	0.472	0.347	9.11E-17	6	Esyt2
Mapk14	5.03E-21	0.47455112	0.604	0.522	9.34E-17	6	Mapk14
Ddhd1	5.46E-21	0.43373203	0.44	0.3	1.01E-16	6	Ddhd1
Dazap2	5.70E-21	0.50570968	0.791	0.832	1.06E-16	6	Dazap2
Zbtb7a	9.31E-21	0.51450457	0.592	0.505	1.73E-16	6	Zbtb7a
Trpv2	1.15E-20	0.46563182	0.641	0.569	2.13E-16	6	Trpv2
mt-Co1	1.53E-20	0.50096093	0.994	0.998	2.84E-16	6	mt-Co1

Dusp3	1.54E-20	0.46211802	0.572	0.469	2.87E-16	6	Dusp3
Rbm25	1.91E-20	0.45553125	0.823	0.845	3.54E-16	6	Rbm25
Tsc22d2	2.52E-20	0.52212099	0.55	0.429	4.68E-16	6	Tsc22d2
Fyn	2.97E-20	0.42485975	0.671	0.61	5.53E-16	6	Fyn
Cyfip2	3.50E-20	0.46353623	0.665	0.633	6.51E-16	6	Cyfip2
Ccr21	3.81E-20	0.48061365	0.855	0.801	7.08E-16	6	Ccr2
Fmnl2	5.31E-20	0.49726113	0.602	0.516	9.86E-16	6	Fmnl2
Mbtd1	5.63E-20	0.46355861	0.536	0.43	1.05E-15	6	Mbtd1
Prrc2a	5.69E-20	0.46749928	0.566	0.477	1.06E-15	6	Prrc2a
Adam8	6.58E-20	0.58545674	0.683	0.6	1.22E-15	6	Adam8
Tgoln1	7.37E-20	0.5059821	0.691	0.646	1.37E-15	6	Tgoln1
Trim8	8.57E-20	0.50525988	0.536	0.426	1.59E-15	6	Trim8
Nabp1	8.60E-20	0.49513798	0.653	0.589	1.60E-15	6	Nabp1
Nufip2	1.02E-19	0.45730758	0.665	0.61	1.89E-15	6	Nufip2
Pcf111	1.02E-19	0.5092157	0.566	0.467	1.90E-15	6	Pcf11
Eid11	1.19E-19	0.46289733	0.757	0.778	2.20E-15	6	Eid1
Med13l	1.21E-19	0.47327449	0.562	0.474	2.24E-15	6	Med13l
Nipbl	1.27E-19	0.48382309	0.645	0.58	2.36E-15	6	Nipbl
Btaf1	1.31E-19	0.44061528	0.506	0.385	2.44E-15	6	Btaf1
Arl5b	1.36E-19	0.51653982	0.48	0.357	2.52E-15	6	Arl5b
Tiparp1	1.59E-19	0.52345819	0.803	0.77	2.96E-15	6	Tiparp
Ctnna1	1.84E-19	0.31490723	0.815	0.824	3.43E-15	6	Ctnna1
Mia3	2.69E-19	0.47133457	0.552	0.465	5.00E-15	6	Mia3

Homer1	3.55E-19	0.44708494	0.359	0.222	6.59E-15	6	Homer1
Per1	3.86E-19	0.50134701	0.51	0.394	7.17E-15	6	Per1
Slfn5	5.23E-19	0.38423242	0.829	0.766	9.72E-15	6	Slfn5
Atp6v0a2	5.83E-19	0.39229238	0.745	0.699	1.08E-14	6	Atp6v0a2
Ifngr1	7.26E-19	0.41848331	0.888	0.866	1.35E-14	6	Ifngr1
Baz2b	7.97E-19	0.4750828	0.723	0.683	1.48E-14	6	Baz2b
Tmem30a	9.80E-19	0.45191446	0.633	0.575	1.82E-14	6	Tmem30a
Nfkb1	9.92E-19	0.36222973	0.661	0.585	1.84E-14	6	Nfkb1
Prpf38b	1.01E-18	0.42018619	0.739	0.705	1.87E-14	6	Prpf38b
Kat6a	1.02E-18	0.43296761	0.572	0.477	1.90E-14	6	Kat6a
Setd2	1.29E-18	0.41006793	0.494	0.383	2.40E-14	6	Setd2
Nav1	1.40E-18	0.49477623	0.62	0.542	2.60E-14	6	Nav1
Fam102b	1.72E-18	0.48544387	0.474	0.353	3.19E-14	6	Fam102b
Zfp516	1.98E-18	0.4822434	0.572	0.491	3.69E-14	6	Zfp516
Hnrnpul2	2.18E-18	0.45302941	0.717	0.699	4.05E-14	6	Hnrnpul2
H13	2.60E-18	0.41241732	0.853	0.871	4.83E-14	6	H13
Ier52	2.90E-18	0.32835574	0.98	0.969	5.38E-14	6	Ier5
Mar-07	3.34E-18	0.54134722	0.618	0.586	6.20E-14	6	Mar-07
Uggt1	5.00E-18	0.44678781	0.538	0.439	9.28E-14	6	Uggt1
Nfkbid1	5.42E-18	0.5021036	0.532	0.415	1.01E-13	6	Nfkbid
Ap2a2	5.46E-18	0.47362193	0.675	0.645	1.01E-13	6	Ap2a2
Nktr1	5.57E-18	0.46223492	0.727	0.696	1.03E-13	6	Nktr
Rnf19b1	5.84E-18	0.58708443	0.614	0.537	1.09E-13	6	Rnf19b

Rab322	6.57E-18	0.45955015	0.719	0.702	1.22E-13	6	Rab32
Pbxip1	6.99E-18	0.42444611	0.526	0.418	1.30E-13	6	Pbxip1
Hipk1	7.55E-18	0.44642617	0.568	0.493	1.40E-13	6	Hipk1
Nup98	9.95E-18	0.4488147	0.645	0.599	1.85E-13	6	Nup98
Polr1a	1.18E-17	0.40667963	0.536	0.427	2.19E-13	6	Polr1a
Itsn2	1.41E-17	0.43182617	0.566	0.487	2.62E-13	6	Itsn2
Smchd1	1.55E-17	0.47717423	0.743	0.738	2.87E-13	6	Smchd1
Ehbp1l1	1.65E-17	0.44411388	0.59	0.526	3.06E-13	6	Ehbp1l1
Slc6a6	1.78E-17	0.36946055	0.753	0.726	3.31E-13	6	Slc6a6
Lrrc8d	2.00E-17	0.42382538	0.414	0.289	3.72E-13	6	Lrrc8d
Htt	2.05E-17	0.42649642	0.476	0.361	3.80E-13	6	Htt
Itprip	2.29E-17	0.4086326	0.382	0.257	4.25E-13	6	Itprip
Ndel11	2.64E-17	0.48830172	0.749	0.747	4.91E-13	6	Ndel1
Gna131	2.92E-17	0.42436817	0.809	0.835	5.43E-13	6	Gna13
Fnip2	3.35E-17	0.46652044	0.45	0.335	6.23E-13	6	Fnip2
2900097C17Rik	3.76E-17	0.43717532	0.532	0.445	6.99E-13	6	2900097C17Rik
Gramd2	4.03E-17	0.43812774	0.438	0.321	7.49E-13	6	Gramd2
Pianp3	4.30E-17	0.44257164	0.518	0.396	7.99E-13	6	Pianp
Tet3	4.58E-17	0.41145843	0.498	0.387	8.50E-13	6	Tet3
Bclaf1	4.72E-17	0.45192799	0.715	0.7	8.77E-13	6	Bclaf1
Ubr5	4.80E-17	0.41464018	0.43	0.313	8.92E-13	6	Ubr5
Foxp1	5.09E-17	0.53597923	0.661	0.6	9.46E-13	6	Foxp1
Ivns1abp	6.26E-17	0.42280459	0.683	0.662	1.16E-12	6	Ivns1abp

Tbl1xr1	7.11E-17	0.38371666	0.508	0.401	1.32E-12	6	Tbl1xr1
Ppp1r18	7.97E-17	0.49480249	0.723	0.743	1.48E-12	6	Ppp1r18
Lifr	8.28E-17	0.31837792	0.249	0.129	1.54E-12	6	Lifr
Pdlim5	1.01E-16	0.51491204	0.542	0.462	1.88E-12	6	Pdlim5
Lamp2	1.21E-16	0.53010073	0.673	0.648	2.24E-12	6	Lamp2
Rnf145	1.25E-16	0.40614859	0.426	0.312	2.33E-12	6	Rnf145
Fos2	1.41E-16	0.33616573	0.998	0.975	2.62E-12	6	Fos
Plekhm3	1.44E-16	0.46569078	0.574	0.491	2.68E-12	6	Plekhm3
Pacs2	1.82E-16	0.34653738	0.361	0.236	3.39E-12	6	Pacs2
Cmtr1	2.07E-16	0.4921687	0.705	0.671	3.84E-12	6	Cmtr1
Pkn1	2.29E-16	0.47374917	0.62	0.579	4.25E-12	6	Pkn1
Azin1	2.31E-16	0.45582528	0.58	0.531	4.29E-12	6	Azin1
Mef2d	2.33E-16	0.39515923	0.544	0.453	4.33E-12	6	Mef2d
Znrf2	2.34E-16	0.42905436	0.554	0.485	4.35E-12	6	Znrf2
Tab2	3.58E-16	0.43013703	0.631	0.578	6.65E-12	6	Tab2
Ranbp9	3.90E-16	0.37154837	0.355	0.233	7.25E-12	6	Ranbp9
Ankrd17	4.27E-16	0.53582332	0.572	0.522	7.94E-12	6	Ankrd17
Wac	4.35E-16	0.46994597	0.681	0.652	8.09E-12	6	Wac
Ncaph2	4.45E-16	0.36478083	0.398	0.281	8.26E-12	6	Ncaph2
Nxf1	4.51E-16	0.34854444	0.416	0.304	8.38E-12	6	Nxf1
Cpeb2	4.70E-16	0.35147482	0.335	0.21	8.74E-12	6	Cpeb2
Canx1	5.23E-16	0.39760413	0.807	0.822	9.71E-12	6	Canx
Kmt2e	5.48E-16	0.3920246	0.703	0.669	1.02E-11	6	Kmt2e

Crebbp	5.50E-16	0.42442368	0.574	0.509	1.02E-11	6	Crebbp
Zfp106	5.64E-16	0.42360211	0.729	0.727	1.05E-11	6	Zfp106
Ap1b1	5.74E-16	0.37805684	0.57	0.502	1.07E-11	6	Ap1b1
Arhgef6	6.15E-16	0.45620363	0.663	0.622	1.14E-11	6	Arhgef6
Pik3ap1	6.42E-16	0.41936945	0.739	0.718	1.19E-11	6	Pik3ap1
Dusp16	6.68E-16	0.4403219	0.454	0.345	1.24E-11	6	Dusp16
Ubl3	7.20E-16	0.39487777	0.727	0.719	1.34E-11	6	Ubl3
Zfp207	7.39E-16	0.41323354	0.61	0.556	1.37E-11	6	Zfp207
Ankrd11	9.19E-16	0.46445742	0.709	0.711	1.71E-11	6	Ankrd11
Fli1	9.28E-16	0.41402266	0.492	0.399	1.72E-11	6	Fli1
Xiap	9.64E-16	0.40793482	0.574	0.508	1.79E-11	6	Xiap
Fosl2	9.83E-16	0.49852965	0.51	0.416	1.83E-11	6	Fosl2
Tlr31	9.89E-16	0.42084579	0.757	0.713	1.84E-11	6	Tlr3
Cd1801	1.20E-15	0.58906447	0.596	0.518	2.24E-11	6	Cd180
Arih2	1.23E-15	0.43988961	0.699	0.699	2.29E-11	6	Arih2
Cnot1	1.42E-15	0.39706859	0.528	0.437	2.64E-11	6	Cnot1
Caprin1	1.44E-15	0.40089006	0.719	0.722	2.68E-11	6	Caprin1
P3h2	2.47E-15	0.3171519	0.265	0.15	4.59E-11	6	P3h2
Btg11	2.65E-15	0.30635885	0.982	0.989	4.93E-11	6	Btg1
Ccnt1	3.06E-15	0.39711276	0.546	0.46	5.69E-11	6	Ccnt1
Cd53	3.32E-15	0.33453322	0.871	0.91	6.17E-11	6	Cd53
Gnb1	3.34E-15	0.32957514	0.815	0.865	6.20E-11	6	Gnb1
Pan3	3.71E-15	0.43049226	0.522	0.433	6.88E-11	6	Pan3

Ppp2r5a	4.52E-15	0.3699462	0.59	0.526	8.39E-11	6	Ppp2r5a
Setd7	6.00E-15	0.31363929	0.323	0.207	1.12E-10	6	Setd7
Atf7ip1	6.30E-15	0.45568151	0.446	0.34	1.17E-10	6	Atf7ip
Usp25	7.62E-15	0.4420111	0.598	0.535	1.42E-10	6	Usp25
Shtn11	8.02E-15	0.31205725	0.88	0.885	1.49E-10	6	Shtn1
Acss1	9.59E-15	0.374744	0.39	0.283	1.78E-10	6	Acss1
Gramd4	9.80E-15	0.33125809	0.376	0.258	1.82E-10	6	Gramd4
Plekhb2	9.90E-15	0.37445045	0.45	0.354	1.84E-10	6	Plekhb2
Amz1	1.10E-14	0.45370406	0.498	0.413	2.05E-10	6	Amz1
Gpr141b	1.12E-14	0.4422287	0.534	0.435	2.08E-10	6	Gpr141b
Galc	1.14E-14	0.43856984	0.311	0.198	2.12E-10	6	Galc
Myo9b	1.14E-14	0.37931758	0.434	0.332	2.13E-10	6	Myo9b
Ilf3	1.22E-14	0.44050758	0.418	0.311	2.27E-10	6	Ilf3
Hepacam21	1.29E-14	0.47761539	0.576	0.503	2.40E-10	6	Hepacam2
Dusp5	1.33E-14	0.50955702	0.795	0.81	2.47E-10	6	Dusp5
Smg7	1.40E-14	0.38830002	0.426	0.328	2.60E-10	6	Smg7
Pcbp2	1.44E-14	0.30246333	0.89	0.93	2.68E-10	6	Pcbp2
Arf3	1.58E-14	0.35429536	0.649	0.611	2.94E-10	6	Arf3
Pik3r1	1.67E-14	0.38999491	0.528	0.427	3.10E-10	6	Pik3r1
Acox32	1.69E-14	0.48204457	0.622	0.568	3.15E-10	6	Acox3
Prkcd	1.85E-14	0.44147153	0.633	0.592	3.44E-10	6	Prkcd
Antxr2	2.04E-14	0.47620822	0.655	0.629	3.78E-10	6	Antxr2
Ogt	2.09E-14	0.40558282	0.426	0.325	3.89E-10	6	Ogt

Pirb	2.10E-14	0.39657535	0.675	0.658	3.90E-10	6	Pirb
Khdrbs1	2.11E-14	0.37500612	0.735	0.761	3.93E-10	6	Khdrbs1
Mllt3	2.13E-14	0.4062343	0.494	0.404	3.96E-10	6	Mllt3
Rassf5	2.21E-14	0.49140813	0.518	0.454	4.10E-10	6	Rassf5
Ctdsp2	2.38E-14	0.43731223	0.564	0.511	4.42E-10	6	Ctdsp2
Arid2	2.48E-14	0.34775682	0.422	0.31	4.60E-10	6	Arid2
Slc20a11	2.55E-14	0.51705337	0.424	0.318	4.73E-10	6	Slc20a1
Ppp3r1	2.56E-14	0.41306581	0.492	0.414	4.75E-10	6	Ppp3r1
Tnks	2.69E-14	0.43262317	0.42	0.329	4.99E-10	6	Tnks
Egln1	2.75E-14	0.28391102	0.259	0.151	5.11E-10	6	Egln1
Gm63772	2.88E-14	0.42047461	0.773	0.77	5.34E-10	6	Gm6377
Tle31	3.80E-14	0.40732586	0.504	0.43	7.07E-10	6	Tle3
Pten	4.55E-14	0.42641557	0.671	0.673	8.46E-10	6	Pten
Slc25a201	4.66E-14	0.4545948	0.727	0.722	8.66E-10	6	Slc25a20
Slc8b1	4.79E-14	0.37370615	0.614	0.564	8.90E-10	6	Slc8b1
Efr3a	6.43E-14	0.40409544	0.57	0.526	1.19E-09	6	Efr3a
Arid1b	7.78E-14	0.36119368	0.442	0.345	1.45E-09	6	Arid1b
Fam71a	8.24E-14	0.37377118	0.241	0.135	1.53E-09	6	Fam71a
Slc5a31	8.57E-14	0.33704333	0.213	0.109	1.59E-09	6	Slc5a3
Cop1	9.51E-14	0.39858038	0.496	0.415	1.77E-09	6	Cop1
Hps3	9.53E-14	0.39833141	0.58	0.535	1.77E-09	6	Hps3
Trim59	1.15E-13	0.4183831	0.299	0.191	2.14E-09	6	Trim59
Ubc1	1.24E-13	0.25887285	0.992	0.992	2.31E-09	6	Ubc

Lfng	1.26E-13	0.45278632	0.47	0.382	2.35E-09	6	Lfng
Safb2	1.35E-13	0.41500398	0.528	0.468	2.50E-09	6	Safb2
Srgap3	1.59E-13	0.42167656	0.466	0.369	2.95E-09	6	Srgap3
Ctla4	1.59E-13	0.44766938	0.273	0.162	2.96E-09	6	Ctla4
Gm30978	1.85E-13	0.20538937	0.211	0.11	3.44E-09	6	Gm30978
Xist1	1.90E-13	0.57236986	0.719	0.669	3.53E-09	6	Xist
Tra2a	1.94E-13	0.38823355	0.785	0.81	3.61E-09	6	Tra2a
Chd4	1.97E-13	0.40227046	0.729	0.734	3.66E-09	6	Chd4
Fam91a1	1.99E-13	0.33843913	0.406	0.301	3.69E-09	6	Fam91a1
Nectin1	1.99E-13	0.36797366	0.382	0.274	3.70E-09	6	Nectin1
Psme2b	1.99E-13	0.25892571	0.257	0.15	3.70E-09	6	Psme2b
Itga1	2.06E-13	0.44601112	0.371	0.26	3.83E-09	6	Itga1
Nfat5	2.29E-13	0.40497819	0.4	0.296	4.25E-09	6	Nfat5
Arhgap15	2.70E-13	0.40779126	0.707	0.709	5.02E-09	6	Arhgap15
Fbxo11	3.00E-13	0.41023402	0.524	0.472	5.58E-09	6	Fbxo11
Themis2	3.25E-13	0.38781437	0.59	0.545	6.05E-09	6	Themis2
Usp34	3.33E-13	0.35226141	0.582	0.532	6.19E-09	6	Usp34
Syncrip	3.40E-13	0.40171645	0.606	0.595	6.32E-09	6	Syncrip
Itpripl2	3.62E-13	0.4020819	0.482	0.397	6.72E-09	6	Itpripl2
Nrp1	3.76E-13	0.39425631	0.512	0.428	6.99E-09	6	Nrp1
Apaf1	3.78E-13	0.38570578	0.452	0.367	7.02E-09	6	Apaf1
mt-Co2	4.71E-13	0.43797666	0.99	0.999	8.76E-09	6	mt-Co2
Trim35	5.00E-13	0.4529075	0.871	0.886	9.28E-09	6	Trim35

Paxbp1	5.43E-13	0.44043668	0.631	0.605	1.01E-08	6	Paxbp1
Dusp18	5.89E-13	0.30102406	0.261	0.154	1.09E-08	6	Dusp18
Fnip1	5.97E-13	0.38557992	0.337	0.234	1.11E-08	6	Fnip1
Srsf5	6.16E-13	0.29084628	0.942	0.963	1.14E-08	6	Srsf5
mt-Nd3	6.70E-13	0.40674229	0.938	0.944	1.24E-08	6	mt-Nd3
Mib1	6.79E-13	0.31300873	0.283	0.179	1.26E-08	6	Mib1
Glud1	7.92E-13	0.32079761	0.807	0.843	1.47E-08	6	Glud1
Cul3	7.97E-13	0.32996321	0.418	0.317	1.48E-08	6	Cul3
Thrap3	9.11E-13	0.37944693	0.729	0.735	1.69E-08	6	Thrap3
Ell2	9.77E-13	0.41872702	0.444	0.362	1.82E-08	6	Ell2
Kat2b	9.96E-13	0.33011005	0.307	0.207	1.85E-08	6	Kat2b
Junb1	1.02E-12	0.36976163	0.994	0.99	1.90E-08	6	Junb
Mat2a1	1.03E-12	0.35973824	0.709	0.714	1.92E-08	6	Mat2a
Ubap2l	1.05E-12	0.41740959	0.727	0.751	1.95E-08	6	Ubap2l
Fgd6	1.17E-12	0.34833162	0.382	0.28	2.18E-08	6	Fgd6
Mga	1.19E-12	0.33471008	0.418	0.319	2.20E-08	6	Mga
Phlpp1	1.36E-12	0.36344523	0.432	0.331	2.52E-08	6	Phlpp1
Mtmr10	1.47E-12	0.38075676	0.319	0.217	2.73E-08	6	Mtmr10
Lypla2	1.53E-12	0.44387111	0.544	0.504	2.84E-08	6	Lypla2
Kmt2c	1.60E-12	0.37365348	0.52	0.452	2.97E-08	6	Kmt2c
Trim30a	1.61E-12	0.3346962	0.829	0.837	2.99E-08	6	Trim30a
Ncl1	1.61E-12	0.28907273	0.944	0.954	3.00E-08	6	Ncl
Zfp710	1.62E-12	0.38638896	0.546	0.487	3.02E-08	6	Zfp710

Hsph11	1.64E-12	0.63259824	0.514	0.438	3.05E-08	6	Hsph1
Khsrp	1.76E-12	0.39816269	0.442	0.362	3.27E-08	6	Khsrp
Dpp4	2.01E-12	0.33980937	0.785	0.8	3.73E-08	6	Dpp4
Lrrc58	2.20E-12	0.37487399	0.492	0.417	4.08E-08	6	Lrrc58
Arid4a	2.21E-12	0.36256678	0.791	0.81	4.11E-08	6	Arid4a
Lima1	2.28E-12	0.29584979	0.5	0.4	4.24E-08	6	Lima1
Ythdc1	2.47E-12	0.38284114	0.769	0.793	4.58E-08	6	Ythdc1
Ppp1r12a	2.66E-12	0.37821182	0.657	0.662	4.94E-08	6	Ppp1r12a
Ppt2	2.68E-12	0.38567631	0.416	0.322	4.98E-08	6	Ppt2
Cep120	3.06E-12	0.30611845	0.285	0.186	5.68E-08	6	Cep120
Emsy	3.43E-12	0.37969528	0.335	0.241	6.38E-08	6	Emsy
Phip	3.49E-12	0.40213208	0.488	0.413	6.48E-08	6	Phip
Kpnb1	3.54E-12	0.3597278	0.48	0.411	6.57E-08	6	Kpnb1
Cd226	3.57E-12	0.4467984	0.446	0.365	6.63E-08	6	Cd226
Rlim	4.04E-12	0.37004427	0.59	0.551	7.52E-08	6	Rlim
Dyrk1a	5.74E-12	0.31421948	0.369	0.272	1.07E-07	6	Dyrk1a
Stat2	5.86E-12	0.42157211	0.578	0.502	1.09E-07	6	Stat2
Zbtb20	5.91E-12	0.37703399	0.343	0.242	1.10E-07	6	Zbtb20
Nsd1	5.97E-12	0.41894066	0.612	0.589	1.11E-07	6	Nsd1
Itgb2	6.48E-12	0.36607539	0.839	0.855	1.20E-07	6	Itgb2
Pqlc1	6.95E-12	0.38109613	0.616	0.575	1.29E-07	6	Pqlc1
Fcrla1	7.19E-12	0.40427789	0.466	0.361	1.34E-07	6	Fcrla
Gnaq	7.57E-12	0.31998664	0.428	0.338	1.41E-07	6	Gnaq

Zc3h7a	7.83E-12	0.39044454	0.558	0.508	1.46E-07	6	Zc3h7a
Etv6	7.99E-12	0.33648106	0.661	0.663	1.49E-07	6	Etv6
Herc1	8.05E-12	0.35098012	0.422	0.338	1.50E-07	6	Herc1
Ank1	8.30E-12	0.27433546	0.249	0.153	1.54E-07	6	Ank1
Ccnt2	9.81E-12	0.36520718	0.44	0.358	1.82E-07	6	Ccnt2
Itga4	9.95E-12	0.32878906	0.727	0.707	1.85E-07	6	Itga4
Ppp3ca	1.00E-11	0.41104989	0.58	0.54	1.86E-07	6	Ppp3ca
Birc6	1.01E-11	0.4049513	0.588	0.566	1.87E-07	6	Birc6
Klrb1b1	1.05E-11	0.42273	0.723	0.696	1.95E-07	6	Klrb1b
Tulp4	1.06E-11	0.35735033	0.442	0.361	1.97E-07	6	Tulp4
Dbn1	1.19E-11	0.36268397	0.554	0.505	2.21E-07	6	Dbn1
Hnrnph2	1.30E-11	0.35800572	0.631	0.632	2.42E-07	6	Hnrnph2
Tnpo1	1.42E-11	0.33138975	0.426	0.335	2.63E-07	6	Tnpo1
Tnrc18	1.48E-11	0.38722051	0.498	0.44	2.75E-07	6	Tnrc18
Adar	1.54E-11	0.30111	0.382	0.288	2.86E-07	6	Adar
Cytip	1.59E-11	0.24534325	0.968	0.984	2.96E-07	6	Cytip
Xpr1	1.65E-11	0.35789155	0.357	0.266	3.06E-07	6	Xpr1
Dock5	1.67E-11	0.31351931	0.373	0.28	3.11E-07	6	Dock5
Rnf144b	1.77E-11	0.32571745	0.313	0.215	3.29E-07	6	Rnf144b
Ankrd13a	1.79E-11	0.36170392	0.532	0.485	3.32E-07	6	Ankrd13a
Fbxo33	1.90E-11	0.292099	0.325	0.224	3.52E-07	6	Fbxo33
Msi2	1.97E-11	0.37348783	0.365	0.273	3.66E-07	6	Msi2
Tnpo3	2.12E-11	0.37079979	0.59	0.562	3.94E-07	6	Tnpo3

Prrc2c	2.31E-11	0.40564958	0.817	0.847	4.29E-07	6	Prrc2c
Uvrag	2.46E-11	0.30135003	0.799	0.812	4.57E-07	6	Uvrag
Capn2	2.68E-11	0.36339994	0.484	0.412	4.98E-07	6	Capn2
Lncpint	2.86E-11	0.6581266	0.53	0.5	5.31E-07	6	Lncpint
Sipa1l3	2.86E-11	0.32629364	0.426	0.342	5.31E-07	6	Sipa1l3
Sec24b	2.87E-11	0.36827217	0.422	0.34	5.32E-07	6	Sec24b
Hipk2	2.93E-11	0.40095689	0.558	0.515	5.44E-07	6	Hipk2
Baz2a	2.99E-11	0.29647029	0.378	0.284	5.56E-07	6	Baz2a
Eif4g1	3.15E-11	0.32482107	0.584	0.536	5.85E-07	6	Eif4g1
Jak1	3.20E-11	0.32029286	0.653	0.649	5.94E-07	6	Jak1
Baz1b	3.55E-11	0.34803638	0.458	0.388	6.59E-07	6	Baz1b
Zzef1	3.87E-11	0.33785915	0.339	0.247	7.19E-07	6	Zzef1
Pptc7	3.93E-11	0.31393377	0.351	0.258	7.30E-07	6	Pptc7
Gse1	4.26E-11	0.30408299	0.313	0.216	7.92E-07	6	Gse1
Plekha2	4.27E-11	0.36052043	0.446	0.378	7.93E-07	6	Plekha2
H2-Ob2	4.68E-11	0.28801352	0.767	0.756	8.70E-07	6	H2-Ob
Glg1	5.06E-11	0.36218623	0.418	0.341	9.39E-07	6	Glg1
Cep170	5.23E-11	0.41275155	0.51	0.462	9.71E-07	6	Cep170
Mical1	5.41E-11	0.36084779	0.454	0.382	1.00E-06	6	Mical1
Dlg1	5.65E-11	0.31619986	0.365	0.271	1.05E-06	6	Dlg1
Rassf2	5.96E-11	0.26892146	0.351	0.253	1.11E-06	6	Rassf2
Aim2	5.98E-11	0.36602372	0.524	0.478	1.11E-06	6	Aim2
Usp4	6.20E-11	0.30976182	0.396	0.311	1.15E-06	6	Usp4

Furin1	6.28E-11	0.53261418	0.592	0.53	1.17E-06	6	Furin
Ikbkb	6.40E-11	0.3556707	0.681	0.684	1.19E-06	6	Ikbkb
Limd1	6.58E-11	0.3697378	0.655	0.653	1.22E-06	6	Limd1
Ppfibp2	6.64E-11	0.37982074	0.536	0.485	1.23E-06	6	Ppfibp2
Adgre52	7.39E-11	0.56670068	0.741	0.731	1.37E-06	6	Adgre5
Ubn1	7.75E-11	0.43334686	0.562	0.523	1.44E-06	6	Ubn1
Igf2r	7.92E-11	0.31973898	0.273	0.179	1.47E-06	6	Igf2r
Far11	7.97E-11	0.3926697	0.588	0.574	1.48E-06	6	Far1
Sfxn2	8.81E-11	0.29644379	0.353	0.26	1.64E-06	6	Sfxn2
Jak2	9.16E-11	0.27457163	0.743	0.756	1.70E-06	6	Jak2
Arhgap5	9.17E-11	0.3503064	0.616	0.592	1.70E-06	6	Arhgap5
Luc7l2	9.78E-11	0.32352717	0.779	0.804	1.82E-06	6	Luc7l2
Gdi1	9.84E-11	0.30121301	0.367	0.279	1.83E-06	6	Gdi1
Cep85l	1.00E-10	0.36422594	0.329	0.24	1.86E-06	6	Cep85l
Cpeb4	1.06E-10	0.29017678	0.299	0.205	1.97E-06	6	Cpeb4
Hdac9	1.13E-10	0.26738355	0.239	0.148	2.10E-06	6	Hdac9
Capn1	1.18E-10	0.33369903	0.384	0.305	2.20E-06	6	Capn1
Kmt2d	1.21E-10	0.31135842	0.327	0.238	2.24E-06	6	Kmt2d
Kdm3a	1.33E-10	0.30973298	0.269	0.179	2.48E-06	6	Kdm3a
Atg2a	1.34E-10	0.32431648	0.351	0.265	2.49E-06	6	Atg2a
Rapgef6	1.45E-10	0.3787473	0.498	0.447	2.70E-06	6	Rapgef6
Plcb2	1.45E-10	0.30521839	0.468	0.409	2.70E-06	6	Plcb2
Ppp6r1	1.50E-10	0.40004489	0.458	0.396	2.78E-06	6	Ppp6r1

Tasor	1.57E-10	0.32411138	0.341	0.245	2.91E-06	6	Tasor
Mnt	1.61E-10	0.30872684	0.392	0.305	2.99E-06	6	Mnt
Alms1	1.65E-10	0.27364201	0.219	0.13	3.07E-06	6	Alms1
Ube2d3	1.71E-10	0.29353441	0.902	0.945	3.18E-06	6	Ube2d3
Tnrc6a	2.07E-10	0.37406268	0.633	0.643	3.85E-06	6	Tnrc6a
Acsl4	2.14E-10	0.40911508	0.492	0.457	3.97E-06	6	Acsl4
Atp1b3	2.48E-10	0.36116325	0.749	0.768	4.60E-06	6	Atp1b3
4932438A13Rik	2.48E-10	0.39124427	0.498	0.436	4.62E-06	6	4932438A13Rik
Tbc1d14	2.86E-10	0.32604975	0.426	0.352	5.31E-06	6	Tbc1d14
Chka	3.20E-10	0.36272171	0.414	0.328	5.95E-06	6	Chka
Tssc4	3.20E-10	0.49024396	0.402	0.332	5.95E-06	6	Tssc4
Inpp4a	3.45E-10	0.32661312	0.305	0.217	6.40E-06	6	Inpp4a
Tbc1d5	3.51E-10	0.31246471	0.414	0.335	6.52E-06	6	Tbc1d5
Hnrnpk	3.53E-10	0.22785997	0.916	0.953	6.56E-06	6	Hnrnpk
Ccser1	3.54E-10	0.28464434	0.233	0.146	6.58E-06	6	Ccser1
Lrp10	3.65E-10	0.35345493	0.556	0.53	6.77E-06	6	Lrp10
Tasor2	3.74E-10	0.36296041	0.382	0.299	6.95E-06	6	Tasor2
Nrros	3.94E-10	0.38282113	0.703	0.763	7.32E-06	6	Nrros
Mepce	4.10E-10	0.31943622	0.311	0.223	7.61E-06	6	Mepce
Sf3b1	4.13E-10	0.28495661	0.928	0.949	7.67E-06	6	Sf3b1
Smc5	4.17E-10	0.28294094	0.303	0.213	7.75E-06	6	Smc5
Ttc71	4.34E-10	0.36756569	0.608	0.601	8.06E-06	6	Ttc7
Plxnd1	4.36E-10	0.28029425	0.271	0.183	8.09E-06	6	Plxnd1

Larp1	4.49E-10	0.34190868	0.56	0.526	8.34E-06	6	Larp1
Usp6nl	4.51E-10	0.26900028	0.283	0.195	8.38E-06	6	Usp6nl
Cd244a	4.78E-10	0.34771805	0.444	0.369	8.87E-06	6	Cd244a
Tap2	5.70E-10	0.31277115	0.801	0.827	1.06E-05	6	Tap2
Etf1	5.90E-10	0.33812811	0.709	0.744	1.10E-05	6	Etf1
Cand1	6.11E-10	0.27978712	0.371	0.283	1.14E-05	6	Cand1
Mbnl2	6.25E-10	0.40489846	0.791	0.828	1.16E-05	6	Mbnl2
Mknk2	6.95E-10	0.35172286	0.61	0.6	1.29E-05	6	Mknk2
Asxl2	7.03E-10	0.36363322	0.42	0.354	1.31E-05	6	Asxl2
Lyn1	7.44E-10	0.26153401	0.878	0.923	1.38E-05	6	Lyn
Ptms1	7.72E-10	0.23415727	0.94	0.969	1.43E-05	6	Ptms
Zfp217	8.00E-10	0.25845815	0.287	0.198	1.49E-05	6	Zfp217
Fam49b2	8.60E-10	0.25486832	0.908	0.942	1.60E-05	6	Fam49b
Cnn2	8.62E-10	0.29089673	0.835	0.875	1.60E-05	6	Cnn2
Zbtb44	8.78E-10	0.3216533	0.301	0.215	1.63E-05	6	Zbtb44
Hspa4	8.78E-10	0.30306904	0.721	0.747	1.63E-05	6	Hspa4
Tlr11	9.49E-10	0.29486705	0.275	0.19	1.76E-05	6	Tlr11
Rab7	9.82E-10	0.32396978	0.829	0.882	1.83E-05	6	Rab7
Vps18	9.88E-10	0.31880111	0.285	0.199	1.84E-05	6	Vps18
Notch1	1.03E-09	0.22855211	0.205	0.121	1.91E-05	6	Notch1
Dennd5a	1.05E-09	0.33056475	0.456	0.393	1.95E-05	6	Dennd5a
Ncor1	1.08E-09	0.36926741	0.697	0.722	2.00E-05	6	Ncor1
Zdhhc231	1.12E-09	0.41286445	0.566	0.531	2.07E-05	6	Zdhhc23

Isy1	1.17E-09	0.40942723	0.608	0.594	2.18E-05	6	Isy1
Axin1	1.26E-09	0.24901153	0.267	0.182	2.34E-05	6	Axin1
Sbno2	1.30E-09	0.33781233	0.47	0.404	2.41E-05	6	Sbno2
Dhx15	1.32E-09	0.32366836	0.673	0.7	2.45E-05	6	Dhx15
Rnf4	1.39E-09	0.32445943	0.6	0.603	2.57E-05	6	Rnf4
Jmy	1.40E-09	0.25550671	0.221	0.137	2.61E-05	6	Jmy
Ctsb	1.43E-09	0.29907848	0.765	0.8	2.65E-05	6	Ctsb
Sptbn1	1.51E-09	0.38531419	0.48	0.433	2.80E-05	6	Sptbn1
Zbtb11	1.65E-09	0.3322749	0.367	0.294	3.06E-05	6	Zbtb11
Hsd17b11	1.65E-09	0.37450345	0.496	0.447	3.06E-05	6	Hsd17b11
Rpl23a	1.68E-09	0.27181148	0.918	0.975	3.12E-05	6	Rpl23a
Pum1	1.69E-09	0.33824831	0.524	0.488	3.15E-05	6	Pum1
Wdr37	1.73E-09	0.32409465	0.341	0.259	3.21E-05	6	Wdr37
Man2a1	1.93E-09	0.3336218	0.363	0.288	3.58E-05	6	Man2a1
Nap1l1	1.93E-09	0.26706102	0.892	0.943	3.58E-05	6	Nap1l1
Rps6ka3	1.95E-09	0.33068285	0.386	0.309	3.62E-05	6	Rps6ka3
Usp22	1.97E-09	0.32269104	0.42	0.348	3.66E-05	6	Usp22
Ppm1k	2.15E-09	0.31827895	0.321	0.239	4.00E-05	6	Ppm1k
Ddi2	2.16E-09	0.35468498	0.373	0.299	4.01E-05	6	Ddi2
Evl	2.23E-09	0.31174214	0.572	0.57	4.15E-05	6	Evl
Nsf	2.36E-09	0.37774799	0.46	0.422	4.38E-05	6	Nsf
Ripk1	2.40E-09	0.3754133	0.434	0.37	4.46E-05	6	Ripk1
Ppp6r3	2.46E-09	0.30520295	0.353	0.273	4.57E-05	6	Ppp6r3

Ipcef1	2.59E-09	0.33622315	0.347	0.266	4.82E-05	6	Ipcef1
Aftph	2.63E-09	0.32714538	0.528	0.489	4.89E-05	6	Aftph
Rapgef2	2.70E-09	0.25753715	0.275	0.188	5.02E-05	6	Rapgef2
Prkx	2.72E-09	0.35578414	0.536	0.49	5.06E-05	6	Prkx
Ttc39b	2.73E-09	0.31894578	0.339	0.259	5.07E-05	6	Ttc39b
Kdm2a	2.78E-09	0.36074193	0.472	0.432	5.16E-05	6	Kdm2a
Ubn2	2.98E-09	0.33251853	0.496	0.447	5.53E-05	6	Ubn2
Prdm2	2.99E-09	0.27718864	0.416	0.335	5.55E-05	6	Prdm2
Colgalt1	3.01E-09	0.3598783	0.588	0.584	5.60E-05	6	Colgalt1
Parp4	3.09E-09	0.30737562	0.458	0.396	5.74E-05	6	Parp4
Fam168b	3.25E-09	0.30114784	0.488	0.446	6.04E-05	6	Fam168b
Hexim1	3.25E-09	0.35480965	0.418	0.344	6.04E-05	6	Hexim1
Mdn1	3.31E-09	0.32628645	0.388	0.314	6.15E-05	6	Mdn1
Ube2z	3.38E-09	0.28239462	0.341	0.268	6.27E-05	6	Ube2z
Neurl1b	3.38E-09	0.22969846	0.209	0.127	6.28E-05	6	Neurl1b
Lrba	3.52E-09	0.33133367	0.384	0.311	6.54E-05	6	Lrba
Frmd4a	3.57E-09	0.32799553	0.442	0.366	6.64E-05	6	Frmd4a
Slc38a2	3.79E-09	0.31766189	0.809	0.861	7.05E-05	6	Slc38a2
Snrnp70	3.87E-09	0.29748482	0.761	0.78	7.19E-05	6	Snrnp70
Mycl	4.23E-09	0.32726617	0.398	0.328	7.85E-05	6	Mycl
Nek9	4.31E-09	0.23818683	0.263	0.18	8.01E-05	6	Nek9
Sec61a11	4.34E-09	0.34948443	0.554	0.527	8.06E-05	6	Sec61a1
Cdk13	4.36E-09	0.32368886	0.43	0.364	8.09E-05	6	Cdk13

Bach1	4.38E-09	0.40374566	0.516	0.48	8.15E-05	6	Bach1
Msl2	4.57E-09	0.25848501	0.347	0.266	8.48E-05	6	Msl2
Strn3	4.67E-09	0.27255931	0.422	0.356	8.68E-05	6	Strn3
Parvb	4.82E-09	0.24513681	0.219	0.14	8.96E-05	6	Parvb
Gm47283	4.82E-09	0.36284791	0.414	0.349	8.96E-05	6	Gm47283
Zc3hav1	4.88E-09	0.37381395	0.612	0.612	9.07E-05	6	Zc3hav1
Lrrc8c	5.35E-09	0.34879616	0.536	0.499	9.93E-05	6	Lrrc8c
Gns	5.72E-09	0.35173823	0.536	0.514	0.00010631	6	Gns
Rnf150	5.73E-09	0.25338243	0.237	0.155	0.00010641	6	Rnf150
Tspyl1	5.89E-09	0.3403652	0.468	0.425	0.00010949	6	Tspyl1
Pias1	6.03E-09	0.26443924	0.355	0.28	0.00011203	6	Pias1
Btbd9	6.40E-09	0.36180321	0.404	0.343	0.00011884	6	Btbd9
Sppl2a	6.63E-09	0.27882734	0.725	0.779	0.0001232	6	Sppl2a
Zbtb461	7.22E-09	0.35190061	0.476	0.436	0.00013406	6	Zbtb46
Chsy1	7.52E-09	0.29609797	0.422	0.356	0.00013971	6	Chsy1
Tm6sf1	7.73E-09	0.32928906	0.733	0.762	0.00014354	6	Tm6sf1
Utrn	7.86E-09	0.37113482	0.422	0.354	0.0001461	6	Utrn
Klf3	7.97E-09	0.38774057	0.524	0.499	0.00014814	6	Klf3
Ahr1	8.01E-09	0.39092569	0.737	0.769	0.00014882	6	Ahr
Wapl	8.89E-09	0.32704689	0.58	0.574	0.00016522	6	Wapl
Sp100	9.68E-09	0.30639482	0.801	0.838	0.00017978	6	Sp100
Rasgef1b	1.00E-08	0.48240345	0.594	0.559	0.00018616	6	Rasgef1b
Zfp62	1.01E-08	0.30829915	0.438	0.369	0.00018726	6	Zfp62

Malat11	1.02E-08	0.56642272	1	1	0.00018862	6	Malat1
Midn1	1.03E-08	0.3080843	0.532	0.497	0.0001916	6	Midn
Cdc14a	1.04E-08	0.42670617	0.51	0.47	0.00019388	6	Cdc14a
Ap3d1	1.06E-08	0.26691773	0.518	0.478	0.00019693	6	Ap3d1
Gatad2b	1.08E-08	0.30775339	0.436	0.377	0.00020095	6	Gatad2b
Kidins220	1.19E-08	0.33854361	0.361	0.291	0.00022155	6	Kidins220
Tmem127	1.21E-08	0.23612605	0.265	0.185	0.00022477	6	Tmem127
Kif1c	1.22E-08	0.207951	0.211	0.133	0.00022751	6	Kif1c
Stk17b1	1.23E-08	0.31051153	0.857	0.896	0.00022912	6	Stk17b
Arrb1	1.28E-08	0.30517451	0.339	0.265	0.00023732	6	Arrb1
Pds5a	1.29E-08	0.29165456	0.394	0.327	0.00024037	6	Pds5a
Rbms1	1.30E-08	0.35312925	0.61	0.616	0.00024191	6	Rbms1
Smarca5	1.32E-08	0.29829957	0.693	0.732	0.00024477	6	Smarca5
Arid1a	1.39E-08	0.35201951	0.56	0.556	0.00025812	6	Arid1a
Lrpprc	1.43E-08	0.25094101	0.241	0.16	0.00026491	6	Lrpprc
Akna	1.51E-08	0.385527	0.562	0.551	0.00027974	6	Akna
Matr3	1.56E-08	0.36917018	0.506	0.49	0.0002905	6	Matr3
Sdad1	1.57E-08	0.30804016	0.709	0.721	0.00029131	6	Sdad1
Rnf44	1.63E-08	0.33266609	0.446	0.401	0.00030328	6	Rnf44
Safb	1.70E-08	0.30401147	0.438	0.385	0.00031609	6	Safb
Cfh	1.72E-08	0.29567852	0.273	0.188	0.00031934	6	Cfh
Heca	1.91E-08	0.23517498	0.243	0.164	0.00035557	6	Heca
Mapre1	1.97E-08	0.27702949	0.787	0.842	0.00036512	6	Mapre1

Ube3a	2.03E-08	0.37452058	0.518	0.502	0.00037626	6	Ube3a
Clasp2	2.03E-08	0.29646574	0.335	0.263	0.00037752	6	Clasp2
Ssbp3	2.06E-08	0.28817102	0.408	0.35	0.00038189	6	Ssbp3
Itgb71	2.10E-08	0.35564911	0.857	0.888	0.00039057	6	Itgb7
Nsmaf	2.13E-08	0.31119225	0.554	0.528	0.00039539	6	Nsmaf
Pcbp1	2.42E-08	0.30677346	0.825	0.892	0.00044881	6	Pcbp1
Slc4a7	2.51E-08	0.37867986	0.48	0.427	0.00046624	6	Slc4a7
Nfam1	2.56E-08	0.32053439	0.498	0.454	0.00047486	6	Nfam1
Padi2	2.57E-08	0.28165302	0.283	0.206	0.00047703	6	Padi2
Sla1	2.59E-08	0.30600758	0.679	0.706	0.00048104	6	Sla
Gsn1	2.91E-08	0.28962395	0.769	0.788	0.00054002	6	Gsn
Ppp4r3a	3.11E-08	0.30860538	0.494	0.454	0.00057853	6	Ppp4r3a
Mapk1ip1l	3.12E-08	0.36678061	0.488	0.462	0.00057879	6	Mapk1ip1l
P4hb1	3.16E-08	0.27586939	0.757	0.797	0.0005864	6	P4hb
Hspa1a2	3.16E-08	0.30209134	0.729	0.632	0.00058702	6	Hspa1a
Adipor2	3.25E-08	0.29985427	0.474	0.44	0.00060369	6	Adipor2
Sh3pxd2b	3.26E-08	0.34697303	0.373	0.31	0.00060616	6	Sh3pxd2b
Golph3	3.40E-08	0.31945028	0.532	0.516	0.00063193	6	Golph3
Pcna	3.51E-08	0.3750854	0.594	0.606	0.00065134	6	Pcna
Atp8b4	3.71E-08	0.35749821	0.504	0.473	0.00068856	6	Atp8b4
Fkbp15	3.76E-08	0.30115818	0.363	0.292	0.00069883	6	Fkbp15
Sgpp1	4.03E-08	0.28545783	0.361	0.292	0.00074811	6	Sgpp1
Itpkb	4.09E-08	0.41462524	0.396	0.33	0.00075969	6	Itpkb

Hspa9	4.20E-08	0.31472439	0.506	0.471	0.00078121	6	Hspa9
Oga	4.45E-08	0.34520843	0.43	0.376	0.00082615	6	Oga
Plk3	4.52E-08	0.29421139	0.281	0.204	0.00084052	6	Plk3
Sema4a	4.59E-08	0.37057588	0.616	0.623	0.0008526	6	Sema4a
Trps1	4.67E-08	0.32695943	0.45	0.397	0.00086702	6	Trps1
Susd6	4.73E-08	0.30903703	0.548	0.528	0.0008791	6	Susd6
Klf22	4.80E-08	0.31199481	0.799	0.747	0.0008926	6	Klf2
Prpf18	4.82E-08	0.32746911	0.416	0.365	0.00089606	6	Prpf18
Arhgap9	4.92E-08	0.3099635	0.472	0.428	0.00091409	6	Arhgap9
Vav3	5.09E-08	0.34092989	0.54	0.523	0.00094581	6	Vav3
Ppp1r16b	5.11E-08	0.27139679	0.239	0.165	0.00095001	6	Ppp1r16b
Pygo2	5.15E-08	0.2609816	0.283	0.207	0.00095601	6	Pygo2
Lats2	5.32E-08	0.31599508	0.446	0.403	0.00098757	6	Lats2
Sh3bp1	5.34E-08	0.32485465	0.679	0.732	0.00099266	6	Sh3bp1
Csnk1g2	5.38E-08	0.29528846	0.422	0.371	0.00099892	6	Csnk1g2
Cux1	5.47E-08	0.30045068	0.61	0.604	0.00101638	6	Cux1
Pld4	5.86E-08	0.276597	0.841	0.897	0.00108924	6	Pld4
Dna2	6.17E-08	0.34785825	0.474	0.438	0.00114709	6	Dna2
Bcl11a	6.40E-08	0.37474841	0.41	0.347	0.00118998	6	Bcl11a
Erlin1	6.61E-08	0.28608323	0.655	0.673	0.00122754	6	Erlin1
Kdm6a	6.61E-08	0.34413337	0.422	0.372	0.00122869	6	Kdm6a
Tm9sf31	6.62E-08	0.26965055	0.693	0.709	0.00123083	6	Tm9sf3
Msn1	6.62E-08	0.21322886	0.892	0.926	0.00123084	6	Msn

Man1b1	6.84E-08	0.34660807	0.38	0.316	0.00127049	6	Man1b1
Abi1	7.13E-08	0.32908142	0.647	0.662	0.00132469	6	Abi1
Rgl2	7.44E-08	0.31774773	0.307	0.237	0.00138234	6	Rgl2
Tut4	7.69E-08	0.37267725	0.643	0.646	0.0014296	6	Tut4
Usp37	7.79E-08	0.27979211	0.275	0.201	0.00144743	6	Usp37
Hps5	8.26E-08	0.30180031	0.378	0.318	0.00153393	6	Hps5
Itpripl1	8.45E-08	0.26374486	0.333	0.264	0.00156976	6	Itpripl1
Arf21	8.56E-08	0.36341838	0.472	0.42	0.00159134	6	Arf2
Got11	8.59E-08	0.28283076	0.707	0.695	0.00159526	6	Got1
Arid3b	8.63E-08	0.27916505	0.325	0.258	0.00160327	6	Arid3b
Aplp2	8.73E-08	0.28225287	0.424	0.365	0.00162114	6	Aplp2
Rc3h1	8.78E-08	0.35571603	0.422	0.367	0.00163202	6	Rc3h1
Ecpas	8.79E-08	0.28230454	0.329	0.259	0.00163281	6	Ecpas
Mgrn1	8.99E-08	0.2295067	0.325	0.255	0.00166997	6	Mgrn1
Psme4	9.26E-08	0.31518061	0.406	0.355	0.00172069	6	Psme4
Cc2d1b	9.43E-08	0.24130074	0.221	0.15	0.00175234	6	Cc2d1b
Rtl5	9.70E-08	0.26343564	0.237	0.164	0.00180202	6	Rtl5
Mxi1	1.00E-07	0.28213737	0.39	0.318	0.00186431	6	Mxi1
Nckap1l	1.08E-07	0.32093316	0.618	0.637	0.00201108	6	Nckap1l
Abcc4	1.10E-07	0.23877365	0.237	0.163	0.00204812	6	Abcc4
Ext1	1.12E-07	0.30885346	0.412	0.351	0.00208615	6	Ext1
Ccdc88a	1.14E-07	0.29038482	0.643	0.667	0.00211154	6	Ccdc88a
Tacc1	1.15E-07	0.30221005	0.647	0.671	0.00214179	6	Tacc1

Nbeal1	1.20E-07	0.27730471	0.317	0.246	0.00223561	6	Nbeal1
Mtss1	1.21E-07	0.27652335	0.209	0.136	0.00225139	6	Mtss1
Xpo6	1.26E-07	0.27895999	0.285	0.211	0.00234505	6	Xpo6
Trip11	1.33E-07	0.28314145	0.46	0.42	0.00247459	6	Trip11
Nfatc2	1.34E-07	0.28820668	0.345	0.282	0.00249485	6	Nfatc2
Mfsd6	1.49E-07	0.45112497	0.464	0.427	0.00276712	6	Mfsd6
Sos1	1.49E-07	0.23134407	0.205	0.133	0.00276902	6	Sos1
Gcsam1	1.52E-07	0.30759141	0.62	0.602	0.00281933	6	Gcsam
Aebp2	1.60E-07	0.29608116	0.454	0.409	0.00296402	6	Aebp2
Lipe	1.65E-07	0.30709669	0.353	0.292	0.00305911	6	Lipe
Poldip3	1.67E-07	0.30025126	0.534	0.524	0.00310776	6	Poldip3
Rc3h2	1.67E-07	0.28363212	0.307	0.238	0.00310984	6	Rc3h2
Col4a3bp	1.76E-07	0.35975297	0.442	0.412	0.0032755	6	Col4a3bp
Kmt2a	1.80E-07	0.31712473	0.472	0.428	0.00334711	6	Kmt2a
Ythdf3	1.81E-07	0.32830742	0.438	0.395	0.0033682	6	Ythdf3
Arhgap21	1.92E-07	0.20936798	0.219	0.145	0.0035753	6	Arhgap21
Osbpl3	1.93E-07	0.28081482	0.291	0.22	0.00358919	6	Osbpl3
Zdhhc20	1.94E-07	0.24716546	0.299	0.229	0.00359686	6	Zdhhc20
Gltp1	1.98E-07	0.30783266	0.785	0.856	0.00367071	6	Gltp
Cpne3	1.98E-07	0.28822298	0.703	0.752	0.00367326	6	Cpne3
Prkch	2.02E-07	0.24319818	0.289	0.217	0.00375135	6	Prkch
Brd4	2.03E-07	0.29660927	0.592	0.609	0.00377605	6	Brd4
Hnrnpm	2.08E-07	0.23608689	0.797	0.855	0.00386714	6	Hnrnpm

Sirt1	2.19E-07	0.20247039	0.221	0.149	0.00406639	6	Sirt1
Clcn3	2.20E-07	0.31616525	0.365	0.305	0.00408344	6	Clcn3
Jund1	2.28E-07	0.20708247	0.978	0.976	0.00423061	6	Jund
Kri1	2.38E-07	0.2329095	0.245	0.173	0.00441707	6	Kri1
Zfp445	2.43E-07	0.30144152	0.337	0.274	0.00452239	6	Zfp445
Sipa1l1	2.48E-07	0.30675842	0.488	0.456	0.004605	6	Sipa1l1
Xpo1	2.59E-07	0.31705238	0.428	0.381	0.0048064	6	Xpo1
Bptf	2.63E-07	0.28985837	0.532	0.5	0.00489315	6	Bptf
Kdm2b	2.88E-07	0.28759825	0.458	0.417	0.00534291	6	Kdm2b
Lpp	3.41E-07	0.22674721	0.267	0.196	0.00633319	6	Lpp
Rtn4	3.68E-07	0.26064102	0.845	0.898	0.00684336	6	Rtn4
Actn4	3.92E-07	0.29425515	0.466	0.443	0.00728066	6	Actn4
Slc12a9	4.00E-07	0.29152776	0.357	0.297	0.0074235	6	Slc12a9
Nlk	4.06E-07	0.20345061	0.215	0.145	0.0075404	6	Nlk
Pbrm1	4.24E-07	0.27268364	0.554	0.535	0.00787722	6	Pbrm1
Tmed51	4.44E-07	0.31128594	0.823	0.874	0.00824314	6	Tmed5
Trpm7	4.55E-07	0.28594091	0.351	0.293	0.00845257	6	Trpm7
Zfp148	4.65E-07	0.29753949	0.394	0.343	0.00864825	6	Zfp148
Fryl	4.84E-07	0.26713514	0.426	0.373	0.00900112	6	Fryl
Gcnt22	5.03E-07	0.38326081	0.747	0.735	0.00934725	6	Gcnt2
Rbm5	5.08E-07	0.40040573	0.596	0.614	0.00943893	6	Rbm5
Cnot6l	5.18E-07	0.32437411	0.404	0.36	0.00961883	6	Cnot6l
Dot1l	5.28E-07	0.31585831	0.468	0.431	0.00980352	6	Dot1l

Il10ra	5.77E-07	0.32018894	0.367	0.305	0.01071598	6	Il10ra
P2ry14	5.91E-07	0.43169459	0.574	0.605	0.01097185	6	P2ry14
Flna1	6.54E-07	0.33038129	0.809	0.846	0.0121494	6	Flna
Ireb2	6.60E-07	0.24222573	0.267	0.202	0.01226408	6	Ireb2
Samd9l	6.62E-07	0.30541164	0.624	0.619	0.01230498	6	Samd9l
Dhx9	6.64E-07	0.34066577	0.574	0.58	0.01234556	6	Dhx9
Ints61	6.76E-07	0.38925522	0.42	0.379	0.01255695	6	Ints6
St3gal4	7.06E-07	0.33162438	0.602	0.622	0.0131089	6	St3gal4
Mycbp2	7.14E-07	0.31646756	0.639	0.653	0.01325752	6	Mycbp2
G3bp1	7.17E-07	0.30456999	0.715	0.783	0.0133181	6	G3bp1
Tardbp	7.23E-07	0.30557688	0.52	0.512	0.01344196	6	Tardbp
Ipo7	7.28E-07	0.29751822	0.388	0.336	0.01352157	6	Ipo7
Il4ra	7.64E-07	0.2986463	0.402	0.345	0.01419989	6	Il4ra
Cyth1	7.76E-07	0.26531534	0.524	0.511	0.01441994	6	Cyth1
Rilpl22	8.48E-07	0.29152731	0.62	0.597	0.01575674	6	Rilpl2
Mbp	8.52E-07	0.2486296	0.586	0.587	0.01582953	6	Mbp
Cap1	8.71E-07	0.23696838	0.755	0.8	0.01617842	6	Cap1
Lmbrd1	8.79E-07	0.39305853	0.492	0.484	0.01633185	6	Lmbrd1
Tnfrsf21	8.82E-07	0.27004243	0.313	0.248	0.01638454	6	Tnfrsf21
Akap9	8.88E-07	0.33609357	0.677	0.724	0.01650763	6	Akap9
Cdkn1b	9.05E-07	0.29721357	0.699	0.733	0.0168061	6	Cdkn1b
Sema4f	9.14E-07	0.2978341	0.295	0.232	0.01697666	6	Sema4f
Mtmr4	9.38E-07	0.27179522	0.378	0.329	0.01742743	6	Mtmr4

Slc8a1	9.50E-07	0.35330985	0.361	0.31	0.01765683	6	Slc8a1
Zcchc24	9.52E-07	0.24900108	0.245	0.178	0.01768596	6	Zcchc24
Epn1	1.02E-06	0.28218943	0.61	0.63	0.01892382	6	Epn1
Zfp366	1.03E-06	0.27744034	0.341	0.283	0.01919442	6	Zfp366
Fam53c	1.04E-06	0.2315838	0.201	0.138	0.0193508	6	Fam53c
Usp8	1.04E-06	0.30584711	0.508	0.499	0.01940661	6	Usp8
Dock7	1.12E-06	0.23226647	0.241	0.175	0.02080272	6	Dock7
Larp4	1.13E-06	0.34437535	0.472	0.45	0.0209028	6	Larp4
Xbp11	1.14E-06	0.29565825	0.713	0.749	0.02109039	6	Xbp1
Stk38	1.19E-06	0.30172074	0.554	0.551	0.02203149	6	Stk38
Spty2d11	1.19E-06	0.29968391	0.731	0.749	0.02216203	6	Spty2d1
Chd3	1.20E-06	0.30603385	0.42	0.385	0.02226917	6	Chd3
Wdfy3	1.21E-06	0.22372481	0.237	0.171	0.02245535	6	Wdfy3
Vps13a	1.21E-06	0.28908643	0.339	0.283	0.0224687	6	Vps13a
Elf4	1.22E-06	0.27885935	0.386	0.342	0.0227102	6	Elf4
Ssr31	1.25E-06	0.25897963	0.725	0.773	0.02318585	6	Ssr3
Tlnrd1	1.32E-06	0.26577601	0.309	0.249	0.02457394	6	Tlnrd1
Acbd5	1.33E-06	0.32271172	0.456	0.43	0.02475385	6	Acbd5
Tshz1	1.35E-06	0.26123852	0.305	0.239	0.02505423	6	Tshz1
Dck	1.41E-06	0.35804175	0.526	0.504	0.02628481	6	Dck
Dock11	1.42E-06	0.27117432	0.442	0.399	0.02640617	6	Dock11
Gfod1	1.43E-06	0.38691192	0.488	0.47	0.02665635	6	Gfod1
Casp2	1.48E-06	0.23502357	0.247	0.18	0.02750933	6	Casp2

Amfr	1.49E-06	0.29820804	0.38	0.338	0.02762869	6	Amfr
Mob1b	1.50E-06	0.26709673	0.373	0.324	0.0278557	6	Mob1b
Grn	1.50E-06	0.30573364	0.715	0.78	0.0279549	6	Grn
Atxn7l1	1.51E-06	0.25741796	0.394	0.339	0.0281293	6	Atxn7l1
Nisch	1.52E-06	0.30278932	0.394	0.348	0.02826126	6	Nisch
Ezr1	1.54E-06	0.22235105	0.709	0.751	0.0286233	6	Ezr
Glyr1	1.56E-06	0.31397803	0.508	0.506	0.02899218	6	Glyr1
Gpbp1l1	1.60E-06	0.30777454	0.4	0.366	0.02966285	6	Gpbp1l1
Fnbp4	1.60E-06	0.29439345	0.446	0.421	0.02981596	6	Fnbp4
Rlf	1.64E-06	0.30375949	0.436	0.406	0.03039794	6	Rlf
Ppp4r3b	1.64E-06	0.25925015	0.538	0.533	0.03040545	6	Ppp4r3b
Grk3	1.71E-06	0.22815575	0.496	0.466	0.031834	6	Grk3
M6pr	1.72E-06	0.27515376	0.825	0.86	0.03198491	6	M6pr
Vps13d	1.73E-06	0.20956173	0.209	0.143	0.03213572	6	Vps13d
Tnfrsf1b	1.74E-06	0.260035	0.343	0.286	0.03238248	6	Tnfrsf1b
Kmo	1.76E-06	0.26984419	0.522	0.508	0.03266533	6	Kmo
Mef2a	1.79E-06	0.3121504	0.637	0.671	0.03322741	6	Mef2a
Git2	1.80E-06	0.29262161	0.424	0.388	0.0333766	6	Git2
Celf1	1.82E-06	0.35251695	0.402	0.371	0.0338756	6	Celf1
Tcf20	1.82E-06	0.2439108	0.299	0.237	0.03390435	6	Tcf20
Homer2	1.85E-06	0.24107305	0.201	0.135	0.03445536	6	Homer2
Copa1	1.85E-06	0.29123915	0.574	0.581	0.03446416	6	Copa
Vav1	2.04E-06	0.28459992	0.478	0.468	0.03797223	6	Vav1

Srek1	2.05E-06	0.34699944	0.424	0.396	0.03816959	6	Srek1
Psd4	2.06E-06	0.24592355	0.307	0.247	0.0383177	6	Psd4
Rnf213	2.10E-06	0.26957741	0.508	0.469	0.03908455	6	Rnf213
Wdfy1	2.14E-06	0.25171292	0.237	0.173	0.03973558	6	Wdfy1
Adora3	2.24E-06	0.22040148	0.221	0.157	0.04155913	6	Adora3
Lmna2	2.25E-06	0.38521846	0.602	0.55	0.04182839	6	Lmna
Gm43914	2.27E-06	0.30726913	0.289	0.232	0.04216964	6	Gm43914
Jun-01	2.35E-06	0.24901334	0.837	0.821	0.04371156	6	Jun
Ggnbp2	2.37E-06	0.24405598	0.51	0.489	0.04397713	6	Ggnbp2
Eps15	2.54E-06	0.28903605	0.514	0.498	0.04722098	6	Eps15
Bach2	2.69E-06	0.21824108	0.233	0.167	0.04992725	6	Bach2
Arnt	2.73E-06	0.26097371	0.317	0.258	0.05080113	6	Arnt
Mier1	2.81E-06	0.29484946	0.637	0.663	0.05213698	6	Mier1
Erf	3.02E-06	0.21594067	0.273	0.208	0.05605218	6	Erf
Pik3r5	3.06E-06	0.24288159	0.376	0.327	0.05686518	6	Pik3r5
Atl3	3.06E-06	0.20755829	0.281	0.22	0.05692158	6	Atl3
Myo18a	3.23E-06	0.29918691	0.436	0.402	0.05998908	6	Myo18a
Prrc2b	3.35E-06	0.32358881	0.367	0.322	0.06224679	6	Prrc2b
Ncoa2	3.39E-06	0.25874511	0.317	0.262	0.0629432	6	Ncoa2
Dctn1	3.59E-06	0.28996194	0.345	0.301	0.06674842	6	Dctn1
Gsk3b	3.67E-06	0.30704473	0.522	0.529	0.06821737	6	Gsk3b
Ewsr1	3.68E-06	0.28250769	0.592	0.628	0.06828705	6	Ewsr1
Prr5l	3.74E-06	0.27978916	0.528	0.508	0.06941624	6	Prr5l

St8sia4	3.78E-06	0.33821233	0.651	0.667	0.07032359	6	St8sia4
Bicd2	3.80E-06	0.2342308	0.273	0.215	0.07053988	6	Bicd2
Epb41	3.88E-06	0.2780224	0.44	0.404	0.07205335	6	Epb41
Pds5b	3.94E-06	0.21312811	0.227	0.164	0.07315787	6	Pds5b
Phf23	3.95E-06	0.29826576	0.341	0.289	0.07333883	6	Phf23
Phactr2	4.05E-06	0.27710668	0.494	0.48	0.07518186	6	Phactr2
Tmem135	4.20E-06	0.21655351	0.239	0.176	0.07796868	6	Tmem135
Tmem131l	4.37E-06	0.26101263	0.46	0.423	0.08119795	6	Tmem131l
Supt20	4.46E-06	0.3239878	0.536	0.556	0.0828493	6	Supt20
Rbm39	4.53E-06	0.20414366	0.964	0.988	0.08408009	6	Rbm39
Ttyh3	4.79E-06	0.31280522	0.4	0.369	0.08909078	6	Ttyh3
Elk4	5.10E-06	0.24918661	0.293	0.24	0.09482834	6	Elk4
Unc93b1	5.23E-06	0.27242202	0.873	0.924	0.09709447	6	Unc93b1
Mfsd14a	5.56E-06	0.31032297	0.496	0.499	0.10334062	6	Mfsd14a
Senp2	5.92E-06	0.28107197	0.468	0.434	0.11002758	6	Senp2
Sgpl1	5.96E-06	0.31334392	0.454	0.438	0.11065338	6	Sgpl1
Tns3	6.04E-06	0.29726857	0.438	0.406	0.11225311	6	Tns3
Tnrc6b	6.40E-06	0.30241718	0.398	0.363	0.11883289	6	Tnrc6b
Dgat1	6.40E-06	0.25948656	0.225	0.166	0.11890048	6	Dgat1
Eif3b	6.41E-06	0.24674247	0.484	0.466	0.11907287	6	Eif3b
Fam193a	6.46E-06	0.26320051	0.339	0.287	0.11995699	6	Fam193a
Zdhhc9	6.56E-06	0.21761453	0.247	0.185	0.12181149	6	Zdhhc9
Slc7a1	6.91E-06	0.21852841	0.297	0.233	0.12829919	6	Slc7a1

Zmym5	7.51E-06	0.23099609	0.293	0.235	0.1395793	6	Zmym5
Cdk11b	7.71E-06	0.26694625	0.622	0.652	0.14329433	6	Cdk11b
Abcc1	7.75E-06	0.22128092	0.305	0.251	0.1439575	6	Abcc1
Gpr4	7.97E-06	0.25106642	0.247	0.191	0.14807229	6	Gpr4
Bag31	7.99E-06	0.23110131	0.211	0.149	0.14844816	6	Bag3
Sp1	8.00E-06	0.27145675	0.313	0.259	0.14863802	6	Sp1
Top2b	8.14E-06	0.30706345	0.566	0.593	0.15121265	6	Top2b
Mafk	8.27E-06	0.26428287	0.361	0.319	0.15365615	6	Mafk
Irf2bp2	8.38E-06	0.32344478	0.494	0.473	0.15567089	6	Irf2bp2
Usp24	8.44E-06	0.2094153	0.229	0.17	0.15679276	6	Usp24
Fem1c	8.50E-06	0.27621415	0.651	0.68	0.157846	6	Fem1c
Ap1s3	8.56E-06	0.28679545	0.47	0.468	0.15900502	6	Ap1s3
Rin3	9.14E-06	0.32888423	0.52	0.519	0.16990651	6	Rin3
Mau2	9.23E-06	0.23407884	0.307	0.254	0.17147101	6	Mau2
Tnrc6c	9.33E-06	0.26392982	0.369	0.328	0.17328444	6	Tnrc6c
Vps13b	1.01E-05	0.28914893	0.275	0.222	0.18702029	6	Vps13b
Gak	1.01E-05	0.2263046	0.341	0.296	0.18761919	6	Gak
Crybg3	1.03E-05	0.23542728	0.261	0.204	0.19075633	6	Crybg3
Myo1f	1.05E-05	0.30204811	0.576	0.607	0.19498414	6	Myo1f
Asxl1	1.06E-05	0.20933604	0.233	0.175	0.19712056	6	Asxl1
Cbfb	1.08E-05	0.27657758	0.426	0.403	0.20100499	6	Cbfb
Cep350	1.08E-05	0.28191661	0.46	0.44	0.20126831	6	Cep350
Mfsd14b	1.10E-05	0.23425541	0.321	0.27	0.20423139	6	Mfsd14b

Cpsf7	1.10E-05	0.27197271	0.309	0.26	0.20439814	6	Cpsf7
Ppm1h	1.11E-05	0.26232021	0.305	0.251	0.20590213	6	Ppm1h
Chd8	1.11E-05	0.28213527	0.371	0.331	0.20705361	6	Chd8
Adnp	1.11E-05	0.23258059	0.329	0.277	0.20707728	6	Adnp
Gpr35	1.15E-05	0.29083903	0.418	0.383	0.21318615	6	Gpr35
Tob1	1.15E-05	0.29656755	0.329	0.272	0.21373586	6	Tob1
Jag2	1.16E-05	0.22423624	0.239	0.182	0.21605915	6	Jag2
Zfp871	1.17E-05	0.24656858	0.295	0.243	0.21825632	6	Zfp871
Rragc	1.19E-05	0.24501764	0.45	0.424	0.22136633	6	Rragc
Slc16a61	1.25E-05	0.36785032	0.386	0.342	0.2322296	6	Slc16a6
Kmt5b	1.28E-05	0.26265037	0.343	0.294	0.23696522	6	Kmt5b
Sh3bgrl	1.30E-05	0.22509323	0.809	0.883	0.24156902	6	Sh3bgrl
Ppp2r5c	1.34E-05	0.25793187	0.542	0.552	0.2483158	6	Ppp2r5c
Dkk3	1.39E-05	0.20364512	0.203	0.143	0.25831876	6	Dkk3
Phf12	1.40E-05	0.21375241	0.303	0.249	0.26097302	6	Phf12
Syvn11	1.42E-05	0.22370078	0.291	0.232	0.26474456	6	Syvn1
Ppp1cb	1.52E-05	0.27593282	0.492	0.491	0.28156439	6	Ppp1cb
Ash1l	1.56E-05	0.25327945	0.45	0.431	0.2896396	6	Ash1l
Slc9a9	1.57E-05	0.23056789	0.301	0.247	0.29244514	6	Slc9a9
Tmem184b	1.65E-05	0.24383619	0.341	0.291	0.30569933	6	Tmem184b
Dclre1c	1.65E-05	0.25804561	0.253	0.195	0.30572158	6	Dclre1c
Chd2	1.65E-05	0.30790722	0.52	0.523	0.30584357	6	Chd2
Eif5b	1.65E-05	0.20506117	0.817	0.842	0.3068478	6	Eif5b

Tmf1	1.68E-05	0.27538024	0.353	0.314	0.31185779	6	Tmf1
Itprid2	1.76E-05	0.31491324	0.424	0.394	0.3264907	6	Itprid2
Mapre2	1.76E-05	0.27211529	0.418	0.397	0.32671453	6	Mapre2
Sesn2	1.80E-05	0.25669186	0.275	0.222	0.33472242	6	Sesn2
Appbp2	1.81E-05	0.2320367	0.335	0.287	0.33648581	6	Appbp2
Stk38l1	1.82E-05	0.2302614	0.309	0.249	0.33769992	6	Stk38l
Adcy7	1.94E-05	0.31991955	0.38	0.347	0.36034946	6	Adcy7
Xylt1	1.95E-05	0.20527492	0.201	0.144	0.36295101	6	Xylt1
Supt16	1.96E-05	0.30207292	0.524	0.543	0.36398221	6	Supt16
Csf2ra	1.97E-05	0.33595268	0.783	0.867	0.36512897	6	Csf2ra
Palld	2.09E-05	0.20509564	0.217	0.157	0.388317	6	Palld
Nras	2.11E-05	0.26372423	0.41	0.391	0.392702	6	Nras
Map3k11	2.15E-05	0.21412485	0.231	0.175	0.39870817	6	Map3k11
Tnfaip3	2.15E-05	0.25144861	0.365	0.312	0.39926829	6	Tnfaip3
Rnf20	2.19E-05	0.29133842	0.424	0.4	0.4065537	6	Rnf20
Slc41a1	2.25E-05	0.21314741	0.257	0.2	0.41835951	6	Slc41a1
Pak2	2.27E-05	0.2121261	0.855	0.916	0.42166221	6	Pak2
Gtf3c2	2.27E-05	0.25120693	0.263	0.212	0.42210151	6	Gtf3c2
Eif2s3x	2.32E-05	0.29257461	0.456	0.437	0.43130227	6	Eif2s3x
Tlk2	2.32E-05	0.23195399	0.333	0.283	0.43191972	6	Tlk2
Cdk12	2.36E-05	0.29736535	0.506	0.506	0.4387764	6	Cdk12
Thap12	2.46E-05	0.39148663	0.351	0.308	0.45714694	6	Thap12
Havcr22	2.55E-05	0.35292453	0.681	0.698	0.47318025	6	Havcr2

Arhgef2	2.57E-05	0.24011469	0.339	0.293	0.47800002	6	Arhgef2
Fam117b	2.63E-05	0.2704462	0.426	0.399	0.48945681	6	Fam117b
Atp6v1b2	2.67E-05	0.27630546	0.4	0.376	0.49552311	6	Atp6v1b2
Sdcbp	2.77E-05	0.24903929	0.799	0.862	0.51385555	6	Sdcbp
Braf	2.78E-05	0.2253421	0.275	0.223	0.51637852	6	Braf
Hnrnpc	2.84E-05	0.20140227	0.817	0.89	0.52761597	6	Hnrnpc
Slc33a1	2.84E-05	0.21355017	0.273	0.225	0.5284698	6	Slc33a1
Kdm5c	2.89E-05	0.20647096	0.319	0.271	0.53733969	6	Kdm5c
Pik3c2a	2.93E-05	0.38592298	0.416	0.396	0.54402738	6	Pik3c2a
Uso11	3.11E-05	0.27763136	0.396	0.363	0.57750584	6	Uso1
Bdp1	3.23E-05	0.26599095	0.464	0.445	0.59982566	6	Bdp1
Zfp263	3.25E-05	0.24345037	0.321	0.27	0.60362156	6	Zfp263
Sh3tc1	3.30E-05	0.25426226	0.299	0.25	0.61402103	6	Sh3tc1
Ilrun	3.37E-05	0.22802411	0.416	0.388	0.62648295	6	Ilrun
Atxn2l	3.42E-05	0.27552332	0.55	0.559	0.63544431	6	Atxn2l
Csnk2a2	3.44E-05	0.23465472	0.295	0.25	0.63998583	6	Csnk2a2
Ankfy1	3.50E-05	0.30807646	0.378	0.346	0.65028367	6	Ankfy1
Lpgat1	3.54E-05	0.26405829	0.456	0.442	0.65773367	6	Lpgat1
Hivep2	3.56E-05	0.368759	0.598	0.626	0.66123622	6	Hivep2
Acer3	3.58E-05	0.32963449	0.544	0.566	0.66528812	6	Acer3
Mctp1	3.67E-05	0.31645246	0.552	0.59	0.68102309	6	Mctp1
Man2b11	3.68E-05	0.34196504	0.904	0.925	0.68412198	6	Man2b1
Pafah1b1	3.93E-05	0.22305384	0.833	0.902	0.73044839	6	Pafah1b1

Klhl24	4.00E-05	0.24865829	0.307	0.258	0.74356017	6	Klhl24
Rab31	4.00E-05	0.2103497	0.255	0.202	0.74399106	6	Rab31
Krt80	4.10E-05	0.22378649	0.253	0.198	0.76240897	6	Krt80
Fam149a	4.11E-05	0.2325375	0.345	0.3	0.76416373	6	Fam149a
Crebrf	4.29E-05	0.34654612	0.333	0.292	0.79699704	6	Crebrf
Raph1	4.36E-05	0.24064141	0.243	0.192	0.81033484	6	Raph1
Sun2	4.44E-05	0.28913818	0.353	0.317	0.82475469	6	Sun2
Ehmt2	4.44E-05	0.23016418	0.297	0.253	0.82539586	6	Ehmt2
Srsf1	4.47E-05	0.25769853	0.434	0.42	0.83053049	6	Srsf1
Rp2	4.69E-05	0.28857784	0.484	0.492	0.87088728	6	Rp2
Mark2	4.78E-05	0.25703624	0.426	0.408	0.88762528	6	Mark2
Itpk1	4.88E-05	0.25925119	0.331	0.294	0.90640297	6	Itpk1
Scap	4.90E-05	0.23349158	0.211	0.161	0.90970038	6	Scap
Dennd4b	4.90E-05	0.23504531	0.373	0.342	0.9098981	6	Dennd4b
Jup	4.95E-05	0.25062129	0.317	0.269	0.91980015	6	Jup
Tmem8	5.09E-05	0.20150896	0.309	0.26	0.94589493	6	Tmem8
Coro1c	5.40E-05	0.24868591	0.43	0.412	1	6	Coro1c
Smc41	5.46E-05	0.273321	0.494	0.478	1	6	Smc4
Prex1	5.46E-05	0.21409756	0.51	0.508	1	6	Prex1
Dido1	5.59E-05	0.23587016	0.287	0.238	1	6	Dido1
Lcorl	5.59E-05	0.28178333	0.297	0.252	1	6	Lcorl
Znfx1	5.67E-05	0.20008437	0.345	0.301	1	6	Znfx1
Gxylt1	5.79E-05	0.24004865	0.259	0.21	1	6	Gxylt1

Usp12	5.80E-05	0.23247586	0.478	0.468	1	6	Usp12
Kctd10	5.80E-05	0.21619959	0.341	0.299	1	6	Kctd10
Usp19	5.95E-05	0.27350824	0.418	0.403	1	6	Usp19
Zfp638	6.07E-05	0.27609666	0.458	0.442	1	6	Zfp638
Fmnl11	6.12E-05	0.22135788	0.596	0.625	1	6	Fmnl1
Kif3b	6.15E-05	0.24743264	0.315	0.271	1	6	Kif3b
Pdpk1	6.16E-05	0.22373223	0.279	0.232	1	6	Pdpk1
Ubr4	6.25E-05	0.28768811	0.392	0.375	1	6	Ubr4
Ski	6.33E-05	0.23737065	0.315	0.27	1	6	Ski
Vcpip1	6.47E-05	0.26444765	0.382	0.344	1	6	Vcpip1
Ahctf1	6.49E-05	0.23187664	0.315	0.269	1	6	Ahctf1
Lbr1	6.55E-05	0.24255102	0.384	0.348	1	6	Lbr
Iqgap21	6.71E-05	0.24717526	0.598	0.614	1	6	Iqgap2
Preb	6.82E-05	0.24086298	0.444	0.44	1	6	Preb
Stk10	6.86E-05	0.30105194	0.371	0.339	1	6	Stk10
Flii	7.00E-05	0.25731425	0.448	0.444	1	6	Flii
Tnks2	7.15E-05	0.29015188	0.414	0.4	1	6	Tnks2
Rab6a	7.40E-05	0.21302282	0.472	0.472	1	6	Rab6a
Zcchc7	7.43E-05	0.32045053	0.576	0.607	1	6	Zcchc7
Rap1gds1	7.48E-05	0.29514531	0.353	0.315	1	6	Rap1gds1
Bst1	7.57E-05	0.20592652	0.235	0.183	1	6	Bst1
Sik3	7.75E-05	0.26881847	0.311	0.27	1	6	Sik3
Dhx16	7.85E-05	0.20891925	0.267	0.214	1	6	Dhx16

Map3k2	7.86E-05	0.29991314	0.337	0.304	1	6	Map3k2
Frmd4b	7.99E-05	0.209946	0.203	0.15	1	6	Frmd4b
Sptan1	8.07E-05	0.28065792	0.392	0.368	1	6	Sptan1
Scaper	8.15E-05	0.21982744	0.235	0.184	1	6	Scaper
Ier31	8.19E-05	0.33981589	0.436	0.385	1	6	Ier3
Mllt10	8.23E-05	0.2158007	0.361	0.328	1	6	Mllt10
Kif5b	8.26E-05	0.22106687	0.717	0.771	1	6	Kif5b
Nsd3	8.39E-05	0.2145313	0.631	0.669	1	6	Nsd3
Elovl5	8.43E-05	0.243836	0.384	0.349	1	6	Elovl5
Bmpr2	8.77E-05	0.22544543	0.231	0.178	1	6	Bmpr2
Setd5	9.17E-05	0.28742627	0.408	0.388	1	6	Setd5
Ptprs	9.22E-05	0.20682346	0.263	0.213	1	6	Ptprs
Grk6	9.23E-05	0.21353878	0.363	0.331	1	6	Grk6
Gm35853	9.29E-05	0.21849645	0.376	0.335	1	6	Gm35853
Gmip	9.36E-05	0.26025552	0.408	0.392	1	6	Gmip
mt-Nd1	9.42E-05	0.42237468	0.96	0.988	1	6	mt-Nd1
Atp6v1h	9.72E-05	0.28953601	0.472	0.473	1	6	Atp6v1h
Add1	0.00010283	0.2350682	0.424	0.409	1	6	Add1
Uhmk1	0.00010396	0.20829484	0.253	0.204	1	6	Uhmk1
Stx16	0.00010431	0.25932356	0.494	0.485	1	6	Stx16
Rbbp6	0.00010521	0.29879148	0.622	0.673	1	6	Rbbp6
Fbxw7	0.00010713	0.21747677	0.267	0.226	1	6	Fbxw7
Fermt3	0.00010843	0.21954941	0.713	0.775	1	6	Fermt3

Rictor	0.00011137	0.24071827	0.388	0.355	1	6	Rictor
Brwd3	0.00011254	0.20910169	0.203	0.153	1	6	Brwd3
Ppp1r101	0.00011868	0.29583634	0.53	0.523	1	6	Ppp1r10
Cdc42se2	0.00012276	0.30046543	0.596	0.641	1	6	Cdc42se2
Cpsf6	0.00012374	0.31917244	0.345	0.323	1	6	Cpsf6
Rnf31	0.00012656	0.20716886	0.209	0.158	1	6	Rnf31
Tox4	0.00012664	0.23261213	0.669	0.706	1	6	Tox4
Mink1	0.00012993	0.24635347	0.386	0.353	1	6	Mink1
Lemd3	0.00013589	0.20443611	0.263	0.219	1	6	Lemd3
Tmem189	0.00014963	0.27689699	0.414	0.407	1	6	Tmem189
Ctnnb1	0.00015034	0.27355729	0.462	0.466	1	6	Ctnnb1
Tcirg1	0.0001511	0.28204337	0.329	0.296	1	6	Tcirg1
Ccpg1	0.00015798	0.23305248	0.259	0.213	1	6	Ccpg1
Nfkb2	0.00016052	0.22138431	0.424	0.408	1	6	Nfkb2
St3gal1	0.00017188	0.25901238	0.359	0.335	1	6	St3gal1
Ccnl2	0.00017244	0.25174026	0.492	0.508	1	6	Ccnl2
Psme3	0.0001752	0.23882564	0.384	0.362	1	6	Psme3
Dhx38	0.00017583	0.20298777	0.311	0.267	1	6	Dhx38
Ddx211	0.00018	0.26863676	0.741	0.797	1	6	Ddx21
Spop	0.00018777	0.21052242	0.717	0.791	1	6	Spop
Adpgk	0.00018942	0.24070642	0.287	0.248	1	6	Adpgk
Trpc4ap	0.00019151	0.2490377	0.552	0.597	1	6	Trpc4ap
Adam19	0.00019257	0.27174362	0.43	0.397	1	6	Adam19

Ncoa3	0.00019515	0.28760704	0.484	0.493	1	6	Ncoa3
Abca3	0.00020065	0.25050877	0.359	0.334	1	6	Abca3
Itgav	0.00020162	0.24755769	0.253	0.209	1	6	Itgav
Gusb	0.00021287	0.29923358	0.681	0.764	1	6	Gusb
Vcl	0.00021853	0.29059592	0.321	0.283	1	6	Vcl
Ciz1	0.00021888	0.21524193	0.261	0.217	1	6	Ciz1
Mtmr12	0.00021968	0.2167559	0.233	0.185	1	6	Mtmr12
Prkcsh	0.00021993	0.22136303	0.355	0.323	1	6	Prkcsh
Ythdf1	0.00022313	0.21208891	0.343	0.319	1	6	Ythdf1
Rab1a	0.00022642	0.2880391	0.532	0.563	1	6	Rab1a
1810013L24Rik	0.00024012	0.27927733	0.382	0.358	1	6	1810013L24Rik
Sgk3	0.00024435	0.25774807	0.532	0.564	1	6	Sgk3
Cdc73	0.00024539	0.22274336	0.311	0.27	1	6	Cdc73
Tbxas1	0.00024862	0.27408019	0.359	0.327	1	6	Tbxas1
Acly	0.00027145	0.20917729	0.378	0.351	1	6	Acly
Riok1	0.00027783	0.29319997	0.392	0.381	1	6	Riok1
Rock2	0.00028171	0.27034192	0.432	0.427	1	6	Rock2
Grap2	0.00028535	0.26128668	0.404	0.399	1	6	Grap2
Hectd1	0.00030241	0.24935259	0.512	0.532	1	6	Hectd1
Atp2c1	0.00030439	0.22528358	0.367	0.34	1	6	Atp2c1
Aagab	0.00032754	0.23821588	0.331	0.3	1	6	Aagab
Ankle2	0.00034733	0.23489223	0.357	0.325	1	6	Ankle2
Npepps	0.00034773	0.21286961	0.285	0.248	1	6	Npepps

1700025G04Rik	0.00035299	0.25323701	0.562	0.615	1	6	1700025G04Rik
Card10	0.00035953	0.20199168	0.201	0.155	1	6	Card10
Irak1	0.00036018	0.27116619	0.444	0.444	1	6	Irak1
Tspan13	0.00036806	0.27115876	0.661	0.733	1	6	Tspan13
Fzd1	0.00037799	0.23559342	0.233	0.188	1	6	Fzd1
Rabgef1	0.00037883	0.20666655	0.261	0.219	1	6	Rabgef1
Tmem106a	0.00038014	0.22942917	0.418	0.397	1	6	Tmem106a
Rgs3	0.00039889	0.24053843	0.305	0.27	1	6	Rgs3
Fbxw11	0.00040155	0.23975091	0.412	0.392	1	6	Fbxw11
Ifnar1	0.00040297	0.28269232	0.556	0.57	1	6	Ifnar1
Med1	0.00040562	0.22973506	0.349	0.325	1	6	Med1
Lasp1	0.00041392	0.24096621	0.458	0.464	1	6	Lasp1
Nptn	0.00042871	0.25487129	0.669	0.737	1	6	Nptn
Adipor1	0.00044725	0.24173363	0.464	0.478	1	6	Adipor1
Naa15	0.00044898	0.28698935	0.482	0.491	1	6	Naa15
Dnajc13	0.00047121	0.26089406	0.345	0.316	1	6	Dnajc13
Arhgap451	0.00047641	0.24471102	0.562	0.601	1	6	Arhgap45
Ankrd13c	0.00047713	0.21856401	0.281	0.247	1	6	Ankrd13c
Trim25	0.00047955	0.26740524	0.604	0.614	1	6	Trim25
Zfp869	0.00048405	0.2089502	0.299	0.255	1	6	Zfp869
Mbd2	0.00049488	0.20365257	0.426	0.414	1	6	Mbd2
Rnf216	0.00052652	0.24703354	0.349	0.335	1	6	Rnf216
Hnrnpll	0.00052725	0.2364285	0.333	0.31	1	6	Hnrnpll

Mafg	0.00053833	0.24373872	0.388	0.372	1	6	Mafg
Tug1	0.00054124	0.23980897	0.323	0.296	1	6	Tug1
Secisbp2l	0.00054324	0.26139606	0.357	0.337	1	6	Secisbp2l
Map7d1	0.00054569	0.20436641	0.321	0.287	1	6	Map7d1
Nfatc1	0.00056263	0.20831795	0.313	0.279	1	6	Nfatc1
Cln3	0.00057336	0.23422416	0.317	0.284	1	6	Cln3
Mapk1	0.00058388	0.24501134	0.532	0.565	1	6	Mapk1
Tm9sf2	0.00060931	0.24886027	0.546	0.572	1	6	Tm9sf2
Gmfb	0.00060984	0.24741973	0.454	0.468	1	6	Gmfb
Sbno1	0.00062267	0.25252445	0.478	0.487	1	6	Sbno1
Atp13a3	0.00063264	0.22328809	0.343	0.312	1	6	Atp13a3
Cdipt	0.00064854	0.25612936	0.365	0.352	1	6	Cdipt
Slc3a2	0.00067251	0.23661043	0.863	0.9	1	6	Slc3a2
Khdc4	0.00067408	0.27058309	0.325	0.293	1	6	Khdc4
Usp16	0.00071968	0.2519227	0.464	0.475	1	6	Usp16
Ptpra	0.00073763	0.23377899	0.369	0.358	1	6	Ptpra
Lrmp	0.00073967	0.24795233	0.367	0.355	1	6	Lrmp
Smarca2	0.00074479	0.20174249	0.335	0.304	1	6	Smarca2
Srcap	0.00075535	0.27153843	0.313	0.287	1	6	Srcap
Tcerg1	0.00076045	0.26509776	0.438	0.44	1	6	Tcerg1
Rps6ka1	0.00076286	0.21410961	0.45	0.447	1	6	Rps6ka1
Tor1aip1	0.00077551	0.212828	0.765	0.836	1	6	Tor1aip1
Vps26a	0.00077735	0.2713286	0.456	0.474	1	6	Vps26a

Smarcd2	0.00077799	0.22415189	0.311	0.284	1	6	Smarcd2
Vsir	0.00078449	0.23245729	0.408	0.404	1	6	Vsir
Gabpb2	0.00080958	0.23948157	0.299	0.266	1	6	Gabpb2
Ankib1	0.00082146	0.20131033	0.293	0.26	1	6	Ankib1
Leng8	0.00083232	0.20985849	0.241	0.203	1	6	Leng8
Sf3b2	0.00083527	0.20622616	0.721	0.791	1	6	Sf3b2
Rb1cc1	0.00083602	0.22330966	0.349	0.329	1	6	Rb1cc1
Ankhd1	0.00083964	0.22624512	0.373	0.351	1	6	Ankhd1
Qtrt1	0.00085745	0.2343581	0.215	0.178	1	6	Qtrt1
Slco3a1	0.00085772	0.30653544	0.49	0.505	1	6	Slco3a1
Pitpnm1	0.00087352	0.21347526	0.251	0.217	1	6	Pitpnm1
Asap1	0.00091261	0.26083742	0.699	0.762	1	6	Asap1
Cnot6	0.00094238	0.24206185	0.382	0.373	1	6	Cnot6
Shc1	0.00094807	0.25548243	0.327	0.304	1	6	Shc1
Zeb1	0.00098354	0.24886996	0.422	0.409	1	6	Zeb1
Golga4	0.00104351	0.23953711	0.317	0.291	1	6	Golga4
Zswim8	0.00105425	0.22395238	0.285	0.253	1	6	Zswim8
Trim12c	0.00106558	0.29375695	0.394	0.387	1	6	Trim12c
Adam17	0.00107405	0.21624918	0.283	0.254	1	6	Adam17
Rara	0.00108806	0.25835338	0.5	0.525	1	6	Rara
Rad21	0.00109177	0.27220919	0.331	0.306	1	6	Rad21
Crem	0.00109457	0.26490802	0.584	0.612	1	6	Crem
AI467606	0.00110493	0.24643606	0.297	0.265	1	6	AI467606

Nup153	0.00110723	0.28686794	0.434	0.446	1	6	Nup153
Pid11	0.00112178	0.23792864	0.512	0.536	1	6	Pid1
Ehd4	0.00113343	0.22374546	0.55	0.594	1	6	Ehd4
Zc3h18	0.00116025	0.23905366	0.313	0.294	1	6	Zc3h18
Irf9	0.00117617	0.24964309	0.544	0.589	1	6	Irf9
Dhx401	0.00122187	0.21707124	0.303	0.271	1	6	Dhx40
Selenot	0.00128781	0.241501	0.514	0.55	1	6	Selenot
Sp3	0.00133524	0.22357389	0.319	0.296	1	6	Sp3
Stk24	0.00136131	0.22298317	0.542	0.58	1	6	Stk24
Pip5k1c	0.00136963	0.26297712	0.462	0.483	1	6	Pip5k1c
Nucks1	0.00139767	0.23106265	0.606	0.65	1	6	Nucks1
Smarca4	0.00143153	0.20508027	0.41	0.408	1	6	Smarca4
Sh2b3	0.00145576	0.25984542	0.42	0.414	1	6	Sh2b3
Pik3cg	0.0014628	0.27204097	0.398	0.401	1	6	Pik3cg
Snx10	0.00149012	0.29644096	0.522	0.552	1	6	Snx10
Chd7	0.00153316	0.30079356	0.552	0.582	1	6	Chd7
Ankrd12	0.00156162	0.28154968	0.594	0.621	1	6	Ankrd12
Acap2	0.00158171	0.23087542	0.442	0.463	1	6	Acap2
Ptpn7	0.0016217	0.20429388	0.289	0.261	1	6	Ptpn7
Tmem214	0.00162331	0.23204978	0.361	0.349	1	6	Tmem214
Vwa5a	0.00163357	0.21863358	0.337	0.313	1	6	Vwa5a
B4galt5	0.00163639	0.25323189	0.319	0.297	1	6	B4galt5
Gtf2h1	0.00167202	0.25406869	0.329	0.309	1	6	Gtf2h1

Rsf1	0.00168867	0.22756864	0.4	0.388	1	6	Rsf1
Slc25a28	0.00170298	0.26886004	0.279	0.253	1	6	Slc25a28
Plcg2	0.00183359	0.23835591	0.524	0.57	1	6	Plcg2
Lsr	0.00198888	0.26126235	0.448	0.462	1	6	Lsr
Rela	0.00203261	0.23743844	0.394	0.388	1	6	Rela
Mcmbp	0.00208484	0.22092168	0.38	0.373	1	6	Mcmbp
Maff	0.0020914	0.24336474	0.428	0.413	1	6	Maff
Wdr91	0.00209755	0.2020687	0.259	0.227	1	6	Wdr91
Dmxl1	0.00209759	0.2452655	0.331	0.317	1	6	Dmxl1
Ids	0.0021201	0.21501244	0.309	0.285	1	6	Ids
Il13ra1	0.00214281	0.24582806	0.303	0.278	1	6	Il13ra1
Cyld	0.00227229	0.20684431	0.528	0.545	1	6	Cyld
Cysltr1	0.00231984	0.25741129	0.494	0.505	1	6	Cysltr1
Rexo1	0.00234135	0.20462107	0.355	0.339	1	6	Rexo1
Srsf11	0.00236506	0.23818653	0.759	0.82	1	6	Srsf11
Slc25a24	0.00240396	0.20034991	0.378	0.361	1	6	Slc25a24
Mprip	0.00251352	0.21412373	0.313	0.298	1	6	Mprip
Cers5	0.00255412	0.20798375	0.323	0.305	1	6	Cers5
Coro7	0.00278979	0.21884245	0.386	0.392	1	6	Coro7
2510039O18Rik	0.0028675	0.21196862	0.247	0.219	1	6	2510039O18Rik
Klf7	0.00288986	0.21228063	0.265	0.234	1	6	Klf7
Resf1	0.00291247	0.2666771	0.482	0.487	1	6	Resf1
Acin1	0.00302548	0.20091352	0.665	0.735	1	6	Acin1

Zfp608	0.00321691	0.21530715	0.211	0.175	1	6	Zfp608
App	0.00321798	0.22082527	0.508	0.549	1	6	App
Hnrnpul1	0.00329453	0.25155379	0.422	0.44	1	6	Hnrnpul1
Galnt10	0.00330614	0.23647619	0.291	0.267	1	6	Galnt10
Trim28	0.00334462	0.2371114	0.357	0.357	1	6	Trim28
Nup88	0.00334471	0.21349644	0.357	0.346	1	6	Nup88
Kcnq1ot11	0.00336767	0.49822101	0.323	0.297	1	6	Kcnq1ot1
Ep400	0.00355385	0.24557257	0.418	0.422	1	6	Ep400
Rad23b	0.00359007	0.2492623	0.476	0.501	1	6	Rad23b
Myd881	0.00360267	0.22344721	0.627	0.68	1	6	Myd88
Mtdh	0.00367294	0.20212774	0.691	0.802	1	6	Mtdh
Ubr2	0.00368913	0.20742069	0.277	0.256	1	6	Ubr2
Cul1	0.00372541	0.24720859	0.498	0.531	1	6	Cul1
Atad2b	0.00379472	0.25585198	0.46	0.477	1	6	Atad2b
Zmynd8	0.00379872	0.21368606	0.327	0.305	1	6	Zmynd8
Cse1l	0.00381189	0.23220299	0.267	0.24	1	6	Cse1l
Akap11	0.00401029	0.21137959	0.237	0.208	1	6	Akap11
Piezo1	0.004132	0.20176166	0.301	0.281	1	6	Piezo1
Ptpre	0.00415298	0.20458182	0.721	0.789	1	6	Ptpre
Ptpa	0.004335	0.28067186	0.484	0.518	1	6	Ptpa
Chd9	0.00437189	0.21210991	0.323	0.31	1	6	Chd9
Cybc1	0.0043784	0.22143202	0.424	0.436	1	6	Cybc1
Ddx39b	0.00441215	0.26507239	0.655	0.734	1	6	Ddx39b

Ppip5k2	0.00442169	0.21465046	0.221	0.193	1	6	Ppip5k2
Cs	0.00445814	0.20939377	0.556	0.612	1	6	Cs
Stag1	0.00449751	0.27330334	0.444	0.472	1	6	Stag1
Ano6	0.00530976	0.20068744	0.241	0.212	1	6	Ano6
Ddx23	0.00547421	0.20508403	0.333	0.318	1	6	Ddx23
Tram11	0.00575277	0.22883787	0.59	0.632	1	6	Tram1
Suco	0.0057777	0.26409355	0.432	0.448	1	6	Suco
Atp6ap2	0.00579409	0.20860823	0.673	0.746	1	6	Atp6ap2
Exoc5	0.00581162	0.22784011	0.365	0.36	1	6	Exoc5
Kit	0.0058176	0.25644728	0.6	0.639	1	6	Kit
Grina	0.00602099	0.23106113	0.293	0.277	1	6	Grina
Cic	0.00611008	0.22039484	0.466	0.484	1	6	Cic
B4galnt11	0.00616148	0.21845357	0.426	0.446	1	6	B4galnt1
Gm20559	0.00639984	0.28230213	0.319	0.304	1	6	Gm20559
Ist1	0.00646294	0.22219334	0.498	0.529	1	6	Ist1
Lemd22	0.00648325	0.22322999	0.52	0.554	1	6	Lemd2
Tgfb11	0.00663971	0.20608814	0.639	0.713	1	6	Tgfb1
Foxn2	0.00672865	0.22940459	0.382	0.386	1	6	Foxn2
Ganab	0.00696937	0.20338601	0.295	0.278	1	6	Ganab
Akt2	0.00712754	0.22794543	0.299	0.285	1	6	Akt2
Oxsr1	0.00717531	0.21067202	0.293	0.279	1	6	Oxsr1
Zfp292	0.00723411	0.24385184	0.361	0.365	1	6	Zfp292
Arcn11	0.00740291	0.20528643	0.472	0.503	1	6	Arcn1

Synrg	0.007616	0.24581487	0.438	0.464	1	6	Synrg
Clasp1	0.00788532	0.20078082	0.289	0.27	1	6	Clasp1
Atp6ap1	0.008099	0.20992645	0.404	0.411	1	6	Atp6ap1
Rgs14	0.00865574	0.20923617	0.349	0.349	1	6	Rgs14
Ptger4	0.00867135	0.22997793	0.462	0.461	1	6	Ptger4
Gapvd1	0.00878816	0.21613988	0.365	0.363	1	6	Gapvd1
Nab1	0.00892275	0.21203115	0.434	0.454	1	6	Nab1
Afdn	0.00911365	0.22325467	0.281	0.266	1	6	Afdn
Tor1b	0.00919863	0.246548	0.41	0.417	1	6	Tor1b
Galnt11	0.00925528	0.27244469	0.422	0.436	1	6	Galnt11
Usf2	0.00972086	0.20174303	0.478	0.511	1	6	Usf2
Ifit2	0	1.90150097	0.659	0.072	0	7	Ifit2
Isg20	2.57E-297	1.76090494	0.865	0.173	4.77E-293	7	Isg20
Rsad2	9.45E-288	1.69914503	0.706	0.097	1.76E-283	7	Rsad2
Ifi213	1.03E-285	0.89694982	0.585	0.058	1.91E-281	7	Ifi213
Ifit3	6.36E-274	1.26074632	0.358	0.014	1.18E-269	7	Ifit3
Ifi47	2.49E-270	1.32229435	0.709	0.106	4.62E-266	7	Ifi47
Ifi204	5.67E-268	1.7620789	0.915	0.238	1.05E-263	7	Ifi204
Isg15	4.16E-261	2.86191608	0.976	0.379	7.73E-257	7	Isg15
Irf7	1.71E-238	2.46421635	0.998	0.549	3.18E-234	7	Irf7
Oasl2	3.66E-228	0.73000657	0.495	0.05	6.79E-224	7	Oasl2
Oasl1	7.97E-214	1.03723961	0.559	0.075	1.48E-209	7	Oasl1
Zbp1	1.73E-199	1.55822929	0.964	0.474	3.22E-195	7	Zbp1

Usp18	2.99E-194	1.61466053	0.841	0.273	5.55E-190	7	Usp18
Slfn1	3.36E-187	1.10975616	0.647	0.131	6.24E-183	7	Slfn1
Oas3	2.97E-178	0.86219678	0.618	0.121	5.51E-174	7	Oas3
Slfn51	1.67E-176	2.05692063	0.991	0.754	3.10E-172	7	Slfn5
Ifi44	2.21E-175	1.21876787	0.69	0.163	4.10E-171	7	Ifi44
Cdh15	3.69E-168	0.46454497	0.268	0.016	6.85E-164	7	Cdh15
Xaf1	7.77E-165	1.0877946	0.863	0.314	1.44E-160	7	Xaf1
Bst2	7.99E-159	1.34160756	0.993	0.879	1.48E-154	7	Bst2
Ms4a4c1	1.46E-157	1.48945763	0.735	0.213	2.72E-153	7	Ms4a4c
Rnf2131	2.11E-154	1.29439105	0.922	0.437	3.92E-150	7	Rnf213
Phf11d	3.33E-153	1.05003716	0.737	0.222	6.19E-149	7	Phf11d
Ifi209	3.43E-149	1.30803135	0.96	0.596	6.37E-145	7	Ifi209
Mndal	1.05E-148	1.41411983	0.991	0.777	1.96E-144	7	Mndal
Cmpk2	1.14E-148	0.62471724	0.389	0.05	2.12E-144	7	Cmpk2
Herc6	1.87E-148	0.85920231	0.654	0.165	3.48E-144	7	Herc6
Phf11b1	1.89E-147	1.26405318	0.976	0.759	3.52E-143	7	Phf11b
Ifih1	3.66E-144	1.00129385	0.637	0.163	6.81E-140	7	Ifih1
Ifit1	8.79E-140	0.59346801	0.23	0.014	1.63E-135	7	Ifit1
Pnp1	2.18E-139	1.48393712	0.967	0.759	4.06E-135	7	Pnp
Ifi211	3.06E-137	1.11690455	0.979	0.758	5.68E-133	7	Ifi211
Lgals9	1.22E-136	1.00858664	0.896	0.435	2.27E-132	7	Lgals9
Sct	7.62E-136	0.73219614	0.282	0.026	1.42E-131	7	Sct
Ifi205	1.60E-132	1.09628149	0.995	0.911	2.98E-128	7	Ifi205

Samhd1	1.76E-132	1.1533318	0.993	0.886	3.27E-128	7	Samhd1
Irgm1	7.07E-128	0.99056929	0.694	0.215	1.31E-123	7	Irgm1
Stat1	1.48E-127	1.2030228	0.962	0.649	2.74E-123	7	Stat1
Ifitm32	2.28E-126	1.36150662	0.998	0.916	4.24E-122	7	Ifitm3
Trim30a1	5.54E-121	0.9817315	0.991	0.824	1.03E-116	7	Trim30a
Tgtp2	9.50E-121	0.30574337	0.261	0.025	1.76E-116	7	Tgtp2
Tspo	5.61E-120	0.99690386	0.998	0.975	1.04E-115	7	Tspo
Ifi206	3.88E-119	0.29671322	0.201	0.013	7.21E-115	7	Ifi206
Ifi203	4.69E-118	1.29961474	0.953	0.712	8.71E-114	7	Ifi203
Ube2l6	7.50E-118	0.76282378	0.713	0.245	1.39E-113	7	Ube2l6
Ms4a6b1	1.19E-117	0.95766838	0.63	0.188	2.20E-113	7	Ms4a6b
Slfn2	1.62E-112	1.35883032	0.948	0.676	3.02E-108	7	Slfn2
Pml	1.05E-111	0.7491971	0.697	0.241	1.96E-107	7	Pml
Pttg1	1.25E-109	0.85488407	0.82	0.385	2.31E-105	7	Pttg1
Tor3a	2.76E-109	0.77902513	0.735	0.283	5.12E-105	7	Tor3a
Dhx58	3.52E-108	0.6557384	0.661	0.221	6.55E-104	7	Dhx58
Ifi207	5.04E-108	1.15976226	0.955	0.709	9.37E-104	7	Ifi207
Parp12	6.08E-103	0.68189344	0.666	0.236	1.13E-98	7	Parp12
Parp14	9.48E-101	1.03793996	0.72	0.299	1.76E-96	7	Parp14
Ddx58	1.88E-99	0.30459233	0.251	0.029	3.49E-95	7	Ddx58
Ifi35	5.35E-99	0.85215024	0.964	0.765	9.94E-95	7	Ifi35
Sp1001	4.75E-96	0.9014339	0.957	0.825	8.83E-92	7	Sp100
Ly6e3	3.34E-95	1.19287332	0.964	0.777	6.20E-91	7	Ly6e

Ly6a	6.96E-94	1.2893757	0.445	0.107	1.29E-89	7	Ly6a
Akr1a11	2.22E-93	0.80266778	0.981	0.922	4.13E-89	7	Akr1a1
Snx2	4.33E-93	0.88583395	0.924	0.633	8.04E-89	7	Snx2
Fcgr1	1.34E-92	0.62925456	0.325	0.057	2.48E-88	7	Fcgr1
Calhm6	4.41E-91	0.29872391	0.22	0.024	8.20E-87	7	Calhm6
Rasa4	4.85E-90	0.6503392	0.706	0.291	9.02E-86	7	Rasa4
Sp110	1.14E-89	0.80477078	0.957	0.719	2.12E-85	7	Sp110
Timeless	4.14E-88	0.7172533	0.668	0.259	7.70E-84	7	Timeless
Phf11a1	1.13E-87	0.8917721	0.898	0.573	2.10E-83	7	Phf11a
Atp6v1d	1.94E-86	0.94806044	0.903	0.644	3.60E-82	7	Atp6v1d
Trim30d	1.25E-85	0.72311475	0.865	0.478	2.32E-81	7	Trim30d
Stat21	2.14E-82	0.85148036	0.851	0.482	3.98E-78	7	Stat2
Slc25a22	1.49E-81	0.46727347	0.479	0.136	2.77E-77	7	Slc25a22
Cnp	2.50E-80	0.83601242	0.905	0.663	4.65E-76	7	Cnp
Nt5c3	1.23E-79	0.67258739	0.611	0.241	2.28E-75	7	Nt5c3
Eif2ak2	1.03E-78	0.80729454	0.77	0.393	1.92E-74	7	Eif2ak2
Ccnd1	7.65E-78	0.88619612	0.969	0.828	1.42E-73	7	Ccnd1
Slfn8	3.14E-77	0.56420787	0.417	0.11	5.84E-73	7	Slfn8
A530064D06Rik	3.61E-75	0.37075816	0.358	0.081	6.71E-71	7	A530064D06Rik
Fam241a	2.22E-74	0.65287064	0.704	0.329	4.13E-70	7	Fam241a
Lgals3bp	2.25E-74	0.58300843	0.737	0.337	4.18E-70	7	Lgals3bp
Usp251	6.89E-74	0.7511999	0.846	0.517	1.28E-69	7	Usp25
A930037H05Rik	2.20E-72	0.31609511	0.265	0.046	4.09E-68	7	A930037H05Rik

Cd40	6.95E-72	0.47846824	0.455	0.131	1.29E-67	7	Cd40
Ms4a6c1	1.16E-71	0.97567926	0.891	0.628	2.16E-67	7	Ms4a6c
Chmp4b	1.92E-71	0.61358588	0.981	0.928	3.58E-67	7	Chmp4b
Sap30	7.04E-71	0.74404804	0.787	0.469	1.31E-66	7	Sap30
Oas1a	1.14E-69	0.34584197	0.329	0.075	2.12E-65	7	Oas1a
Igtp	4.78E-69	0.34772191	0.351	0.083	8.88E-65	7	Igtp
Selenow	8.07E-69	0.68627757	0.976	0.917	1.50E-64	7	Selenow
Nmi	1.33E-68	0.70440326	0.832	0.498	2.47E-64	7	Nmi
Parp9	1.39E-66	0.53374588	0.59	0.234	2.59E-62	7	Parp9
Daxx	3.91E-66	0.58383598	0.633	0.276	7.26E-62	7	Daxx
Acadl	1.50E-65	0.77768296	0.96	0.798	2.78E-61	7	Acadl
Plac83	1.26E-64	1.23343113	0.903	0.727	2.34E-60	7	Plac8
Dtx3l	3.09E-64	0.65140938	0.732	0.375	5.75E-60	7	Dtx3l
Cct3	1.55E-63	0.68931685	0.945	0.771	2.88E-59	7	Cct3
H2-T23	1.89E-61	0.6001188	0.983	0.925	3.52E-57	7	H2-T23
Txn1	1.92E-61	0.90369175	0.983	0.933	3.56E-57	7	Txn1
Tmpo	2.79E-61	0.7195506	0.898	0.671	5.17E-57	7	Tmpo
1600014C10Rik	2.92E-61	0.38466877	0.429	0.135	5.43E-57	7	1600014C10Rik
Pik3ap11	4.44E-61	0.67376129	0.936	0.703	8.26E-57	7	Pik3ap1
Xdh	4.45E-61	0.28539277	0.263	0.053	8.26E-57	7	Xdh
Treml21	2.38E-59	0.41620614	0.436	0.14	4.42E-55	7	Treml2
B430306N03Rik	9.91E-59	0.37535405	0.464	0.157	1.84E-54	7	B430306N03Rik
Setdb2	5.30E-58	0.41993694	0.448	0.151	9.85E-54	7	Setdb2

Tor1aip11	5.37E-58	0.67397406	0.96	0.82	9.98E-54	7	Tor1aip1
Fbxw17	2.21E-57	0.34997276	0.441	0.148	4.12E-53	7	Fbxw17
Phf11c	4.34E-57	0.42531469	0.488	0.179	8.06E-53	7	Phf11c
Npc21	5.91E-57	0.53070564	0.993	0.981	1.10E-52	7	Npc2
Svbp1	6.03E-57	0.58406992	0.694	0.362	1.12E-52	7	Svbp
St8sia1	1.69E-55	0.27855853	0.289	0.069	3.14E-51	7	St8sia1
BC147527	2.54E-54	0.38105489	0.431	0.144	4.72E-50	7	BC147527
Ifi214	2.54E-54	0.37266702	0.441	0.153	4.73E-50	7	Ifi214
Samd9l1	4.21E-54	0.69351395	0.839	0.602	7.83E-50	7	Samd9l
Trafd1	4.62E-53	0.61838067	0.782	0.494	8.59E-49	7	Trafd1
Ndrg1	2.21E-52	0.61973072	0.671	0.346	4.10E-48	7	Ndrg1
Znfx11	3.08E-52	0.55744815	0.597	0.282	5.72E-48	7	Znfx1
Scimp1	3.78E-52	0.67753948	0.825	0.569	7.03E-48	7	Scimp
Nampt	9.56E-52	0.53850398	0.846	0.54	1.78E-47	7	Nampt
Ppa11	1.58E-51	0.65383096	0.773	0.492	2.94E-47	7	Ppa1
Gm205591	2.19E-49	0.46063728	0.602	0.282	4.06E-45	7	Gm20559
Sp140	2.25E-49	0.57015404	0.931	0.735	4.19E-45	7	Sp140
Prdx11	6.83E-49	0.6381971	0.979	0.915	1.27E-44	7	Prdx1
9930111J21Rik22	1.21E-48	0.67323545	0.72	0.413	2.26E-44	7	9930111J21Rik2
Ctla2b	1.66E-48	0.26842712	0.23	0.05	3.08E-44	7	Ctla2b
Dek	3.49E-48	0.62877349	0.976	0.897	6.49E-44	7	Dek
Tor1aip2	2.25E-47	0.56943908	0.678	0.38	4.18E-43	7	Tor1aip2
Frmd4a1	3.13E-47	0.50558085	0.656	0.35	5.82E-43	7	Frmd4a

Mitd1	4.77E-47	0.48526415	0.538	0.236	8.87E-43	7	Mitd1
Ogfr	1.62E-46	0.48415904	0.815	0.521	3.01E-42	7	Ogfr
Cxcl9	2.95E-46	1.40286848	0.327	0.1	5.49E-42	7	Cxcl9
Psma4	3.40E-46	0.48779595	0.979	0.922	6.32E-42	7	Psma4
Sgcb	8.64E-46	0.298184	0.28	0.077	1.60E-41	7	Sgcb
Pkib	1.74E-45	0.58427307	0.948	0.815	3.24E-41	7	Pkib
Helz2	4.60E-45	0.30340555	0.427	0.158	8.55E-41	7	Helz2
Taldo12	5.18E-45	0.49590968	0.995	0.967	9.62E-41	7	Taldo1
Ildr1	6.12E-45	0.4089254	0.479	0.198	1.14E-40	7	Ildr1
Calm1	1.01E-44	0.33811504	0.995	0.994	1.88E-40	7	Calm1
Cd47	2.39E-44	0.40300955	0.995	0.976	4.43E-40	7	Cd47
Cycs2	6.10E-44	0.57176516	0.969	0.902	1.13E-39	7	Cycs
Psmb92	1.01E-43	0.44097497	0.988	0.962	1.88E-39	7	Psmb9
Dck1	1.60E-43	0.59366433	0.765	0.486	2.96E-39	7	Dck
Epsti1	2.20E-43	0.41195863	0.917	0.733	4.08E-39	7	Epsti1
Il15	5.13E-43	0.24561645	0.275	0.076	9.52E-39	7	Il15
Anxa51	6.34E-43	0.60151903	0.955	0.883	1.18E-38	7	Anxa5
Zup1	9.88E-43	0.53557006	0.701	0.415	1.84E-38	7	Zup1
Casp4	1.19E-42	0.40124182	0.424	0.161	2.21E-38	7	Casp4
Tmem219	1.59E-42	0.49181139	0.808	0.556	2.95E-38	7	Tmem219
Nono	2.70E-42	0.52070832	0.893	0.742	5.02E-38	7	Nono
Sass6	3.30E-41	0.32975858	0.415	0.163	6.14E-37	7	Sass6
Jaml1	6.11E-41	0.56351673	0.969	0.897	1.14E-36	7	Jaml

Pgap2	6.90E-41	0.43176319	0.595	0.309	1.28E-36	7	Pgap2
Ptms2	6.55E-40	0.52728315	0.993	0.965	1.22E-35	7	Ptms
Parp10	8.83E-40	0.25939424	0.277	0.083	1.64E-35	7	Parp10
B2m	9.04E-40	0.37741597	1	0.985	1.68E-35	7	B2m
Psme1	2.78E-39	0.3347032	1	0.982	5.17E-35	7	Psme1
Dnajc7	9.90E-39	0.6059585	0.922	0.764	1.84E-34	7	Dnajc7
Bbx	1.59E-37	0.60470457	0.687	0.433	2.96E-33	7	Bbx
Actb	2.73E-37	0.28386281	1	1	5.07E-33	7	Actb
Ppm1k1	3.08E-37	0.48607646	0.486	0.227	5.72E-33	7	Ppm1k
Ostf1	3.17E-37	0.40086376	0.974	0.922	5.90E-33	7	Ostf1
Phyh	4.78E-37	0.44966448	0.754	0.491	8.89E-33	7	Phyh
Itm2b1	5.72E-37	0.38796874	0.995	0.973	1.06E-32	7	Itm2b
Asb13	6.11E-37	0.24345138	0.306	0.102	1.13E-32	7	Asb13
Stxbp3	6.12E-37	0.57194124	0.649	0.391	1.14E-32	7	Stxbp3
Fndc3a	2.53E-36	0.58595478	0.642	0.409	4.70E-32	7	Fndc3a
Arpc5l1	4.02E-36	0.52334251	0.889	0.768	7.47E-32	7	Arpc5l
Bri3	5.16E-36	0.44844202	0.891	0.742	9.59E-32	7	Bri3
Sh3glb1	5.57E-36	0.43530366	0.948	0.862	1.03E-31	7	Sh3glb1
Sco1	2.01E-35	0.28463683	0.289	0.097	3.74E-31	7	Sco1
Armcx6	4.36E-35	0.29054777	0.256	0.079	8.09E-31	7	Armcx6
Apobec3	8.36E-35	0.44142766	0.898	0.724	1.55E-30	7	Apobec3
Capza2	1.91E-34	0.36491096	0.988	0.956	3.56E-30	7	Capza2
Tmem184b1	4.61E-34	0.3269595	0.552	0.275	8.57E-30	7	Tmem184b

Rfc3	6.80E-33	0.31290461	0.348	0.139	1.26E-28	7	Rfc3
Mthfr	9.93E-33	0.28197135	0.372	0.151	1.84E-28	7	Mthfr
Mar-05	1.86E-32	0.52068134	0.652	0.427	3.45E-28	7	Mar-05
Cd69	3.39E-32	0.58093632	0.244	0.078	6.30E-28	7	Cd69
Aldh1b1	6.52E-32	0.22152467	0.244	0.077	1.21E-27	7	Aldh1b1
Gpr33	1.07E-31	0.25387276	0.275	0.094	1.99E-27	7	Gpr33
Dnaja11	1.12E-31	0.56538857	0.991	0.967	2.08E-27	7	Dnaja1
Ehd41	2.10E-31	0.44444607	0.796	0.574	3.91E-27	7	Ehd4
Tpst1	2.53E-31	0.30284549	0.355	0.146	4.71E-27	7	Tpst1
Parp11	5.32E-31	0.22960192	0.277	0.095	9.88E-27	7	Parp11
H2-T22	5.73E-31	0.41941086	0.63	0.386	1.07E-26	7	H2-T22
Rtraf	1.00E-30	0.40431514	0.962	0.876	1.86E-26	7	Rtraf
Rnh11	2.01E-30	0.45304689	0.974	0.898	3.73E-26	7	Rnh1
Fgl21	2.20E-30	0.56033615	0.945	0.828	4.08E-26	7	Fgl2
Arf4	2.78E-30	0.4237521	0.955	0.86	5.16E-26	7	Arf4
Klrk1	3.61E-30	0.48954813	0.898	0.743	6.70E-26	7	Klrk1
Gm21188	3.91E-30	0.27828551	0.341	0.136	7.27E-26	7	Gm21188
Lap3	4.20E-30	0.60268816	0.559	0.342	7.80E-26	7	Lap3
Dbnl	1.53E-29	0.38693459	0.976	0.915	2.85E-25	7	Dbnl
Trim30b	1.55E-29	0.30370578	0.436	0.211	2.87E-25	7	Trim30b
Trim34a	1.60E-29	0.2335778	0.329	0.129	2.97E-25	7	Trim34a
Anxa12	1.87E-29	0.43889778	0.962	0.877	3.47E-25	7	Anxa1
Aida	1.91E-29	0.30573466	0.431	0.206	3.55E-25	7	Aida

Stard3	2.62E-29	0.33711866	0.618	0.38	4.87E-25	7	Stard3
Tdrd7	3.04E-29	0.25646983	0.355	0.149	5.65E-25	7	Tdrd7
Ogfrl1	5.63E-29	0.4267382	0.673	0.45	1.05E-24	7	Ogfrl1
Max	6.41E-29	0.37091927	0.616	0.382	1.19E-24	7	Max
Ccdc86	1.58E-28	0.44210464	0.799	0.583	2.94E-24	7	Ccdc86
Adap2	2.02E-28	0.24677966	0.289	0.11	3.75E-24	7	Adap2
Rab29	5.20E-28	0.27018819	0.427	0.203	9.66E-24	7	Rab29
M6pr1	5.93E-28	0.449581	0.941	0.851	1.10E-23	7	M6pr
BC051226	7.67E-28	0.22619589	0.322	0.13	1.43E-23	7	BC051226
Tcf41	8.24E-28	0.39203507	0.531	0.29	1.53E-23	7	Tcf4
Sppl2a1	8.68E-28	0.4211256	0.903	0.764	1.61E-23	7	Sppl2a
Plin2	1.47E-27	0.41985968	0.87	0.683	2.72E-23	7	Plin2
Trim12a	1.85E-27	0.34097598	0.585	0.339	3.44E-23	7	Trim12a
Uba7	2.86E-27	0.24896916	0.412	0.188	5.32E-23	7	Uba7
Gm4070	6.77E-27	0.245896	0.344	0.145	1.26E-22	7	Gm4070
Mpp1	2.65E-26	0.23448614	0.31	0.128	4.92E-22	7	Mpp1
Ifi27l2a	3.30E-26	1.22393889	0.763	0.656	6.13E-22	7	Ifi27l2a
Cd86	3.69E-26	0.37301724	0.934	0.802	6.86E-22	7	Cd86
Larp1b	3.69E-26	0.33638149	0.569	0.331	6.86E-22	7	Larp1b
Dop1b	9.78E-26	0.23433838	0.377	0.171	1.82E-21	7	Dop1b
Il10ra1	2.20E-25	0.4185133	0.512	0.294	4.08E-21	7	Il10ra
Nsd31	2.22E-25	0.42195437	0.829	0.652	4.12E-21	7	Nsd3
Tmem131	2.58E-25	0.2652504	0.699	0.456	4.79E-21	7	Tmem131

Psmb10	2.58E-25	0.38583761	0.936	0.83	4.79E-21	7	Psmb10
Sdc31	4.01E-25	0.36722785	0.874	0.686	7.45E-21	7	Sdc3
Prpf38a	4.72E-25	0.33791222	0.642	0.425	8.76E-21	7	Prpf38a
Etnk1	4.89E-25	0.42178881	0.597	0.372	9.09E-21	7	Etnk1
Atp6v0a21	7.31E-25	0.39418697	0.848	0.691	1.36E-20	7	Atp6v0a2
Spon1	1.03E-24	0.24862082	0.244	0.089	1.92E-20	7	Spon1
Chmp5	1.14E-24	0.36494142	0.799	0.618	2.12E-20	7	Chmp5
Ppp1r2	1.45E-24	0.39565418	0.848	0.685	2.69E-20	7	Ppp1r2
Naa20	4.87E-24	0.38813802	0.498	0.292	9.04E-20	7	Naa20
Sema4f1	5.64E-24	0.27907552	0.429	0.223	1.05E-19	7	Sema4f
Gramd3	6.69E-24	0.38224274	0.616	0.392	1.24E-19	7	Gramd3
Tex9	1.23E-23	0.27632972	0.377	0.181	2.28E-19	7	Tex9
Clec2d	1.46E-23	0.37661988	0.623	0.403	2.72E-19	7	Clec2d
Cpne31	2.21E-23	0.43221041	0.867	0.738	4.12E-19	7	Cpne3
Usp221	3.52E-23	0.28538971	0.571	0.337	6.55E-19	7	Usp22
Acer31	3.62E-23	0.33463193	0.751	0.55	6.72E-19	7	Acer3
Adar1	4.05E-23	0.29607995	0.495	0.281	7.52E-19	7	Adar
Tapbp1	4.70E-23	0.31565254	0.957	0.879	8.72E-19	7	Tapbp
Nlrc5	1.07E-22	0.20977183	0.393	0.191	2.00E-18	7	Nlrc5
Mcmbp1	1.26E-22	0.32279787	0.588	0.356	2.35E-18	7	Mcmbp
Susd61	1.56E-22	0.36812199	0.706	0.516	2.90E-18	7	Susd6
Tomm70a	1.82E-22	0.3303916	0.69	0.483	3.38E-18	7	Tomm70a
Sdcbp1	1.88E-22	0.32750027	0.941	0.85	3.50E-18	7	Sdcbp

Sumo1	2.87E-22	0.35472381	0.903	0.808	5.34E-18	7	Sumo1
Rcn2	3.52E-22	0.33565597	0.592	0.377	6.55E-18	7	Rcn2
Anxa7	7.37E-22	0.33842582	0.768	0.591	1.37E-17	7	Anxa7
Evi2a	1.25E-21	0.32945393	0.929	0.789	2.33E-17	7	Evi2a
Psmb82	3.08E-21	0.26945097	0.998	0.986	5.72E-17	7	Psmb8
Pmepa11	3.94E-21	0.38580636	0.63	0.434	7.32E-17	7	Pmepa1
Ndufb1-ps2	4.18E-21	0.31305886	0.983	0.949	7.77E-17	7	Ndufb1-ps
Tmbim6	1.18E-20	0.28821947	0.972	0.931	2.19E-16	7	Tmbim6
Ass1	1.25E-20	0.37244476	0.754	0.545	2.31E-16	7	Ass1
Dnaja2	2.61E-20	0.35843531	0.825	0.664	4.84E-16	7	Dnaja2
Keap1	3.52E-20	0.23796478	0.445	0.241	6.54E-16	7	Keap1
Ascc3	4.68E-20	0.35902689	0.756	0.562	8.70E-16	7	Ascc3
Psme2	5.28E-20	0.22902321	0.979	0.94	9.81E-16	7	Psme2
Bag1	1.03E-19	0.32254989	0.877	0.757	1.92E-15	7	Bag1
Csrp1	1.64E-19	0.29353429	0.519	0.323	3.05E-15	7	Csrp1
Cyp27a1	2.88E-19	0.28563656	0.528	0.326	5.35E-15	7	Cyp27a1
Reep3	3.23E-19	0.32896249	0.791	0.622	6.01E-15	7	Reep3
Ank11	3.32E-19	0.20434365	0.315	0.149	6.17E-15	7	Ank1
B4galt51	3.84E-19	0.29525766	0.479	0.285	7.13E-15	7	B4galt5
Xrn1	3.86E-19	0.28209406	0.566	0.36	7.17E-15	7	Xrn1
Nmral1	9.84E-19	0.23922638	0.313	0.154	1.83E-14	7	Nmral1
Serf2	1.02E-18	0.21421381	0.998	0.984	1.90E-14	7	Serf2
A530032D15Rik	1.24E-18	0.2354205	0.379	0.198	2.30E-14	7	A530032D15Rik

Dcp2	1.68E-18	0.31039048	0.652	0.455	3.12E-14	7	Dcp2
Actr21	2.18E-18	0.25478815	0.983	0.947	4.06E-14	7	Actr2
Pstpip1	2.58E-18	0.32913535	0.765	0.597	4.79E-14	7	Pstpip1
S100a62	5.16E-18	0.38226738	0.957	0.882	9.58E-14	7	S100a6
Zyx1	8.77E-18	0.36535482	0.974	0.936	1.63E-13	7	Zyx
Ppp1r11	9.18E-18	0.29548703	0.957	0.873	1.71E-13	7	Ppp1r11
Dpy19l11	9.27E-18	0.33107647	0.68	0.486	1.72E-13	7	Dpy19l1
Gmppb1	1.01E-17	0.26408102	0.578	0.376	1.88E-13	7	Gmppb
Cited22	1.17E-17	0.48710572	0.642	0.448	2.18E-13	7	Cited2
Naa25	2.80E-17	0.21615996	0.431	0.244	5.20E-13	7	Naa25
Prdx21	4.05E-17	0.30784495	0.957	0.885	7.53E-13	7	Prdx2
AI6622701	4.26E-17	0.39197959	0.893	0.775	7.92E-13	7	AI662270
Morc3	4.53E-17	0.29657044	0.69	0.52	8.41E-13	7	Morc3
Ffar41	5.35E-17	0.3882199	0.467	0.296	9.94E-13	7	Ffar4
Vcpip11	9.40E-17	0.25387997	0.521	0.334	1.75E-12	7	Vcpip1
Gng12	1.11E-16	0.31847761	0.782	0.657	2.05E-12	7	Gng12
Arid4a1	1.18E-16	0.39088151	0.898	0.801	2.19E-12	7	Arid4a
Mgat1	1.45E-16	0.27081322	0.434	0.26	2.69E-12	7	Mgat1
Tbl1x	1.51E-16	0.23686961	0.571	0.377	2.80E-12	7	Tbl1x
Elac2	1.54E-16	0.2037482	0.391	0.218	2.86E-12	7	Elac2
Trim12c1	3.13E-16	0.28876085	0.555	0.374	5.81E-12	7	Trim12c
Ndufaf6	3.58E-16	0.2010408	0.365	0.199	6.66E-12	7	Ndufaf6
Ldha1	6.24E-16	0.24181105	0.993	0.967	1.16E-11	7	Ldha

Rab8a	9.66E-16	0.26021231	0.855	0.736	1.79E-11	7	Rab8a
Zc3hav11	9.83E-16	0.29401889	0.763	0.6	1.83E-11	7	Zc3hav1
Pigf	1.63E-15	0.20080353	0.474	0.296	3.04E-11	7	Pigf
Isoc1	2.23E-15	0.25990735	0.64	0.467	4.14E-11	7	Isoc1
Ints8	2.29E-15	0.20336007	0.277	0.14	4.26E-11	7	Ints8
Hmgn3	2.79E-15	0.24870174	0.569	0.394	5.19E-11	7	Hmgn3
Cd522	3.96E-15	0.26709948	1	0.993	7.36E-11	7	Cd52
Lpxn	6.57E-15	0.26467209	0.493	0.325	1.22E-10	7	Lpxn
Myd882	7.68E-15	0.25421633	0.825	0.664	1.43E-10	7	Myd88
D1Ertd622e	1.17E-14	0.28673453	0.521	0.362	2.16E-10	7	D1Ertd622e
Gtf3c6	1.22E-14	0.28042998	0.799	0.631	2.26E-10	7	Gtf3c6
Scarb2	1.23E-14	0.22428466	0.723	0.546	2.28E-10	7	Scarb2
Irf91	1.31E-14	0.25920643	0.756	0.572	2.44E-10	7	Irf9
Mycbp21	1.34E-14	0.33758025	0.787	0.641	2.48E-10	7	Mycbp2
Map3k8	1.59E-14	0.28750262	0.505	0.339	2.95E-10	7	Map3k8
Plaat3	1.92E-14	0.28125463	0.704	0.553	3.56E-10	7	Plaat3
Utp3	3.23E-14	0.26368557	0.713	0.558	5.99E-10	7	Utp3
Vdac3	3.70E-14	0.27814063	0.836	0.736	6.87E-10	7	Vdac3
Grn1	4.25E-14	0.24347941	0.891	0.766	7.89E-10	7	Grn
Slamf71	4.93E-14	0.25700561	0.941	0.848	9.17E-10	7	Slamf7
Gnb4	6.13E-14	0.21288987	0.455	0.295	1.14E-09	7	Gnb4
Tmsb103	6.89E-14	0.27429323	1	0.993	1.28E-09	7	Tmsb10
Rab11a	9.09E-14	0.25798169	0.884	0.806	1.69E-09	7	Rab11a

Psma51	9.69E-14	0.25233508	0.915	0.823	1.80E-09	7	Psma5
Hmox2	9.84E-14	0.2601182	0.777	0.624	1.83E-09	7	Hmox2
Casp3	1.10E-13	0.25894957	0.609	0.441	2.04E-09	7	Casp3
Nasp	1.10E-13	0.24813976	0.531	0.368	2.04E-09	7	Nasp
P4ha1	3.76E-13	0.20308095	0.415	0.258	6.99E-09	7	P4ha1
Mospd2	6.97E-13	0.23111092	0.6	0.418	1.29E-08	7	Mospd2
Crlf3	1.06E-12	0.20419927	0.616	0.448	1.97E-08	7	Crlf3
Tlk21	1.14E-12	0.21807686	0.427	0.277	2.12E-08	7	Tlk2
Slirp	1.32E-12	0.26314469	0.761	0.633	2.46E-08	7	Slirp
Gch11	1.35E-12	0.26025967	0.547	0.387	2.50E-08	7	Gch1
Sri1	1.52E-12	0.22737305	0.979	0.927	2.82E-08	7	Sri
Gpr141	1.54E-12	0.23370395	0.344	0.202	2.85E-08	7	Gpr141
Plcb4	1.58E-12	0.23459236	0.464	0.306	2.94E-08	7	Plcb4
P2ry141	1.69E-12	0.25147475	0.749	0.591	3.14E-08	7	P2ry14
Acaa1a	2.08E-12	0.22297024	0.784	0.646	3.87E-08	7	Acaa1a
Stx161	2.61E-12	0.21802721	0.628	0.474	4.85E-08	7	Stx16
Ptpn61	2.74E-12	0.25722706	0.813	0.687	5.09E-08	7	Ptpn6
Abcg1	3.05E-12	0.203684	0.5	0.339	5.66E-08	7	Abcg1
Lgals31	3.92E-12	0.32190804	0.998	0.976	7.28E-08	7	Lgals3
Fbrsl1	6.57E-12	0.2030677	0.467	0.314	1.22E-07	7	Fbrsl1
Dennd1b1	1.31E-11	0.29406088	0.713	0.595	2.44E-07	7	Dennd1b
Irf21	1.45E-11	0.24833839	0.756	0.632	2.69E-07	7	Irf2
Pfkp2	2.02E-11	0.24874562	0.945	0.883	3.75E-07	7	Pfkp

Ccnd21	3.31E-11	0.24325928	0.424	0.272	6.16E-07	7	Ccnd2
Fdps2	4.07E-11	0.2515863	0.618	0.446	7.57E-07	7	Fdps
Tmem106a1	4.09E-11	0.24993061	0.536	0.388	7.60E-07	7	Tmem106a
Fos3	5.59E-11	0.30991355	0.995	0.975	1.04E-06	7	Fos
Elf1	6.46E-11	0.27167481	0.666	0.531	1.20E-06	7	Elf1
Phf6	6.86E-11	0.20041598	0.386	0.248	1.27E-06	7	Phf6
Hdac1	1.05E-10	0.21611231	0.63	0.501	1.94E-06	7	Hdac1
Usp15	1.08E-10	0.23796083	0.664	0.525	2.01E-06	7	Usp15
Irf11	1.27E-10	0.20170138	0.481	0.322	2.35E-06	7	Irf1
G3bp2	1.81E-10	0.22459937	0.829	0.746	3.35E-06	7	G3bp2
Rbms11	1.86E-10	0.23473116	0.737	0.606	3.46E-06	7	Rbms1
Aif11	2.17E-10	0.23783733	0.979	0.925	4.04E-06	7	Aif1
Tap11	2.43E-10	0.23299429	0.945	0.89	4.52E-06	7	Tap1
Zfp800	3.31E-10	0.25902698	0.668	0.525	6.15E-06	7	Zfp800
Il182	7.26E-10	0.24866201	0.637	0.5	1.35E-05	7	Il18
Msn2	8.98E-10	0.21549137	0.953	0.921	1.67E-05	7	Msn
Themis21	1.04E-09	0.23423834	0.661	0.54	1.94E-05	7	Themis2
S100a111	1.46E-09	0.21911275	0.995	0.985	2.71E-05	7	S100a11
Selenot1	1.52E-09	0.21801328	0.659	0.539	2.82E-05	7	Selenot
Lnpep1	1.68E-09	0.23542549	0.68	0.544	3.12E-05	7	Lnpep
Xrn21	1.82E-09	0.20275641	0.889	0.815	3.38E-05	7	Xrn2
Rnf41	2.16E-09	0.21022482	0.735	0.593	4.01E-05	7	Rnf4
Hspa81	2.23E-09	0.31494735	0.998	0.996	4.14E-05	7	Hspa8

Shisa5	2.29E-09	0.20245789	0.896	0.793	4.25E-05	7	Shisa5
Ncoa72	2.30E-09	0.27465778	0.893	0.801	4.26E-05	7	Ncoa7
Uqcrb1	3.67E-09	0.21458212	0.962	0.914	6.81E-05	7	Uqcrb
Plekhf2	4.02E-09	0.21230539	0.512	0.379	7.47E-05	7	Plekhf2
Usp121	4.06E-09	0.205806	0.597	0.459	7.54E-05	7	Usp12
Atp5o1	4.20E-09	0.21015745	0.903	0.85	7.81E-05	7	Atp5o
Fcer1g1	5.20E-09	0.21398292	0.974	0.929	9.66E-05	7	Fcer1g
Cacybp	6.35E-09	0.22299471	0.803	0.714	0.00011804	7	Cacybp
Snx222	7.49E-09	0.240499	0.645	0.523	0.00013912	7	Snx22
Tlr32	1.54E-08	0.27196018	0.789	0.711	0.0002865	7	Tlr3
Socs1	1.62E-08	0.23233442	0.263	0.159	0.00030121	7	Socs1
Mxd11	1.93E-08	0.31877448	0.445	0.314	0.00035908	7	Mxd1
Atf31	2.08E-08	0.28952295	0.953	0.912	0.00038663	7	Atf3
Ccl41	1.60E-07	0.50363288	0.723	0.641	0.0029725	7	Ccl4
Atp1b31	2.42E-07	0.21290318	0.846	0.76	0.00449321	7	Atp1b3
Tuba1b	3.82E-07	0.21115814	0.777	0.687	0.00709761	7	Tuba1b
Spty2d12	7.11E-07	0.20245515	0.82	0.742	0.01321068	7	Spty2d1
Ikzf11	8.69E-07	0.22038647	0.664	0.582	0.01615029	7	Ikzf1
Clec9a1	1.64E-06	0.21203605	0.628	0.537	0.03052047	7	Clec9a
Hspa1a3	2.09E-06	0.36093805	0.739	0.633	0.03881435	7	Hspa1a
Hmgb21	1.21E-05	0.31921717	0.908	0.87	0.22418651	7	Hmgb2
H3f3b3	1.98E-05	0.21794137	1	0.994	0.36706407	7	H3f3b
Grasp1	3.06E-05	0.2262792	0.787	0.692	0.56867839	7	Grasp

Spp11	0.00055072	0.21846459	0.429	0.337	1	7	Spp1
Hsp90aa12	0.00088094	0.24758117	0.981	0.975	1	7	Hsp90aa1
Ccr7	0	4.56913441	0.827	0.015	0	8	Ccr7
Il12b	0	4.25355436	0.698	0.035	0	8	Il12b
Ccl22	0	4.04046222	0.78	0.035	0	8	Ccl22
Fscn1	0	3.43108811	0.475	0.006	0	8	Fscn1
Cd63	0	3.02908026	0.624	0.019	0	8	Cd63
Serpinb6b	0	2.96846912	0.671	0.046	0	8	Serpinb6b
Serpinb9	0	2.8610836	0.792	0.08	0	8	Serpinb9
Cacnb3	0	2.7272618	0.686	0.055	0	8	Cacnb3
Il4i1	0	2.40810203	0.635	0.013	0	8	Il4i1
Arl5c	0	2.18531095	0.671	0.049	0	8	Arl5c
Socs2	0	2.00204491	0.596	0.007	0	8	Socs2
Tnfrsf4	0	1.45823595	0.333	0.005	0	8	Tnfrsf4
Mreg	0	1.28580419	0.506	0.001	0	8	Mreg
Ankrd33b	0	1.15694204	0.412	0.001	0	8	Ankrd33b
Atxn1	0	1.11083584	0.353	0.002	0	8	Atxn1
Nudt17	0	1.0496181	0.341	0.002	0	8	Nudt17
Pakap.1	0	0.99699582	0.306	0.001	0	8	Pakap.1
Snn	0	0.84762627	0.42	0.003	0	8	Snn
Nipal1	0	0.60361506	0.294	0.002	0	8	Nipal1
Anxa3	9.90E-307	1.8218716	0.482	0.02	1.84E-302	8	Anxa3
Adcy6	8.70E-290	1.4719923	0.561	0.034	1.62E-285	8	Adcy6

Npr1	8.33E-250	0.73283825	0.251	0.003	1.55E-245	8	Npr1
Tmem150c	1.95E-245	0.62343961	0.212	0.001	3.62E-241	8	Tmem150c
Cx3cl1	1.64E-234	0.5862063	0.31	0.008	3.06E-230	8	Cx3cl1
Gm13546	8.77E-233	1.05981567	0.439	0.024	1.63E-228	8	Gm13546
Mmp25	1.39E-228	1.8472548	0.588	0.055	2.58E-224	8	Mmp25
Mab21l3	5.43E-226	0.56877492	0.286	0.007	1.01E-221	8	Mab21l3
Stap2	9.31E-223	0.48373222	0.204	0.001	1.73E-218	8	Stap2
Flrt3	8.89E-222	1.49012334	0.353	0.014	1.65E-217	8	Flrt3
Cd274	1.00E-220	2.00746188	0.753	0.11	1.86E-216	8	Cd274
Gm47662	7.29E-216	0.85295725	0.208	0.002	1.35E-211	8	Gm47662
Pdcd1lg2	3.82E-212	0.98793796	0.447	0.03	7.10E-208	8	Pdcd1lg2
Rgs12	8.02E-207	0.62017443	0.345	0.015	1.49E-202	8	Rgs12
Mir155hg	3.19E-205	0.92559061	0.353	0.016	5.92E-201	8	Mir155hg
Adora2a	6.66E-192	0.8729792	0.369	0.021	1.24E-187	8	Adora2a
Stat4	4.69E-191	2.81784941	0.745	0.131	8.72E-187	8	Stat4
Samsn1	3.37E-188	3.77536514	0.886	0.235	6.27E-184	8	Samsn1
Zmynd15	6.47E-186	1.67253028	0.373	0.023	1.20E-181	8	Zmynd15
Pdlim4	2.17E-182	0.92420153	0.282	0.011	4.04E-178	8	Pdlim4
Il7r	3.30E-175	1.01584717	0.325	0.017	6.13E-171	8	Il7r
Ramp3	6.37E-171	2.63563866	0.627	0.097	1.18E-166	8	Ramp3
Spred1	1.31E-170	1.24553185	0.549	0.066	2.44E-166	8	Spred1
Cd80	3.75E-170	1.73309391	0.769	0.155	6.98E-166	8	Cd80
Relb	1.60E-169	2.41765153	0.882	0.261	2.98E-165	8	Relb

Insl6	1.16E-167	0.55342622	0.224	0.006	2.15E-163	8	Insl6
Foxp4	2.18E-167	0.87358124	0.408	0.033	4.04E-163	8	Foxp4
Il2rg	4.46E-167	1.22250943	0.608	0.087	8.29E-163	8	Il2rg
Sema7a	8.18E-166	1.06745324	0.514	0.057	1.52E-161	8	Sema7a
Arpin	1.81E-155	0.38310532	0.243	0.009	3.35E-151	8	Arpin
Ccser2	1.54E-153	1.52151413	0.635	0.107	2.86E-149	8	Ccser2
Il15ra	3.81E-141	1.15406269	0.4	0.04	7.08E-137	8	Il15ra
Arc	1.85E-136	0.4664955	0.235	0.011	3.44E-132	8	Arc
Traf1	2.24E-136	2.56732581	0.961	0.617	4.16E-132	8	Traf1
Ly75	1.32E-132	1.98709776	0.773	0.223	2.46E-128	8	Ly75
Cd401	2.33E-131	1.57148246	0.651	0.131	4.32E-127	8	Cd40
Arhgap28	2.54E-131	0.4427075	0.212	0.008	4.72E-127	8	Arhgap28
Dnajc12	5.98E-131	0.49482578	0.247	0.013	1.11E-126	8	Dnajc12
Abtb2	1.42E-129	0.59354341	0.251	0.014	2.63E-125	8	Abtb2
Fam177a	1.49E-126	1.09801204	0.545	0.094	2.78E-122	8	Fam177a
Ccl5	3.59E-125	4.802528	0.62	0.134	6.67E-121	8	Ccl5
Gadd45b1	1.05E-124	2.71010279	0.929	0.468	1.94E-120	8	Gadd45b
Adgrg6	1.45E-124	0.59334852	0.247	0.014	2.69E-120	8	Adgrg6
Rftn1	3.51E-122	1.33614979	0.624	0.132	6.53E-118	8	Rftn1
Map4k41	2.37E-118	2.60345332	0.953	0.611	4.40E-114	8	Map4k4
Tmem123	3.25E-118	3.07953886	0.871	0.417	6.05E-114	8	Tmem123
Tnip3	1.53E-117	1.62511502	0.624	0.136	2.85E-113	8	Tnip3
Scin	1.60E-116	1.48032993	0.357	0.039	2.98E-112	8	Scin

H2-Eb2	6.22E-116	0.96065093	0.408	0.052	1.16E-111	8	H2-Eb2
Poglut1	1.39E-115	1.18380985	0.565	0.11	2.58E-111	8	Poglut1
Fas	3.96E-113	0.57826099	0.278	0.022	7.36E-109	8	Fas
Eno3	7.11E-113	1.27427803	0.482	0.079	1.32E-108	8	Eno3
Castor2	1.80E-111	0.84673306	0.322	0.032	3.35E-107	8	Castor2
Rel1	4.15E-111	2.00623437	0.98	0.892	7.71E-107	8	Rel
Plxnc1	5.17E-111	1.61915263	0.682	0.182	9.61E-107	8	Plxnc1
Tbc1d4	1.00E-110	2.67497655	0.937	0.605	1.86E-106	8	Tbc1d4
Basp1	4.62E-110	2.35734504	0.929	0.683	8.59E-106	8	Basp1
Swap70	1.23E-107	1.87283758	0.737	0.246	2.28E-103	8	Swap70
Bcl2a1b	2.97E-107	2.90723958	0.839	0.401	5.52E-103	8	Bcl2a1b
Il21r	1.20E-106	1.17155212	0.525	0.1	2.23E-102	8	Il21r
Ccl17	4.87E-106	2.30391138	0.345	0.039	9.04E-102	8	Ccl17
Adap1	1.98E-105	0.94957216	0.471	0.08	3.68E-101	8	Adap1
Pcgf5	3.70E-104	2.06359595	0.753	0.27	6.88E-100	8	Pcgf5
Bcl2l14	9.07E-104	2.06336307	0.635	0.17	1.69E-99	8	Bcl2l14
Marcksl1	1.64E-103	2.28671906	0.843	0.387	3.04E-99	8	Marcksl1
Myo1g	2.17E-103	2.02665724	0.918	0.617	4.04E-99	8	Myo1g
Tank	1.28E-102	1.37186273	0.667	0.183	2.37E-98	8	Tank
4930523C07Rik	2.88E-102	1.71648115	0.824	0.301	5.36E-98	8	4930523C07Rik
Tmcc3	5.06E-101	1.33814198	0.471	0.083	9.41E-97	8	Tmcc3
Gbp5	6.97E-100	0.57516597	0.31	0.032	1.29E-95	8	Gbp5
Rras2	7.05E-100	0.56701324	0.373	0.049	1.31E-95	8	Rras2

Rab8b	4.81E-99	1.84029421	0.957	0.871	8.94E-95	8	Rab8b
Nfkbia1	5.35E-99	2.15617615	0.933	0.698	9.95E-95	8	Nfkbia
Cmc2	1.57E-98	1.42338105	0.553	0.13	2.91E-94	8	Cmc2
Bhlhe40	7.96E-96	1.80466472	0.878	0.477	1.48E-91	8	Bhlhe40
Gypc	3.12E-95	0.52190909	0.29	0.03	5.80E-91	8	Gypc
Map3k14	3.53E-95	1.67483666	0.824	0.384	6.56E-91	8	Map3k14
Nfkb21	6.47E-95	1.41448158	0.839	0.39	1.20E-90	8	Nfkb2
Actn1	6.52E-95	1.32349456	0.427	0.073	1.21E-90	8	Actn1
Strip2	3.72E-94	1.43275584	0.616	0.166	6.91E-90	8	Strip2
H2-K1	6.98E-94	1.56901901	0.984	0.991	1.30E-89	8	H2-K1
Cxcl161	7.20E-93	1.91963475	0.871	0.579	1.34E-88	8	Cxcl16
Fam49a	1.79E-91	1.09007257	0.533	0.121	3.33E-87	8	Fam49a
Cflar	5.13E-89	1.45425457	0.682	0.227	9.54E-85	8	Cflar
Gem	8.48E-89	0.66210831	0.345	0.047	1.58E-84	8	Gem
Pik3r11	8.54E-89	2.07892965	0.831	0.418	1.59E-84	8	Pik3r1
Rogdi	1.06E-88	1.59733924	0.647	0.207	1.97E-84	8	Rogdi
Prnp	3.57E-87	0.98308796	0.263	0.027	6.64E-83	8	Prnp
Cblb	4.84E-87	1.63638407	0.788	0.352	8.99E-83	8	Cblb
Cd200	7.67E-87	1.64475814	0.514	0.12	1.42E-82	8	Cd200
Zc3h12c	2.36E-86	1.32995208	0.6	0.161	4.38E-82	8	Zc3h12c
Clic41	3.17E-86	1.63473116	0.937	0.748	5.90E-82	8	Clic4
Oasl11	9.31E-86	1.08754473	0.475	0.093	1.73E-81	8	Oasl1
Etv31	4.47E-84	1.70189924	0.949	0.779	8.31E-80	8	Etv3

Ggta1	1.83E-83	1.0025203	0.6	0.17	3.41E-79	8	Ggta1
Slc27a3	2.14E-82	1.26403577	0.396	0.072	3.97E-78	8	Slc27a3
Mfhas1	2.59E-82	0.83096356	0.396	0.068	4.81E-78	8	Mfhas1
Gfpt1	2.76E-82	1.59803806	0.82	0.425	5.12E-78	8	Gfpt1
Rap2a	2.07E-81	1.27421676	0.769	0.333	3.85E-77	8	Rap2a
N4bp2l1	2.40E-80	1.24748541	0.635	0.21	4.46E-76	8	N4bp2l1
Birc2	4.77E-80	1.62599994	0.631	0.203	8.86E-76	8	Birc2
Gbp2	5.01E-80	0.65073312	0.259	0.028	9.32E-76	8	Gbp2
Tmem176a1	7.09E-80	1.69665219	0.431	0.088	1.32E-75	8	Tmem176a
Hivep1	7.68E-80	1.7442947	0.796	0.384	1.43E-75	8	Hivep1
Pi4k2b	1.57E-78	0.89675984	0.42	0.083	2.92E-74	8	Pi4k2b
Marcks1	3.71E-78	1.42062968	0.961	0.928	6.90E-74	8	Marcks
H2-Q6	5.85E-77	1.50441453	0.702	0.264	1.09E-72	8	H2-Q6
Spsb1	6.63E-77	0.68793074	0.271	0.033	1.23E-72	8	Spsb1
Fgfbp3	4.63E-75	0.76388675	0.239	0.026	8.61E-71	8	Fgfbp3
Tmem39a	6.22E-75	1.39589201	0.592	0.186	1.16E-70	8	Tmem39a
Laptm4b	6.87E-75	1.30509102	0.62	0.207	1.28E-70	8	Laptm4b
Mxd12	4.01E-74	1.67514773	0.722	0.306	7.45E-70	8	Mxd1
Rgs11	1.15E-73	2.13733926	0.929	0.613	2.14E-69	8	Rgs1
Gca	5.60E-73	0.40972696	0.235	0.026	1.04E-68	8	Gca
Castor1	1.84E-72	0.45729394	0.22	0.022	3.43E-68	8	Castor1
Glipr2	2.10E-71	2.13029303	0.62	0.233	3.90E-67	8	Glipr2
Rasa2	2.39E-71	1.36606506	0.549	0.163	4.45E-67	8	Rasa2

Rhoc	6.50E-71	0.46872636	0.263	0.034	1.21E-66	8	Rhoc
Tmem63b	1.03E-70	0.66970426	0.329	0.054	1.92E-66	8	Tmem63b
Csrp11	1.28E-69	1.68431819	0.698	0.321	2.38E-65	8	Csrp1
Hsf2	1.80E-69	0.92623735	0.357	0.066	3.35E-65	8	Hsf2
Nudt9	1.81E-69	1.73433296	0.749	0.403	3.37E-65	8	Nudt9
Tmem131l1	2.88E-69	1.45950346	0.788	0.409	5.34E-65	8	Tmem131l
B2m1	8.50E-69	1.40115418	0.937	0.988	1.58E-64	8	B2m
Ccrl2	1.72E-68	1.08112461	0.463	0.112	3.19E-64	8	Ccrl2
Arhgap31	5.83E-68	1.80540082	0.725	0.343	1.08E-63	8	Arhgap31
Ssh1	7.28E-68	0.86738017	0.416	0.093	1.35E-63	8	Ssh1
Ktn1	3.22E-67	2.50415595	0.757	0.422	5.99E-63	8	Ktn1
Specc1	4.21E-67	1.97212636	0.71	0.32	7.83E-63	8	Specc1
Tmem1311	9.90E-67	1.58486383	0.8	0.458	1.84E-62	8	Tmem131
H2-Q7	1.57E-66	1.90924164	0.835	0.546	2.92E-62	8	H2-Q7
Itga41	1.70E-66	1.92544491	0.898	0.7	3.16E-62	8	Itga4
Malt1	1.35E-65	1.78465893	0.698	0.297	2.51E-61	8	Malt1
Aebp21	2.44E-65	1.45476983	0.769	0.396	4.53E-61	8	Aebp2
Mex3b	4.86E-65	0.59148235	0.29	0.045	9.03E-61	8	Mex3b
Sdc4	5.36E-65	0.56090758	0.259	0.035	9.96E-61	8	Sdc4
Sdhaf1	5.34E-64	0.68491055	0.494	0.138	9.92E-60	8	Sdhaf1
Syngr2	2.19E-63	1.24483476	0.894	0.959	4.08E-59	8	Syngr2
Tspan3	3.43E-63	1.54730254	0.494	0.146	6.38E-59	8	Tspan3
Lactb	8.65E-63	1.60913676	0.816	0.583	1.61E-58	8	Lactb

Lgmn2	2.13E-62	1.51444546	0.588	0.217	3.95E-58	8	Lgmn
Procr	5.76E-62	0.42084522	0.227	0.028	1.07E-57	8	Procr
Ric1	5.78E-61	1.1119551	0.51	0.159	1.07E-56	8	Ric1
Ncoa73	1.70E-60	1.46451719	0.918	0.803	3.15E-56	8	Ncoa7
Sqstm1	1.71E-60	1.63421726	0.82	0.628	3.18E-56	8	Sqstm1
Gm12216	5.14E-60	0.71128247	0.224	0.029	9.54E-56	8	Gm12216
Zfp36l12	5.59E-60	1.628459	0.89	0.71	1.04E-55	8	Zfp36l1
Tmem176b1	2.60E-59	1.3280521	0.408	0.101	4.83E-55	8	Tmem176b
C9orf72	3.51E-59	0.88711859	0.522	0.164	6.51E-55	8	C9orf72
Anxa4	3.05E-58	1.03645007	0.631	0.273	5.67E-54	8	Anxa4
Mif4gd	3.48E-58	1.23095124	0.718	0.369	6.47E-54	8	Mif4gd
Nlrc51	4.90E-58	1.05585271	0.557	0.189	9.11E-54	8	Nlrc5
Rassf21	6.83E-58	1.19659336	0.62	0.245	1.27E-53	8	Rassf2
Inf2	1.07E-57	0.73674706	0.384	0.088	1.99E-53	8	Inf2
Kcnk6	2.10E-57	0.67601998	0.431	0.112	3.90E-53	8	Kcnk6
Stx11	3.62E-57	0.60664785	0.404	0.097	6.73E-53	8	Stx11
Cdkn1a1	3.82E-57	1.69620797	0.776	0.453	7.10E-53	8	Cdkn1a
Ptafr	7.95E-57	1.04903709	0.545	0.186	1.48E-52	8	Ptafr
Nfkbie	1.42E-56	1.19404061	0.722	0.358	2.64E-52	8	Nfkbie
Mcemp1	1.54E-55	0.49528462	0.255	0.04	2.87E-51	8	Mcemp1
Nup85	1.63E-55	0.43927847	0.361	0.08	3.03E-51	8	Nup85
Fnbp1l	3.71E-55	1.2171397	0.655	0.28	6.89E-51	8	Fnbp1l
Nfkbib	1.37E-54	1.25396165	0.769	0.519	2.54E-50	8	Nfkbib

Lima11	3.17E-54	1.65310523	0.729	0.394	5.88E-50	8	Lima1
Lrrc32	4.32E-54	0.4678463	0.204	0.026	8.02E-50	8	Lrrc32
Malat12	8.39E-54	1.56390255	1	1	1.56E-49	8	Malat1
Uap1	1.02E-53	1.46305672	0.561	0.215	1.90E-49	8	Uap1
Rabgap1l	3.16E-53	1.19003905	0.643	0.276	5.88E-49	8	Rabgap1l
Htra2	5.04E-53	0.8184291	0.498	0.166	9.37E-49	8	Htra2
Slc4a8	5.88E-53	0.98127485	0.231	0.035	1.09E-48	8	Slc4a8
Bcl2a1d1	1.82E-52	1.50196812	0.792	0.662	3.37E-48	8	Bcl2a1d
Iscu	3.23E-52	1.43960681	0.769	0.579	6.00E-48	8	Iscu
H2-Q4	5.08E-52	1.33224103	0.627	0.283	9.45E-48	8	H2-Q4
H2-D1	5.75E-52	0.95171601	0.984	0.998	1.07E-47	8	H2-D1
Wnt11	2.44E-51	0.67070324	0.322	0.068	4.52E-47	8	Wnt11
Axl	6.25E-51	0.4192445	0.22	0.032	1.16E-46	8	Axl
Jak21	1.87E-50	1.25497906	0.886	0.749	3.47E-46	8	Jak2
Rps27l1	2.18E-50	1.6910138	0.867	0.927	4.05E-46	8	Rps27l
Iffo2	3.04E-50	0.70069167	0.353	0.086	5.65E-46	8	Iffo2
Itgb11	4.97E-49	1.11487708	0.906	0.905	9.23E-45	8	Itgb1
Dip2b	5.13E-49	1.29705081	0.631	0.296	9.54E-45	8	Dip2b
Cers6	1.23E-48	0.94764421	0.486	0.166	2.28E-44	8	Cers6
Birc3	1.50E-48	0.97922084	0.639	0.288	2.79E-44	8	Birc3
Lrrk1	3.59E-48	1.28455843	0.733	0.427	6.68E-44	8	Lrrk1
Bcl2a1a1	1.19E-47	1.432442	0.686	0.385	2.22E-43	8	Bcl2a1a
Dusp51	2.76E-47	1.31046365	0.878	0.805	5.12E-43	8	Dusp5

Grk31	3.26E-47	1.43426672	0.773	0.455	6.05E-43	8	Grk3
Irf12	3.33E-47	1.24511208	0.663	0.319	6.19E-43	8	Irf1
Rassf3	7.10E-47	1.20389605	0.639	0.307	1.32E-42	8	Rassf3
Vopp1	2.42E-46	0.45539293	0.239	0.042	4.50E-42	8	Vopp1
Nr4a31	4.42E-46	1.42879176	0.867	0.705	8.21E-42	8	Nr4a3
Gucd1	5.45E-46	0.53346967	0.251	0.047	1.01E-41	8	Gucd1
Chpt1	9.16E-46	0.75945012	0.31	0.072	1.70E-41	8	Chpt1
Ehd1	1.20E-45	0.9148246	0.584	0.262	2.22E-41	8	Ehd1
AA4671971	1.33E-45	1.4513948	0.439	0.145	2.48E-41	8	AA467197
Arf41	1.36E-45	1.03435514	0.894	0.866	2.53E-41	8	Arf4
Adam191	1.61E-45	1.16728612	0.694	0.387	2.99E-41	8	Adam19
Sh3bp4	1.72E-45	0.42049903	0.302	0.066	3.20E-41	8	Sh3bp4
Fabp5	6.50E-45	1.78107733	0.286	0.064	1.21E-40	8	Fabp5
Agap1	1.05E-44	0.48070078	0.208	0.033	1.95E-40	8	Agap1
Rrad	1.18E-44	1.41218646	0.478	0.165	2.18E-40	8	Rrad
Ccnd22	1.29E-44	1.80659825	0.58	0.269	2.39E-40	8	Ccnd2
Plekhg2	1.79E-44	0.72801517	0.298	0.066	3.32E-40	8	Plekhg2
Clec2d1	2.08E-44	1.23347068	0.714	0.405	3.86E-40	8	Clec2d
Psme21	5.31E-44	1.31290303	0.875	0.946	9.88E-40	8	Psme2
Mllt6	7.56E-44	0.79795181	0.455	0.151	1.41E-39	8	Mllt6
Klf63	1.68E-43	1.06292402	0.957	0.926	3.11E-39	8	Klf6
Tgif1	3.09E-43	0.74985376	0.518	0.197	5.75E-39	8	Tgif1
Rap2b	3.13E-42	1.07916073	0.475	0.182	5.81E-38	8	Rap2b

Sinhcaf	3.32E-42	1.12093801	0.722	0.466	6.17E-38	8	Sinhcaf
Gga2	4.09E-42	0.57249027	0.22	0.039	7.59E-38	8	Gga2
Slc2a6	4.85E-42	0.42424359	0.255	0.052	9.01E-38	8	Slc2a6
Stat5a	5.40E-42	0.94589356	0.463	0.166	1.00E-37	8	Stat5a
Stxbp31	3.15E-41	1.10216031	0.69	0.397	5.85E-37	8	Stxbp3
Tec	3.68E-41	0.57521902	0.341	0.089	6.84E-37	8	Tec
Gpr1321	5.22E-41	1.10037128	0.859	0.695	9.70E-37	8	Gpr132
Plekhm2	6.04E-41	0.70080294	0.38	0.114	1.12E-36	8	Plekhm2
Stk4	6.44E-41	1.17434869	0.769	0.584	1.20E-36	8	Stk4
Ptger41	1.92E-40	1.09800965	0.737	0.449	3.57E-36	8	Ptger4
Tmtc2	4.34E-39	0.73145724	0.298	0.075	8.06E-35	8	Tmtc2
Tnip1	4.36E-39	0.62300223	0.396	0.127	8.10E-35	8	Tnip1
Osm	6.49E-39	0.47172539	0.212	0.039	1.21E-34	8	Osm
Atmin	6.72E-39	0.72150687	0.416	0.144	1.25E-34	8	Atmin
AW112010	6.91E-39	2.53428248	0.69	0.479	1.28E-34	8	AW112010
Tuba1a	7.10E-38	1.74637751	0.773	0.672	1.32E-33	8	Tuba1a
Tnfrsf11a	1.32E-37	0.66569835	0.365	0.115	2.46E-33	8	Tnfrsf11a
Nabp11	1.43E-37	1.22520921	0.788	0.586	2.65E-33	8	Nabp1
Rcsd1	2.83E-37	1.06957917	0.478	0.198	5.27E-33	8	Rcsd1
Kif21b	3.25E-37	0.81519578	0.451	0.166	6.04E-33	8	Kif21b
Gm38115	1.36E-36	0.6811341	0.396	0.133	2.53E-32	8	Gm38115
Slc22a23	1.41E-36	0.69304889	0.267	0.066	2.62E-32	8	Slc22a23
Gnb41	3.67E-36	1.6037661	0.553	0.295	6.82E-32	8	Gnb4

Gm37529	4.27E-36	0.51493641	0.208	0.041	7.93E-32	8	Gm37529
Tubb2b	5.69E-36	0.75064371	0.322	0.093	1.06E-31	8	Tubb2b
Nfkb11	6.17E-36	1.42226477	0.769	0.583	1.15E-31	8	Nfkb1
Galnt7	7.92E-36	0.81234373	0.545	0.247	1.47E-31	8	Galnt7
Map3k12	1.96E-35	1.19700896	0.761	0.55	3.64E-31	8	Map3k1
Arap2	3.99E-35	1.05240747	0.369	0.121	7.42E-31	8	Arap2
Eva1b	9.33E-35	0.97548627	0.529	0.254	1.73E-30	8	Eva1b
Kpna3	1.62E-34	0.9100814	0.525	0.255	3.01E-30	8	Kpna3
Smarce1	2.64E-34	1.29126164	0.733	0.581	4.90E-30	8	Smarce1
Sbf2	2.64E-34	0.66006421	0.282	0.075	4.91E-30	8	Sbf2
Lilrb4a1	4.58E-34	1.05148194	0.529	0.242	8.52E-30	8	Lilrb4a
Tpm1	6.08E-34	1.03887982	0.486	0.221	1.13E-29	8	Tpm1
Rnf115	7.25E-34	1.01147411	0.604	0.343	1.35E-29	8	Rnf115
Zdhhc14	7.29E-34	0.37279224	0.259	0.063	1.36E-29	8	Zdhhc14
Tnfaip31	1.76E-33	1.05142664	0.612	0.303	3.27E-29	8	Tnfaip3
Lilr4b	5.56E-33	1.06484833	0.529	0.245	1.03E-28	8	Lilr4b
Tes1	2.19E-32	0.92448516	0.757	0.613	4.08E-28	8	Tes
Dleu2	2.79E-32	1.41934773	0.847	0.705	5.19E-28	8	Dleu2
Tnfsf9	3.60E-32	0.73743351	0.369	0.126	6.69E-28	8	Tnfsf9
Uvrag1	5.97E-32	0.99209576	0.867	0.808	1.11E-27	8	Uvrag
Cd831	1.26E-31	0.81842028	0.914	0.961	2.34E-27	8	Cd83
Smchd11	1.53E-31	1.39741584	0.808	0.735	2.85E-27	8	Smchd1
Dnajc10	1.78E-31	0.80026681	0.584	0.317	3.31E-27	8	Dnajc10

Ctnna11	3.05E-31	1.42104021	0.867	0.821	5.67E-27	8	Ctnna1
Map4	3.14E-31	0.94160721	0.592	0.337	5.84E-27	8	Map4
Ddx61	5.58E-31	1.12046063	0.827	0.753	1.04E-26	8	Ddx6
Rab21	6.38E-31	0.92770415	0.616	0.369	1.18E-26	8	Rab21
Gngt21	6.48E-31	0.80889557	0.373	0.135	1.20E-26	8	Gngt2
Atp6v0c	8.30E-31	1.04467798	0.839	0.901	1.54E-26	8	Atp6v0c
Smurf1	1.03E-30	0.51406536	0.302	0.091	1.91E-26	8	Smurf1
Sowahc	1.14E-30	0.85032466	0.647	0.371	2.11E-26	8	Sowahc
Avpi1	1.86E-30	1.05544572	0.725	0.572	3.45E-26	8	Avpi1
Csf2rb1	3.27E-30	1.07427333	0.808	0.698	6.07E-26	8	Csf2rb
Btg12	4.22E-30	0.63619571	0.957	0.99	7.83E-26	8	Btg1
Mar-51	4.59E-30	0.87166915	0.647	0.434	8.52E-26	8	Mar-05
Lpp1	5.14E-30	0.84205823	0.447	0.19	9.55E-26	8	Lpp
Selplg	1.25E-29	0.82430835	0.863	0.903	2.32E-25	8	Selplg
Txndc17	1.28E-29	1.94157587	0.784	0.819	2.38E-25	8	Txndc17
Nfe2l22	1.36E-29	0.91516906	0.82	0.711	2.53E-25	8	Nfe2l2
Epb411	2.47E-29	1.16421562	0.643	0.397	4.58E-25	8	Epb41
Bmp2k	5.35E-29	1.43545066	0.663	0.46	9.94E-25	8	Bmp2k
Tnfaip22	5.69E-29	0.81061748	0.482	0.209	1.06E-24	8	Tnfaip2
Txnrd1	9.82E-29	0.97238714	0.584	0.345	1.82E-24	8	Txnrd1
Traf3	1.05E-28	0.71002641	0.353	0.125	1.94E-24	8	Traf3
Iqgap11	1.09E-28	0.91599662	0.961	0.967	2.03E-24	8	Iqgap1
Dok1	2.76E-28	0.56257697	0.447	0.198	5.13E-24	8	Dok1

Id21	4.30E-28	0.98459434	0.882	0.902	7.99E-24	8	Id2
Cacna1s	5.90E-28	0.634165	0.455	0.211	1.10E-23	8	Cacna1s
Arhgef40	6.45E-28	0.79963956	0.357	0.131	1.20E-23	8	Arhgef40
Clip2	9.68E-28	0.40688213	0.271	0.079	1.80E-23	8	Clip2
Ifrd12	1.11E-27	0.91835775	0.847	0.777	2.05E-23	8	Ifrd1
Lamp11	2.20E-27	1.28267327	0.647	0.497	4.08E-23	8	Lamp1
Adam81	3.08E-27	1.03914154	0.757	0.6	5.72E-23	8	Adam8
Icam11	4.11E-27	0.78107788	0.741	0.529	7.64E-23	8	Icam1
Etv61	5.67E-27	0.83832176	0.808	0.656	1.05E-22	8	Etv6
Nampt1	5.88E-27	1.08585267	0.71	0.556	1.09E-22	8	Nampt
Mrtfa	6.19E-27	0.92679384	0.58	0.339	1.15E-22	8	Mrtfa
Slc44a1	7.45E-27	0.4867846	0.255	0.075	1.38E-22	8	Slc44a1
Serpinb1a	1.43E-26	1.21285075	0.22	0.058	2.66E-22	8	Serpinb1a
Epb41l2	1.63E-26	0.84647907	0.569	0.327	3.02E-22	8	Epb41l2
Scpep11	1.95E-26	0.80376797	0.588	0.352	3.62E-22	8	Scpep1
Pfkfb3	2.12E-26	1.26850117	0.502	0.277	3.93E-22	8	Pfkfb3
Herpud11	3.38E-26	0.85250268	0.796	0.673	6.27E-22	8	Herpud1
4833407H14Rik	4.49E-26	0.47219281	0.329	0.117	8.34E-22	8	4833407H14Rik
Gpr55	1.26E-25	0.37829775	0.251	0.073	2.34E-21	8	Gpr55
Bri31	2.67E-25	0.8868075	0.753	0.752	4.96E-21	8	Bri3
Ninj1	3.00E-25	0.34910284	0.298	0.101	5.57E-21	8	Ninj1
Tmem19	3.67E-25	0.55583465	0.302	0.106	6.82E-21	8	Tmem19
Ftl11	4.00E-25	0.85204649	0.922	0.996	7.43E-21	8	Ftl1

Wnk11	4.72E-25	1.13330354	0.929	0.928	8.77E-21	8	Wnk1
Akap131	6.59E-25	1.09960989	0.89	0.851	1.22E-20	8	Akap13
Spint2	1.10E-24	0.7481216	0.624	0.422	2.05E-20	8	Spint2
Arl5a	1.59E-24	0.73532066	0.58	0.361	2.95E-20	8	Arl5a
Tcf7l2	2.30E-24	0.57637126	0.4	0.168	4.28E-20	8	Tcf7l2
Cxcr4	3.42E-24	0.50122573	0.314	0.111	6.36E-20	8	Cxcr4
Tnfaip82	4.04E-24	0.73882433	0.855	0.867	7.51E-20	8	Tnfaip8
Zfc3h1	4.32E-24	1.06787098	0.463	0.23	8.02E-20	8	Zfc3h1
Pcyt1a	4.77E-24	0.80295038	0.353	0.146	8.86E-20	8	Pcyt1a
Gclc	6.84E-24	0.60927653	0.247	0.078	1.27E-19	8	Gclc
Actb1	7.54E-24	0.41321826	0.996	1	1.40E-19	8	Actb
Bclaf3	9.42E-24	0.67522866	0.275	0.095	1.75E-19	8	Bclaf3
Tbc1d15	1.95E-23	0.62615683	0.584	0.368	3.63E-19	8	Tbc1d15
Pim11	3.14E-23	0.63940632	0.949	0.975	5.84E-19	8	Pim1
Ubxn2a	3.21E-23	0.8328116	0.596	0.407	5.96E-19	8	Ubxn2a
Csf2rb2	3.62E-23	0.54009079	0.369	0.153	6.72E-19	8	Csf2rb2
Litaf1	6.71E-23	0.68996173	0.776	0.7	1.25E-18	8	Litaf
Ncf1	1.07E-22	0.82897371	0.698	0.562	1.99E-18	8	Ncf1
Net1	1.09E-22	1.24334613	0.612	0.435	2.02E-18	8	Net1
Tmem168	1.17E-22	0.71847371	0.51	0.279	2.17E-18	8	Tmem168
Gyg	1.19E-22	1.29687256	0.663	0.51	2.21E-18	8	Gyg
Atl31	1.98E-22	0.70482202	0.435	0.215	3.68E-18	8	Atl3
Gpr137b	2.03E-22	0.74288403	0.631	0.455	3.77E-18	8	Gpr137b

Gpbp1	2.69E-22	0.88840895	0.757	0.651	5.01E-18	8	Gpbp1
Nrde2	3.03E-22	0.57891446	0.275	0.095	5.62E-18	8	Nrde2
Oasl21	4.32E-22	0.4594138	0.239	0.075	8.02E-18	8	Oasl2
Atp6v0a1	4.46E-22	0.7239603	0.341	0.139	8.29E-18	8	Atp6v0a1
Mxi11	5.01E-22	0.70423768	0.541	0.314	9.31E-18	8	Mxi1
Phlpp11	5.73E-22	0.79193366	0.561	0.33	1.06E-17	8	Phlpp1
Cyba1	5.84E-22	0.55634663	0.894	0.991	1.08E-17	8	Cyba
Cpne2	8.76E-22	0.88137632	0.612	0.436	1.63E-17	8	Cpne2
Gramd1b	1.07E-21	0.64621864	0.247	0.081	1.99E-17	8	Gramd1b
Arl4c1	1.07E-21	0.89764397	0.714	0.561	1.99E-17	8	Arl4c
Polb	1.10E-21	0.96507434	0.647	0.509	2.04E-17	8	Polb
Sp1401	1.89E-21	1.07295004	0.78	0.748	3.51E-17	8	Sp140
Cxcl91	1.96E-21	1.00607611	0.302	0.108	3.65E-17	8	Cxcl9
Cytip1	2.35E-21	0.55873041	0.949	0.984	4.37E-17	8	Cytip
Nub1	4.78E-21	0.80171367	0.639	0.467	8.88E-17	8	Nub1
Atrx1	5.53E-21	1.12944198	0.808	0.757	1.03E-16	8	Atrx
Nfat51	5.86E-21	0.95546082	0.51	0.295	1.09E-16	8	Nfat5
Slc6a61	7.64E-21	0.7924428	0.808	0.725	1.42E-16	8	Slc6a6
Snhg151	1.05E-20	0.80790273	0.627	0.467	1.95E-16	8	Snhg15
Cd811	1.05E-20	1.1553033	0.69	0.538	1.96E-16	8	Cd81
Ctsz1	1.44E-20	0.74067013	0.855	0.97	2.68E-16	8	Ctsz
Pnpla8	1.54E-20	0.62571244	0.518	0.29	2.86E-16	8	Pnpla8
Nfe2l1	3.02E-20	0.79392752	0.584	0.408	5.61E-16	8	Nfe2l1

Dstn	3.19E-20	1.39305554	0.565	0.402	5.93E-16	8	Dstn
Bmt2	4.41E-20	0.65635397	0.412	0.199	8.19E-16	8	Bmt2
Cox17	5.32E-20	0.74284461	0.824	0.929	9.89E-16	8	Cox17
Pvr	6.40E-20	0.70767606	0.553	0.339	1.19E-15	8	Pvr
Osgin2	9.18E-20	0.39376352	0.329	0.134	1.71E-15	8	Osgin2
Rnaset2a	9.34E-20	0.87653912	0.424	0.23	1.73E-15	8	Rnaset2a
Map3k81	1.66E-19	0.82013916	0.557	0.341	3.09E-15	8	Map3k8
Aftph1	1.87E-19	0.80757748	0.659	0.485	3.48E-15	8	Aftph
Mgat4a	2.64E-19	0.34259756	0.243	0.082	4.90E-15	8	Mgat4a
Stat11	3.23E-19	0.93217424	0.757	0.667	6.00E-15	8	Stat1
Tor1aip21	4.47E-19	0.58464669	0.576	0.394	8.31E-15	8	Tor1aip2
Tbc1d1	7.02E-19	0.74233677	0.541	0.346	1.30E-14	8	Tbc1d1
N4bp1	7.99E-19	0.67929251	0.486	0.272	1.49E-14	8	N4bp1
Ccdc88a1	8.30E-19	1.1932636	0.741	0.661	1.54E-14	8	Ccdc88a
Pde4b2	9.08E-19	0.83000638	0.733	0.566	1.69E-14	8	Pde4b
Resf11	9.46E-19	0.94023156	0.647	0.479	1.76E-14	8	Resf1
Zfp809	1.77E-18	0.23359659	0.204	0.063	3.29E-14	8	Zfp809
Pkib1	2.08E-18	0.98688089	0.78	0.827	3.87E-14	8	Pkib
Rnf19b2	2.14E-18	0.53593101	0.733	0.535	3.98E-14	8	Rnf19b
Ccdc50	2.33E-18	0.8226933	0.706	0.584	4.34E-14	8	Ccdc50
Triobp	2.71E-18	0.50856694	0.424	0.219	5.03E-14	8	Triobp
Peak1	3.00E-18	0.56041124	0.271	0.105	5.58E-14	8	Peak1
Kdm2b1	3.10E-18	1.02312277	0.576	0.413	5.77E-14	8	Kdm2b

Mrpl14	3.43E-18	0.66937743	0.655	0.518	6.38E-14	8	Mrpl14
Cep3501	3.82E-18	0.87318296	0.608	0.434	7.10E-14	8	Cep350
Grb2	4.65E-18	0.57757029	0.835	0.928	8.63E-14	8	Grb2
Spop1	5.29E-18	0.68170618	0.824	0.783	9.84E-14	8	Spop
Gabarap	9.19E-18	0.52517109	0.839	0.966	1.71E-13	8	Gabarap
Chka1	9.38E-18	1.24178207	0.522	0.327	1.74E-13	8	Chka
Zmiz2	1.12E-17	0.6984513	0.635	0.48	2.09E-13	8	Zmiz2
Kif2a	1.24E-17	0.58776992	0.471	0.287	2.31E-13	8	Kif2a
Dusp21	1.32E-17	1.01705113	0.518	0.318	2.45E-13	8	Dusp2
Ino80d	1.91E-17	0.65502574	0.478	0.28	3.55E-13	8	Ino80d
Zfand3	2.05E-17	0.5505117	0.576	0.394	3.81E-13	8	Zfand3
Filip1l	2.27E-17	0.86915235	0.694	0.571	4.22E-13	8	Filip1l
Rapgef21	2.60E-17	0.5250232	0.38	0.187	4.84E-13	8	Rapgef2
Gnptab	2.97E-17	0.66431539	0.325	0.153	5.52E-13	8	Gnptab
Foxn3	3.11E-17	0.77806293	0.506	0.309	5.78E-13	8	Foxn3
Rab22a	5.40E-17	0.49662327	0.482	0.28	1.00E-12	8	Rab22a
Crtc2	7.95E-17	0.48042375	0.459	0.264	1.48E-12	8	Crtc2
Txn11	8.12E-17	0.97143404	0.843	0.941	1.51E-12	8	Txn1
Ccdc6	8.86E-17	0.51281887	0.298	0.129	1.65E-12	8	Ccdc6
Arhgap22	1.15E-16	0.88201212	0.561	0.413	2.14E-12	8	Arhgap22
Tpm41	1.18E-16	0.62953415	0.835	0.911	2.19E-12	8	Tpm4
Rab9	1.33E-16	0.4351283	0.388	0.202	2.47E-12	8	Rab9
Traf6	1.37E-16	0.40077856	0.31	0.135	2.55E-12	8	Traf6

Kdm4a	1.51E-16	1.06207502	0.478	0.316	2.81E-12	8	Kdm4a
Rap1b	1.64E-16	0.48706816	0.859	0.934	3.05E-12	8	Rap1b
Arrb2	1.94E-16	0.67321465	0.533	0.354	3.60E-12	8	Arrb2
Rab13	2.15E-16	0.30879678	0.255	0.099	4.00E-12	8	Rab13
Eif11	2.57E-16	0.33818529	0.941	0.995	4.77E-12	8	Eif1
Gramd31	4.22E-16	0.67993826	0.561	0.402	7.83E-12	8	Gramd3
Nuak21	4.75E-16	0.88459534	0.741	0.607	8.82E-12	8	Nuak2
Micu1	5.25E-16	0.48029691	0.286	0.124	9.75E-12	8	Micu1
Peli11	6.49E-16	0.75375307	0.729	0.609	1.21E-11	8	Peli1
Ppp4r2	7.78E-16	0.78523723	0.588	0.474	1.45E-11	8	Ppp4r2
Slc3a21	8.17E-16	0.61517243	0.89	0.897	1.52E-11	8	Slc3a2
Rhog	1.13E-15	0.7056411	0.722	0.645	2.11E-11	8	Rhog
Il151	1.33E-15	0.31868122	0.224	0.084	2.47E-11	8	Il15
Thap2	1.92E-15	0.49306058	0.255	0.103	3.56E-11	8	Thap2
Pnrc11	2.01E-15	0.70411529	0.831	0.885	3.73E-11	8	Pnrc1
Rad211	2.07E-15	0.5894633	0.478	0.3	3.85E-11	8	Rad21
Traf2	2.21E-15	0.39647354	0.298	0.134	4.10E-11	8	Traf2
Mvp	3.45E-15	0.617554	0.627	0.485	6.41E-11	8	Mvp
Ass11	3.48E-15	1.0528251	0.635	0.557	6.47E-11	8	Ass1
Zbtb18	4.04E-15	0.42961414	0.243	0.098	7.51E-11	8	Zbtb18
Cyfip1	4.16E-15	0.65978977	0.584	0.438	7.72E-11	8	Cyfip1
Inpp5b	6.21E-15	0.57323687	0.267	0.122	1.15E-10	8	Inpp5b
Tbc1d81	6.61E-15	0.80902178	0.737	0.666	1.23E-10	8	Tbc1d8

Appl1	8.52E-15	0.74737903	0.573	0.401	1.58E-10	8	Appl1
Anxa52	9.06E-15	0.64548674	0.816	0.891	1.68E-10	8	Anxa5
Atxn7l11	9.39E-15	0.79265596	0.506	0.337	1.74E-10	8	Atxn7l1
Mthfs	1.01E-14	0.43536086	0.384	0.2	1.88E-10	8	Mthfs
Arhgef21	1.24E-14	0.51628271	0.471	0.289	2.30E-10	8	Arhgef2
Cxcl10	1.24E-14	0.28986602	0.216	0.079	2.30E-10	8	Cxcl10
Npc22	1.33E-14	0.77569793	0.894	0.986	2.48E-10	8	Npc2
Cish	1.43E-14	0.41402746	0.216	0.08	2.66E-10	8	Cish
Plgrkt	1.44E-14	0.56497044	0.518	0.354	2.68E-10	8	Plgrkt
Ddhd11	2.35E-14	0.80283002	0.467	0.304	4.37E-10	8	Ddhd1
Gtf2a1	2.52E-14	0.46800842	0.443	0.258	4.69E-10	8	Gtf2a1
Tapbpl	2.76E-14	0.66932811	0.494	0.329	5.12E-10	8	Tapbpl
Sbno11	3.13E-14	0.70544409	0.612	0.48	5.81E-10	8	Sbno1
Il4ra1	3.97E-14	0.73994094	0.525	0.342	7.38E-10	8	Il4ra
Pik3r51	4.14E-14	0.69479025	0.49	0.324	7.69E-10	8	Pik3r5
Alkbh6	4.53E-14	0.31890066	0.349	0.18	8.42E-10	8	Alkbh6
Stat31	5.41E-14	0.97098184	0.765	0.729	1.00E-09	8	Stat3
Dennd5a1	5.57E-14	0.69027746	0.545	0.391	1.03E-09	8	Dennd5a
C130026I21Rik	6.28E-14	0.42310838	0.318	0.156	1.17E-09	8	C130026I21Rik
Jup1	6.33E-14	0.58339434	0.439	0.266	1.18E-09	8	Jup
Tmcc1	6.47E-14	0.49949002	0.38	0.202	1.20E-09	8	Tmcc1
Hax1	7.04E-14	0.4738075	0.471	0.3	1.31E-09	8	Hax1
Sav1	7.14E-14	0.45504729	0.341	0.176	1.33E-09	8	Sav1

Renbp	7.58E-14	0.22996473	0.227	0.091	1.41E-09	8	Renbp
Ppp1r15a2	8.91E-14	0.53037564	0.843	0.817	1.66E-09	8	Ppp1r15a
Mthfd21	9.15E-14	0.68326372	0.557	0.409	1.70E-09	8	Mthfd2
Myh91	9.47E-14	0.77653475	0.831	0.834	1.76E-09	8	Myh9
Ilrun1	9.79E-14	0.5376761	0.541	0.384	1.82E-09	8	Ilrun
Calm11	1.04E-13	1.07346849	0.957	0.996	1.93E-09	8	Calm1
Prr14	1.23E-13	0.50416876	0.353	0.19	2.29E-09	8	Prr14
Gga3	1.32E-13	0.44716096	0.275	0.125	2.45E-09	8	Gga3
Slc15a31	1.60E-13	0.76871577	0.482	0.306	2.98E-09	8	Slc15a3
Tapbp2	1.93E-13	0.60904729	0.859	0.886	3.59E-09	8	Tapbp
Sfmbt1	2.13E-13	0.36518243	0.235	0.1	3.96E-09	8	Sfmbt1
Sepsecs	2.19E-13	0.52631666	0.255	0.116	4.06E-09	8	Sepsecs
Bcl2l1	2.34E-13	0.4584487	0.333	0.173	4.35E-09	8	Bcl2l1
Nostrin	3.42E-13	0.95695516	0.427	0.288	6.35E-09	8	Nostrin
Abcg11	3.66E-13	0.65033564	0.51	0.344	6.81E-09	8	Abcg1
Sh3glb11	5.00E-13	0.484446	0.812	0.871	9.29E-09	8	Sh3glb1
Hmgn31	5.24E-13	0.84933471	0.537	0.401	9.73E-09	8	Hmgn3
Nxf11	6.03E-13	0.76915925	0.475	0.306	1.12E-08	8	Nxf1
Myo1c	6.78E-13	0.61190561	0.459	0.294	1.26E-08	8	Myo1c
Ifi303	6.92E-13	0.69126365	0.871	0.955	1.29E-08	8	Ifi30
Got12	1.10E-12	0.69690471	0.722	0.694	2.04E-08	8	Got1
Prr71	1.35E-12	0.41880578	0.376	0.212	2.52E-08	8	Prr7
Psme11	1.46E-12	0.3927088	0.918	0.987	2.71E-08	8	Psme1

Zeb11	1.87E-12	0.77789801	0.537	0.405	3.47E-08	8	Zeb1
Cd861	1.95E-12	0.92202711	0.749	0.815	3.62E-08	8	Cd86
Gtpbp1	2.18E-12	0.37697879	0.349	0.189	4.06E-08	8	Gtpbp1
St8sia41	2.52E-12	0.98822495	0.722	0.663	4.67E-08	8	St8sia4
Med11	2.55E-12	0.42634254	0.408	0.244	4.74E-08	8	Med11
Wasl	2.83E-12	0.42393968	0.298	0.149	5.25E-08	8	Wasl
Nectin11	3.02E-12	0.46260744	0.443	0.276	5.61E-08	8	Nectin1
Kdm6a1	3.48E-12	0.67996463	0.529	0.369	6.46E-08	8	Kdm6a
Casp41	3.52E-12	0.39384917	0.325	0.173	6.54E-08	8	Casp4
Herc61	6.10E-12	0.47123471	0.353	0.193	1.13E-07	8	Herc6
Gnai3	6.84E-12	0.59400254	0.627	0.522	1.27E-07	8	Gnai3
Odc11	7.29E-12	1.02715956	0.722	0.713	1.35E-07	8	Odc1
Ikbkb1	7.60E-12	0.76407769	0.725	0.681	1.41E-07	8	Ikbkb
Fem1b	8.23E-12	0.33909934	0.22	0.095	1.53E-07	8	Fem1b
Mob3a	9.48E-12	0.66751204	0.608	0.507	1.76E-07	8	Mob3a
Dynll2	9.90E-12	0.67173819	0.635	0.569	1.84E-07	8	Dynll2
Zfp2171	1.05E-11	0.49128954	0.361	0.199	1.95E-07	8	Zfp217
Gnas	1.11E-11	0.49134664	0.851	0.958	2.06E-07	8	Gnas
Epsti11	1.13E-11	1.66838137	0.678	0.749	2.09E-07	8	Epsti1
Rbbp8	1.24E-11	0.71620914	0.675	0.56	2.30E-07	8	Rbbp8
Rab12	1.30E-11	0.32010032	0.255	0.121	2.41E-07	8	Rab12
Ext11	1.46E-11	0.68202847	0.502	0.349	2.72E-07	8	Ext1
Rnf157	1.58E-11	0.77512292	0.376	0.227	2.94E-07	8	Rnf157

Josd1	1.87E-11	0.50351552	0.294	0.152	3.47E-07	8	Josd1
Ncoa4	1.93E-11	0.48554453	0.369	0.219	3.58E-07	8	Ncoa4
Stx6	2.05E-11	0.39678315	0.349	0.191	3.82E-07	8	Stx6
Apaf11	2.14E-11	0.71444011	0.51	0.368	3.97E-07	8	Apaf1
Clasp21	2.65E-11	0.53281063	0.424	0.262	4.91E-07	8	Clasp2
Sik31	2.97E-11	0.62881519	0.416	0.267	5.52E-07	8	Sik3
Kansl3	3.46E-11	0.39947148	0.31	0.166	6.44E-07	8	Kansl3
Foxn21	4.45E-11	0.57784287	0.525	0.379	8.27E-07	8	Foxn2
Dnajb2	6.14E-11	0.57510842	0.38	0.241	1.14E-06	8	Dnajb2
Rest	6.21E-11	0.57232218	0.447	0.297	1.15E-06	8	Rest
Fyn1	6.41E-11	0.66026633	0.702	0.611	1.19E-06	8	Fyn
Pde6d	6.59E-11	0.28870393	0.286	0.146	1.22E-06	8	Pde6d
Etnk11	7.69E-11	0.54741957	0.537	0.381	1.43E-06	8	Etnk1
Grasp2	8.39E-11	0.55724856	0.765	0.696	1.56E-06	8	Grasp
Csnk1g1	9.66E-11	0.57514369	0.361	0.215	1.80E-06	8	Csnk1g1
Tuba1b1	1.21E-10	0.6472693	0.714	0.692	2.24E-06	8	Tuba1b
Marf1	1.55E-10	0.5221658	0.333	0.198	2.88E-06	8	Marf1
Clptm1	1.69E-10	0.39275093	0.404	0.258	3.15E-06	8	Clptm1
Cnn21	1.71E-10	0.68082896	0.784	0.876	3.18E-06	8	Cnn2
Tubb51	2.19E-10	0.70187636	0.765	0.783	4.07E-06	8	Tubb5
Chst11	2.32E-10	0.23477305	0.224	0.103	4.32E-06	8	Chst11
Stat6	2.50E-10	0.54726002	0.522	0.377	4.65E-06	8	Stat6
Ube2z1	2.98E-10	0.39120122	0.416	0.267	5.54E-06	8	Ube2z

Pgap21	3.85E-10	0.41433264	0.471	0.323	7.16E-06	8	Pgap2
Cbfa2t33	4.47E-10	0.76591523	0.831	0.905	8.31E-06	8	Cbfa2t3
Dph5	4.65E-10	0.46540767	0.282	0.152	8.64E-06	8	Dph5
Palld1	5.39E-10	0.44170859	0.286	0.156	1.00E-05	8	Palld
Prkcd1	5.73E-10	0.63372715	0.663	0.592	1.06E-05	8	Prkcd
Prex11	5.91E-10	0.71170794	0.612	0.503	1.10E-05	8	Prex1
Lcp11	6.03E-10	0.53047269	0.867	0.974	1.12E-05	8	Lcp1
Krit1	6.11E-10	0.62905299	0.463	0.317	1.14E-05	8	Krit1
Prpf38b1	6.75E-10	0.73817812	0.741	0.707	1.25E-05	8	Prpf38b
Stat5b	6.85E-10	0.31711956	0.275	0.146	1.27E-05	8	Stat5b
Polr3c	7.45E-10	0.29447209	0.341	0.202	1.38E-05	8	Polr3c
Kbtbd3	7.61E-10	0.22513263	0.239	0.117	1.41E-05	8	Kbtbd3
Afap1	7.61E-10	0.48136185	0.267	0.144	1.41E-05	8	Afap1
Ldlr1	7.73E-10	0.36619097	0.322	0.179	1.44E-05	8	Ldlr
Crybg11	8.12E-10	0.55709541	0.6	0.473	1.51E-05	8	Crybg1
Hat1	9.88E-10	0.36891633	0.463	0.326	1.84E-05	8	Hat1
Thap121	1.05E-09	0.60211797	0.447	0.305	1.95E-05	8	Thap12
Isg151	1.14E-09	1.18147579	0.537	0.417	2.13E-05	8	Isg15
Osbpl8	1.65E-09	0.6146404	0.38	0.244	3.06E-05	8	Osbpl8
Cxcl2	1.71E-09	0.92286754	0.357	0.222	3.19E-05	8	Cxcl2
Nrip11	2.10E-09	0.78703887	0.62	0.535	3.91E-05	8	Nrip1
Slc38a21	2.22E-09	0.82669089	0.835	0.858	4.12E-05	8	Slc38a2
B3gnt5	2.86E-09	0.4767816	0.439	0.297	5.32E-05	8	B3gnt5

Nsmce3	3.20E-09	0.70290552	0.341	0.217	5.95E-05	8	Nsmce3
Trim7	3.61E-09	0.50838563	0.282	0.158	6.70E-05	8	Trim7
Rsad21	4.02E-09	0.7248421	0.259	0.136	7.48E-05	8	Rsad2
Ndrg11	4.80E-09	0.78327326	0.478	0.365	8.93E-05	8	Ndrg1
Hmg20b	4.89E-09	0.55873149	0.573	0.486	9.08E-05	8	Hmg20b
Gtpbp2	4.99E-09	0.50992055	0.349	0.216	9.27E-05	8	Gtpbp2
Edem11	5.11E-09	0.49880482	0.561	0.431	9.49E-05	8	Edem1
Rras	6.25E-09	0.26759409	0.286	0.161	0.00011608	8	Rras
Ube2e3	7.57E-09	0.50246469	0.58	0.492	0.00014061	8	Ube2e3
Myl6	7.99E-09	0.38162353	0.882	0.986	0.00014849	8	Myl6
Pphln1	8.40E-09	0.30424641	0.373	0.239	0.00015606	8	Pphln1
Ube2l61	1.04E-08	0.39803429	0.4	0.274	0.00019404	8	Ube2l6
Nbn	1.12E-08	0.25011101	0.22	0.11	0.00020776	8	Nbn
Tnf1	1.19E-08	0.25431372	0.22	0.107	0.00022112	8	Tnf
Ptprj1	1.23E-08	0.57425547	0.541	0.421	0.00022856	8	Ptprj
Amn1	1.26E-08	0.44992808	0.243	0.129	0.00023411	8	Amn1
Atp2b11	1.29E-08	0.85760584	0.816	0.843	0.00023926	8	Atp2b1
Zfand6	1.30E-08	0.81135661	0.612	0.586	0.00024237	8	Zfand6
Tmsb4x1	1.43E-08	0.24486836	1	1	0.00026524	8	Tmsb4x
Myl12a	1.48E-08	0.49818444	0.784	0.908	0.00027525	8	Myl12a
Ints3	1.53E-08	0.29183334	0.263	0.145	0.00028502	8	Ints3
Oga1	1.55E-08	0.56664122	0.506	0.375	0.00028856	8	Oga
Ncoa21	1.71E-08	0.62803244	0.388	0.261	0.00031687	8	Ncoa2

S100a42	2.02E-08	0.80926908	0.557	0.442	0.00037602	8	S100a4
Rtn41	2.18E-08	0.4295741	0.831	0.896	0.00040435	8	Rtn4
Helz21	2.48E-08	0.76755969	0.282	0.172	0.00046047	8	Helz2
Cd2bp2	3.06E-08	0.29735826	0.38	0.251	0.00056941	8	Cd2bp2
Mtmr41	3.62E-08	0.35783175	0.455	0.328	0.00067168	8	Mtmr4
Mapre21	3.69E-08	0.46047826	0.51	0.393	0.00068588	8	Mapre2
Ogfrl11	3.72E-08	0.86341701	0.518	0.464	0.00069183	8	Ogfrl1
Asl	3.91E-08	0.60308981	0.408	0.3	0.00072654	8	Asl
M6pr2	5.28E-08	0.44656999	0.792	0.86	0.00098125	8	M6pr
Ift57	5.60E-08	0.3702129	0.251	0.141	0.00103977	8	Ift57
Bcl9	6.07E-08	0.30729746	0.322	0.196	0.00112806	8	Bcl9
Tbl1x1	6.13E-08	0.53184922	0.494	0.386	0.00113935	8	Tbl1x
Ciao2b	6.56E-08	0.44555724	0.631	0.604	0.0012188	8	Ciao2b
Rufy3	7.21E-08	0.2409003	0.204	0.102	0.00133962	8	Rufy3
Srrm21	9.76E-08	0.59312337	0.929	0.952	0.00181372	8	Srrm2
Tmem1271	1.01E-07	0.3082537	0.31	0.187	0.00187552	8	Tmem127
Uba71	1.11E-07	0.46372939	0.314	0.199	0.0020603	8	Uba7
Ifi2091	1.59E-07	0.54221747	0.639	0.622	0.00295078	8	Ifi209
Enox2	1.61E-07	0.28496324	0.204	0.106	0.00300019	8	Enox2
Edf1	1.73E-07	0.46098075	0.757	0.889	0.00321152	8	Edf1
Rab11fip12	1.78E-07	0.56817829	0.718	0.685	0.00329909	8	Rab11fip1
Tnfrsf1b1	1.85E-07	0.44950735	0.396	0.286	0.00343092	8	Tnfrsf1b
Tmod3	1.92E-07	0.40805293	0.608	0.537	0.00356313	8	Tmod3

Nfkbiz	1.98E-07	0.34168356	0.349	0.224	0.00367772	8	Nfkbiz
A530032D15Rik1	2.08E-07	0.57291072	0.325	0.206	0.0038589	8	A530032D15Rik
Sgpl11	2.13E-07	0.50723715	0.514	0.436	0.00394833	8	Sgpl1
Siglecg	2.93E-07	0.28548575	0.18	0.346	0.00543551	8	Siglecg
Zup11	3.26E-07	0.53377803	0.537	0.432	0.00605549	8	Zup1
Gabpb1	3.46E-07	0.20663112	0.243	0.132	0.00642802	8	Gabpb1
Fam110a	3.57E-07	0.39015377	0.255	0.147	0.00664176	8	Fam110a
Pmvk	3.67E-07	0.73429898	0.612	0.617	0.00682308	8	Pmvk
Trim24	3.70E-07	0.50128438	0.286	0.184	0.00687242	8	Trim24
Cdc42bpb	3.76E-07	0.43394245	0.286	0.176	0.00699507	8	Cdc42bpb
Specc1l	3.93E-07	0.49941463	0.357	0.244	0.00730587	8	Specc1l
Nup981	4.23E-07	0.61729117	0.647	0.6	0.0078629	8	Nup98
Elk3	4.24E-07	0.3140044	0.2	0.103	0.00787905	8	Elk3
Nck2	4.26E-07	0.21113142	0.267	0.157	0.00790789	8	Nck2
Creb1	4.36E-07	0.43750303	0.439	0.326	0.0080993	8	Creb1
Timd41	5.08E-07	0.69595669	0.255	0.154	0.00943064	8	Timd4
Ahctf11	5.13E-07	0.29007337	0.4	0.268	0.0095371	8	Ahctf1
Bcl10	6.03E-07	0.45269044	0.667	0.649	0.01119979	8	Bcl10
Auh	6.05E-07	0.51820532	0.388	0.292	0.01124718	8	Auh
Hsd17b111	6.23E-07	0.61030415	0.525	0.448	0.01157914	8	Hsd17b11
Dhrs3	8.50E-07	0.2103948	0.22	0.121	0.01580183	8	Dhrs3
Tuba1c	8.56E-07	0.52891312	0.588	0.541	0.01590763	8	Tuba1c
Cpeb41	8.69E-07	0.29036682	0.322	0.208	0.0161406	8	Cpeb4

Mpc12	8.98E-07	0.56083419	0.729	0.783	0.0166797	8	Mpc1
Mthfsl	1.08E-06	0.33593389	0.357	0.252	0.02004849	8	Mthfsl
Gm43305	1.28E-06	0.26801466	0.227	0.128	0.02381024	8	Gm43305
Mtmr14	1.33E-06	0.32258101	0.475	0.373	0.02470942	8	Mtmr14
Khnyn	1.41E-06	0.28881197	0.216	0.123	0.0262413	8	Khnyn
Spr	1.55E-06	0.34032493	0.341	0.238	0.02874078	8	Spr
Apobec31	1.69E-06	0.46234916	0.722	0.738	0.03136369	8	Apobec3
Clasp11	1.74E-06	0.42711271	0.376	0.266	0.03231566	8	Clasp1
Znfx12	1.74E-06	0.41254513	0.412	0.3	0.03237588	8	Znfx1
Paf1	1.92E-06	0.37605124	0.408	0.294	0.03566268	8	Paf1
Bcl2l11	1.97E-06	0.68451568	0.533	0.448	0.03662806	8	Bcl2l11
Sppl3	2.02E-06	0.29886564	0.282	0.177	0.03761339	8	Sppl3
Aplp21	2.43E-06	0.58726458	0.455	0.366	0.04517361	8	Aplp2
Synj11	2.48E-06	0.45509466	0.737	0.715	0.04601877	8	Synj1
Atp6v1b21	2.59E-06	0.35293275	0.467	0.374	0.04817364	8	Atp6v1b2
Kansl1	2.62E-06	0.58517067	0.498	0.417	0.04866892	8	Kansl1
Lsp1	2.69E-06	0.29547162	0.929	0.992	0.05003899	8	Lsp1
Parp141	2.95E-06	0.4427013	0.424	0.325	0.05485204	8	Parp14
D930030I03Rik	3.04E-06	0.22742377	0.212	0.119	0.05645879	8	D930030I03Rik
Slc25a19	3.28E-06	0.24616513	0.275	0.172	0.06089687	8	Slc25a19
Daam1	3.56E-06	0.2196389	0.216	0.122	0.06621836	8	Daam1
Piezo11	4.10E-06	0.4277114	0.376	0.279	0.0760906	8	Piezo1
Cyth3	4.68E-06	0.29901552	0.227	0.133	0.08689966	8	Cyth3

Cmip	4.94E-06	0.46262878	0.42	0.329	0.09177768	8	Cmip
Vcp2	5.14E-06	0.47691326	0.784	0.832	0.09549202	8	Vcp
Bcor	5.54E-06	0.20635849	0.204	0.114	0.10299154	8	Bcor
Map2k1	6.89E-06	0.43359161	0.467	0.368	0.12808786	8	Map2k1
Fam13b	7.57E-06	0.37624621	0.333	0.229	0.14056599	8	Fam13b
Rbm4b	8.51E-06	0.29510696	0.294	0.195	0.15804936	8	Rbm4b
Socs11	8.61E-06	0.43400634	0.259	0.163	0.15992793	8	Socs1
Gm269171	9.18E-06	1.62959604	0.424	0.33	0.17064307	8	Gm26917
Ankib11	9.43E-06	0.34760824	0.365	0.258	0.17516101	8	Ankib1
Cdc42ep3	9.80E-06	0.46182579	0.325	0.232	0.18216095	8	Cdc42ep3
Gng52	1.05E-05	0.32619225	0.812	0.943	0.19441578	8	Gng5
Prrc2b1	1.12E-05	0.33024352	0.416	0.322	0.20874992	8	Prrc2b
Vwa5a1	1.13E-05	0.70758628	0.388	0.312	0.21085925	8	Vwa5a
Ranbp21	1.20E-05	0.20024158	0.58	0.766	0.22379278	8	Ranbp2
Sbds	1.29E-05	0.38891004	0.596	0.533	0.23957712	8	Sbds
Rela1	1.31E-05	0.2685908	0.471	0.384	0.2435915	8	Rela
Tab21	1.36E-05	0.41456179	0.62	0.58	0.25350855	8	Tab2
Itprid21	1.54E-05	0.43237927	0.486	0.393	0.28568786	8	Itprid2
Lpin2	1.64E-05	0.37270389	0.267	0.177	0.30402984	8	Lpin2
Tmbim1	1.66E-05	0.2635036	0.255	0.16	0.30810894	8	Tmbim1
Sumo21	1.71E-05	0.3838271	0.78	0.91	0.31816638	8	Sumo2
Stk401	1.97E-05	0.29941555	0.384	0.298	0.3661304	8	Stk40
Fnbp11	2.19E-05	0.35714047	0.906	0.958	0.40693002	8	Fnbp1

Emd	2.30E-05	0.36215775	0.686	0.708	0.42776301	8	Emd
Slc41a11	2.49E-05	0.46714612	0.29	0.201	0.46237294	8	Slc41a1
Washc2	2.50E-05	0.29858321	0.376	0.276	0.46433681	8	Washc2
Fbrs	2.79E-05	0.41578083	0.459	0.369	0.51749001	8	Fbrs
Fam32a	2.84E-05	0.44504323	0.569	0.543	0.52859902	8	Fam32a
Taok11	2.85E-05	0.51822178	0.482	0.382	0.52969952	8	Taok1
Chd71	2.92E-05	0.64208082	0.604	0.578	0.54281057	8	Chd7
Slc25a331	3.04E-05	0.21566816	0.247	0.154	0.56542119	8	Slc25a33
Dapp1	3.32E-05	0.47067076	0.82	0.846	0.61596635	8	Dapp1
Chp1	4.19E-05	0.38577661	0.463	0.396	0.77937062	8	Chp1
Ccdc82	4.58E-05	0.22443365	0.247	0.161	0.8503504	8	Ccdc82
Dennd4b1	4.68E-05	0.37847303	0.427	0.341	0.86865857	8	Dennd4b
Dennd4a1	4.87E-05	0.60389943	0.847	0.907	0.90550699	8	Dennd4a
Frmd4a2	5.09E-05	0.58044061	0.443	0.369	0.94571919	8	Frmd4a
Cpsf71	5.47E-05	0.4827541	0.345	0.261	1	8	Cpsf7
Kmt2a1	5.75E-05	0.37946813	0.498	0.429	1	8	Kmt2a
Sel1l1	5.81E-05	0.41381306	0.361	0.27	1	8	Sel1l
Ash1l1	5.90E-05	0.48884935	0.502	0.429	1	8	Ash1l
Spag91	6.06E-05	0.40231375	0.949	0.963	1	8	Spag9
Zmym2	6.56E-05	0.26050565	0.278	0.187	1	8	Zmym2
mt-Co11	8.09E-05	0.63866806	0.992	0.998	1	8	mt-Co1
Ebi3	8.95E-05	0.34424377	0.278	0.198	1	8	Ebi3
Riok3	9.38E-05	0.35636526	0.667	0.672	1	8	Riok3

Kif1b	9.55E-05	0.22369159	0.22	0.141	1	8	Kif1b
Rasa41	9.95E-05	0.32954329	0.408	0.317	1	8	Rasa4
Sugt1	0.00011204	0.32489153	0.651	0.685	1	8	Sugt1
Dync1i2	0.00011698	0.335835	0.686	0.718	1	8	Dync1i2
Gpd21	0.00012143	0.42209253	0.655	0.642	1	8	Gpd2
Plekhb21	0.00012714	0.33661239	0.431	0.359	1	8	Plekhb2
Lrrc8c1	0.00012948	0.55030271	0.549	0.5	1	8	Lrrc8c
Psip1	0.00013632	0.37139082	0.349	0.27	1	8	Psip1
Arrdc1	0.00013763	0.29537352	0.427	0.344	1	8	Arrdc1
Zfp598	0.00014058	0.33864224	0.208	0.133	1	8	Zfp598
Cttnbp2nl	0.00014222	0.41844819	0.392	0.317	1	8	Cttnbp2nl
Phip1	0.00014365	0.84277235	0.482	0.416	1	8	Phip
Necap2	0.0001506	0.42130155	0.631	0.653	1	8	Necap2
Sertad2	0.000167	0.35804017	0.361	0.27	1	8	Sertad2
Haus8	0.00017056	0.23801717	0.314	0.224	1	8	Haus8
Arpp19	0.0001725	0.23012238	0.788	0.909	1	8	Arpp19
Rgs31	0.00020432	0.31706505	0.353	0.269	1	8	Rgs3
Gosr2	0.00021463	0.32812649	0.545	0.505	1	8	Gosr2
Grina1	0.00022406	0.35822362	0.357	0.275	1	8	Grina
Ube2h	0.00024364	0.38241611	0.424	0.36	1	8	Ube2h
Gm17056	0.00026325	0.4359139	0.204	0.129	1	8	Gm17056
Mbtd11	0.00027012	0.47503685	0.498	0.436	1	8	Mbtd1
Tmbim4	0.0002825	0.88424841	0.533	0.526	1	8	Tmbim4

Vkorc1l1	0.0002901	0.20153713	0.243	0.167	1	8	Vkorc1l1
Ccni	0.00029584	0.2956488	0.451	0.388	1	8	Ccni
Dgat11	0.00029621	0.60218219	0.243	0.167	1	8	Dgat1
Plaat31	0.00032051	0.62684708	0.569	0.564	1	8	Plaat3
Stx12	0.00034023	0.3173426	0.42	0.34	1	8	Stx12
Il10ra2	0.00035108	0.29785767	0.396	0.306	1	8	Il10ra
Gramd1a	0.0003703	0.33166632	0.22	0.144	1	8	Gramd1a
Tap12	0.00037569	0.32770129	0.827	0.897	1	8	Tap1
Rspry1	0.00037948	0.23381679	0.31	0.231	1	8	Rspry1
Flnb	0.00038466	0.38848664	0.267	0.193	1	8	Flnb
Srgn	0.00040325	0.25623643	0.937	0.995	1	8	Srgn
Ankrd441	0.00041481	0.74776342	0.643	0.643	1	8	Ankrd44
Sdf4	0.00041585	0.36202561	0.612	0.622	1	8	Sdf4
Pkn2	0.00043091	0.44486389	0.522	0.474	1	8	Pkn2
Kmt2b	0.00043949	0.25185765	0.298	0.22	1	8	Kmt2b
Clip1	0.00044812	0.34094457	0.263	0.189	1	8	Clip1
Tmbim61	0.00047766	0.24377922	0.816	0.939	1	8	Tmbim6
mt-Atp6	0.00049575	0.21522406	0.976	0.997	1	8	mt-Atp6
Skil1	0.00050798	0.37364627	0.871	0.887	1	8	Skil
Pan31	0.00057946	0.55383133	0.494	0.438	1	8	Pan3
Eea1	0.00058715	0.42532596	0.396	0.321	1	8	Eea1
Rell11	0.00060179	0.46329121	0.451	0.4	1	8	Rell1
Atxn7	0.00061991	0.53856715	0.306	0.237	1	8	Atxn7

Lgals32	0.00062855	0.32546273	0.792	0.986	1	8	Lgals3
Slc29a3	0.00069218	0.30068486	0.31	0.236	1	8	Slc29a3
Pirb1	0.00071392	0.47675417	0.647	0.66	1	8	Pirb
Map4k5	0.00074099	0.35958484	0.282	0.212	1	8	Map4k5
Ube2i	0.00076978	0.30817739	0.729	0.839	1	8	Ube2i
Dusp111	0.00084015	0.41740148	0.698	0.747	1	8	Dusp11
Pex13	0.00084913	0.2051201	0.282	0.208	1	8	Pex13
Akt3	0.00085579	0.20506879	0.243	0.173	1	8	Akt3
Cstb	0.00091405	0.54484598	0.588	0.64	1	8	Cstb
Cstf3	0.00091983	0.34726497	0.29	0.216	1	8	Cstf3
Hif1a	0.00093718	0.32773897	0.541	0.522	1	8	Hif1a
Casc3	0.00096778	0.27009501	0.388	0.311	1	8	Casc3
Tasor1	0.0009795	0.43895813	0.322	0.25	1	8	Tasor
Tfe3	0.00108378	0.31298177	0.31	0.24	1	8	Tfe3
Degs11	0.00117245	0.27434489	0.447	0.381	1	8	Degs1
Asap2	0.00122184	0.30636719	0.322	0.261	1	8	Asap2
Rac1	0.00128807	0.2858494	0.788	0.908	1	8	Rac1
Rnaset2b	0.00136691	0.24046241	0.267	0.199	1	8	Rnaset2b
Aff41	0.00138826	0.39860125	0.541	0.506	1	8	Aff4
Tmem81	0.00148762	0.32875599	0.325	0.262	1	8	Tmem8
Gm424181	0.00151052	0.70722069	1	1	1	8	Gm42418
Nae1	0.00156705	0.34293748	0.306	0.242	1	8	Nae1
Ahcyl2	0.00165777	0.38380143	0.49	0.455	1	8	Ahcyl2

Nfil31	0.00169891	0.37918261	0.545	0.499	1	8	Nfil3
Kdm5c1	0.00176432	0.34712449	0.337	0.272	1	8	Kdm5c
Mar-71	0.0019018	0.46363353	0.627	0.587	1	8	Mar-07
Polr2l	0.00208509	0.49065676	0.412	0.558	1	8	Polr2l
Fbxo111	0.00217634	0.35423458	0.51	0.475	1	8	Fbxo11
Gsdmd	0.00243164	0.27727103	0.451	0.404	1	8	Gsdmd
Ak2	0.00244913	0.28088549	0.427	0.38	1	8	Ak2
Gdi11	0.00245965	0.23946674	0.349	0.284	1	8	Gdi1
Ptpn2	0.00246004	0.21572734	0.533	0.498	1	8	Ptpn2
Psmd7	0.00246131	0.27444487	0.643	0.654	1	8	Psmd7
Zc3h12a	0.00266588	0.24798551	0.263	0.199	1	8	Zc3h12a
Pde3b	0.00267434	0.22047879	0.212	0.15	1	8	Pde3b
Pank2	0.00279613	0.23185221	0.341	0.279	1	8	Pank2
Plxnd11	0.00290111	0.31287184	0.251	0.188	1	8	Plxnd1
Smc51	0.00293127	0.4040644	0.282	0.218	1	8	Smc5
Mrps7	0.00297882	0.27044162	0.506	0.484	1	8	Mrps7
Atp6v1f	0.0030209	0.21377351	0.773	0.905	1	8	Atp6v1f
Nup881	0.00306269	0.31569014	0.408	0.344	1	8	Nup88
Npepps1	0.00313967	0.24278767	0.322	0.248	1	8	Npepps
Gpx42	0.00317397	0.54709035	0.663	0.748	1	8	Gpx4
Ttc39a	0.00326858	0.24216038	0.204	0.145	1	8	Ttc39a
Sdhaf2	0.00360633	0.25386096	0.349	0.295	1	8	Sdhaf2
Abhd2	0.00363134	0.28469868	0.2	0.144	1	8	Abhd2

Mgat4b	0.0036821	0.24160049	0.216	0.153	1	8	Mgat4b
Dnajc5	0.00369106	0.23203438	0.459	0.413	1	8	Dnajc5
Ankrd121	0.00371216	0.5210724	0.616	0.618	1	8	Ankrd12
Cand11	0.00372131	0.36588553	0.349	0.288	1	8	Cand1
Mdm2	0.0037306	0.24624039	0.361	0.309	1	8	Mdm2
Scaper1	0.00397289	0.22244959	0.251	0.186	1	8	Scaper
Dync1h11	0.00413868	0.40465399	0.557	0.533	1	8	Dync1h1
Nfkbid2	0.00440553	0.35182368	0.471	0.423	1	8	Nfkbid
Wsb2	0.00481218	0.20900886	0.263	0.197	1	8	Wsb2
Kmt5a	0.00492618	0.3452816	0.471	0.421	1	8	Kmt5a
Vps13d1	0.00497938	0.23635372	0.204	0.146	1	8	Vps13d
R3hdm2	0.00502262	0.53827777	0.541	0.525	1	8	R3hdm2
Ppfibp21	0.00508349	0.43011158	0.494	0.489	1	8	Ppfibp2
Sppl2a2	0.00544319	0.3498704	0.741	0.776	1	8	Sppl2a
Slain2	0.00571419	0.28040355	0.333	0.266	1	8	Slain2
Irf5	0.00584379	0.29118068	0.741	0.86	1	8	Irf5
Wipf2	0.00599076	0.26117612	0.329	0.279	1	8	Wipf2
Arl8a	0.00603299	0.26333732	0.384	0.344	1	8	Arl8a
Trip121	0.00616108	0.35809637	0.557	0.54	1	8	Trip12
Ddx18	0.00624282	0.22038405	0.392	0.548	1	8	Ddx18
Irf71	0.00631386	0.49394834	0.416	0.59	1	8	Irf7
Katna1	0.00641237	0.27202791	0.357	0.306	1	8	Katna1
Sema4a1	0.00641896	0.33125871	0.584	0.624	1	8	Sema4a

Ubash3b1	0.00655001	0.36063804	0.467	0.647	1	8	Ubash3b
Mkln1	0.00658568	0.32255316	0.408	0.351	1	8	Mkln1
Fam129a1	0.00680439	0.47484805	0.62	0.637	1	8	Fam129a
Ncoa31	0.00684879	0.40918336	0.514	0.491	1	8	Ncoa3
Il6st	0.00696297	0.31279776	0.227	0.17	1	8	Il6st
Sptan11	0.00802193	0.32285875	0.42	0.368	1	8	Sptan1
Nr1h2	0.0080287	0.28079892	0.506	0.473	1	8	Nr1h2
Ap1g2	0.00809939	0.30508773	0.224	0.171	1	8	Ap1g2
Mtf1	0.00810852	0.31803586	0.322	0.27	1	8	Mtf1
Aup1	0.00838872	0.29330762	0.522	0.51	1	8	Aup1
Mecp2	0.00867916	0.222208	0.243	0.184	1	8	Mecp2
Gigyf1	0.0091038	0.2285285	0.204	0.147	1	8	Gigyf1
Slc35b2	0.00940911	0.22216483	0.271	0.222	1	8	Slc35b2
Washc4	0.00964733	0.35828925	0.361	0.306	1	8	Washc4
Nfkbiz1	3.52E-136	2.08651402	0.914	0.21	6.54E-132	9	Nfkbiz
Dusp22	2.55E-114	2.50104376	0.951	0.309	4.74E-110	9	Dusp2
Tnfaip32	7.08E-97	1.81304614	0.932	0.299	1.32E-92	9	Tnfaip3
Nfkbia2	3.32E-93	2.43747754	1	0.7	6.18E-89	9	Nfkbia
Marcksl11	4.21E-75	1.61209032	0.92	0.392	7.83E-71	9	Marcksl1
Icam12	5.03E-74	1.86174784	0.938	0.526	9.34E-70	9	Icam1
Cxcl101	5.67E-64	0.89530349	0.444	0.074	1.05E-59	9	Cxcl10
Ppp1r15a3	9.96E-64	1.74757901	0.994	0.813	1.85E-59	9	Ppp1r15a
Phlda1	3.34E-63	1.62910517	0.784	0.286	6.21E-59	9	Phlda1

Sowahc1	7.48E-61	1.17395264	0.87	0.369	1.39E-56	9	Sowahc
Gadd45b2	6.41E-59	1.39347488	0.963	0.475	1.19E-54	9	Gadd45b
Cxcl92	2.21E-57	0.9336209	0.512	0.105	4.10E-53	9	Cxcl9
Tnf2	4.07E-57	1.24578606	0.481	0.102	7.56E-53	9	Tnf
Egr12	1.49E-51	1.8422854	0.994	0.783	2.77E-47	9	Egr1
Maff1	8.36E-50	0.9879502	0.846	0.402	1.55E-45	9	Maff
Bcl2a1b1	1.34E-49	1.4858789	0.858	0.408	2.50E-45	9	Bcl2a1b
Zc3h12a1	8.96E-40	0.52244433	0.586	0.19	1.67E-35	9	Zc3h12a
Nfkbid3	2.54E-39	0.90991238	0.827	0.413	4.72E-35	9	Nfkbid
Ier22	9.67E-39	1.33738591	0.981	0.934	1.80E-34	9	Ier2
Birc31	1.97E-38	0.70836282	0.71	0.292	3.67E-34	9	Birc3
Kdm6b1	7.53E-36	0.91810332	0.981	0.846	1.40E-31	9	Kdm6b
Zfp362	7.90E-35	0.97890096	1	0.953	1.47E-30	9	Zfp36
Nfkb12	8.80E-34	0.93065189	0.883	0.583	1.64E-29	9	Nfkb1
Srgn1	3.11E-33	0.65790758	1	0.992	5.77E-29	9	Srgn
Tgif11	3.87E-33	0.62527652	0.562	0.201	7.18E-29	9	Tgif1
Itpkc	4.48E-33	0.29731004	0.241	0.041	8.33E-29	9	Itpkc
Tnfsf91	7.33E-33	0.96367718	0.432	0.128	1.36E-28	9	Tnfsf9
Egr32	1.06E-32	1.05827413	0.944	0.688	1.97E-28	9	Egr3
Mki67	1.73E-32	0.40994705	0.333	0.076	3.22E-28	9	Mki67
Bcl2a1d2	2.85E-32	0.94355026	0.914	0.661	5.29E-28	9	Bcl2a1d
Sdc41	3.10E-31	0.87594024	0.228	0.04	5.77E-27	9	Sdc4
Mcl12	3.27E-31	0.63234477	0.994	0.982	6.08E-27	9	Mcl1

Pim12	9.03E-31	0.67458036	1	0.973	1.68E-26	9	Pim1
Fosb2	3.04E-30	1.01943279	1	0.914	5.65E-26	9	Fosb
Bcl2a1a2	1.52E-29	0.9251692	0.735	0.388	2.83E-25	9	Bcl2a1a
Junb2	2.52E-29	0.6804031	1	0.99	4.68E-25	9	Junb
Rel2	5.78E-29	0.72978474	0.981	0.894	1.07E-24	9	Rel
Ifrd13	9.75E-29	0.79762302	0.957	0.775	1.81E-24	9	Ifrd1
H2-Q61	2.80E-26	0.46914797	0.642	0.273	5.20E-22	9	H2-Q6
Herpud12	3.63E-26	0.94351082	0.914	0.672	6.74E-22	9	Herpud1
Ahnak2	4.55E-24	0.73020472	1	0.948	8.46E-20	9	Ahnak
Etv32	4.88E-24	0.68567517	0.957	0.782	9.08E-20	9	Etv3
Nr4a12	1.65E-23	0.6234926	1	0.947	3.06E-19	9	Nr4a1
Ddx51	4.21E-23	0.51405583	1	0.993	7.83E-19	9	Ddx5
Irf13	6.25E-23	0.62104844	0.648	0.325	1.16E-18	9	Irf1
Jun-02	6.82E-23	1.22449751	0.932	0.819	1.27E-18	9	Jun
Clic42	2.75E-22	0.71348416	0.914	0.752	5.10E-18	9	Clic4
Dnaja12	5.55E-22	0.80308116	0.994	0.968	1.03E-17	9	Dnaja1
Trim351	2.61E-21	0.72050009	0.975	0.882	4.86E-17	9	Trim35
Furin2	7.01E-21	0.90479339	0.809	0.528	1.30E-16	9	Furin
Traf11	7.20E-21	0.6643423	0.846	0.626	1.34E-16	9	Traf1
Rasgef1b1	1.38E-20	0.90781283	0.79	0.555	2.56E-16	9	Rasgef1b
Dnajb12	2.84E-20	1.38406241	0.802	0.633	5.28E-16	9	Dnajb1
4930523C07Rik1	3.45E-20	0.49076454	0.636	0.315	6.41E-16	9	4930523C07Rik
D930030I03Rik1	6.33E-20	0.30605208	0.352	0.117	1.18E-15	9	D930030I03Rik

Marcks2	6.91E-20	0.62641876	0.981	0.928	1.28E-15	9	Marcks
Wsb12	1.21E-19	0.64500398	0.889	0.659	2.25E-15	9	Wsb1
Cd832	1.22E-19	0.61280529	0.994	0.958	2.28E-15	9	Cd83
Hspa51	1.28E-19	0.58698782	1	0.98	2.38E-15	9	Hspa5
Myo1g1	1.45E-19	0.4222681	0.864	0.623	2.70E-15	9	Myo1g
Tbc1d41	2.45E-19	0.66791164	0.87	0.612	4.55E-15	9	Tbc1d4
Prr72	2.55E-19	0.4020842	0.488	0.212	4.74E-15	9	Prr7
Klf10	4.39E-19	0.55990581	0.623	0.316	8.16E-15	9	Klf10
Dok2	5.29E-19	0.30359154	0.315	0.099	9.82E-15	9	Dok2
Neat12	1.37E-18	1.01728902	0.901	0.768	2.54E-14	9	Neat1
Klf64	4.45E-18	0.60138085	0.988	0.926	8.26E-14	9	Klf6
Csrnp11	4.75E-18	0.61174483	0.951	0.768	8.82E-14	9	Csrnp1
Relb1	5.11E-18	0.28324557	0.568	0.28	9.50E-14	9	Relb
Glipr1	1.06E-17	0.67328249	0.809	0.594	1.96E-13	9	Glipr1
Arhgap172	1.22E-17	0.69326448	0.901	0.708	2.26E-13	9	Arhgap17
Dleu21	1.22E-17	0.55895515	0.901	0.706	2.28E-13	9	Dleu2
Map3k82	1.38E-17	0.46690619	0.642	0.342	2.56E-13	9	Map3k8
Gm14636	2.21E-17	0.33024836	0.259	0.078	4.11E-13	9	Gm14636
Ccnl12	2.50E-17	0.59510504	1	0.95	4.64E-13	9	Ccnl1
Cdkn1a2	9.41E-17	0.99816965	0.716	0.46	1.75E-12	9	Cdkn1a
Clcf1	1.24E-16	0.35489975	0.222	0.061	2.30E-12	9	Clcf1
Nrros1	6.94E-16	0.54507798	0.914	0.753	1.29E-11	9	Nrros
Pmaip12	2.31E-15	0.58397138	0.963	0.857	4.29E-11	9	Pmaip1

Cd36	3.17E-15	0.38952827	0.58	0.304	5.89E-11	9	Cd36
Klf23	4.45E-15	0.78843647	0.914	0.747	8.28E-11	9	Klf2
Tfe31	6.36E-15	0.33254845	0.494	0.236	1.18E-10	9	Tfe3
Brd22	1.15E-14	0.52644065	0.926	0.811	2.13E-10	9	Brd2
Arf42	6.92E-14	0.4116404	0.988	0.863	1.29E-09	9	Arf4
Sqstm11	8.09E-14	0.8158556	0.796	0.631	1.50E-09	9	Sqstm1
Jup2	1.84E-13	0.44112338	0.512	0.266	3.43E-09	9	Jup
Tgoln11	1.86E-13	0.42864299	0.815	0.645	3.46E-09	9	Tgoln1
H2-Q71	2.00E-13	0.37774037	0.815	0.552	3.72E-09	9	H2-Q7
Nfkb22	2.14E-13	0.38971671	0.673	0.402	3.99E-09	9	Nfkb2
Pabpc12	2.87E-13	0.35535006	1	0.977	5.34E-09	9	Pabpc1
Sh3tc11	3.71E-13	0.29979904	0.494	0.248	6.89E-09	9	Sh3tc1
Mbtd12	4.51E-13	0.58585351	0.66	0.433	8.39E-09	9	Mbtd1
Btg13	6.18E-13	0.43365322	1	0.988	1.15E-08	9	Btg1
Ppp1r102	7.41E-13	0.58815725	0.722	0.518	1.38E-08	9	Ppp1r10
Asap11	7.43E-13	0.4210547	0.907	0.752	1.38E-08	9	Asap1
Rnf19b3	8.08E-13	0.51976923	0.765	0.538	1.50E-08	9	Rnf19b
Bcl101	8.59E-13	0.45469194	0.821	0.645	1.60E-08	9	Bcl10
Sun21	9.82E-13	0.34434268	0.556	0.313	1.82E-08	9	Sun2
H2-K11	1.70E-12	0.26822532	0.994	0.991	3.16E-08	9	H2-K1
Tnip31	1.79E-12	0.49746346	0.352	0.152	3.33E-08	9	Tnip3
Nlrp3	1.84E-12	0.44895345	0.586	0.359	3.42E-08	9	Nlrp3
Casp42	1.97E-12	0.33824294	0.383	0.174	3.66E-08	9	Casp4

Tmem39a1	2.47E-12	0.23541308	0.426	0.198	4.59E-08	9	Tmem39a
Sep-34	3.25E-12	0.40413035	0.951	0.732	6.04E-08	9	Sep-03
C9orf721	3.54E-12	0.25228978	0.383	0.174	6.57E-08	9	C9orf72
Zfp36l13	4.23E-12	0.43445292	0.883	0.713	7.86E-08	9	Zfp36l1
H2-D11	4.61E-12	0.30896393	1	0.997	8.57E-08	9	H2-D1
Ddx3x1	5.51E-12	0.39761751	0.895	0.743	1.02E-07	9	Ddx3x
Ccl61	1.11E-11	0.40527127	0.642	0.398	2.06E-07	9	Ccl6
Jund2	1.16E-11	0.39056742	0.988	0.976	2.15E-07	9	Jund
Cd812	1.29E-11	0.47988779	0.778	0.538	2.39E-07	9	Cd81
Uap11	1.50E-11	0.2902413	0.432	0.224	2.80E-07	9	Uap1
Rap1b1	1.89E-11	0.37085202	0.963	0.929	3.51E-07	9	Rap1b
Egr23	2.24E-11	0.41154585	0.654	0.395	4.16E-07	9	Egr2
Clec4b1	2.64E-11	0.21825268	0.253	0.096	4.91E-07	9	Clec4b1
Sema4a2	2.77E-11	0.31649095	0.821	0.617	5.14E-07	9	Sema4a
Rbpj1	2.85E-11	0.34712154	1	0.959	5.30E-07	9	Rbpj
H2-Q41	2.99E-11	0.23452963	0.525	0.291	5.55E-07	9	H2-Q4
Chd41	3.20E-11	0.52610343	0.87	0.73	5.94E-07	9	Chd4
Ints62	4.61E-11	0.45553197	0.58	0.377	8.57E-07	9	Ints6
Tuba1c1	4.90E-11	0.36078128	0.759	0.537	9.11E-07	9	Tuba1c
Lima12	5.38E-11	0.30470698	0.648	0.402	1.00E-06	9	Lima1
Frmd4b1	8.75E-11	0.24823506	0.333	0.15	1.63E-06	9	Frmd4b
Nfkbib1	8.80E-11	0.37438731	0.71	0.525	1.63E-06	9	Nfkbib
Atp6v0c1	9.62E-11	0.40315082	0.932	0.897	1.79E-06	9	Atp6v0c

Slc8b11	9.71E-11	0.41478525	0.79	0.562	1.80E-06	9	Slc8b1
Rab11fip13	1.24E-10	0.40818714	0.84	0.682	2.31E-06	9	Rab11fip1
Odc12	1.40E-10	0.80172303	0.815	0.71	2.59E-06	9	Odc1
Snhg152	1.45E-10	0.41939719	0.679	0.468	2.69E-06	9	Snhg15
Dennd4a2	1.54E-10	0.58507719	0.981	0.902	2.87E-06	9	Dennd4a
Dusp52	2.40E-10	0.40014751	0.926	0.805	4.45E-06	9	Dusp5
Cd402	3.22E-10	0.35722531	0.327	0.149	5.98E-06	9	Cd40
Chka2	3.28E-10	0.42088934	0.525	0.33	6.09E-06	9	Chka
Lmna3	3.68E-10	0.68591737	0.747	0.549	6.84E-06	9	Lmna
Slc5a32	6.02E-10	0.40773636	0.265	0.114	1.12E-05	9	Slc5a3
Gm36486	7.39E-10	0.22965068	0.333	0.154	1.37E-05	9	Gm36486
Rab71	7.41E-10	0.41797499	0.957	0.875	1.38E-05	9	Rab7
Pcf112	8.19E-10	0.43672731	0.654	0.471	1.52E-05	9	Pcf11
Baiap2	1.20E-09	0.34227728	0.519	0.322	2.24E-05	9	Baiap2
Alcam1	1.42E-09	0.35353681	0.895	0.766	2.65E-05	9	Alcam
Azin11	1.47E-09	0.41278879	0.691	0.531	2.73E-05	9	Azin1
Dnajb92	1.94E-09	0.47626611	0.71	0.56	3.61E-05	9	Dnajb9
Skil2	2.36E-09	0.45544177	0.944	0.884	4.38E-05	9	Skil
Dot1l1	2.41E-09	0.38680008	0.623	0.428	4.48E-05	9	Dot1l
Hivep11	2.56E-09	0.38060251	0.586	0.397	4.76E-05	9	Hivep1
Chd21	2.71E-09	0.42231776	0.704	0.518	5.04E-05	9	Chd2
App1	2.98E-09	0.25879063	0.741	0.54	5.55E-05	9	App
Arid21	3.12E-09	0.32960364	0.506	0.314	5.80E-05	9	Arid2

Ezr2	5.02E-09	0.39385567	0.852	0.744	9.32E-05	9	Ezr
Fgfr14	5.03E-09	0.31024949	0.728	0.5	9.35E-05	9	Fgfr1
Psap1	5.92E-09	0.22742816	1	0.993	0.00011002	9	Psap
Cdk141	6.34E-09	0.32899872	0.846	0.661	0.00011777	9	Cdk14
Ywhaz1	1.03E-08	0.26871521	0.988	0.978	0.00019218	9	Ywhaz
Lcp12	1.07E-08	0.31807111	0.994	0.969	0.00019941	9	Lcp1
Mtmr141	1.20E-08	0.28875188	0.568	0.372	0.00022379	9	Mtmr14
Sertad21	1.32E-08	0.29666997	0.444	0.269	0.00024566	9	Sertad2
Fnbp1l1	1.44E-08	0.28571174	0.488	0.291	0.00026756	9	Fnbp1l
Itpr11	1.45E-08	0.39493627	0.796	0.675	0.00027014	9	Itpr1
Gls1	1.48E-08	0.40432092	0.889	0.748	0.00027534	9	Gls
Nabp12	1.72E-08	0.34604646	0.759	0.59	0.00031982	9	Nabp1
Spag92	1.75E-08	0.42222365	1	0.961	0.00032479	9	Spag9
Hspa1a4	2.02E-08	0.78665332	0.79	0.636	0.00037489	9	Hspa1a
Erbin1	2.24E-08	0.35686347	0.679	0.495	0.00041698	9	Erbin
Rbbp81	2.24E-08	0.36261839	0.741	0.56	0.00041708	9	Rbbp8
Ryr1	2.44E-08	0.22583764	0.519	0.307	0.00045335	9	Ryr1
Hspa1b2	3.02E-08	0.72759755	0.698	0.522	0.00056187	9	Hspa1b
Slfn21	3.18E-08	0.3308913	0.827	0.692	0.00059072	9	Slfn2
Mat2a2	3.63E-08	0.3723249	0.84	0.71	0.00067355	9	Mat2a
Elp5	4.31E-08	0.24756975	0.593	0.391	0.00080165	9	Elp5
Rela2	4.66E-08	0.21146501	0.58	0.383	0.00086493	9	Rela
Cd2261	4.84E-08	0.23389928	0.574	0.366	0.00089993	9	Cd226

Btg22	5.37E-08	0.26358112	1	0.982	0.00099803	9	Btg2
Arl13b1	7.10E-08	0.28573785	0.426	0.253	0.00131834	9	Arl13b
Hsph12	7.60E-08	0.5704226	0.593	0.44	0.0014119	9	Hsph1
Fos4	7.89E-08	0.49706711	1	0.976	0.00146528	9	Fos
Cited23	8.23E-08	0.49859756	0.623	0.458	0.00152837	9	Cited2
Xist2	1.07E-07	0.36329023	0.858	0.668	0.00198882	9	Xist
Cadm13	1.08E-07	0.35075709	0.654	0.445	0.00200473	9	Cadm1
Tmco3	1.19E-07	0.21222066	0.29	0.144	0.0022126	9	Tmco3
Mbnl21	1.21E-07	0.38414073	0.907	0.822	0.00224781	9	Mbnl2
Grap3	1.24E-07	0.25473529	0.438	0.254	0.00229677	9	Grap
Dusp13	1.29E-07	0.30301198	0.975	0.883	0.00239875	9	Dusp1
Hsp90aa13	1.64E-07	0.42362569	1	0.975	0.00304134	9	Hsp90aa1
Arl5b1	1.75E-07	0.228095	0.556	0.362	0.00324853	9	Arl5b
Mbnl11	2.07E-07	0.23782922	0.988	0.961	0.00385083	9	Mbnl1
Pnrc12	2.15E-07	0.28818657	0.944	0.88	0.00399945	9	Pnrc1
Rbm38	2.27E-07	0.26380396	0.432	0.269	0.00421183	9	Rbm38
Nrp11	2.63E-07	0.34682937	0.599	0.43	0.00489246	9	Nrp1
Camk1d1	2.72E-07	0.28199771	0.963	0.82	0.00505626	9	Camk1d
Zc3h12c1	3.58E-07	0.28253369	0.321	0.177	0.0066476	9	Zc3h12c
Naaa3	3.69E-07	0.26764665	1	0.956	0.00686028	9	Naaa
Tut71	4.11E-07	0.32896029	0.858	0.735	0.00764018	9	Tut7
Rab8b1	4.29E-07	0.25255354	0.975	0.872	0.00797398	9	Rab8b
Lilr4b1	4.37E-07	0.21301565	0.426	0.252	0.00812717	9	Lilr4b

Bag32	4.95E-07	0.25135193	0.29	0.151	0.00919935	9	Bag3
Cfh1	5.41E-07	0.24897257	0.346	0.191	0.01004782	9	Cfh
Ppp1r16b1	5.57E-07	0.22434734	0.315	0.167	0.01035205	9	Ppp1r16b
Tubb61	5.61E-07	0.29771296	0.66	0.503	0.01043152	9	Tubb6
Qpct	6.86E-07	0.24866292	0.525	0.342	0.0127426	9	Qpct
Prkcd2	7.69E-07	0.29173181	0.741	0.591	0.01429572	9	Prkcd
Pianp4	8.75E-07	0.22909992	0.605	0.401	0.01624966	9	Pianp
Macf12	9.37E-07	0.23681719	0.735	0.548	0.01741239	9	Macf1
Ear2	9.48E-07	0.27928266	0.309	0.164	0.01761944	9	Ear2
Cks21	9.50E-07	0.23688633	0.358	0.199	0.01764762	9	Cks2
Cyp2s1	9.58E-07	0.20929157	0.253	0.125	0.01780609	9	Cyp2s1
Mndal1	1.14E-06	0.22338281	0.92	0.789	0.02112789	9	Mndal
Srrm22	1.39E-06	0.26485189	0.988	0.95	0.02581851	9	Srrm2
Ncl2	1.46E-06	0.27815836	0.957	0.953	0.02712512	9	Ncl
Gpr1322	1.59E-06	0.25299991	0.815	0.699	0.02952691	9	Gpr132
Aim21	1.60E-06	0.25399925	0.63	0.477	0.02975766	9	Aim2
Golph31	1.62E-06	0.23893675	0.673	0.513	0.0300918	9	Golph3
G3bp11	1.69E-06	0.31098405	0.87	0.774	0.03145374	9	G3bp1
Tgfb12	2.00E-06	0.3285582	0.821	0.704	0.03720139	9	Tgfb1
Gpbp11	2.09E-06	0.27056161	0.815	0.651	0.03889169	9	Gpbp1
Rap2a1	2.16E-06	0.30001694	0.5	0.348	0.04019236	9	Rap2a
Lipa	2.38E-06	0.24169382	0.469	0.315	0.04430688	9	Lipa
Ddit31	2.45E-06	0.20907503	0.556	0.373	0.04557531	9	Ddit3

Lilrb4a2	2.92E-06	0.23425476	0.407	0.25	0.05421473	9	Lilrb4a
Napsa2	3.33E-06	0.2678467	0.981	0.963	0.06184391	9	Napsa
Itgal1	3.33E-06	0.30323256	0.765	0.576	0.06184694	9	Itgal
Pik3cd1	3.63E-06	0.27436611	0.66	0.49	0.067396	9	Pik3cd
Peli12	3.84E-06	0.29153724	0.753	0.611	0.07125484	9	Peli1
Sertad11	3.96E-06	0.34198102	0.5	0.352	0.07360754	9	Sertad1
Gm367231	4.06E-06	0.31871541	0.519	0.376	0.07537199	9	Gm36723
Hexim11	4.28E-06	0.28396731	0.5	0.346	0.07957977	9	Hexim1
Sbno12	4.73E-06	0.25826293	0.648	0.481	0.08786277	9	Sbno1
Ltb4r1	4.78E-06	0.29499741	0.395	0.232	0.08885055	9	Ltb4r1
Arih11	5.09E-06	0.35699924	0.741	0.614	0.09454395	9	Arih1
Anxa61	5.18E-06	0.23981319	0.932	0.822	0.09630592	9	Anxa6
Trerf11	5.55E-06	0.22033954	0.809	0.636	0.10312417	9	Trerf1
Tob11	5.60E-06	0.28130582	0.42	0.273	0.10411537	9	Tob1
Pfkp3	5.68E-06	0.26005675	0.988	0.884	0.10560943	9	Pfkp
Ehd11	5.78E-06	0.23480326	0.426	0.272	0.10744473	9	Ehd1
Mapkapk2	5.93E-06	0.30488915	0.722	0.575	0.11021265	9	Mapkapk2
Hsp90ab11	6.10E-06	0.2544879	0.994	0.996	0.11335571	9	Hsp90ab1
Eif52	6.45E-06	0.23230461	0.963	0.918	0.11986589	9	Eif5
Pcna1	6.61E-06	0.29713973	0.747	0.601	0.12284835	9	Pcna
Sde21	7.75E-06	0.24115054	0.759	0.573	0.14398234	9	Sde2
Ptbp1	8.41E-06	0.27970695	0.691	0.577	0.15627673	9	Ptbp1
Anpep2	9.04E-06	0.20141646	0.796	0.622	0.16791436	9	Anpep

Arid4a2	9.04E-06	0.25191642	0.883	0.806	0.16793872	9	Arid4a
Iqgap12	9.14E-06	0.23907456	1	0.966	0.16989066	9	Iqgap1
Prpf40a	9.52E-06	0.24077546	0.846	0.716	0.17684951	9	Prpf40a
Slc6a62	9.68E-06	0.30029221	0.79	0.727	0.17980177	9	Slc6a6
Senp21	9.75E-06	0.23759499	0.586	0.433	0.18108354	9	Senp2
Txnip	9.80E-06	0.24122058	0.586	0.424	0.18217547	9	Txnip
Med131	1.08E-05	0.23209153	0.531	0.378	0.20126853	9	Med13
Arhgap221	1.31E-05	0.26561051	0.556	0.415	0.24421395	9	Arhgap22
Pik3cb2	1.37E-05	0.22943181	0.796	0.652	0.25503676	9	Pik3cb
Tmem2481	1.75E-05	0.21551274	0.506	0.366	0.32503996	9	Tmem248
Colgalt11	1.82E-05	0.23526948	0.741	0.58	0.33768087	9	Colgalt1
Mrps6	1.82E-05	0.22952621	0.401	0.266	0.33879833	9	Mrps6
Bhlhe401	1.85E-05	0.25805439	0.642	0.49	0.34449406	9	Bhlhe40
Lmo1	1.89E-05	0.26305298	0.475	0.339	0.35199507	9	Lmo1
Adam82	1.99E-05	0.25634571	0.772	0.603	0.36984177	9	Adam8
Midn2	2.10E-05	0.26546672	0.63	0.496	0.39083557	9	Midn
Emp31	2.15E-05	0.20322666	0.926	0.857	0.39988545	9	Emp3
Stmn11	2.19E-05	0.27208562	0.346	0.224	0.40749949	9	Stmn1
Ranbp22	2.26E-05	0.24716217	0.84	0.755	0.42026348	9	Ranbp2
Resf12	2.47E-05	0.29036519	0.611	0.483	0.4591821	9	Resf1
Cbfa2t34	2.76E-05	0.2215676	0.957	0.901	0.5136585	9	Cbfa2t3
Rbms12	3.10E-05	0.22922212	0.747	0.611	0.57632902	9	Rbms1
Efhd21	3.14E-05	0.21169468	0.981	0.953	0.58312289	9	Efhd2

Zc3h7a1	3.63E-05	0.23437	0.667	0.508	0.67362205	9	Zc3h7a
Rnase62	3.68E-05	0.23000288	0.944	0.866	0.68365228	9	Rnase6
Zmiz11	3.92E-05	0.20811692	0.765	0.659	0.72869911	9	Zmiz1
Sf3b11	3.97E-05	0.29172869	0.963	0.947	0.73782159	9	Sf3b1
Hmgb22	4.10E-05	0.32939401	0.951	0.87	0.76211736	9	Hmgb2
Nop582	4.21E-05	0.26331483	0.815	0.729	0.78213021	9	Nop58
Atp1b32	4.65E-05	0.23408342	0.877	0.763	0.86355273	9	Atp1b3
Clk12	4.74E-05	0.27334492	0.957	0.901	0.88130475	9	Clk1
Zfp8001	4.82E-05	0.27693611	0.667	0.531	0.89485488	9	Zfp800
Eif3a1	5.29E-05	0.22572215	0.951	0.89	0.98327562	9	Eif3a
Atp8b41	5.71E-05	0.23795939	0.617	0.472	1	9	Atp8b4
Hnrnph11	6.13E-05	0.24676299	0.722	0.603	1	9	Hnrnph1
Pdlim51	6.38E-05	0.27634163	0.611	0.465	1	9	Pdlim5
Zc3hav12	7.02E-05	0.24525462	0.698	0.609	1	9	Zc3hav1
Sfpq1	7.48E-05	0.2633227	0.938	0.864	1	9	Sfpq
Katna11	8.27E-05	0.24379013	0.426	0.305	1	9	Katna1
Gm472831	8.35E-05	0.22079154	0.5	0.35	1	9	Gm47283
Plekhm31	8.67E-05	0.20328344	0.623	0.495	1	9	Plekhm3
Srsf22	8.76E-05	0.20637297	0.938	0.906	1	9	Srsf2
Homer11	8.86E-05	0.21813999	0.352	0.23	1	9	Homer1
Gm2a3	9.55E-05	0.25383888	1	0.968	1	9	Gm2a
Bptf1	9.66E-05	0.20131828	0.636	0.499	1	9	Bptf
Socs32	9.74E-05	0.269669	0.549	0.409	1	9	Socs3

Pglyrp11	0.00010788	0.23099591	0.463	0.324	1	9	Pglyrp1
Rbbp61	0.00011667	0.25952541	0.772	0.666	1	9	Rbbp6
Zfp131	0.00012069	0.20946793	0.469	0.336	1	9	Zfp131
Celf21	0.0001244	0.26132939	0.914	0.861	1	9	Celf2
Xcr12	0.00012823	0.2992156	0.846	0.733	1	9	Xcr1
Mpeg11	0.00013252	0.2006816	1	0.973	1	9	Mpeg1
Cd442	0.00013983	0.25352605	0.846	0.7	1	9	Cd44
Mknk21	0.00014007	0.20091714	0.747	0.597	1	9	Mknk2
Ier53	0.0001524	0.21402955	1	0.969	1	9	Ier5
Slk1	0.00015426	0.20886945	0.889	0.785	1	9	Slk
Jak22	0.00016873	0.20058432	0.846	0.752	1	9	Jak2
Dapp11	0.00016877	0.25756339	0.926	0.842	1	9	Dapp1
Slbp	0.00018867	0.39151975	0.506	0.404	1	9	Slbp
Gatad2b1	0.00019121	0.2417624	0.512	0.378	1	9	Gatad2b
Nup1531	0.00020539	0.22475802	0.568	0.442	1	9	Nup153
Fcrla2	0.00021875	0.23273729	0.512	0.366	1	9	Fcrla
Slc25a202	0.00021913	0.28536152	0.827	0.72	1	9	Slc25a20
Tssc41	0.00023196	0.2267479	0.457	0.335	1	9	Tssc4
Pip4k2a1	0.00023706	0.2012403	0.932	0.862	1	9	Pip4k2a
Zfand52	0.00024317	0.28466776	0.926	0.889	1	9	Zfand5
Sh3pxd2b1	0.00029204	0.20850686	0.438	0.312	1	9	Sh3pxd2b
Atf42	0.00030075	0.2782861	0.938	0.895	1	9	Atf4
Ier32	0.00036458	0.2357609	0.531	0.386	1	9	Ier3

Arsb2	0.00037099	0.23071273	0.728	0.617	1	9	Arsb
Rtn42	0.00041816	0.20817544	0.938	0.892	1	9	Rtn4
BC0055371	0.00044172	0.21701997	0.821	0.707	1	9	BC005537
Hnrnpu1	0.00044197	0.21012227	0.914	0.873	1	9	Hnrnpu
Flna2	0.00046681	0.21672454	0.926	0.84	1	9	Flna
Rab432	0.0004683	0.20300083	0.957	0.908	1	9	Rab43
Slamf72	0.00051003	0.21265119	0.932	0.853	1	9	Slamf7
Sod11	0.00051293	0.25496271	0.877	0.806	1	9	Sod1
Ubc2	0.0005514	0.21202909	1	0.991	1	9	Ubc
Gtf2b	0.000602	0.23742879	0.636	0.538	1	9	Gtf2b
Nav11	0.00066844	0.21265164	0.673	0.546	1	9	Nav1
Abcg12	0.00067523	0.20876016	0.463	0.348	1	9	Abcg1
Adgre53	0.00069395	0.26772304	0.802	0.73	1	9	Adgre5
Lrrk21	0.00077707	0.20083518	0.716	0.619	1	9	Lrrk2
Usp161	0.00105077	0.22467137	0.574	0.471	1	9	Usp16
Hk21	0.00108534	0.22520604	0.753	0.651	1	9	Hk2
Sec631	0.00143488	0.20658669	0.537	0.419	1	9	Sec63
Hivep21	0.0018745	0.22836932	0.716	0.621	1	9	Hivep2
Nufip21	0.00200187	0.23887666	0.704	0.612	1	9	Nufip2
Trib11	0.00228918	0.27365895	0.846	0.786	1	9	Trib1
Sirt22	0.00238951	0.21158561	0.617	0.53	1	9	Sirt2
Kdm2b2	0.00401646	0.2550015	0.494	0.418	1	9	Kdm2b
Cxcl21	0.00406188	0.51680265	0.309	0.226	1	9	Cxcl2

Hsp90b11	0.00430258	0.20224628	0.988	0.949	1	9	Hsp90b1
Nasp1	0.0063072	0.20623186	0.469	0.377	1	9	Nasp
Ncoa74	0.0083616	0.24585465	0.864	0.806	1	9	Ncoa7
Klf43	0.00957678	0.26049801	0.784	0.703	1	9	Klf4
Serpina3g	2.04E-188	2.3666988	0.737	0.038	3.80E-184	10	Serpina3g
Gbp51	3.10E-182	1.42961856	0.711	0.035	5.76E-178	10	Gbp5
Serpina3f	3.41E-147	0.52216347	0.25	0.004	6.33E-143	10	Serpina3f
Iigp1	1.57E-134	1.06882817	0.368	0.011	2.92E-130	10	Iigp1
Cxcl93	5.82E-129	4.76678545	0.921	0.106	1.08E-124	10	Cxcl9
Cxcl102	2.40E-95	4.05063229	0.711	0.076	4.47E-91	10	Cxcl10
Gbp21	1.06E-84	0.88495178	0.461	0.033	1.97E-80	10	Gbp2
Cd2741	4.02E-72	1.62287355	0.803	0.129	7.47E-68	10	Cd274
Igtp1	2.00E-64	1.04572917	0.658	0.095	3.71E-60	10	Igtp
Gbp3	4.25E-64	0.64439287	0.395	0.032	7.89E-60	10	Gbp3
Ifi471	1.01E-61	1.36184845	0.789	0.142	1.89E-57	10	Ifi47
Cd403	2.86E-58	1.49570728	0.763	0.146	5.30E-54	10	Cd40
Irf14	1.60E-54	2.29098222	0.974	0.325	2.97E-50	10	Irf1
Calhm61	1.13E-52	0.90281337	0.368	0.034	2.10E-48	10	Calhm6
Gbp7	8.30E-50	0.47831409	0.368	0.036	1.54E-45	10	Gbp7
Tgtp1	9.87E-49	0.42995792	0.276	0.02	1.83E-44	10	Tgtp1
Fcgr4	7.77E-46	0.56818603	0.289	0.024	1.44E-41	10	Fcgr4
Sdc42	1.75E-44	1.02888899	0.368	0.04	3.24E-40	10	Sdc4
Socs12	5.35E-44	1.23650971	0.697	0.16	9.93E-40	10	Socs1

Kynu	1.19E-40	0.69171989	0.408	0.054	2.22E-36	10	Kynu
Cish1	8.55E-37	0.83824752	0.474	0.081	1.59E-32	10	Cish
Sell1	1.22E-34	1.35666187	0.5	0.095	2.26E-30	10	Sell
Ly6a1	5.75E-32	1.39137574	0.566	0.125	1.07E-27	10	Ly6a
Cd691	1.72E-31	0.75950753	0.461	0.085	3.20E-27	10	Cd69
Irgm11	1.64E-30	1.23637777	0.711	0.243	3.05E-26	10	Irgm1
AA4671972	2.03E-30	1.65107514	0.592	0.152	3.76E-26	10	AA467197
Pdcd1lg21	3.27E-29	0.58911297	0.316	0.044	6.08E-25	10	Pdcd1lg2
Ms4a6d1	3.34E-29	1.40654863	0.5	0.116	6.20E-25	10	Ms4a6d
Ccnd23	4.86E-29	1.7100807	0.763	0.276	9.03E-25	10	Ccnd2
Casp43	9.75E-29	0.84082856	0.632	0.174	1.81E-24	10	Casp4
Samhd11	6.70E-27	1.31740846	0.987	0.893	1.24E-22	10	Samhd1
Cd300lf	7.09E-27	0.39399889	0.316	0.048	1.32E-22	10	Cd300lf
Tgtp21	4.40E-25	0.31025223	0.276	0.039	8.18E-21	10	Tgtp2
Gpr1411	9.08E-25	1.02028762	0.632	0.206	1.69E-20	10	Gpr141
Hhex	8.25E-24	1.42227576	0.763	0.346	1.53E-19	10	Hhex
Il10ra3	9.00E-24	0.90190081	0.763	0.304	1.67E-19	10	Il10ra
Slc30a4	1.33E-23	0.5042661	0.342	0.063	2.48E-19	10	Slc30a4
Parp142	7.30E-21	1.0066438	0.75	0.324	1.36E-16	10	Parp14
Ifi2041	2.90E-20	0.85774059	0.697	0.282	5.38E-16	10	Ifi204
Cdkn1a3	7.97E-20	1.48664725	0.829	0.462	1.48E-15	10	Cdkn1a
Pkib2	8.21E-20	1.00185146	0.974	0.823	1.53E-15	10	Pkib
Ppa12	2.70E-19	1.0376139	0.829	0.508	5.02E-15	10	Ppa1

Cd209a1	4.09E-19	1.60957632	0.382	0.093	7.61E-15	10	Cd209a
Armcx61	1.04E-18	0.66614877	0.368	0.089	1.93E-14	10	Armcx6
Glipr21	5.16E-18	0.59762564	0.632	0.245	9.58E-14	10	Glipr2
Pnp2	6.43E-18	0.9797624	0.947	0.771	1.19E-13	10	Pnp
Ms4a4c2	1.79E-17	1.19345537	0.618	0.246	3.32E-13	10	Ms4a4c
Ifi2051	2.62E-17	0.84473661	1	0.916	4.86E-13	10	Ifi205
Bcl2a1b2	4.33E-17	1.24982596	0.803	0.416	8.05E-13	10	Bcl2a1b
Atp6v0a11	6.59E-17	0.64102927	0.461	0.144	1.22E-12	10	Atp6v0a1
Nampt2	9.94E-17	0.83252889	0.842	0.559	1.85E-12	10	Nampt
Stx111	1.45E-16	0.4324997	0.395	0.107	2.69E-12	10	Stx11
Socs33	2.05E-16	0.96603295	0.776	0.408	3.81E-12	10	Socs3
Slamf8	2.62E-16	0.85408299	0.724	0.371	4.88E-12	10	Slamf8
Ly6e4	8.46E-16	0.80517612	0.974	0.788	1.57E-11	10	Ly6e
Tnip32	9.95E-16	1.04487733	0.461	0.153	1.85E-11	10	Tnip3
Zbp11	1.54E-15	1.00326367	0.816	0.506	2.85E-11	10	Zbp1
Slfn22	1.99E-15	1.03082382	0.921	0.693	3.69E-11	10	Slfn2
Eif5a3	2.21E-15	0.67736919	0.987	0.965	4.10E-11	10	Eif5a
Fdps3	2.52E-15	0.94013684	0.842	0.453	4.68E-11	10	Fdps
Apobec32	3.08E-15	0.70566998	0.921	0.734	5.72E-11	10	Apobec3
Ms4a6c2	5.75E-15	1.16787713	0.895	0.644	1.07E-10	10	Ms4a6c
Malt11	6.21E-15	0.95419274	0.632	0.311	1.15E-10	10	Malt1
Traf12	7.16E-15	0.79902784	0.882	0.628	1.33E-10	10	Traf1
Tagap	8.95E-15	0.87162895	0.461	0.162	1.66E-10	10	Tagap

Fcgr11	1.73E-14	0.52991378	0.303	0.074	3.22E-10	10	Fcgr1
Kit1	1.92E-14	0.880333	0.855	0.633	3.57E-10	10	Kit
Mefv	2.47E-14	0.34625435	0.237	0.048	4.58E-10	10	Mefv
Nfkbia3	3.10E-14	0.72694061	0.947	0.705	5.76E-10	10	Nfkbia
Lilrb4a3	4.34E-14	0.81741835	0.592	0.25	8.06E-10	10	Lilrb4a
Denr1	4.60E-14	0.7700463	0.724	0.423	8.55E-10	10	Denr
Tes2	5.21E-14	0.82166027	0.855	0.617	9.68E-10	10	Tes
Ifitm33	5.59E-14	1.05255277	0.987	0.921	1.04E-09	10	Ifitm3
Stat22	5.69E-14	0.65727044	0.842	0.504	1.06E-09	10	Stat2
Rab3il1	5.97E-14	0.29362545	0.25	0.054	1.11E-09	10	Rab3il1
Psma73	1.51E-13	0.56611567	0.987	0.951	2.81E-09	10	Psma7
Ccl171	1.57E-13	2.07131892	0.237	0.05	2.91E-09	10	Ccl17
Mxd13	2.95E-13	1.03179433	0.658	0.319	5.47E-09	10	Mxd1
Gpr18	4.01E-13	0.36235267	0.224	0.047	7.45E-09	10	Gpr18
Slc4a81	5.98E-13	0.23981715	0.211	0.042	1.11E-08	10	Slc4a8
Il4ra2	1.40E-12	0.57562939	0.684	0.346	2.61E-08	10	Il4ra
Ifi2071	1.41E-12	0.74380122	0.921	0.725	2.62E-08	10	Ifi207
Rmdn3	1.67E-12	0.42134358	0.355	0.11	3.10E-08	10	Rmdn3
Ccr51	2.62E-12	0.76641175	0.592	0.282	4.87E-08	10	Ccr5
Stat12	3.27E-12	0.94678336	0.868	0.669	6.08E-08	10	Stat1
Ifi304	3.31E-12	0.80326364	0.974	0.951	6.15E-08	10	Ifi30
Pml1	3.44E-12	0.57130129	0.605	0.27	6.39E-08	10	Pml
Oasl22	3.50E-12	0.53996418	0.289	0.079	6.51E-08	10	Oasl2

Kmo1	4.69E-12	0.83294229	0.737	0.506	8.72E-08	10	Kmo
Tmem1312	4.96E-12	0.68199649	0.776	0.469	9.21E-08	10	Tmem131
Irf72	1.01E-11	0.76083622	0.829	0.579	1.87E-07	10	Irf7
Rasgef1b2	3.33E-11	0.6843166	0.842	0.558	6.19E-07	10	Rasgef1b
Oasl12	3.79E-11	0.54443673	0.342	0.107	7.04E-07	10	Oasl1
Fam241a1	4.39E-11	0.60397502	0.645	0.353	8.16E-07	10	Fam241a
Bcl31	6.26E-11	0.53171306	0.539	0.259	1.16E-06	10	Bcl3
Ms4a6b2	1.46E-10	0.62426507	0.487	0.216	2.72E-06	10	Ms4a6b
Gadd45g2	1.73E-10	1.03487173	0.618	0.357	3.21E-06	10	Gadd45g
S100a63	1.85E-10	1.13676218	0.974	0.886	3.43E-06	10	S100a6
Arid5a1	1.87E-10	0.56706357	0.513	0.238	3.47E-06	10	Arid5a
Gm40701	2.17E-10	0.44568263	0.395	0.156	4.03E-06	10	Gm4070
Txnrd11	2.68E-10	0.72250445	0.632	0.352	4.98E-06	10	Txnrd1
Ndufb1-ps3	2.81E-10	0.44402382	0.961	0.951	5.21E-06	10	Ndufb1-ps
Psme22	4.47E-10	0.48322492	0.974	0.943	8.30E-06	10	Psme2
Rel3	4.76E-10	0.7774813	0.947	0.896	8.85E-06	10	Rel
Cd801	6.75E-10	0.34484009	0.447	0.179	1.25E-05	10	Cd80
Nfkb13	7.87E-10	0.64052587	0.816	0.588	1.46E-05	10	Nfkb1
Jak23	8.16E-10	0.66087475	0.895	0.753	1.52E-05	10	Jak2
Snx202	9.02E-10	0.59272153	0.921	0.83	1.68E-05	10	Snx20
Mkrn11	1.06E-09	0.58007226	0.882	0.789	1.98E-05	10	Mkrn1
Tspo1	1.65E-09	0.57213083	1	0.976	3.06E-05	10	Tspo
Atp6v0c2	1.80E-09	0.57108409	0.961	0.897	3.34E-05	10	Atp6v0c

Slfn52	2.10E-09	0.71915889	0.895	0.769	3.91E-05	10	Slfn5
Tuba1b2	2.13E-09	0.59472337	0.842	0.691	3.96E-05	10	Tuba1b
S100a43	2.61E-09	0.99101617	0.724	0.443	4.84E-05	10	S100a4
Gatm1	2.90E-09	0.69867558	0.855	0.735	5.38E-05	10	Gatm
Clec2d2	2.90E-09	0.67578377	0.645	0.416	5.40E-05	10	Clec2d
Nfkb23	2.95E-09	0.58732693	0.645	0.407	5.48E-05	10	Nfkb2
Scimp2	4.13E-09	0.65398469	0.816	0.585	7.68E-05	10	Scimp
Ctsz2	4.88E-09	0.5058905	0.974	0.964	9.06E-05	10	Ctsz
Cox7b2	6.25E-09	0.40247444	0.921	0.929	0.00011609	10	Cox7b
Bach11	6.79E-09	0.8167347	0.697	0.48	0.00012624	10	Bach1
Lpcat22	6.96E-09	0.33965426	0.382	0.15	0.00012926	10	Lpcat2
Slc22a15	7.01E-09	0.26989232	0.276	0.089	0.00013017	10	Slc22a15
Pde4b3	7.28E-09	0.78853035	0.803	0.571	0.00013529	10	Pde4b
Abcb1b1	8.94E-09	0.3556551	0.303	0.106	0.0001661	10	Abcb1b
Tap13	9.21E-09	0.47470811	0.921	0.894	0.00017117	10	Tap1
Hk22	9.77E-09	0.62273741	0.829	0.651	0.00018155	10	Hk2
Lilr4b2	1.16E-08	0.81155429	0.5	0.254	0.00021499	10	Lilr4b
Cycs3	1.19E-08	0.4898992	0.947	0.907	0.00022113	10	Cycs
Dapp12	1.24E-08	0.64559509	0.934	0.843	0.00022976	10	Dapp1
Arrdc4	1.27E-08	0.37793991	0.474	0.229	0.00023639	10	Arrdc4
Sowahc2	1.35E-08	0.50860085	0.645	0.38	0.00025084	10	Sowahc
1810037I17Rik	1.54E-08	0.54606644	0.908	0.852	0.00028575	10	1810037I17Rik
Morf4l1	1.59E-08	0.39742595	1	0.947	0.00029547	10	Morf4l1

Ldlr2	1.84E-08	0.65014269	0.421	0.182	0.00034135	10	Ldlr
Cd862	1.95E-08	0.58340259	0.895	0.811	0.00036317	10	Cd86
Susd62	2.08E-08	0.60634219	0.737	0.527	0.00038608	10	Susd6
Arid5b	2.48E-08	0.39945399	0.474	0.235	0.00046052	10	Arid5b
Hif1a1	2.84E-08	0.71212721	0.711	0.52	0.00052694	10	Hif1a
Isg152	2.85E-08	0.38320853	0.697	0.418	0.00053019	10	Isg15
Noc4l	2.86E-08	0.26864233	0.368	0.153	0.00053229	10	Noc4l
Nme23	3.06E-08	0.4353201	0.961	0.954	0.00056783	10	Nme2
Pim13	3.07E-08	0.57768906	1	0.974	0.00057003	10	Pim1
Casp8	3.21E-08	0.43516969	0.618	0.377	0.00059557	10	Casp8
Sh3glb12	3.46E-08	0.47796034	0.934	0.868	0.00064376	10	Sh3glb1
A930037H05Rik1	3.75E-08	0.28242721	0.211	0.06	0.00069676	10	A930037H05Rik
Nfkbie1	4.20E-08	0.55467003	0.605	0.371	0.00078062	10	Nfkbie
Plac84	4.71E-08	0.87040751	0.842	0.739	0.0008759	10	Plac8
Plet12	4.83E-08	1.52639231	0.368	0.159	0.00089781	10	Plet1
Ywhae1	5.60E-08	0.40181111	0.987	0.955	0.00103956	10	Ywhae
Klrk11	5.86E-08	0.63212666	0.895	0.753	0.00108837	10	Klrk1
Tmsb104	5.90E-08	0.44578324	1	0.994	0.00109537	10	Tmsb10
AI6622702	7.26E-08	0.49358098	0.908	0.782	0.00134809	10	AI662270
Relb2	7.94E-08	0.25277638	0.526	0.285	0.00147474	10	Relb
Tle32	8.00E-08	0.52015951	0.658	0.433	0.0014855	10	Tle3
Treml22	9.92E-08	0.33710582	0.382	0.159	0.00184389	10	Treml2
Rhoh	1.02E-07	0.43435581	0.316	0.123	0.00190052	10	Rhoh

Chd72	1.17E-07	0.65614 57	0.776	0.577	0.00218 2	10	Chd7
Atp7a	1.81E-07	0.41007 617	0.447	0.229	0.00335 711	10	Atp7a
Psma41	2.10E-07	0.40101 079	0.961	0.926	0.00390 213	10	Psma4
Pomp1	2.33E-07	0.38448 723	0.987	0.969	0.00433 509	10	Pomp
Zyx2	2.36E-07	0.46080 442	0.974	0.938	0.00438 237	10	Zyx
Birc32	2.40E-07	0.57513 092	0.539	0.3	0.00446 737	10	Birc3
Clic43	2.54E-07	0.53303 947	0.882	0.755	0.00471 812	10	Clic4
Icam13	3.08E-07	0.57743 579	0.763	0.535	0.00572 242	10	Icam1
Cox171	3.54E-07	0.53443 448	0.895	0.925	0.00657 99	10	Cox17
Ifi2111	3.96E-07	0.44403 804	0.895	0.772	0.00736 347	10	Ifi211
Cd300a1	4.74E-07	0.96501 032	0.618	0.451	0.00880 196	10	Cd300a
Ptpn21	4.98E-07	0.60788 491	0.671	0.497	0.00925 797	10	Ptpn2
Tceanc2	5.00E-07	0.52906 714	0.5	0.29	0.00928 803	10	Tceanc2
Rnf19b4	6.00E-07	0.53231 627	0.711	0.542	0.01115 135	10	Rnf19b
Arf43	6.50E-07	0.40354 715	0.947	0.866	0.01207 159	10	Arf4
Ak21	6.67E-07	0.45051 848	0.579	0.379	0.01239 628	10	Ak2
Tapbpl1	6.72E-07	0.35028 851	0.539	0.334	0.01248 564	10	Tapbpl
Ifitm12	7.05E-07	0.88652 948	0.632	0.441	0.01310 61	10	Ifitm1
Snrpf3	7.43E-07	0.35582 96	0.947	0.92	0.01379 956	10	Snrpf
Fam49b3	7.46E-07	0.35248 901	0.974	0.939	0.01386 18	10	Fam49b
Tec1	7.97E-07	0.32934 572	0.263	0.098	0.01480 353	10	Tec
Irf51	8.07E-07	0.43570 591	0.908	0.854	0.01499 879	10	Irf5
Prkcd3	1.00E-06	0.67051 226	0.75	0.593	0.01857 582	10	Prkcd

Gm381151	1.10E-06	0.32218152	0.329	0.142	0.02039851	10	Gm38115
Srsf31	1.22E-06	0.35181956	0.974	0.967	0.02272457	10	Srsf3
Gm19951	1.28E-06	0.2409005	0.237	0.082	0.02383751	10	Gm19951
Csf2rb21	1.41E-06	0.37420012	0.342	0.16	0.02612046	10	Csf2rb2
Prpf31	1.48E-06	0.43748373	0.487	0.287	0.02745354	10	Prpf31
Map3k83	1.56E-06	0.43362946	0.592	0.348	0.02906357	10	Map3k8
Idi12	1.61E-06	0.55116692	0.447	0.237	0.02986092	10	Idi1
Arl11	1.67E-06	0.49148133	0.763	0.585	0.03100386	10	Arl1
Jtb2	1.82E-06	0.52738904	0.816	0.714	0.03381946	10	Jtb
Etnk12	1.95E-06	0.51668382	0.579	0.386	0.03628008	10	Etnk1
Ccl42	1.99E-06	0.74993337	0.803	0.645	0.03705897	10	Ccl4
Aig1	2.09E-06	0.39727888	0.342	0.168	0.03891284	10	Aig1
Rnf149	2.20E-06	0.25493931	0.25	0.095	0.04082539	10	Rnf149
Zup12	2.35E-06	0.63359311	0.645	0.434	0.04363934	10	Zup1
Lcp2	2.36E-06	0.38084661	0.382	0.186	0.04389996	10	Lcp2
Trim30a2	2.51E-06	0.50494109	0.908	0.835	0.04665334	10	Trim30a
Slfn81	2.77E-06	0.33397627	0.303	0.13	0.05155795	10	Slfn8
Cmtm6	2.99E-06	0.45148384	0.724	0.56	0.05555451	10	Cmtm6
Lcp13	3.61E-06	0.40575843	1	0.969	0.0671447	10	Lcp1
Rnf2132	3.88E-06	0.32712454	0.711	0.469	0.07217651	10	Rnf213
Bhlhe402	4.00E-06	0.72667906	0.671	0.492	0.07431497	10	Bhlhe40
Adprh	4.25E-06	0.3706551	0.632	0.466	0.0789324	10	Adprh
Tifa	5.48E-06	0.54006665	0.539	0.37	0.10188841	10	Tifa

Dr1	5.79E-06	0.31184036	0.421	0.234	0.10761517	10	Dr1
Ifitm62	5.80E-06	0.63786796	0.329	0.155	0.10769872	10	Ifitm6
Galnt71	6.85E-06	0.47384664	0.447	0.258	0.12722975	10	Galnt7
2610507B11Rik	7.34E-06	0.34013551	0.526	0.319	0.13634685	10	2610507B11Rik
B4galt3	7.95E-06	0.37827742	0.355	0.185	0.14772368	10	B4galt3
Cd833	8.10E-06	0.47601877	0.961	0.959	0.1504521	10	Cd83
Ppia3	8.25E-06	0.29162374	1	0.988	0.15334401	10	Ppia
Gab2	8.43E-06	0.22050713	0.237	0.094	0.15670108	10	Gab2
Psma52	8.76E-06	0.41208363	0.842	0.829	0.16284756	10	Psma5
Anxa53	8.79E-06	0.35782494	0.974	0.887	0.16331181	10	Anxa5
Hivep12	9.51E-06	0.32449163	0.605	0.399	0.17676656	10	Hivep1
Syngr21	1.02E-05	0.48287675	0.947	0.956	0.18864931	10	Syngr2
Arl8b	1.02E-05	0.35617453	0.618	0.44	0.19003897	10	Arl8b
Sav11	1.03E-05	0.26085009	0.368	0.18	0.19177872	10	Sav1
Cox7a22	1.06E-05	0.33059996	0.934	0.921	0.19726513	10	Cox7a2
Mpp11	1.10E-05	0.33218387	0.303	0.139	0.20355436	10	Mpp1
Snhg153	1.13E-05	0.45811473	0.658	0.471	0.21085524	10	Snhg15
B2m2	1.18E-05	0.24299465	0.974	0.986	0.21955142	10	B2m
Gyg1	1.25E-05	0.38896892	0.658	0.515	0.23267362	10	Gyg
Ube2f	1.34E-05	0.4052249	0.75	0.601	0.24932787	10	Ube2f
Tpm3	1.50E-05	0.3334645	0.974	0.975	0.27887324	10	Tpm3
Myl12a1	1.78E-05	0.31064842	0.947	0.902	0.3301255	10	Myl12a
Lsm41	1.82E-05	0.35854343	0.908	0.815	0.33788543	10	Lsm4

Atp2c11	1.88E-05	0.45973398	0.513	0.34	0.34866378	10	Atp2c1
Cox5a2	1.91E-05	0.36753093	0.921	0.912	0.35541567	10	Cox5a
Napsa3	2.23E-05	0.38377406	0.987	0.963	0.41383927	10	Napsa
Cfap36	2.25E-05	0.40469519	0.487	0.306	0.4187418	10	Cfap36
Hip1	2.34E-05	0.38566804	0.368	0.196	0.43393013	10	Hip1
Actb2	2.40E-05	0.25807669	1	1	0.44603007	10	Actb
Riok31	2.40E-05	0.46271458	0.75	0.671	0.44603577	10	Riok3
Tnfaip23	2.46E-05	0.55796257	0.408	0.219	0.45635355	10	Tnfaip2
Plgrkt1	2.52E-05	0.32188245	0.553	0.359	0.46882662	10	Plgrkt
Txndc171	2.76E-05	0.42748627	0.855	0.817	0.51356389	10	Txndc17
Daxx1	2.83E-05	0.35550861	0.487	0.299	0.52644405	10	Daxx
Slc4a71	3.01E-05	0.61679738	0.592	0.43	0.55969683	10	Slc4a7
Gadd45b3	3.20E-05	0.39954923	0.711	0.486	0.59479322	10	Gadd45b
Tnfaip33	3.41E-05	0.43000754	0.513	0.314	0.63272271	10	Tnfaip3
Gnb42	3.46E-05	0.64891237	0.461	0.304	0.64196877	10	Gnb4
Lig1	3.49E-05	0.20680426	0.211	0.082	0.64930654	10	Lig1
Slamf73	3.50E-05	0.41408584	0.882	0.855	0.65103348	10	Slamf7
Phf61	3.97E-05	0.33355076	0.434	0.256	0.73778302	10	Phf6
Srgn2	4.34E-05	0.40520578	1	0.992	0.80626389	10	Srgn
Hnrnpk1	4.70E-05	0.28130309	0.961	0.949	0.87334276	10	Hnrnpk
Pgs1	4.76E-05	0.40057049	0.592	0.485	0.88452867	10	Pgs1
Rexo22	5.05E-05	0.47119723	0.724	0.582	0.93835425	10	Rexo2
Sqle1	5.14E-05	0.28401165	0.237	0.102	0.95492569	10	Sqle

Etv62	5.40E-05	0.50232255	0.763	0.662	1	10	Etv6
Ggta11	5.41E-05	0.31894955	0.355	0.186	1	10	Ggta1
Ywhaq1	5.48E-05	0.36884353	0.842	0.814	1	10	Ywhaq
Mdh22	5.64E-05	0.30949406	0.855	0.731	1	10	Mdh2
Sec23b	5.98E-05	0.25773719	0.434	0.24	1	10	Sec23b
Xaf11	5.99E-05	0.32221343	0.539	0.351	1	10	Xaf1
Psma22	6.53E-05	0.32152688	0.921	0.887	1	10	Psma2
Pmepa12	6.56E-05	0.40978057	0.632	0.446	1	10	Pmepa1
Dnaja21	6.60E-05	0.36336728	0.803	0.674	1	10	Dnaja2
Batf31	7.34E-05	0.43390455	0.895	0.892	1	10	Batf3
BC0350441	7.40E-05	0.57347867	0.513	0.366	1	10	BC035044
Suz12	7.81E-05	0.38981908	0.474	0.319	1	10	Suz12
Dtx3l1	8.92E-05	0.37690599	0.566	0.398	1	10	Dtx3l
Sdcbp2	9.18E-05	0.36504328	0.908	0.856	1	10	Sdcbp
Fcho2	9.40E-05	0.40882229	0.566	0.401	1	10	Fcho2
Eif2ak21	9.82E-05	0.36365009	0.618	0.418	1	10	Eif2ak2
Cttnbp2nl1	0.00010218	0.2988778	0.487	0.318	1	10	Cttnbp2nl
Cct31	0.00010677	0.41825758	0.842	0.783	1	10	Cct3
Chmp4b1	0.00010861	0.35580076	0.961	0.931	1	10	Chmp4b
Slfn11	0.00010869	0.24348947	0.329	0.166	1	10	Slfn1
Cd471	0.00010927	0.28479415	1	0.977	1	10	Cd47
Prmt13	0.00010947	0.38733665	0.776	0.657	1	10	Prmt1
Cflar1	0.00011084	0.35134277	0.395	0.245	1	10	Cflar

Rtf2	0.00011848	0.35987468	0.737	0.663	1	10	Rtf2
Rap2a2	0.00012155	0.52916544	0.513	0.35	1	10	Rap2a
Hivep22	0.00012741	0.42035402	0.737	0.622	1	10	Hivep2
Hdac11	0.00012913	0.29445387	0.671	0.508	1	10	Hdac1
Mllt61	0.00012961	0.333022	0.316	0.163	1	10	Mllt6
Atf32	0.00013134	0.45436185	1	0.914	1	10	Atf3
Arap21	0.00013796	0.32467662	0.263	0.13	1	10	Arap2
Pnpt1	0.00013868	0.29740376	0.329	0.177	1	10	Pnpt1
Ass12	0.00014107	0.42173852	0.697	0.558	1	10	Ass1
Ripk11	0.00014194	0.43435486	0.513	0.374	1	10	Ripk1
Nub11	0.00015463	0.3424577	0.618	0.472	1	10	Nub1
Prdx12	0.00015673	0.43670919	0.921	0.92	1	10	Prdx1
Crem1	0.00016016	0.48893838	0.75	0.608	1	10	Crem
Irak2	0.000165	0.35311828	0.395	0.234	1	10	Irak2
Atp5h3	0.00017084	0.24659554	0.961	0.961	1	10	Atp5h
Ranbp12	0.0001753	0.43938224	0.868	0.814	1	10	Ranbp1
Lrrk11	0.00019976	0.32687489	0.579	0.438	1	10	Lrrk1
Cdk2ap23	0.00020353	0.64388491	0.803	0.716	1	10	Cdk2ap2
Cox8a2	0.00020882	0.29952063	0.961	0.965	1	10	Cox8a
Ninj11	0.00021589	0.29323275	0.237	0.108	1	10	Ninj1
Hlx	0.0002277	0.25265589	0.316	0.169	1	10	Hlx
I830077J02Rik1	0.00022831	0.28793803	0.25	0.12	1	10	I830077J02Rik
Mtmr142	0.00023033	0.35812536	0.513	0.376	1	10	Mtmr14

Sumo11	0.00024655	0.34795927	0.855	0.815	1	10	Sumo1
Pdcd52	0.00025277	0.26792608	0.816	0.697	1	10	Pdcd5
Marcks3	0.00025738	0.25395303	0.961	0.929	1	10	Marcks
Flnb1	0.0002614	0.28024637	0.342	0.195	1	10	Flnb
Armcx3	0.00026265	0.34734595	0.342	0.208	1	10	Armcx3
Gpbp12	0.00027619	0.37426396	0.776	0.654	1	10	Gpbp1
Ndufa91	0.00028093	0.34488564	0.671	0.507	1	10	Ndufa9
Diaph11	0.00028418	0.43385514	0.776	0.725	1	10	Diaph1
Dync1i21	0.00029543	0.27083185	0.789	0.715	1	10	Dync1i2
Picalm1	0.00031261	0.44551367	0.961	0.93	1	10	Picalm
Entpd11	0.00032622	0.38479282	0.382	0.237	1	10	Entpd1
Csrp12	0.0003505	0.21639517	0.5	0.335	1	10	Csrp1
Peli13	0.00035269	0.30789027	0.789	0.612	1	10	Peli1
Atp5g13	0.00036735	0.2942256	0.908	0.896	1	10	Atp5g1
4930523C07Rik2	0.00038118	0.2482973	0.5	0.322	1	10	4930523C07Rik
Pdcl3	0.00038699	0.30896694	0.671	0.519	1	10	Pdcl3
Rbx12	0.00038863	0.27243542	0.947	0.904	1	10	Rbx1
Ndufab12	0.00038896	0.31371472	0.855	0.755	1	10	Ndufab1
Mar-52	0.00041087	0.50144721	0.566	0.442	1	10	Mar-05
Tmem59	0.00043107	0.31314684	0.842	0.77	1	10	Tmem59
Ube2l62	0.00043287	0.25512148	0.447	0.277	1	10	Ube2l6
Eif4e2	0.00044085	0.29660293	0.803	0.662	1	10	Eif4e2
Atp11b	0.00045581	0.31004633	0.605	0.473	1	10	Atp11b

Sar1a1	0.00045765	0.37347111	0.684	0.605	1	10	Sar1a
Morc31	0.00053246	0.27803011	0.684	0.53	1	10	Morc3
Polr2c	0.00056823	0.26961107	0.671	0.54	1	10	Polr2c
Csnk2a1	0.00058344	0.33813976	0.803	0.763	1	10	Csnk2a1
Gramd32	0.00059557	0.29043418	0.539	0.407	1	10	Gramd3
Gripap1	0.00061164	0.36935182	0.539	0.391	1	10	Gripap1
Tkt2	0.00061673	0.31179335	0.868	0.811	1	10	Tkt
Wars	0.00062424	0.33001685	0.329	0.195	1	10	Wars
Hnrnph21	0.00063611	0.30424503	0.75	0.63	1	10	Hnrnph2
Tor3a1	0.00063892	0.34339437	0.447	0.314	1	10	Tor3a
Nrros2	0.00065147	0.33436146	0.789	0.757	1	10	Nrros
Eif12	0.00067181	0.26042045	1	0.993	1	10	Eif1
Arid4a3	0.00067587	0.40936913	0.868	0.808	1	10	Arid4a
D16Ertd472e1	0.00072154	0.33818662	0.526	0.363	1	10	D16Ertd472e
Taldo13	0.00074639	0.25614462	0.947	0.97	1	10	Taldo1
Acot9	0.00081892	0.29423016	0.342	0.209	1	10	Acot9
Ndufb22	0.00083552	0.30946368	0.829	0.776	1	10	Ndufb2
Elmo1	0.00085528	0.29004016	0.592	0.433	1	10	Elmo1
Ier33	0.00086015	0.43807527	0.553	0.388	1	10	Ier3
Tmem131l2	0.00086504	0.27158584	0.566	0.424	1	10	Tmem131l
Pfkp4	0.00087246	0.29254087	0.921	0.887	1	10	Pfkp
Psmc51	0.00087526	0.22915226	0.776	0.682	1	10	Psmc5
Map1lc3b	0.00088684	0.33138491	0.882	0.833	1	10	Map1lc3b

Dok21	0.00091476	0.38014347	0.211	0.103	1	10	Dok2
Ctss2	0.00092821	0.31256701	1	0.98	1	10	Ctss
Lsm121	0.00096715	0.29565771	0.763	0.722	1	10	Lsm12
Slc33a11	0.00097925	0.2255896	0.368	0.227	1	10	Slc33a1
Plek2	0.00099286	0.24304396	0.961	0.945	1	10	Plek
Eny21	0.00101863	0.32907542	0.671	0.553	1	10	Eny2
Tomm72	0.0010724	0.20651346	0.934	0.95	1	10	Tomm7
Nfkbiz2	0.00111096	0.45472095	0.355	0.228	1	10	Nfkbiz
Nfe2l23	0.00114809	0.3962728	0.829	0.714	1	10	Nfe2l2
Dnajc2	0.00116685	0.35637448	0.684	0.601	1	10	Dnajc2
Rab14	0.00117648	0.29633028	0.816	0.774	1	10	Rab14
Scpep12	0.00120366	0.3553865	0.487	0.361	1	10	Scpep1
Jaml2	0.00121334	0.3315973	0.908	0.902	1	10	Jaml
Nol72	0.0012278	0.25274516	0.895	0.861	1	10	Nol7
Tor1aip22	0.00122798	0.28267361	0.553	0.4	1	10	Tor1aip2
Nipa2	0.00123623	0.27433764	0.645	0.517	1	10	Nipa2
Hmgcs11	0.00123987	0.56261143	0.368	0.25	1	10	Hmgcs1
Runx11	0.00125741	0.39535707	0.803	0.69	1	10	Runx1
Plekha1	0.00134686	0.27286438	0.526	0.394	1	10	Plekha1
Gsap	0.00137137	0.21454361	0.487	0.335	1	10	Gsap
Gpr1323	0.00146612	0.28998372	0.789	0.701	1	10	Gpr132
Ptpn1	0.00147331	0.48008345	0.829	0.796	1	10	Ptpn1
M6pr3	0.00148959	0.27929196	0.868	0.857	1	10	M6pr

Sec61b3	0.00150182	0.23851206	0.961	0.949	1	10	Sec61b
Gabarapl11	0.00156462	0.21386867	0.408	0.263	1	10	Gabarapl1
Ndufc12	0.00163424	0.26021978	0.829	0.794	1	10	Ndufc1
Usp181	0.00165234	0.31638986	0.461	0.312	1	10	Usp18
Psma3	0.00173133	0.25798646	0.947	0.95	1	10	Psma3
Smap21	0.00175328	0.33493581	0.671	0.551	1	10	Smap2
Trim30d1	0.00175894	0.31019911	0.632	0.505	1	10	Trim30d
Gpr331	0.00182441	0.24131772	0.211	0.106	1	10	Gpr33
Naa251	0.00188312	0.31667952	0.382	0.256	1	10	Naa25
Lap31	0.00189585	0.45355455	0.461	0.357	1	10	Lap3
Cct81	0.00192667	0.20057683	0.895	0.868	1	10	Cct8
Zfand31	0.0019528	0.23377145	0.526	0.4	1	10	Zfand3
H2afj2	0.00197194	0.35215514	0.829	0.766	1	10	H2afj
Pdcd10	0.00204356	0.27112005	0.829	0.786	1	10	Pdcd10
Ikzf12	0.002045	0.43230787	0.684	0.586	1	10	Ikzf1
Ergic2	0.00206526	0.25479559	0.566	0.431	1	10	Ergic2
Sf3b62	0.00206636	0.2341514	0.868	0.818	1	10	Sf3b6
Wfdc172	0.00207378	0.7824486	0.711	0.676	1	10	Wfdc17
Ndufb71	0.00210496	0.21133148	0.921	0.896	1	10	Ndufb7
Psmd82	0.00216541	0.21583544	0.882	0.832	1	10	Psmd8
Setdb21	0.00216841	0.24029313	0.289	0.171	1	10	Setdb2
Pmvk1	0.00220258	0.45636279	0.697	0.616	1	10	Pmvk
Rab8b2	0.00225229	0.25744236	0.882	0.874	1	10	Rab8b

Aida1	0.00225719	0.23904992	0.355	0.221	1	10	Aida
Sc5d1	0.00229083	0.23313094	0.263	0.15	1	10	Sc5d
Casp11	0.00230034	0.2292302	0.421	0.29	1	10	Casp1
Mrpl11	0.00232588	0.32772067	0.579	0.457	1	10	Mrpl11
Ube2a	0.00238399	0.30377666	0.632	0.558	1	10	Ube2a
Psmb93	0.00241431	0.24116949	0.974	0.963	1	10	Psmb9
Pdap13	0.00243839	0.33905305	0.816	0.764	1	10	Pdap1
Fam204a	0.00248137	0.24700657	0.487	0.372	1	10	Fam204a
Rpn11	0.00253339	0.30395966	0.789	0.672	1	10	Rpn1
Stx7	0.00255983	0.27458331	0.763	0.743	1	10	Stx7
Cldnd11	0.00256589	0.51393438	0.842	0.797	1	10	Cldnd1
Sipa1l31	0.00260245	0.20176064	0.474	0.347	1	10	Sipa1l3
Bst21	0.00261597	0.2223711	0.895	0.887	1	10	Bst2
Znhit1	0.00263176	0.24475043	0.605	0.501	1	10	Znhit1
Ier3ip1	0.00275853	0.261936	0.868	0.812	1	10	Ier3ip1
Vps29	0.00286496	0.25876102	0.816	0.709	1	10	Vps29
Ccdc25	0.00291019	0.33486189	0.408	0.288	1	10	Ccdc25
Maff2	0.00291619	0.25924742	0.592	0.412	1	10	Maff
Pitpnb	0.003031	0.21233794	0.526	0.406	1	10	Pitpnb
Ciao2b1	0.00303136	0.24591052	0.697	0.604	1	10	Ciao2b
Zfp3661	0.00303703	0.23515434	0.408	0.287	1	10	Zfp366
Psmb52	0.00304262	0.2073704	0.882	0.864	1	10	Psmb5
Ube2l3	0.0030469	0.23315581	0.882	0.869	1	10	Ube2l3

Csf2rb3	0.00315851	0.24139501	0.776	0.701	1	10	Csf2rb
4932438A13Rik1	0.00320814	0.25725111	0.579	0.44	1	10	4932438A13Rik
Ndrg12	0.00337212	0.32810516	0.5	0.368	1	10	Ndrg1
Dcun1d5	0.00344636	0.23061081	0.75	0.66	1	10	Dcun1d5
Cd531	0.00347183	0.2833333	0.947	0.906	1	10	Cd53
Ly861	0.00348388	0.37960733	0.842	0.783	1	10	Ly86
G3bp12	0.00354222	0.28758124	0.816	0.776	1	10	G3bp1
Atp5g31	0.0036834	0.27963542	0.882	0.877	1	10	Atp5g3
Atp13a1	0.00376109	0.24218861	0.303	0.183	1	10	Atp13a1
Tagln21	0.00377243	0.4010508	0.921	0.958	1	10	Tagln2
Nsmce1	0.00378563	0.2385562	0.513	0.416	1	10	Nsmce1
Emilin21	0.00378993	0.34572358	0.224	0.119	1	10	Emilin2
Ndufa15	0.0039362	0.21769329	0.868	0.878	1	10	Ndufa1
Aff11	0.00396022	0.28051953	0.658	0.602	1	10	Aff1
Lmo41	0.00397144	0.23670972	0.553	0.438	1	10	Lmo4
Tet21	0.0040228	0.27107644	0.605	0.479	1	10	Tet2
Gosr21	0.0040833	0.21696992	0.618	0.505	1	10	Gosr2
Ptprc1	0.00418614	0.2834717	0.987	0.96	1	10	Ptprc
Ube2i1	0.00419214	0.2207022	0.842	0.834	1	10	Ube2i
Snx8	0.00443026	0.22614757	0.289	0.182	1	10	Snx8
Cltc1	0.00453674	0.2271546	0.829	0.737	1	10	Cltc
Csrnp12	0.00454604	0.3953244	0.803	0.773	1	10	Csrnp1
Cggbp1	0.00456706	0.25939515	0.789	0.718	1	10	Cggbp1

Psmb101	0.00460484	0.30020565	0.895	0.837	1	10	Psmb10
Fndc3a1	0.00462937	0.20201097	0.566	0.424	1	10	Fndc3a
Rbbp82	0.00466021	0.37728769	0.658	0.564	1	10	Rbbp8
Rtraf1	0.0047951	0.21428954	0.855	0.882	1	10	Rtraf
Rela3	0.00489316	0.24763463	0.513	0.387	1	10	Rela
Eif4a11	0.00498876	0.22694344	0.974	0.97	1	10	Eif4a1
Mpc13	0.00523902	0.26053645	0.842	0.78	1	10	Mpc1
Vav11	0.00524487	0.25058588	0.566	0.468	1	10	Vav1
Rwdd12	0.00529644	0.25641829	0.776	0.738	1	10	Rwdd1
Pim3	0.00532162	0.31648736	0.355	0.245	1	10	Pim3
Prkd3	0.00552659	0.24563446	0.447	0.34	1	10	Prkd3
Hnrnpc1	0.00563767	0.26594918	0.908	0.884	1	10	Hnrnpc
Snx6	0.00565897	0.34850687	0.632	0.561	1	10	Snx6
Abracl2	0.00570543	0.2248096	0.882	0.898	1	10	Abracl
Capza21	0.00589544	0.21097831	0.961	0.958	1	10	Capza2
Tmem68	0.0059135	0.26719988	0.211	0.117	1	10	Tmem68
Pdha1	0.00597807	0.30880268	0.355	0.255	1	10	Pdha1
Mndal2	0.00608679	0.28383584	0.855	0.792	1	10	Mndal
Bbx1	0.00615615	0.31914558	0.526	0.451	1	10	Bbx
Vdac21	0.00634689	0.27450304	0.908	0.906	1	10	Vdac2
Batf	0.00644676	0.33307188	0.342	0.22	1	10	Batf
Cops8	0.0064483	0.21494063	0.618	0.488	1	10	Cops8
Sys1	0.00652575	0.26047605	0.75	0.686	1	10	Sys1

Pfdn11	0.00654548	0.22902688	0.618	0.527	1	10	Pfdn1
Grb21	0.00659409	0.21003487	0.908	0.924	1	10	Grb2
Trap1	0.00665121	0.20602128	0.355	0.239	1	10	Trap1
mt-Atp61	0.00694217	0.24503292	1	0.996	1	10	mt-Atp6
Nfil32	0.00704487	0.41317003	0.579	0.5	1	10	Nfil3
Pdk3	0.00706727	0.21367093	0.316	0.214	1	10	Pdk3
B930036N10Rik1	0.00707355	0.22029187	0.421	0.309	1	10	B930036N10Rik
Eif6	0.00724877	0.25736876	0.789	0.728	1	10	Eif6
Snrnp27	0.00729017	0.22378913	0.737	0.643	1	10	Snrnp27
Banf1	0.00733703	0.28316223	0.724	0.641	1	10	Banf1
Tuba1a1	0.00746568	0.23480721	0.724	0.675	1	10	Tuba1a
Creb11	0.00752024	0.22084307	0.447	0.329	1	10	Creb1
Psmb83	0.00755433	0.21152003	0.987	0.986	1	10	Psmb8
Ilrun2	0.00771997	0.21781659	0.5	0.389	1	10	Ilrun
Bcl2l111	0.00775465	0.28212204	0.566	0.45	1	10	Bcl2l11
Tmem2582	0.00782715	0.23045844	0.908	0.876	1	10	Tmem258
Nab11	0.00802285	0.25508578	0.553	0.451	1	10	Nab1
Psmb71	0.00807389	0.21932128	0.763	0.754	1	10	Psmb7
Il1b2	0.00825789	0.5477885	0.921	0.896	1	10	Il1b
Nfya	0.00828612	0.25071945	0.461	0.332	1	10	Nfya
Tor1aip12	0.00836858	0.23848765	0.895	0.829	1	10	Tor1aip1
Pno11	0.00847834	0.28907623	0.408	0.309	1	10	Pno1
Hnrnpa32	0.00866484	0.244455	0.947	0.953	1	10	Hnrnpa3

Cyba2	0.00873073	0.21726826	0.987	0.987	1	10	Cyba
Zfp622	0.00874324	0.31906377	0.632	0.535	1	10	Zfp622
Eif1a2	0.00924996	0.20453522	0.908	0.789	1	10	Eif1a
Ogfr1	0.00940109	0.20428165	0.632	0.541	1	10	Ogfr
Lgals13	0.0096361	0.37046208	0.947	0.884	1	10	Lgals1
Pebp11	0.00968453	0.25400135	0.789	0.765	1	10	Pebp1
Gm46224	0.00984032	0.21906621	0.316	0.215	1	10	Gm46224
Lta4h2	0.00987838	0.36608401	0.461	0.355	1	10	Lta4h
mt-Atp62	2.33E-38	2.51333521	1	0.996	4.32E-34	11	mt-Atp6
mt-Co31	4.87E-38	2.14240119	1	0.999	9.05E-34	11	mt-Co3
mt-Co12	1.20E-37	1.88241445	1	0.998	2.24E-33	11	mt-Co1
mt-Co21	2.35E-37	2.22513958	1	0.998	4.37E-33	11	mt-Co2
mt-Nd2	4.68E-37	2.39320372	1	0.977	8.70E-33	11	mt-Nd2
mt-Cytb1	2.04E-34	2.39930912	1	0.989	3.78E-30	11	mt-Cytb
mt-Nd11	2.43E-34	2.0762448	1	0.986	4.51E-30	11	mt-Nd1
mt-Nd4	2.82E-33	1.93257608	1	0.987	5.24E-29	11	mt-Nd4
Psap2	1.20E-26	1.1422832	1	0.994	2.22E-22	11	Psap
mt-Nd4l	5.16E-26	2.08667627	0.95	0.932	9.59E-22	11	mt-Nd4l
mt-Atp8	1.08E-23	1.89655035	0.917	0.892	2.01E-19	11	mt-Atp8
Spag93	3.08E-22	1.63656655	0.933	0.962	5.73E-18	11	Spag9
Rrbp11	4.46E-22	1.71240541	0.933	0.931	8.28E-18	11	Rrbp1
Malat13	5.83E-22	1.26970354	1	1	1.08E-17	11	Malat1
mt-Nd31	6.63E-22	1.98570955	0.917	0.944	1.23E-17	11	mt-Nd3

Pdia61	5.41E-21	1.80216093	0.917	0.856	1.01E-16	11	Pdia6
Son	1.21E-20	1.21084633	0.95	0.979	2.26E-16	11	Son
mt-Nd5	1.42E-20	1.62842422	0.917	0.926	2.64E-16	11	mt-Nd5
Hsp90b12	7.74E-20	1.82630257	0.983	0.95	1.44E-15	11	Hsp90b1
Hspa52	2.10E-18	1.70541452	0.95	0.981	3.89E-14	11	Hspa5
Rbm391	3.58E-18	1.22170466	0.933	0.986	6.65E-14	11	Rbm39
Slc38a22	4.97E-18	1.77049508	0.867	0.857	9.23E-14	11	Slc38a2
Ptprc2	5.28E-15	1.37259918	0.9	0.961	9.81E-11	11	Ptprc
Wnk12	1.36E-14	1.28621153	0.933	0.928	2.53E-10	11	Wnk1
Iqgap13	4.74E-14	1.23029138	0.9	0.967	8.81E-10	11	Iqgap1
Ctss3	8.50E-14	1.3643876	0.95	0.981	1.58E-09	11	Ctss
Trim352	3.30E-13	1.14902399	0.85	0.885	6.14E-09	11	Trim35
Wdfy41	7.72E-13	1.06104084	0.883	0.934	1.43E-08	11	Wdfy4
Dennd4a3	2.15E-12	1.47843413	0.85	0.905	4.00E-08	11	Dennd4a
H2-T231	1.82E-11	0.98225934	0.833	0.93	3.38E-07	11	H2-T23
Dock101	3.05E-11	1.27510979	0.817	0.936	5.68E-07	11	Dock10
Pdia31	4.62E-11	1.2681768	0.883	0.958	8.59E-07	11	Pdia3
Calr1	1.04E-10	1.19368709	0.883	0.945	1.93E-06	11	Calr
Ccnl13	1.98E-10	1.00281795	0.883	0.952	3.68E-06	11	Ccnl1
Itgb12	2.09E-10	1.23583112	0.783	0.906	3.88E-06	11	Itgb1
Ctsz3	9.55E-10	0.9033838	0.85	0.966	1.77E-05	11	Ctsz
Ifi305	1.27E-09	1.15537243	0.917	0.951	2.36E-05	11	Ifi30
Itgb72	2.03E-09	1.2432793	0.767	0.887	3.77E-05	11	Itgb7

Picalm2	3.82E-09	0.96500589	0.817	0.932	7.09E-05	11	Picalm
Srrm23	7.03E-09	0.9026128	0.8	0.953	0.00013069	11	Srrm2
Tmed91	1.76E-08	1.32924503	0.717	0.831	0.00032647	11	Tmed9
Canx2	7.03E-08	1.34874334	0.717	0.822	0.00130674	11	Canx
Ahnak3	8.23E-08	1.03645563	0.867	0.951	0.00152936	11	Ahnak
Jmjd1c1	8.49E-08	1.36184289	0.7	0.762	0.00157743	11	Jmjd1c
Ranbp23	1.86E-07	1.2483139	0.683	0.758	0.00346283	11	Ranbp2
H2-K12	4.50E-07	0.79162138	0.883	0.992	0.00835355	11	H2-K1
Grn2	5.44E-07	1.37078447	0.667	0.776	0.01011508	11	Grn
Man2b12	6.31E-07	1.15639384	0.733	0.925	0.01171942	11	Man2b1
Mpeg12	9.01E-07	0.86139846	0.85	0.975	0.01674086	11	Mpeg1
Ahr2	2.26E-06	1.44113934	0.667	0.767	0.04190641	11	Ahr
Fus1	2.32E-06	0.77170482	0.733	0.911	0.04311898	11	Fus
Luc7l21	3.82E-06	1.3018541	0.667	0.804	0.07088273	11	Luc7l2
Ctsh2	4.05E-06	1.16250914	0.7	0.883	0.07518302	11	Ctsh
Skil3	4.84E-06	0.95451577	0.733	0.888	0.08995764	11	Skil
Prrc2c1	5.26E-06	1.08883633	0.683	0.846	0.09780868	11	Prrc2c
Cmtr11	5.91E-06	1.25450509	0.633	0.675	0.10985488	11	Cmtr1
H2-Eb12	7.80E-06	0.47044688	1	0.999	0.14486483	11	H2-Eb1
H2-D12	7.91E-06	0.64564738	0.983	0.998	0.14692404	11	H2-D1
Atp1a11	8.98E-06	0.83938512	0.717	0.859	0.16692005	11	Atp1a1
Dpp41	1.18E-05	1.37641606	0.65	0.8	0.21933474	11	Dpp4
Lcp14	1.23E-05	0.75804203	0.833	0.971	0.22890549	11	Lcp1

Syngr22	1.50E-05	0.69856029	0.8	0.958	0.27830746	11	Syngr2
Dnajb111	1.77E-05	1.31497766	0.617	0.676	0.32937174	11	Dnajb11
Itgb21	2.01E-05	0.92267488	0.717	0.855	0.37376281	11	Itgb2
Tln1	2.37E-05	0.84210707	0.733	0.931	0.44057845	11	Tln1
Erp292	2.46E-05	0.79620334	0.767	0.955	0.45660531	11	Erp29
Atp2b12	2.49E-05	1.12881837	0.683	0.844	0.46298098	11	Atp2b1
Rab8b3	2.65E-05	0.76922363	0.717	0.876	0.49261847	11	Rab8b
Eif4ebp2	3.51E-05	0.20301327	0.133	0.483	0.65150032	11	Eif4ebp2
Ckap4	4.04E-05	0.96367265	0.217	0.085	0.75151424	11	Ckap4
U2surp	4.73E-05	0.23427175	0.217	0.651	0.87798187	11	U2surp
Mbnl12	4.92E-05	0.52884505	0.833	0.963	0.91385595	11	Mbnl1
Uvrag2	5.03E-05	1.16832189	0.65	0.813	0.93485878	11	Uvrag
Ccr52	5.11E-05	1.4085693	0.417	0.285	0.94870413	11	Ccr5
Rpn21	5.32E-05	1.56006398	0.583	0.633	0.98929652	11	Rpn2
Tra2b2	5.42E-05	0.74278442	0.733	0.94	1	11	Tra2b
Yy1	5.60E-05	0.23712323	0.233	0.681	1	11	Yy1
Fam173a	7.21E-05	0.30045959	0.133	0.468	1	11	Fam173a
U2af2	7.61E-05	0.24087437	0.167	0.534	1	11	U2af2
H2-Aa2	7.89E-05	0.34838302	1	0.999	1	11	H2-Aa
Mar-22	8.27E-05	0.23262661	0.183	0.561	1	11	Mar-02
Stard31	8.39E-05	0.21599953	0.1	0.4	1	11	Stard3
Peli14	9.46E-05	0.20814422	0.217	0.619	1	11	Peli1
Metrnl1	0.00011817	0.20548308	0.15	0.49	1	11	Metrnl

H2-Ab1	0.00011967	0.50579022	1	0.997	1	11	H2-Ab1
Kit2	0.00012088	1.40627393	0.583	0.636	1	11	Kit
Nav12	0.00012332	0.25634119	0.183	0.553	1	11	Nav1
Sdf41	0.00012772	0.26607431	0.217	0.626	1	11	Sdf4
Hk23	0.00013682	1.44430052	0.6	0.654	1	11	Hk2
Ifngr11	0.00013838	0.83789736	0.717	0.869	1	11	Ifngr1
Gpr183	0.00014045	1.4643921	0.617	0.622	1	11	Gpr183
Rsbn1l	0.00014049	0.22965614	0.167	0.516	1	11	Rsbn1l
Xist3	0.00014757	1.75928478	0.6	0.674	1	11	Xist
Mbnl22	0.00015853	1.10633222	0.65	0.826	1	11	Mbnl2
Emilin22	0.00016603	1.18549028	0.25	0.119	1	11	Emilin2
P2ry102	0.00017417	0.95878834	0.667	0.839	1	11	P2ry10
Clk13	0.00018833	0.83221629	0.717	0.904	1	11	Clk1
Tomm401	0.00019046	0.23013562	0.15	0.479	1	11	Tomm40
Rbmxl1	0.00020865	0.25207851	0.15	0.478	1	11	Rbmxl1
Dleu22	0.00023233	1.064162	0.617	0.712	1	11	Dleu2
Psmd1	0.00024564	0.23455871	0.167	0.501	1	11	Psmd1
Timm17a1	0.00025152	0.22085397	0.15	0.471	1	11	Timm17a
Ppp4r3b1	0.0002526	0.20647034	0.183	0.537	1	11	Ppp4r3b
Ppp1r14b1	0.00027375	0.20828862	0.233	0.641	1	11	Ppp1r14b
Efr3a1	0.00028209	0.26889502	0.183	0.534	1	11	Efr3a
Tmem1671	0.00028499	0.21981305	0.283	0.747	1	11	Tmem167
Nars1	0.00031611	1.08264849	0.65	0.867	1	11	Nars

Stap11	0.00033912	0.3148598	0.083	0.337	1	11	Stap1
2210016F16Rik	0.00034844	0.26801824	0.133	0.432	1	11	2210016F16Rik
Snrpa12	0.00035019	0.30375357	0.217	0.597	1	11	Snrpa1
Nktr2	0.0003805	1.17695937	0.583	0.699	1	11	Nktr
Csf2rb4	0.00039224	1.32293175	0.6	0.703	1	11	Csf2rb
Slc38a11	0.00040358	1.21086808	0.633	0.762	1	11	Slc38a1
Rpf1	0.00041378	0.30121775	0.133	0.426	1	11	Rpf1
Asap12	0.00041466	0.42218322	0.3	0.761	1	11	Asap1
Iqgap22	0.00041482	1.21573193	0.55	0.613	1	11	Iqgap2
Arfrp1	0.00042669	0.23697922	0.083	0.331	1	11	Arfrp1
Bcl7c	0.00045546	0.20281911	0.183	0.52	1	11	Bcl7c
Havcr23	0.0004631	0.99098881	0.6	0.697	1	11	Havcr2
2900097C17Rik1	0.00046632	0.22860585	0.15	0.455	1	11	2900097C17Rik
Pld41	0.0004722	0.83920704	0.667	0.895	1	11	Pld4
Nup982	0.00047851	1.29181725	0.55	0.603	1	11	Nup98
Rap2a3	0.00049635	0.25046511	0.1	0.355	1	11	Rap2a
Derl11	0.00050202	0.24795213	0.233	0.622	1	11	Derl1
Ltv1	0.00050792	0.21142784	0.083	0.327	1	11	Ltv1
Tbc1d11	0.00051246	0.20414356	0.1	0.357	1	11	Tbc1d1
Rasgrp41	0.00054623	0.23715272	0.217	0.584	1	11	Rasgrp4
Twistnb	0.00056572	0.22411015	0.2	0.553	1	11	Twistnb
Phf14	0.00057107	0.2228406	0.117	0.387	1	11	Phf14
Gorasp21	0.00057464	0.24817337	0.117	0.387	1	11	Gorasp2

Abcg13	0.00059179	0.26444319	0.1	0.353	1	11	Abcg1
Actn41	0.00060221	0.22813799	0.15	0.448	1	11	Actn4
Phactr21	0.00062677	0.42215124	0.167	0.484	1	11	Phactr2
Mef2c2	0.00066847	1.50505788	0.533	0.57	1	11	Mef2c
Akirin2	0.00067109	0.21124312	0.067	0.289	1	11	Akirin2
Bin1	0.00068741	0.28187312	0.2	0.548	1	11	Bin1
Psmd11	0.00072287	0.41484762	0.217	0.582	1	11	Psmd11
Orai1	0.00072434	0.2131256	0.317	0.782	1	11	Orai1
Cysltr11	0.0007755	0.3274226	0.183	0.507	1	11	Cysltr1
Dhps	0.00079601	0.31298877	0.133	0.408	1	11	Dhps
Ece11	0.00079605	0.93709645	0.617	0.808	1	11	Ece1
B230219D22Rik	0.00081906	0.31006303	0.2	0.544	1	11	B230219D22Rik
Rab24	0.00086252	0.30515143	0.183	0.51	1	11	Rab24
Xbp12	0.00088852	1.16844494	0.6	0.747	1	11	Xbp1
Cebpz1	0.00088939	1.1280075	0.617	0.792	1	11	Cebpz
Tnip2	0.00089019	0.31409062	0.133	0.405	1	11	Tnip2
Acer32	0.00090037	0.29657671	0.217	0.568	1	11	Acer3
Dhrs1	0.00091322	0.26184115	0.117	0.376	1	11	Dhrs1
Cirbp	0.00092862	0.20662671	0.183	0.5	1	11	Cirbp
Nucb21	0.00093681	0.2996387	0.183	0.485	1	11	Nucb2
Mrpl55	0.00094297	0.30928579	0.1	0.344	1	11	Mrpl55
Eloa	0.00096018	0.21869051	0.2	0.532	1	11	Eloa
Me2	0.00097592	0.22187139	0.267	0.679	1	11	Me2

Pag1	0.00098479	0.93551298	0.217	0.106	1	11	Pag1
Adprh1	0.00099406	0.34542743	0.167	0.472	1	11	Adprh
Slc12a6	0.00099514	0.93856708	0.25	0.135	1	11	Slc12a6
Derl21	0.00100406	0.21395363	0.183	0.5	1	11	Derl2
Chmp1b	0.00101415	0.26511629	0.133	0.402	1	11	Chmp1b
Tra2a1	0.00109046	0.92542451	0.617	0.81	1	11	Tra2a
Crlf31	0.00112618	0.31655743	0.167	0.464	1	11	Crlf3
Lyn2	0.00119777	0.78694097	0.7	0.921	1	11	Lyn
Nmt2	0.00120686	0.2441497	0.067	0.275	1	11	Nmt2
Pura	0.00124848	0.26827932	0.217	0.561	1	11	Pura
Ppfibp22	0.00126934	0.2952403	0.183	0.493	1	11	Ppfibp2
Ap3b1	0.00126951	0.21366213	0.183	0.495	1	11	Ap3b1
Sae1	0.00127874	0.21471685	0.083	0.305	1	11	Sae1
Laptm51	0.00129037	0.57895775	0.833	0.982	1	11	Laptm5
Colgalt12	0.00129782	0.25676238	0.233	0.588	1	11	Colgalt1
Scarb21	0.00139702	0.32479973	0.217	0.563	1	11	Scarb2
Napsa4	0.00141779	0.72024746	0.85	0.965	1	11	Napsa
Ccdc85b	0.00150447	0.3165053	0.117	0.363	1	11	Ccdc85b
Ppp2r5c1	0.0015154	0.31126616	0.217	0.555	1	11	Ppp2r5c
Rars1	0.00155546	0.22559681	0.15	0.426	1	11	Rars
Mydgf1	0.00156426	0.39661428	0.25	0.625	1	11	Mydgf
Clk4	0.00158563	0.48318505	0.15	0.424	1	11	Clk4
Ddb1	0.00158938	0.26287109	0.1	0.328	1	11	Ddb1

Ssh22	0.00159191	1.30165648	0.4	0.326	1	11	Ssh2
Kdelr21	0.00167674	0.34220221	0.15	0.424	1	11	Kdelr2
Abce1	0.00168044	0.32037031	0.133	0.392	1	11	Abce1
Baz1a2	0.00171442	0.83035961	0.7	0.907	1	11	Baz1a
Tmed31	0.00175253	0.24712001	0.267	0.661	1	11	Tmed3
Fam168b1	0.00177836	0.2194454	0.167	0.453	1	11	Fam168b
Unc93b11	0.00178292	0.87174714	0.65	0.922	1	11	Unc93b1
Abi11	0.00178896	0.34774459	0.267	0.664	1	11	Abi1
Nr3c1	0.00183376	0.35021944	0.167	0.448	1	11	Nr3c1
Tmem1472	0.00183894	0.25058611	0.167	0.451	1	11	Tmem147
Trip122	0.00186121	0.28219268	0.217	0.544	1	11	Trip12
Tmem30a1	0.00193763	0.26924941	0.233	0.584	1	11	Tmem30a
Rrp1	0.00193763	0.41409177	0.25	0.625	1	11	Rrp1
Hivep23	0.00195471	1.12819916	0.55	0.624	1	11	Hivep2
Rnps1	0.00204798	0.29857399	0.2	0.518	1	11	Rnps1
Orc4	0.00205708	0.29405641	0.1	0.323	1	11	Orc4
Gfpt11	0.00207573	0.20976279	0.167	0.445	1	11	Gfpt1
Supt161	0.00209045	0.36754301	0.217	0.545	1	11	Supt16
Vrk2	0.00211533	0.27317799	0.183	0.482	1	11	Vrk2
Arl4c2	0.00216584	0.25340672	0.233	0.571	1	11	Arl4c
Sh3gl1	0.00216673	0.2262061	0.15	0.416	1	11	Sh3gl1
Ifngr21	0.00217084	0.34830204	0.233	0.582	1	11	Ifngr2
Top1	0.00220244	0.72527822	0.667	0.908	1	11	Top1

Smarcc1	0.00220647	0.29705046	0.117	0.35	1	11	Smarcc1
5031439G07Rik1	0.00221529	0.44019939	0.267	0.652	1	11	5031439G07Rik
Naa50	0.00226513	0.24563076	0.233	0.577	1	11	Naa50
Usp162	0.00231196	0.36111516	0.183	0.477	1	11	Usp16
Rtf1	0.00231265	0.33929729	0.25	0.621	1	11	Rtf1
Cnpy3	0.00236976	0.26795081	0.183	0.478	1	11	Cnpy3
Ddx171	0.00239137	0.21271541	0.267	0.639	1	11	Ddx17
Txndc162	0.00240001	1.06502155	0.2	0.47	1	11	Txndc16
Dynll21	0.00240206	0.20830587	0.233	0.575	1	11	Dynll2
Add11	0.00240866	0.31249904	0.15	0.413	1	11	Add1
Hspa91	0.00240953	0.42039514	0.183	0.477	1	11	Hspa9
Cast	0.00240997	0.21675379	0.233	0.569	1	11	Cast
Sec24b1	0.00244005	0.23977898	0.117	0.349	1	11	Sec24b
Smarca51	0.00246463	0.23541359	0.317	0.733	1	11	Smarca5
Dnajb93	0.00247072	0.46237527	0.233	0.568	1	11	Dnajb9
Pan32	0.00247697	0.35026455	0.167	0.444	1	11	Pan3
Map3k4	0.00248705	0.27111791	0.167	0.446	1	11	Map3k4
Shoc2	0.0025084	0.23323602	0.133	0.38	1	11	Shoc2
Actr10	0.00253093	0.22315118	0.217	0.543	1	11	Actr10
Nfkbie2	0.00259134	0.25337	0.133	0.376	1	11	Nfkbie
Cd371	0.00260632	0.31735784	0.25	0.617	1	11	Cd37
Phb2	0.00260794	0.37939821	0.317	0.76	1	11	Phb2
Glmp	0.00262529	0.32163252	0.167	0.444	1	11	Glmp

H2-DMa1	0.0026508	0.46236198	0.867	0.974	1	11	H2-DMa
Tmem1891	0.0026874	0.20686718	0.15	0.411	1	11	Tmem189
Polr2l1	0.00273961	0.37678902	0.233	0.555	1	11	Polr2l
Stk101	0.00274416	0.21519687	0.117	0.344	1	11	Stk10
Fam129a2	0.00280827	0.37036898	0.267	0.64	1	11	Fam129a
Immt	0.00285231	0.33971825	0.15	0.407	1	11	Immt
Vps72	0.0029022	0.23948479	0.083	0.284	1	11	Vps72
Herc4	0.00298894	0.21850034	0.05	0.223	1	11	Herc4
Tor1aip23	0.00303361	0.20020141	0.15	0.404	1	11	Tor1aip2
P2ry6	0.00305114	0.23623561	0.133	0.373	1	11	P2ry6
Bag6	0.00310315	0.273777	0.117	0.344	1	11	Bag6
Gna151	0.00311579	0.26106116	0.2	0.502	1	11	Gna15
Stk17b2	0.00314437	0.62628856	0.717	0.894	1	11	Stk17b
Kmt2a2	0.0032044	0.31180976	0.167	0.435	1	11	Kmt2a
Egln2	0.00322416	0.24901771	0.1	0.312	1	11	Egln2
Sft2d1	0.00328512	0.35243536	0.133	0.374	1	11	Sft2d1
Tmem2482	0.00333535	0.21785534	0.133	0.372	1	11	Tmem248
Senp6	0.00334698	0.23796769	0.233	0.565	1	11	Senp6
Uba51	0.00339445	0.25294952	0.133	0.371	1	11	Uba5
Trf1	0.00340461	0.29351303	0.217	0.527	1	11	Trf
Gpbp1l11	0.0034131	0.33620442	0.133	0.372	1	11	Gpbp1l1
Mfsd14a1	0.00343724	0.3551792	0.2	0.502	1	11	Mfsd14a
Synrg1	0.00347459	0.34050501	0.183	0.465	1	11	Synrg

Setd71	0.00347986	0.33829528	0.05	0.219	1	11	Setd7
Thoc2	0.00354693	0.29608573	0.233	0.561	1	11	Thoc2
Ormdl21	0.00356958	0.22833847	0.133	0.371	1	11	Ormdl2
Mapre22	0.00358968	0.23021869	0.15	0.401	1	11	Mapre2
Chp11	0.00362552	0.39226048	0.15	0.401	1	11	Chp1
Gsap1	0.00362722	0.21691589	0.117	0.339	1	11	Gsap
Sgk12	0.00363306	0.30548228	0.2	0.485	1	11	Sgk1
Smad4	0.0036669	0.32503786	0.117	0.339	1	11	Smad4
Esco1	0.00366752	0.25437242	0.133	0.368	1	11	Esco1
Itgal2	0.00371423	0.24687724	0.25	0.585	1	11	Itgal
Arid1a1	0.00375108	0.33670521	0.233	0.56	1	11	Arid1a
Pigx	0.00401961	0.22643551	0.15	0.399	1	11	Pigx
Tmbim62	0.00406712	0.70389317	0.667	0.937	1	11	Tmbim6
Fmnl21	0.00408393	0.28141441	0.217	0.526	1	11	Fmnl2
Srrt	0.00410094	0.20265886	0.167	0.43	1	11	Srrt
Nipa21	0.00416052	0.20634944	0.217	0.522	1	11	Nipa2
Dhx151	0.00416282	0.23810239	0.3	0.702	1	11	Dhx15
Ddx24	0.00424434	0.36658567	0.3	0.697	1	11	Ddx24
Ddx41	0.0042454	0.22266591	0.167	0.428	1	11	Ddx41
Nipbl1	0.00431848	0.30431857	0.25	0.589	1	11	Nipbl
Tex261	0.00436918	0.25701182	0.183	0.46	1	11	Tex261
Supt201	0.00437141	0.29587722	0.233	0.557	1	11	Supt20
Impdh2	0.00442114	0.28777724	0.25	0.589	1	11	Impdh2

Lrrc581	0.00446636	0.20903643	0.167	0.426	1	11	Lrrc58
Nsf1	0.00448801	0.3631804	0.167	0.428	1	11	Nsf
Setd21	0.00449162	0.30196919	0.15	0.395	1	11	Setd2
Slc25a241	0.00453205	0.27645553	0.133	0.365	1	11	Slc25a24
Pak1ip1	0.00456764	0.2116827	0.283	0.656	1	11	Pak1ip1
Zfx	0.00469823	0.30022187	0.15	0.395	1	11	Zfx
Rapgef61	0.00473199	0.28593493	0.183	0.455	1	11	Rapgef6
Kat6a1	0.00479776	0.22462037	0.2	0.489	1	11	Kat6a
Wbp2	0.00481531	0.25107224	0.1	0.301	1	11	Wbp2
Hook31	0.00481548	0.20662187	0.2	0.487	1	11	Hook3
Usp14	0.00495148	0.34907447	0.15	0.392	1	11	Usp14
Lrrc1	0.00495311	0.24053899	0.117	0.33	1	11	Lrrc1
Tmco11	0.00506403	0.60697693	0.3	0.712	1	11	Tmco1
Itpk11	0.00509162	0.23631799	0.1	0.299	1	11	Itpk1
Bdp11	0.00510516	0.22589183	0.183	0.449	1	11	Bdp1
Sf3b12	0.00511386	0.62753263	0.683	0.95	1	11	Sf3b1
Bcap29	0.00514433	0.20080032	0.183	0.452	1	11	Bcap29
Tep11	0.00518915	0.24020304	0.233	0.552	1	11	Tep1
Khdc41	0.00520573	0.31924786	0.1	0.298	1	11	Khdc4
Usp381	0.00523073	0.21154708	0.1	0.299	1	11	Usp38
Edem21	0.00531241	0.34076442	0.117	0.328	1	11	Edem2
Macf13	0.00534381	1.21077238	0.5	0.554	1	11	Macf1
Ebp2	0.00536667	0.38053763	0.217	0.52	1	11	Ebp

Tap21	0.00553067	1.05020901	0.6	0.827	1	11	Tap2
Gcnt23	0.0056009	0.64131306	0.633	0.737	1	11	Gcnt2
Dck2	0.00566207	0.207728	0.217	0.509	1	11	Dck
Gtpbp4	0.00575344	0.32817873	0.3	0.705	1	11	Gtpbp4
Ptpa1	0.00579697	0.34630546	0.217	0.518	1	11	Ptpa
Coro1c1	0.0058461	0.20228223	0.167	0.416	1	11	Coro1c
Rnmt	0.00589383	0.20466867	0.1	0.294	1	11	Rnmt
Cnot2	0.00593579	0.21462935	0.117	0.325	1	11	Cnot2
Vps41	0.00594089	0.31863769	0.133	0.356	1	11	Vps41
Cpq	0.00595218	0.23753063	0.15	0.387	1	11	Cpq
Smchd12	0.00597237	0.9809678	0.583	0.74	1	11	Smchd1
Rab211	0.00605649	0.23315516	0.15	0.382	1	11	Rab21
Dnajc51	0.00616268	0.39425767	0.167	0.418	1	11	Dnajc5
Trim30d2	0.00620184	0.20323084	0.217	0.51	1	11	Trim30d
Agps	0.00623013	0.20089496	0.1	0.293	1	11	Agps
Ube3a1	0.00632972	0.2695122	0.217	0.506	1	11	Ube3a
Lypla21	0.00637434	0.21944667	0.217	0.511	1	11	Lypla2
Asah11	0.00638943	1.15392108	0.567	0.774	1	11	Asah1
Sirt7	0.00640918	0.20758582	0.233	0.546	1	11	Sirt7
Larp4b1	0.0064258	0.36313417	0.233	0.542	1	11	Larp4b
Ddrgk1	0.00648588	0.36823569	0.217	0.514	1	11	Ddrgk1
Map41	0.00653003	0.39253408	0.133	0.35	1	11	Map4
Tmem2141	0.00661533	0.26071732	0.133	0.353	1	11	Tmem214

H2-DMb11	0.00665965	0.4644587	0.867	0.97	1	11	H2-DMb1
Rbbp83	0.00668654	0.21648287	0.25	0.569	1	11	Rbbp8
Fbxo112	0.00669007	0.37788601	0.2	0.48	1	11	Fbxo11
Ddx52	0.00671774	0.20561789	0.133	0.352	1	11	Ddx52
Zfp1481	0.00702243	0.36203556	0.133	0.349	1	11	Zfp148
Selenot2	0.00703554	0.39335217	0.233	0.551	1	11	Selenot
Emc10	0.00707646	0.3501072	0.217	0.509	1	11	Emc10
Brd41	0.00710138	0.45853532	0.267	0.611	1	11	Brd4
Smarcad1	0.00711359	0.23250302	0.117	0.32	1	11	Smarcad1
Gatad2a	0.00718702	0.32736169	0.217	0.51	1	11	Gatad2a
CAAA01118383.1	0.00724096	0.34751356	0.167	0.41	1	11	CAAA01118383.1
Sgpl12	0.00724478	0.2054559	0.183	0.442	1	11	Sgpl1
Cblb1	0.00733749	0.22390948	0.15	0.374	1	11	Cblb
Ctbp1	0.00737011	0.30860653	0.183	0.446	1	11	Ctbp1
Sde22	0.00739188	0.43202573	0.25	0.581	1	11	Sde2
Preb1	0.00747222	0.32531741	0.183	0.443	1	11	Preb
Hlcs	0.00748219	0.23130418	0.083	0.258	1	11	Hlcs
Gnl2	0.0075133	0.20205602	0.083	0.258	1	11	Gnl2
Chtop	0.00752347	0.37339952	0.183	0.446	1	11	Chtop
Tor3a2	0.00759005	0.27859518	0.117	0.318	1	11	Tor3a
Zmiz12	0.00765229	1.23223106	0.533	0.663	1	11	Zmiz1
Midn3	0.00770796	0.20490664	0.217	0.503	1	11	Midn
Rbck1	0.00774069	0.33586523	0.2	0.478	1	11	Rbck1

Clpp	0.0077876	0.26326514	0.083	0.258	1	11	Clpp
Eif4e1	0.00781083	0.76686407	0.267	0.615	1	11	Eif4e
Dnm1l	0.0078579	0.24052509	0.167	0.409	1	11	Dnm1l
1700025G04Rik1	0.00788397	0.36464229	0.267	0.615	1	11	1700025G04Rik
Dennd4b2	0.00804436	0.32657426	0.133	0.347	1	11	Dennd4b
Pabpc13	0.00817351	0.4735987	0.8	0.979	1	11	Pabpc1
Ctsa1	0.00818158	0.21549924	0.133	0.345	1	11	Ctsa
Ric8a1	0.00818927	0.3275501	0.183	0.442	1	11	Ric8a
Ppan	0.00837756	0.24996956	0.1	0.286	1	11	Ppan
Xcr13	0.0084039	0.37967954	0.35	0.74	1	11	Xcr1
Ppib3	0.00850012	0.46412759	0.783	0.956	1	11	Ppib
Zc3h15	0.00853853	0.27017748	0.317	0.701	1	11	Zc3h15
Stx121	0.00857102	0.2890992	0.133	0.345	1	11	Stx12
Ppp1r103	0.00858488	0.27664501	0.233	0.527	1	11	Ppp1r10
Hspa41	0.00859862	0.39486726	0.333	0.749	1	11	Hspa4
Luc7l3	0.00866958	0.34399943	0.25	0.566	1	11	Luc7l3
Mtch2	0.00868377	0.27004545	0.183	0.441	1	11	Mtch2
Dnajc21	0.00880371	0.22893279	0.117	0.313	1	11	Dnajc21
Maz	0.00881803	0.33294858	0.15	0.376	1	11	Maz
Sec62	0.00890249	1.10372834	0.583	0.814	1	11	Sec62
Rpn12	0.00901905	1.15990213	0.533	0.675	1	11	Rpn1
Ubp1	0.00925738	0.35861267	0.15	0.373	1	11	Ubp1
Cd472	0.00931634	0.37715543	0.8	0.98	1	11	Cd47

Arhgap51	0.00935934	0.24200522	0.267	0.598	1	11	Arhgap5
Comt	0.00939398	0.25939977	0.133	0.341	1	11	Comt
H2-DMb2	0.00942806	0.33209146	0.2	0.465	1	11	H2-DMb2
Gps1	0.00959337	0.33805034	0.133	0.343	1	11	Gps1
Cd82	0.00977724	0.25394916	0.233	0.53	1	11	Cd82
Atraid	0.00984052	0.20398811	0.15	0.372	1	11	Atraid
Fnbp12	0.00984801	0.56932179	0.75	0.958	1	11	Fnbp1
Mpp7	0.00999395	1.44926311	0.417	0.396	1	11	Mpp7

Table S2

Antibodies used for FFPE staining

Order of staining	Target	Catalogue no	supplier	Primary dilution	Secondary	Catalogue no	Supplier	Secondary dilution	Opal	Opal dilution	Antigen retrieval/pre-treatment & stripping conditions
1	TCF1	2203T	CST	1:100	*Novolink Polymer*	*RE7200-CE*	*Leica*	RTU	520	520 1:500	pH6 20min 95˚C
2	mCherry	ab125096	Abcam	1:400	biotinylated anti-mouse + Streptavidin HRP	BA-2001 P0397	Vector Dako	1:400 + 1:500	570	570 1:500	
3	CD8	ab217344	Abcam	1:500	*Novolink Polymer*	*RE7200-CE*	*Leica*	RTU	690	690 1:200	
1	TCF1	2203T	CST	1:100	*Novolink Polymer*	*RE7200-CE*	*Leica*	RTU	520	520 1:500	pH6 20min 95˚C
2	mCherry	ab125096	Abcam	1:400	biotinylated anti-mouse + Streptavidin HRP	BA-2001 P0397	Vector Dako	1:400 + 1:500	570	570 1:500	
3	CD4	ab183685	Abcam	0.5625	*Novolink Polymer*	*RE7200-CE*	*Leica*	RTU	690	690 1:200	
1	CD31	AF3628	R&D	1:50	Immpress HRP anti Goat	MP-7405-50	Vector	RTU	480	1:300	pH9 20min 95˚C
2	GFP	ab6673	Abcam	1:300	Immpress HRP anti Goat	MP-7405-50	Vector	RTU	520	1:300	
3	CD8	ab217344	Abcam	1:500	*Novolink Polymer*	*RE7200-CE*	*Leica*	RTU	690	1:150	
4	mCherry	NBP225157	Bio-techne	1:1000	*Novolink Polymer*	*RE7200-CE*	*Leica*	RTU	570	1:300	
1	Cxcl9	Probe	ACD	RTU					570	1:1000	FFPE: pH9 15min 95˚C + 15min protease 37˚C<br>Fix Fz: pH9 5min 88˚C + 10min protease RT
2	Il2b	Probe	ACD	1:50					690	1:1000	
3	CD31	AF3628	R&D	1:50	Immpress HRP anti Goat	MP-7405-50	Vector	RTU	480	1:400	pH6 20min 95˚C
4	GFP	ab6673	Abcam	1:300	Immpress HRP anti Goat	MP-7405-50	Vector	RTU	520	1:500	
5	CD8	ab217344	Abcam	1:500	*Novolink Polymer*	*RE7200-CE*	*Leica*	RTU	780	1:200 + 1:50	

Antibodies used for Fixed Frozen IF staining

**Target**	**Clone**	**Dilution**	**Catalogue #**
Rabbit anti-mouse CD8a AF647 (EPR21769)	Abcam	1:100	EPR21769
Rat anti-mouse endomucin unconjugated (V.7C7)	Santa Cruz	1:100	sc-65495
Goat anti RFP (Goat Polyclonal)	BioServ UK Ltd (Rockland)	1:500	200-101-379S
TCF1/TCF7 (C63D9) Rabbit mAb #2203	Cell Signaling Technology	1:100	2203T
Rat anti-mouse I-A/I-E AF594 (clone M5/114.15.2)	Biolegend	1:100	107650
Brilliant Violet 570™ anti-mouse CD45.1 Antibody	Biolegend	1:50	110733
Anti-goat AF594	Thermo Fisher	1:400	A32758
Anti-rabbit AF647	Thermo Fisher	1:400	A32795
Anti-rat AF405	Thermo Fisher	1:400	A48268
Anti-mouse IgG2a AF594	Thermo Fisher	1:400	A21135

Antibodies used for Flow cytometry Staining

**Target**	**Colour**	**Brand**	**Cat #**
B220	FITC	BD biosciences	CAT#553088
B220	ef450	ThermoFisher	CAT#48-0452-82
CCR7	PeCy7	Biolegend	CAT#120123

CD11b	BUV395	BD biosciences	CAT#565976
CD11c	BUV805	BD biosciences	CAT#749038
CD11c	BV421	Biolegend	CAT#117343
CD135	Percp ef10	ThermoFisher	CAT#46-1351-82
CD16/32	purified	BD biosciences	CAT#553142
CD19	V450	BD biosciences	CAT#560353
CD19	FITC	Biolegend	CAT#152404
CD25	FITC	BD biosciences	CAT#553072
CD3e	BUV805	BD biosciences	CAT#741928
Cd3e	FITC	Biolegend	CAT#100306
CD40	BUV661	BD biosciences	CAT# 121218
CD43	PE-Cy7	Biolegend	CAT# 121218
CD44	PE-Cy7	ThermoFisher	CAT#25-0441-81
CD45	BV711	Biolegend	CAT#103147
CD45	APC-eFluor 780	ThermoFisher	CAT#47-0451-82
CD45.1	AF647	Biolegend	CAT#110720
CD45.1	PE	Biolegend	CAT#110708
CD45.2	BUV661	Biolegend	CAT#741516
CD62L	BUV395	BD biosciences	CAT#740218
CD69	BV605	Biolegend	CAT#104529
CD80	PE	BD biosciences	CAT#553769
CD86	BV786	Biolegend	CAT#105043
CD88	BUV563	BD biosciences	CAT#748613
CD88	APC	Biolegend	CAT#135808
Cd8a	APC Fire750	Biolegend	CAT#100766
CXCL9	AF647	Biolegend	CAT#515606
F4/80	BUV563	BD biosciences	CAT#749284
IL-12p40	FITC	BD biosciences	CAT#560564
Ly6D	FITC	Biolegend	CAT#138605
Ly6D	eF450	ThermoFisher	CAT#48-5974-80
Ly6G	FITC	Biolegend	CAT#127605
Ly6G	BV421	Biolegend	CAT#127627
MHCII	AF700	ThermoFisher	CAT#56-5321-82
NK1.1	FITC	Biolegend	CAT#108705
NK1.1	PB	Biolegend	CAT#108721
PD-1	BV786	Biolegend	CAT#135225
PD-L1	BV421	BD biosciences	CAT#564716
SiglecF	FITC	BD biosciences	CAT#155503
SiglecF	BV421	BD biosciences	CAT#562681
Sirpa	BV510	ThermoFisher	CAT# 740159
TCRb	APC	BD biosciences	CAT#553174
TCRb	BV786	Biolegend	CAT#109249

TER119	FITC	Biolegend	CAT#116205
TER119	PB	Biolegend	CAT#116231
Tim3	BV711	Biolegend	CAT#119706
XCR1	BV650	Biolegend	CAT#148220
